# A Molecular Hypothesis on Malignant Transformation of Oral Lichen Planus: A Systematic Review and Meta-Analysis of Cancer Hallmarks Expression in This Oral Potentially Malignant Disorder

**DOI:** 10.3390/cancers16152614

**Published:** 2024-07-23

**Authors:** Carmen Keim-del Pino, Pablo Ramos-García, Miguel Ángel González-Moles

**Affiliations:** 1 School of Dentistry, University of Granada, 18071 Granada, Spain; carmenkeim@correo.ugr.es; 2 Biohealth Research Institute (Ibs.GRANADA), 18012 Granada, Spain

**Keywords:** oral lichen planus, oral cancer, hallmarks of cancer, oral potentially malignant disorders, systematic review, meta-analysis

## Abstract

**Simple Summary:**

Oral lichen planus (OLP) is a chronic inflammatory disease of autoimmune nature and unknown etiology which affects approximately 1% of the world’s population. The most important feature of OLP is its behavior as an oral potentially malignant disorder (OPMD). The current study is the first systematic review and meta-analysis designed to evaluate the degree of existing scientific evidence on the cancer hallmarks proposed in 2011 by Hanahan and Weinberg, defined as the characteristics that cells must fulfill in order to be considered neoplastic cells in all types of tumors that affect humans. This systematic review and meta-analysis includes 110 studies which recruited 7064 cases of OLP, in which the expression of 104 molecular biomarkers were analyzed through an immunohistochemical technique. The earliest oncogenic molecular mechanisms that could justify the malignant transformation of this disease are analyzed in depth and critically discussed on the basis of evidence.

**Abstract:**

We aimed to qualitatively and quantitatively analyze, through a systematic review and meta-analysis, the current evidence on the differential expression of the hallmarks of cancer in oral lichen planus (OLP) samples, in order to know the earliest molecular mechanisms that could be involved in the malignant transformation of this oral potentially malignant disorder. We searched MEDLINE/PubMed, Embase, Web of Science, and Scopus for studies published before November 2023. We evaluated the methodological quality of studies and carried out meta-analyses to fulfill our objectives. Inclusion criteria were met by 110 primary-level studies, with 7065 OLP samples, in which the expression of 104 biomarkers were analyzed through immunohistochemistry. Most OLP samples showed sustained cell proliferation signaling (65.48%, 95%CI = 51.87–78.02), anti-apoptotic pathways (55.93%, 95%CI = 35.99–75.0), genome instability (48.44%, 95%CI = 13.54–84.19), and tumor-promoting inflammation events (83.10%, 95%CI = 73.93–90.74). Concurrently, OLP samples also harbored tumor growth suppressor mechanisms (64.00%, 95%CI = 53.27–74.12). In conclusion, current evidence indicates that molecular mechanisms promoting hyperproliferative signaling, an antiapoptotic state with genomic instability, and an escape of epithelial cells from immune destruction, are developed in LP-affected oral mucosa. It is plausible that these events are due to the actions exerted by the chronic inflammatory infiltrate. Malignant transformation appears to be prevented by tumor suppressor genes, which showed consistent upregulation in OLP samples.

## 1. Introduction

Oral lichen planus (OLP) is a chronic inflammatory disease of unknown etiology and autoimmune nature that presents with white reticular lesions accompanied or not by atrophic and/or erosive lesions, plaque lesions, and occasionally bullous lesions [1]. It is a common disorder affecting 1.01% of the world’s population, with a clear geographical distribution, Europe being the continent where the disease is most common (1.43%); the prevalence of OLP increases significantly and progressively from the age of 40 years [2]. The most relevant aspect of the disease lies in its current consideration as an oral potentially malignant disorder (OPMD) which, despite the controversies that this has generated [3], has been ratified in the last consensus meeting (Glasgow 2020) of the international group of experts selected by the WHO Collaborating Centre for the Study of Oral Cancer (King’s College-London) [4]. The current consideration of OLP as an OPMD is based on some recently published evidence in the form of systematic reviews and meta-analyses that figure its malignancy rate between 0.44% and 2.28% of cases [5,6,7,8,9,10,11,12]. To date, 97 papers on malignant transformation of OLP have been published, presenting data on a total of 36,889 patients [11], indicating the growing interest in this aspect of OLP and reflecting that the current consideration of the disease as an OPMD derives from solid evidence-based information. Accepting that some patients with OLP will develop oral cancer during their lifetime implies a radical change in clinical practice that requires the establishment of measures, essentially related to prevention and patient follow-up, tending to reduce their malignancy rate and to achieve early diagnosis of cancer in order to improve survival.

At present, the molecular mechanisms involved in the malignant transformation of OLP are unknown, although presumably they could be associated with the oncogenic aggressions exerted by the inflammatory infiltrate on the basal and parabasal cells of the oral epithelium, as occurs in other autoimmune diseases [3,13]. The results of a research line developed in recent years by our group, essentially applying immunohistochemical techniques [14,15,16,17,18,19,20,21], seem to indicate—in summary terms—that oral mucosa affected by lichen planus responds with a hyperproliferative state and with the establishment of molecular antiapoptotic mechanisms which, hypothetically, could prevent or minimize the development of a hyperproliferative state and with the establishment of molecular antiapoptotic mechanisms which, hypothetically, could prevent or minimize the collapse of the epithelium mediated by autoimmune aggression, which would represent the most severe form of the clinical spectrum of the disease—atrophic-erosive OLP. Presumably, the expected consequence of this type of hyperproliferative response to autoimmune aggression would be the establishment of genomic instability that would increase the risk of acquiring summative oncogenic events and would favor the development and expansion of fields harboring molecular alterations promoting the appearance of malignant clones; on the other hand, a compensatory mechanism that could justify the low malignancy rate of OLP compared to other OPMD could come from the establishment of antitumor surveillance phenomena mediated by tumor suppressor genes, which in our series are frequently expressed [21,22], whose failure could culminate in malignancy, perhaps with the help of other carcinogenic factors—it has been reported that OLP malignancy is significantly higher in smokers and drinkers [5,11,12,23]. Our hypothesis of malignant OLP transformation is based on the multistep model of oral carcinogenesis [24] according to which, and as a consequence of genomic instability mediated by a cellular hyperproliferative state, cell clones acquire additive oncogenic capacities that will eventually endow them with the distinctive hallmarks of neoplastic cells. The characteristics that neoplastic cells should exhibit to be considered as such—hallmarks of cancer—were initially proposed in 2000 by Hanahan and Weinberg [25] who pointed out that these are acquired over a multistage process, from the earliest, even premalignant stages, to those in which the cancer has become fully established. The authors’ final proposal in 2011 [26] included six hallmarks (sustaining proliferative signaling, evading growth suppressors, resisting cell death, enabling replicative immortality, inducing angiogenesis, activating invasion and metastasis), two enabling characteristics (genome instability and mutation, and tumor-promoting inflammation), and two emerging hallmarks (deregulating cellular energetics and avoiding immune destruction). The main consequence of the definition of cancer hallmarks has been that it has helped us to better understand the biopathogenic mechanisms involved in cancer initiation and progression, which would also be applicable to oral carcinogenesis.

Although OLP is a relevant OPMD, there is currently no evidence-based knowledge about which hallmarks of cancer are expressed in oral epithelium affected and, consequently, we lack a plausible approximation of the earliest molecular events that operate in their malignant transformation. For this reason, we set out to perform the present systematic review and meta-analysis of 110 studies, which focuses on the analysis of the expression of cancer hallmarks in 7065 samples of oral epithelium affected by OLP, which aims to know, based on the evidence, which are the earliest oncogenic molecular mechanisms that could justify the malignant transformation of this disease, which could perhaps favor the future establishment of preventive strategies for the development of cancer in this disorder.

## 2. Materials and Methods

With the aim to approach this systematic review and meta-analysis, MOOSE and PRISMA reporting guidelines [27,28] were embraced. Likewise, standard methodological criteria were chosen from Cochrane [29] and Joanna Briggs Institute Organization (University of Adelaide, Australia) [30] to comply with the appropriate design of our work.

### 2.1. Protocol

At first, a protocol was carried out and submitted in a widely recognized database which assembles data of prospectively registered systematic reviews (*PROSPERO*; www.crd.york.ac.uk/PROSPERO (accessed on 21 February 2024); registration code ID-511457/CRD42024511457) with the purpose of decreasing the risk of bias (RoB) and upgrading the transparency, accuracy, and integrity of our systematic review and meta-analysis. Furthermore, our protocol was lean on PRISMA-P statement, ensuring thereby its strict assent [31].

### 2.2. Search Strategy

We searched MEDLINE (via PubMed), Embase, Web of Science, and Scopus databases’ studies published before our upper search limit date (November 2023), without lower date or language limitation. The search strategy was designed by combining databases’ thesaurus (i.e., MeSH and EMTREE) with free terms, using the terms “oral lichen planus”, “malignant transformation” and synonyms (i.e., “Lichen Planus, Oral” [MeSH] or “oral lichen planus” [All Fields] or “olp” [All Fields]) and (malign* or premalign* or “potentially malignant disorder” or “precancer” or “cancer” [All Fields] or “Carcinoma, Squamous Cell” [MeSH] or “squamous cell carcinoma” [All Fields] or “oscc” [All Fields] or “transformation” [All Fields] or “risk” [All Fields] or “progression” [All Fields]); an equivalent syntax was adapted to each consulted database, available in the supplementary information, Table S1, designed with the aim to maximize sensitivity. We have a preference for this approach, which enabled us to include a large sample of studies exploring OLP, rather than the design of a more precise strategy (e.g., using more specific terms such as “biomarker” or “ hallmarks of cancer”), since several papers lack biomarkers within their appointed keywords, titles, and abstracts. Furthermore, additional records were screened by handsearching the reference lists of retrieved studies and identified through Google Scholar. Every reference in addition to the elimination of duplicated records were managed by Mendeley v.1.19.8 (Elsevier, Amsterdam, The Netherlands).

### 2.3. Eligibility Criteria

Inclusion criteria: (1) Primary-level studies, without restrictions by publication language or date; a primary-level study was defined as an original epidemiological individual study generating new data, from a recruited sample of participants, through the implementation of the scientific method. These studies constitute the most relevant source of raw data for secondary research for evidence synthesis, e.g., systematic reviews and meta-analyses (definition modified from the *National Institute for Health and Care Research, NIHR*); (2) Observational study design; (3) Studies analyzing the relative differential expression of any protein, assessed through immunohistochemistry, in samples from patients with OLP, compared or not with two distinct control groups, i.e., healthy mucosa (from completely healthy patients) or oral cancer tissues (from patients suffering from OSCC). For the acceptation of cases of OLP, we fully respected the diagnostic criteria applied by the authors of primary-level studies, i.e., clinical with/without histological diagnosis, due to the lack of international consensus on universal diagnostic criteria for this disease; and (4) Patients of any age, sex, or geographic area. 

Exclusion criteria: (1) Studies that do not involve protein expression, assessed through immunohistochemistry, in OLP samples; (2) Lichen planus lesions on different anatomical sites or without distinction among oral, cutaneous, or genital lichen planus; (3) Lack of essential statistical data for meta-analyses; (4) Retracted articles, basic research with animals or in vitro, secondary/tertiary-level studies (e.g., scoping, systematic, or umbrella reviews, with or without meta-analyses), case reports, meeting abstracts, editorials, book chapters, letters, medical hypotheses, or personal comments.

### 2.4. Study Selection Process

Eligibility criteria were applied individually by two authors (CKDP and PRG). Evaluators fulfilled the articles’ selection in two phases which consisted, first, on the screening by titles and abstracts to include records that appeared to meet the inclusion criteria; second, the potentially selected articles were read and assessed in detail. The articles that failed to comply with the aforementioned criteria were excluded. Any disparities were solved through consensus.

### 2.5. Data Extraction

One author (CKDP) utilized Excel v.16.53 spreadsheet data collection form (Microsoft, Redmond, WA, USA) to extract information from the selected studies in a standardized manner. The collected data comprehended information on the first author, year of publication, sample size, language and publication date, country, continent, anatomical subsites, clinical type, sex and age of patients, tobacco, areca nut and alcohol consumption, study design, immunohistochemical methods (i.e., antibody, dilution, incubation time, and temperature), cut-off point for positivity and cellular pattern; regarding biomarkers, the number of positive and negative cases which their respective proportions in the different layers of epithelium, lamina propria, and inflammatory infiltrate, biomarker’s biological and oncogenic role allowing to attribute a cancer hallmark to each biomarker, consulting the GENE (https://www.ncbi.nlm.nih.gov/gene), HUGO (https://www.genenames.org/) databases and specific publications on their oncogenic implications in OLP and cancer. Furthermore, number of total and positive cases in healthy controls and OSCC cases were also gathered. 

### 2.6. Appraisal of Quality and Risk of Bias

Joanna Briggs Institute [30,32] methods were implemented to the primary-level studies by one author (CKDP) who critically evaluated the methodological quality and risk of bias (RoB) attending to the following questions, specifically designed for meta-analyses of proportions: (a) “Was the sample representative of the target population?”; (b) “Were the study participants recruited in an appropriate way?”; (c) “Was the sample size adequate?”; (d) “Were the study subjects and the settings described in detail?”; (e) “Was the data analysis conducted with sufficient coverage of the identified sample?”; (f) “Were objectives, standard criteria used for the measurement of the condition?”; (g) “Was the condition measured reliably?”; (h) Was the statistical analysis appropriate?”; (i) “Were all-important confounding factors/subgroups/differences identified and accounted for?”; and (j) “Were subpopulations identified using objective criteria?”. The potential risk of bias was qualified as high RoB, unclear/moderate RoB, or low RoB. Moreover, if a biomarker could not be classified within a given cancer hallmark, it was instead included in a group called “unspecified”. This consideration was considered to be the highest source of potential bias in the present study. Consequently, studies reporting results for these biomarkers were excluded from the meta-analysis as a source of high risk of bias, in order to improve the quality and reliability of conclusions.

### 2.7. Statistical Analysis

The differential expression of biomarkers in OLP samples was estimated by pooled proportions (PP) combined together with their corresponding 95% confidence intervals (CIs). These proportions were first calculated by extracting the raw numerators (number of cases with positive expression) and raw denominators (total number of OLP samples). Hence, 95% CIs were constructed for each individual study using the Wilson score method [33]. Freeman–Tukey double arcsine transformation was implemented to minimize the influence of studies with extreme values (values 0, 100, or close to these) by stabilizing the variance of the specific proportions of each study [34]. Transformed proportions were then meta-analyzed and sequentially backtransformed [35] to finally display pooled proportions (PP), expressed as percentage. The magnitude of association between the expression of biomarkers comparing different groups (i.e., OSCC vs. OLP, OLP vs. healthy oral mucosa, OSCC vs. healthy oral mucosa) was also separately explored estimating and combining odds ratios (ORs) with 95%CI. 

All meta-analyses were performed using random-effects models, weighed by the inverse variance based on the DerSimonian and Laird method [36], in order to account for different underlying results across potential study subpopulations (e.g., differences inherent to the variability of experimental methods, such as different biomarkers, antibodies, cut-off points, etc.) [37]. We constructed forest plots to graphically illustrate the overall effect sizes and subsequent for visual inspection analyses. Heeding the assessment of heterogeneity between studies, Cochran’s Q test, based on Chi-square test, was applied; due to its low statistical power, *p* < 0.10 was assumed as significant. Additionally, the proportion of heterogeneity was quantified by the Higgins’ I^2^ in order to describe the percentage of variability in effect estimates reflected in true effects, instead of sampling error [38,39]. Furthermore, secondary analyses were used to check stability and reliability of meta-analysis results. Stratified meta-analyses were run to appraise potential sources of heterogeneity and to determine subgroups-specific relative frequencies [40]. With the aim to evaluate small-study effects, such as publication bias, funnel plots were constructed and the Egger regression test [41] was also applied (considering a *p_Egger_*-value < 0.10 as significant). All statistical analyses were performed operating with Stata software (version 16.1, Stata Corp, College Station, TX, USA).

## 3. Results

### 3.1. Results of the Literature Search

**Figure 1:**
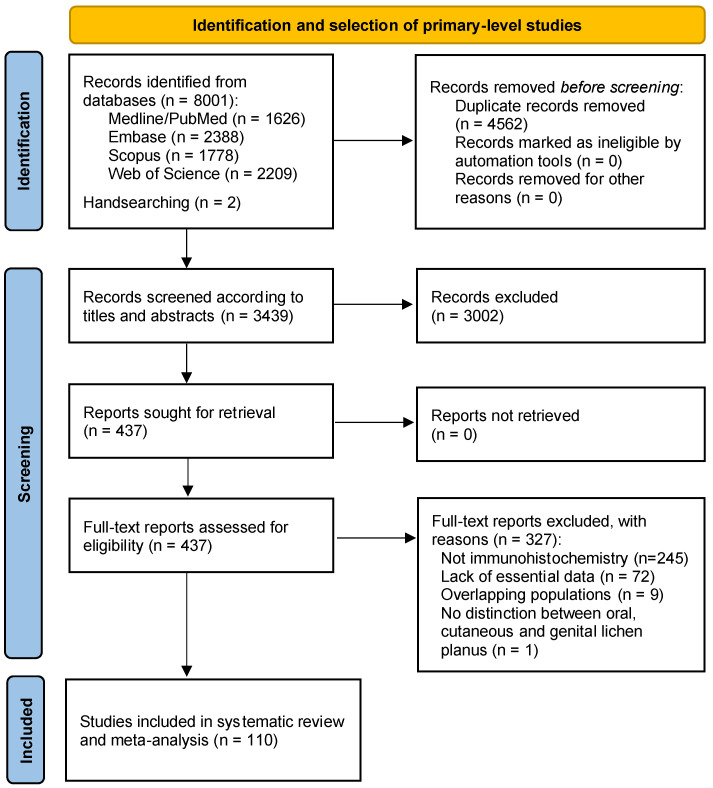
Flow diagram of the process of identification and selection of primary-level studies offering scientific information on the hallmarks of cancer in oral lichen planus samples.

The results that arise from the study selection process are depicted in the flow diagram (Figure 1). A total of 8003 records published before November 2023 were retrieved: 1626 from Medline/PubMed, 2388 from Embase, 2209 from Web of Science, 1778 from Scopus, and 2 through handsearching methods. After the withdrawal of duplicates, 3439 studies were considered to be potentially eligible. Upon being screened according to titles and abstracts, 437 records were assessed in full-text, of which 327 studies did not meet the inclusion criteria. Eventually, 110 studies [17,20,21,42,43,44,45,46,47,48,49,50,51,52,53,54,55,56,57,58,59,60,61,62,63,64,65,66,67,68,69,70,71,72,73,74,75,76,77,78,79,80,81,82,83,84,85,86,87,88,89,90,91,92,93,94,95,96,97,98,99,100,101,102,103,104,105,106,107,108,109,110,111,112,113,114,115,116,117,118,119,120,121,122,123,124,125,126,127,128,129,130,131,132,133,134,135,136,137,138,139,140,141,142,143,144,145,146] were included in the qualitative and quantitative analysis (all included and excluded studies’ references—with their reasons for exclusion—are enumerated in the supplementary information). 

### 3.2. Study Characteristics

The characteristics and variables gathered are exposed thoroughly in Table S2 in the supplementary information. Table 1 outlines the general characteristics of the 110 primary-level studies systematically reviewed, which included 7065 OLP oral mucosa samples, in which the differential protein expression of a total of 104 different biomarkers were analyzed through immunohistochemical techniques (all biomarkers classified by hallmarks of cancer and roles are listed in Table S3). Considering the study countries and continents, 54 studies (8 countries) took place in Asia, 37 studies (13 countries) in Europe, 13 studies (3 countries) in South America, 3 studies (3 countries) in North America, and only one study was included from Oceania.

### 3.3. Qualitative Evaluation

According to our methodological quality and risk of bias analysis across primary-level studies, using the Joanna Briggs Institute tool, not all studies were conducted with the same rigor. As expected, the items Q2, Q9, and Q10 showed the highest risk of potential bias. Considering domain Q2, sampling methods were not employed in most of the primary-level studies (i.e., random recruitment methods, statistical calculation of sample size). Domain Q9 demonstrated primary-level studies to fail at communicating of potentially confounding variables. (i.e., alcohol or tobacco consumption) whist lastly, domain Q10 harbored a lack of subgroups’ identification using objective criteria. (i.e., sex, age, alcohol or tobacco consumption). More importantly, the source of highest risk of bias in this systematic review was considered to be the failure or infeasibility to appropriately classify a particular protein in a specific hallmark. These studies and biomarkers were considered to be of high risk of bias, and consequently excluded from the meta-analysis.

### 3.4. Quantitative Evaluation (Meta-Analysis)

The main quantitative results of our meta-analyses have been reported in Table 2, available in the supplementary information (i.e., Forest plots from Figures S1–S34), and graphically represented in a forest top plot (Figure 2).

#### 3.4.1. Hallmark 1: Sustaining Proliferative Signaling

Differential expression in OLP. The estimated pooled proportion (PP) for pro-proliferative biomarkers was 65.48% (95%CI = 51.87–78.02), with a considerable heterogeneity degree (*I*^2^ = 94.5%, *p* < 0.001).

Magnitude of association between OSCC and OLP. OSCC cases showed a significantly higher frequency for pro-proliferative biomarkers expression than the OLP mucosa samples (OR = 4.39, 95%CI = 2.22–8.71, *p* < 0.001; Table 2, supplementary information). 

Magnitude of association between OLP and healthy controls. Cases with OLP showed a significantly higher frequency for pro-proliferative biomarkers expression than the healthy controls (OR = 2.90, 95%CI = 1.27–6.65, *p* = 0.01; Table 2, supplementary information). 

Magnitude of association between OSCC and healthy controls. OSCC cases showed a significantly higher frequency for pro-proliferative biomarkers expression than the healthy controls (OR = 7.50, 95%CI = 2.58–21.73, *p* < 0.001; Table 2, supplementary information). 

#### 3.4.2. Hallmark 2: Evading Growth Suppressors

Differential expression in OLP. The estimated PP for tumor growth suppressor biomarkers was 63.15% (95%CI = 52.26–73.45), with a high degree of heterogeneity (*I*^2^ = 91.9%, *p* < 0.001).

Magnitude of association between OSCC and OLP. Patients with OSCC showed a significantly higher frequency for tumor growth suppressor biomarkers expression in comparison with the OLP cases (OR = 2.16, 95%CI = 1.26–3.69, *p* = 0.005; Table 2, supplementary information).

Magnitude of association between OLP and healthy controls. OLP samples showed a significantly higher frequency for tumor growth suppressor biomarkers expression than the healthy controls (OR = 11.43, 95%CI = 6.89–18.95, *p* < 0.001; Table 2, supplementary information). 

Magnitude of association between OSCC and healthy controls. Patients with OSCC showed a significantly higher frequency for tumor growth suppressor biomarkers expression than the healthy controls (OR = 19.18, 95%CI = 8.25–44.61, *p* < 0.001; Table 2, supplementary information). 

#### 3.4.3. Hallmark 3: Resisting Cell Death

Differential expression in OLP. The estimated PP for anti-apoptotic biomarkers was 55.93% (95%CI = 35.99–75.01), with a considerable heterogeneity degree (*I*^2^ = 95.0%, *p* < 0.001). In contrast, PP for pro-apoptotic biomarkers was 64.92% (95%CI = 55.15–74.14), with a significant heterogeneity degree (*I*^2^ = 83.8%, *p* < 0.001). 

Magnitude of association between OSCC and OLP. OSCC cases showed a significantly higher frequency of anti-apoptotic biomarkers expression than the OLP group (OR = 2.34, 95%CI = 1.16–4.70, *p* = 0.02), while patients with OSCC did not show significant differences with OLP samples for pro-apoptotic biomarkers (OR = 0.90, 95%CI = 0.27–3.03, *p* = 0.87; Table 2, supplementary information). 

Magnitude of association between OLP and healthy controls. Samples of OLP showed a significantly higher frequency of anti-apoptotic biomarkers expression than the healthy control group (OR = 3.95, 95%CI = 1.07–14.63, *p* = 0.04). Patients with OLP showed a significantly higher frequency of pro-apoptotic biomarkers expression than the healthy control group (OR = 5.25, 95%CI = 2.07–13.31, *p* < 0.001; Table 2, supplementary information).

Magnitude of association between OSCC and healthy controls. Patients with OSCC showed a significantly higher frequency of anti-apoptotic biomarkers expression than the healthy control group (OR = 8.16, 95%CI = 2.19–30.35, *p* = 0.002). On the other hand, OSCC cases showed a significantly higher frequency of pro-apoptotic biomarkers expression than the healthy controls (OR = 48.53, 95%CI = 10.52–223.82, *p* < 0.001; Table 2, supplementary information). 

#### 3.4.4. Hallmark 4: Enabling Replicative Immortality

Differential expression in OLP. PP was 41.67% (95%CI = 32.31–51.66) for the immortalization biomarkers. No heterogeneity was present as just one primary-level study was included in this analysis (Table 2, supplementary information). 

Magnitude of association between OSCC and OLP. Patients with OSCC showed a significantly higher frequency of the immortalization biomarkers expression than the OLP patients (OR = 18.14, 95%CI = 0.99–331.13, *p* = 0.051; Table 2, supplementary information). 

Magnitude of association between OLP and healthy controls. Patients with OLP showed a significantly higher frequency of the immortalization biomarkers expression than the healthy control group (OR = 15.05, 95%CI = 0.86–264.32, *p* = 0.06; Table 2, supplementary information).

Magnitude of association between OSCC and healthy controls. Patients with OSCC showed a significantly higher frequency of the immortalization biomarkers expression than the healthy control group (OR = 273.00, 95%CI = 4.80–15,515, *p* = 0.007; Table 2, supplementary information).

#### 3.4.5. Hallmark 5: Inducing Angiogenesis

Differential expression in OLP. PP was 94.76% (95%CI = 65.81–100), with a high degree of heterogeneity (*I*^2^ = 91.0%, *p* < 0.001) for pro-angiogenic biomarkers (Table 2, supplementary information).

Magnitude of association between OSCC and OLP. There were no studies found which compared frequency of pro-angiogenic biomarkers expression between patients with OSCC and patients with OLP. 

Magnitude of association between OLP and healthy controls. Patients with OLP did not show significant differences in the frequency of pro-angiogenic biomarkers expression than the healthy control group (OR = 2.40, 95%CI = 0.62–9.27, *p* = 0.20; Table 2, supplementary information).

Magnitude of association between OSCC and healthy controls. There were no studies entered in the meta-analysis to compare the frequency of pro-angiogenic biomarkers expression between patients with OSCC and healthy controls.

#### 3.4.6. Hallmark 6: Activating Invasion and Metastasis

Differential expression in OLP. The PP for pro-invasive biomarkers was 69.76% (95%CI = 55.72–82.29), with a high degree of heterogeneity (*I*^2^ = 94.2%, *p* < 0.001). PP for anti-invasive biomarkers was 86.59% (95%CI = 29.86–100), with a high degree of heterogeneity (*I*^2^ = 95.3%, *p* < 0.001) (Table 2, supplementary information). 

Magnitude of association between OSCC and OLP. Patients with OSCC showed a significantly higher frequency of pro-invasive biomarkers than the OLP group (OR = 6.95, 95%CI = 3.20–15.10, *p* < 0.001). Patients with OSCC did not show significant differences in the expression of anti-invasive biomarkers than the OLP group (OR = 1.38, 95%CI = 0.37–5.15, *p* = 0.64; Table 2, supplementary information).

Magnitude of association between OLP and healthy controls. Patients with OLP showed a significantly higher frequency of pro-invasive biomarkers than the healthy control group (OR = 13.50, 95%CI = 5.12–35.59, *p* < 0.001). Patients with OLP showed a significantly higher frequency of anti-invasive biomarkers than the healthy control group (OR = 15.59, 95%CI = 2.58–93.99, *p* = 0.003; Table 2, supplementary information).

Magnitude of association between OSCC and healthy controls. Patients with OSCC showed a significantly higher frequency of pro-invasive biomarkers than the healthy control group (OR = 28.04, 95%CI = 8.71–90.28, *p* < 0.001). Patients with OSCC showed a significantly higher frequency of anti-invasive biomarkers than the healthy control group (OR = 20.00, 95%CI = 1.97–203.32), *p* = 0.01; Table 2, supplementary information).

#### 3.4.7. Hallmark 7: Avoiding Immune Destruction

Differential expression in OLP. The PP for biomarkers avoiding immune destruction was 77.96% (95%CI = 51.96–95.96), with a high degree of heterogeneity (*I*^2^ = 92.8%, *p* < 0.001; Table 2, supplementary information). 

Magnitude of association between OSCC and OLP. There were no studies entered in the meta-analysis to compare the frequency of anti-tumor arrest biomarkers between patients with OSCC and OLP.

Magnitude of association between OLP and healthy controls. Patients with OLP showed a significantly higher frequency of biomarkers avoiding immune destruction than the healthy control group (OR = 107.92, 95%CI = 13.63–843.45, *p* < 0.001; Table 2, supplementary information).

Magnitude of association between OSCC and healthy controls. No studies were identified that compared the frequency of anti-tumor arrest biomarkers between patients with OSCC and healthy controls.

#### 3.4.8. Hallmark 8: Deregulating Cellular Energetics

Differential expression in OLP. The pooled proportion (PP) for deregulating cellular energetics biomarkers was 69.57% (95%CI = 49.13–84.40). Heterogeneity was not studied as just one record was included in this analysis.

Magnitude of association between OSCC and OLP. There were no studies entered in the meta-analysis to compare the frequency of deregulating cellular energetics biomarkers expression between patients with OSCC and OLP.

Magnitude of association between OLP and healthy controls. Patients with OLP showed a significantly higher frequency of deregulating cellular energetics biomarkers than the healthy patients (OR = 33.00, 95%CI = 1.66–656.23, *p* = 0.02; Table 2, supplementary information).

Magnitude of association between OSCC and healthy controls. No studies were identified that compared the frequency of deregulating cellular energetics biomarkers between patients with OSCC and healthy controls.

#### 3.4.9. Hallmark 9: Genome Instability and Mutation

Differential expression in OLP. The estimated PP for biomarkers involved in DNA instability was 48.44% (95%CI = 13.54–84.19), with a high degree of heterogeneity (*I*^2^ = 95.3%, *p* < 0.001). The PP for biomarkers involved in DNA damage repair was 72.37% (95%CI = 32.96–98.42), with a high degree of heterogeneity (*I*^2^ = 91.6%, *p* = 0.001; Table 2, supplementary information).

Magnitude of association between OSCC and OLP. There were no studies entered in the meta-analysis to compare the frequency of biomarkers involved in DNA instability biomarkers between patients with OSCC and OLP. Patients with OSCC showed a significantly higher frequency of biomarkers expression involved in DNA damage repair than the OLP patients (OR = 2.88, 95%CI = 0.24–60.81, *p* = 0.50; Table 2, supplementary information).

Magnitude of association between OLP and healthy controls. No studies were identified that compared the frequency of biomarkers involved in DNA instability between patients with OLP and healthy controls. Patients with OSCC showed a significantly lower frequency of biomarkers involved in DNA damage repair than the OLP patients (OR = 0.28, 95%CI = 0.11–0.73, *p* = 0.009; Table 2, supplementary information).

Magnitude of association between OSCC and healthy controls. Patients with OSCC showed a significantly higher frequency of biomarkers involved in DNA instability than the healthy controls (OR = 1653.00, 95%CI = 30.82–88,665, *p* < 0.001; Table 2, supplementary information). There were no studies entered in the meta-analysis to compare the frequency of biomarkers involved in DNA damage repair biomarkers between patients with OLP and healthy controls.

#### 3.4.10. Hallmark 10: Tumor-Promoting Inflammation

Differential expression in OLP. The estimated PP for pro-inflammatory biomarkers was 83.10% (95%CI = 73.93–90.74), with a high degree of heterogeneity (*I*^2^ = 93.7%, *p* < 0.001) (Table 2, supplementary information). 

Magnitude of association between OSCC and OLP. Patients with OSCC showed a significantly higher frequency for pro-inflammatory biomarkers than the OLP patients (OR = 2.40, 95%CI = 0.88–6.51, *p* = 0.09; Table 2, supplementary information).

Magnitude of association between OLP and healthy controls. Patients with OLP showed a significantly higher frequency of biomarkers involved in DNA damage repair than the healthy controls (OR = 7.50, 95%CI =1.97–28.56, *p* = 0.003; Table 2, supplementary information).

Magnitude of association between OSCC and healthy controls. Patients with OSCC showed a significantly higher frequency of biomarkers involved in DNA damage repair than the healthy controls (OR = 15.24, 95%CI = 2.54–91.34, *p* = 0.003; Table 2, supplementary information).

#### 3.4.11. Unspecified

Finally, some biomarkers were classified as “unspecified” and were not categorized within the preceding hallmarks (Table S3 in supplementary information). All biomarkers included in this unspecified group were excluded from the meta-analysis because they were considered to be the highest source of risk of bias in the present study. This was the case for all biomarkers with a well-known pleiotropism, with poorly characterized biological roles, or with insufficient information provided by primary-level studies (e.g., lack of reporting on the topographic cell localization of β-catenin, a biomarker whose functions are very different according to its overexpression in the cell membrane—as a protector, promoting cell–cell junctions and regulating epithelial tissue homeostasis—or in the cell nucleus—as an oncogene, regulating the activation of key transcription factors of cell proliferation or epithelial–mesenchymal transition phenomenon pathways).

### 3.5. Analysis of Small-Study Effects

Visual inspection analysis of the asymmetry of the funnel plots (supplementary information) and the statistical tests performed for the same purpose confirm the absence of small-study effects on the meta-analyses of the differential expression on the hallmarks of cancer in OLP (hallmark 1: sustaining proliferative signaling [*p_Egger_* = 0.36]; hallmark 2: evading growth suppressors [*p_Egger_* = 0.48]; hallmark 3: anti-apoptotic role [*p_Egger_* = 0.26] and pro-apoptotic role [*p_Egger_* = 0.19]), with the exception of the hallmark 6: activating invasion and metastasis (*p_Egger_* = 0.004) and hallmark 10: tumor-promoting inflammation (*p_Egger_* = 0.07), for which biases—e.g., publication bias—could not be ruled out. The rest of the variables did not enter in the statistical analysis of small-study effects, since a low number of studies (n < 10) were included in their corresponding meta-analyses of proportions.

## 4. Discussion

Our results regarding the capacity of the oral epithelium affected by lichen planus to maintain *sustained proliferative signaling* derive from the meta-analysis of 35 primary-level studies on 1011 oral mucosal samples focusing on the immunohistochemical overexpression of a total of 18 proliferative biomarkers. These markers represent the upregulation of proteins that at different levels, from membrane receptors to activated genes, are part of pathways whose function is to stimulate cell proliferation. The main conclusion of our meta-analysis in this aspect indicates that OLP essentially develops a hyperproliferative epithelial response since 65.48% (95%CI = 51.87–78.02) of the analyzed cases express proliferation markers. Evidence derived from our meta-analysis points to EGFR alterations as a molecular mechanism frequently implicated in the development of the hyperproliferative state in OLP with 85.27% of cases overexpressing this protein, which presumably overregulates its actions constitutively without the requirement of its ligand binding, although other oncogenic mechanisms linked to EGFR upregulation are plausible [147]. Probably, the upregulation of the *CCND1* gene with overexpression of its cyclin D1 protein is also relevant as an oncogenic mechanism, since it is a tributary gene of several pro-proliferative pathways [148,149,150,151,152], although research in this aspect is limited and only relies on two primary-level studies that report *CCND1*/cyclin D1 activation in 69.23% of OLP cases. Furthermore, we observed that the probabilities of OLP-affected epithelium developing a hyperproliferative response is significantly higher compared to normal oral mucosa used as a control (OR = 2.90; 95%CI = 1.27–6.65, *p* = 0.01). Our comparison with proliferative activity in OSCC demonstrates, as expected, that tumor tissue expresses proliferation markers 4.39 times more frequently compared to OLP (95%CI = 2.22–8.71, *p* < 0.001), which seems to place OLP at an intermediate stage between normal and cancer in terms of its proliferative activity. This ability to maintain a sustained proliferative signaling constitutes one of the essential mechanisms promoting oral oncogenesis since it facilitates, by creating a state of genomic instability, the acquisition of mutations that endow the different cell clones with oncogenic advantages. 

Our results demonstrate in relation to the *acquisition of cell death resistance*, based on 32 studies and 537 samples, that both proapoptotic (PP = 64.92%; 95%CI = 55.15–74.14) and antiapoptotic (PP = 55.93%; 95%CI = 35.99–75.05) mechanisms occur in OLP. Proapoptotic molecular phenomena are to be expected since apoptosis is part of the histopathological spectrum of OLP—Civatte bodies—and reflects cellular damage produced by autoimmune aggression; however, the activation of proteins with anti-apoptotic functions should be interpreted, in our opinion, as a mechanism to prevent cell death caused by autoimmune aggression in an attempt to maintain epithelial regenerative capacity and avoid the appearance of the most severe clinical forms of the disease—erosive OLP. Both proapoptotic (OR = 5.25; 95%CI = 2.07–13.31, *p* < 0.001) and antiapoptotic (OR = 3.95, 95%CI = 1.07–14.63, *p* = 0.04) phenomena appear with significantly higher frequency in OLP vs. healthy oral mucosa. Our results further indicate that the probability of finding overexpression of anti-apoptotic markers is higher in OSCC compared to OLP (OR = 2.34, 95%CI = 1.16–4.70, *p* = 0.02), which seems reasonable because in cancer the establishment of cell survival mechanisms is determinant; nevertheless, our study also reflects that resistance to cell death could be acquired early in the course of OLP malignization. The main evidence for the development of anti-apoptotic molecular mechanisms in OLP derives from the study of bcl-2 overexpression observed in 46.92% of cases (14 primary-level studies, 373 patients). The fact that some hyperproliferative cell clones acquire resistance to cell death could be key in the development of cancer on OLP [62].

The concept of *genomic instability*—categorized by Hanahan and Weinberg as an enabling characteristic—refers to the tendency for mutations and other chromosomal aberrations to develop as a consequence of the lack of control of cell proliferation. It stands to reason that the hyperproliferative and antiapoptotic state observed in some OLPs results in genomic instability and may become a clonally transmitted force driving oncogenesis in this OPMD [153]. Although research in this regard is scarce, the results of five primary-level studies on different markers of genomic instability in OLPs (Table S3 in supplementary information) indicate that in 48.44% of cases (95%CI = 13.54–84.19), this oncogenic facilitating mechanism is developing.

The pro-oncogenic molecular field developed in OLP—hyperproliferation, resistance to cell death, genomic instability—draws an epithelium theoretically strongly predisposed to cancer development. However, this is one of the OPMD with less predisposition to malignization compared to others such as leukoplakia, erythroplakia, or proliferative verrucous leukoplakia [4]. This low malignancy ratio could be due to the establishment of protective molecular mechanisms against malignant transformation essentially linked to the actions of tumor suppressor genes. Our meta-analysis on 36 primary-level studies and 1096 patients points out that tumor suppressor gene activation is observed in 63.15% of OLP cases (95%CI = 52.26–73.45) and this activation is much more frequent in OLP compared to normal oral mucosa used as control (OR = 11.43, 95%CI = 6.89–18.95, *p* < 0.001). The strongest evidence comes from studies focusing on p53 (24 primary-level studies, 689 patients) reporting overexpression of the protein in 56.74% of cases. It is known to be difficult to differentiate by immunohistochemistry the mutated p53 protein with lost or aberrant functions from the wild-type form with conserved physiologic functions, although some studies, including those of our group [21,22], consider that the overexpression of p53 in OLP is primarily due to the wild-type form that is essentially arresting the cell cycle to repair DNA damaged by autoimmune cytotoxic insults [105,154]. There is also very little evidence for the existence of TP53 mutations in OLP, although they appear to occur as they have been detected in varying percentages of cases in small series that are not very representative [49,93].

The ability of the inflammatory microenvironment to promote tumor development is based on the evidence that chronic inflammatory diseases increase the risk for some types of cancers (including bladder, cervical, gastric, intestinal, esophageal, ovarian, prostate, and thyroid cancers); this oncogenic promotion is linked to pathways that are activated in inflammatory processes by mutations in oncogenes (such as mutations in the genes encoding *RAS* and *MYC*); furthermore, inflammatory cells, chemokines, and cytokines are found in all tumors from the early stages of their development [155]. All these evidences and some others have conditioned that *tumor-promoting inflammation* has been considered as an enabling characteristic [26]. We have observed that 83.10% (95%CI = 73.93–90.74) of OLP cases express inflammatory markers with tumor-promoting functions. The probability that this group of markers is overexpressed in OLP is significantly higher compared to normal oral mucosa (OR = 7.50, 95%CI = 1.97–28.56, *p* = 0.003), which provides evidence for the involvement of autoimmune-linked inflammation in the malignant transformation of OLP. Although there are multiple molecules in the inflammatory microenvironment with tumor-promoting functions, some of them have been considered as key factors. The transcription factor NF-κB appears crucial by operating downstream through signaling pathways linked to the TLR-MyD88 receptor, and through pathways mediated by the inflammatory cytokines TNF-α and IL-1β [155]. We know that in epithelial cells at risk of malignization, NF-κB activates the expression of genes encoding inflammatory cytokines, adhesion molecules, COX-2, iNOS, and proangiogenic factors among others [155]. NF-κB can also promote cell survival via induction of the anti-apoptotic gene BCL-2; recall that this is one of the markers of resistance to cell death found in this meta-analysis. Despite the importance of NF-κB in tumor promotion, no primary-level studies have been published to date on its relevance in OLP, although some of the cytokines and enzymes tributary to its actions (TNF-α and COX-2) have been studied. The importance of TNF-α upregulation in OLP has been evaluated in three primary-level studies reporting an overexpression in 96.30% of OLP cases analyzed, which is relevant because of the known implications of TNF-α in the mesenchymal epithelial transition phenomenon by which epithelial cells acquire motility and invasive capacity [156]. COX-2 has been evaluated in six primary-level studies analyzing 253 patients, in which an overexpression of this proinflammatory enzyme is reported in 92.12% of OLP cases. This information is significant because COX-2 is rarely expressed in normal mucosa and, in affected tissue, it behaves as a tumor promoter through its apoptosis inhibitory, hyperproliferative, and neoangiogenic effect [78,157]. It is obvious that knowledge on the implications of the inflammatory microenvironment in tumor promotion in OLP is scarce and should be increased in future research, essentially because an adequate control of the autoimmune inflammatory phenomenon through the use of immunosuppressants should hypothetically decrease its risk of malignant transformation, although there is no evidence on this.

Tumor or premalignant cells should be able to *avoid destruction mediated by the antitumor immune response*, which has been proposed by Hanahan and Weinberg as an emerging hallmark. A very relevant molecular mechanism of acquisition of this hallmark is linked to the overexpression of PD-L proteins in tumor cells, which would activate T lymphocyte apoptosis after binding to their receptors (PD-1 and PD-2) expressed on the lymphocyte membrane [158,159,160]. All primary-level studies concerning the acquisition of this hallmark in OLP (four studies, 186 patients) refer to overexpression of PD-L proteins. The results of our meta-analysis reflect that a high percentage of OLP cases overexpress PD-L proteins (77.96%, 95%CI = 51.96–95.96), with the probability of overexpression of these proteins being much higher in OLP vs. normal oral mucosa (OR = 107.92, 95%CI = 13.63–843.45, *p* < 0.001). Thus, PD-L overregulation in OLP could reflect both a protective mechanism of oral epithelial cells against immune aggression and an early mechanism of escape from immune-mediated antitumor surveillance in case of malignant transformation.

Our results demonstrate that the expression of proteins reflecting the *activation of invasion and metastasis* is frequent in OLP. On the basis of 21 studies and 914 tissue samples, we report that 69.76% of cases (95%CI = 55.72–82.29) overexpress these markers which points to the fact that the molecular mechanism of metastatic activation may begin to be established very early [161]. Furthermore, the probability that the affected tissue expresses invasion and metastasis-activating proteins is 13.50 times higher in OLP compared to normal mucosa used as control (95%CI = 5.12–35.59; *p* < 0.001); on the contrary and as expected, these molecular mechanisms are more common in OSCC vs. OLP (OR = 6.95, 95%CI = 3.20–15.10, *p* < 0.001) as a consequence of metastatic capacity being at the very essence of tumor development. The acquisition of metastatic capacity is promoted in the oral epithelium by the phenomenon called epithelial mesenchymal transition whose most distinctive molecular mark is the loss of expression of the adhesion molecule E-cadherin. The primary-level studies in OLP on this aspect (five studies, 153 cases) only report E-cadherin overexpression, which appears between 51.92% and 100% of cases depending on the studies and not its loss of expression; although for reasons of methodological heterogeneity, E-cadherin expression could not be included in the meta-analysis of this hallmark, an indirect estimation of the results of the primary-level studies allows us to deduce that in a percentage of OLP cases (17.6%), E-cadherin is under-expressed as an early signal of the establishment of epithelial–mesenchymal transition phenomenon.

## 5. Conclusions

In conclusion, our meta-analysis indicates established molecular mechanisms in OLP that promote a hyperproliferative and antiapoptotic state with genomic instability, which would ultimately drive the acquisition of clonally transmitted summative oncogenic molecular events in some cell lineages. These events could plausibly derive from the actions exerted by the inflammatory infiltrate itself; moreover, epithelial cells on the way to malignization could acquire the ability to avoid destruction mediated by the antitumor immune response essentially via overexpression of PD-L proteins. This malignant transformation-promoting environment should be prevented by the upregulated actions of tumor suppressor genes, essentially p53, which seem to function very well in OLP in view of the fact that this is one of the OPMDs with the lowest malignization rate compared to the others. In contrast, it seems reasonable to accept that the failure of the functions of these tumor suppressor genes, by mutation or other alterations, must be determinant in the acquisition of a definitive malignant phenotype.

Our study, however, has some limitations among which is the absence of primary-level studies that focus on the analysis of cancer markers in OLP cases that have undergone malignant transformation and compare with non-malignant cases; furthermore, although very interesting, it was not possible to explore through stratified meta-analyses the influence of relevant potential risk factors, such as smoking, gender, and age of patients, clinical appearance of lesions, among others, and consequently, there are studies based on heterogeneous cases of OLP [162,163]. Unfortunately, these analyses could not be performed due to the failure to report individual participant data across the included primary-level studies. Therefore, this is certainly an inherent limitation of the studies included in the present systematic review and meta-analysis. Based on the above limitations, we recommend that future studies report detailed protein overexpression profiles stratified by the precedent parameters, preferably in the form of individual participant data to enable a comprehensive and exhaustive control of potentially confounding variables and sources of heterogeneity. Moreover, future observational studies that combine clinical with basic research conducted with this purpose (i.e., analyze the differential expression of the hallmarks of cancer in oral lichen planus) should be carefully designed, preferably as prospective cohorts. These studies should also provide clear information on the OLP diagnostic criteria used (clinical and histopathological criteria are desirable), preliminary sample size determination, follow-up periods should be large, the demographic and clinicopathological characteristics of OLP and carcinomas should be clearly reported, and finally, the experimental immunohistochemistry conditions should be carefully reported (i.e., type of antibody, cut-point for positive cases, intracellular pattern, antibody incubation time, concentration, and dilution). In addition, the lack of reporting of individualized patients’ data also prevents us from knowing in what proportion of lesions all these cancer hallmarks are globally expressed to select which of them are more relevant in malignization and to reach a pattern of hallmark expression that allows us to reliably establish risk predictions in specific cases; on the other hand, more specific meta-analyses related to individual proteins rather than groups of proteins would be particularly useful, singularly for the most frequently analyzed proteins across studies, such as bcl-2, caspases, bax-2, p16, p21, p53, and PD-L. Deeper meta-analyses by biomarkers individually and adjusted for all relevant variables would be undoubtedly enriching, in order to provide a clearer understanding of the role of each protein in OLP. Finally, it is difficult to attribute a concrete function to many markers (Table S3 in supplementary information) due to the multifunctional pleiotropism that is frequently present; and finally, as has become evident in our discussion, there is no research on many of the cancer hallmarks in OLP, which should stimulate the implementation of future lines of research on the subject. 

## Figures and Tables

**Figure 2:**
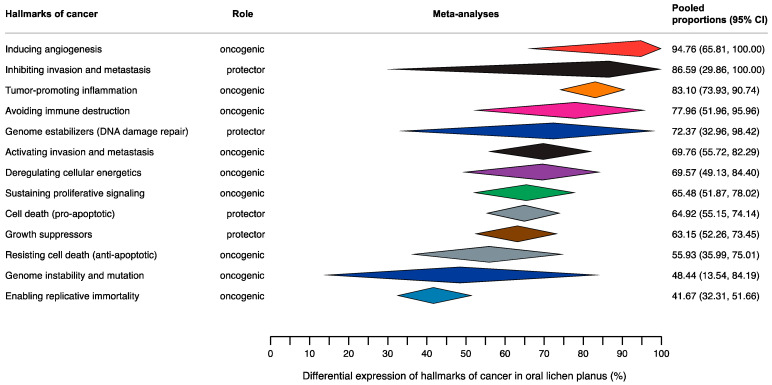
Summary Forest Plot (aka forest top plot) graphically representing pooled proportions—expressed as percentages—with their corresponding 95% confidence intervals, obtained through the meta-analyses on the hallmarks of cancer in samples of mucosa from OLP patients. This plot exhibits the results of all meta-analyses carried out—row by row, the meta-analyses findings were depicted as diamonds—according to the different hallmarks of cancer expressed in OLP (*n* = 13 different meta-analyses of proportions performed in this study).

**Table 1:** Summarized characteristics of the study sample.

Total	110 studies
Year of publication	1994–2023
Total cases (range)	7065 * (3–123)
Study design	
Retrospective cohort	108
Prospective cohort	2
Experimental methods	
Immunohistochemistry	110 studies
Geographical region	
Europe	37 studies (13 countries)
Asia	54 studies (8 countries)
North America	3 studies (3 countries)
South America	13 studies (3 countries)
Africa	2 studies (1 country)
Oceania	1 study (1 country)
Total	5 continents, 29 countries

* Note: more than one biomarker was analyzed per study. Table S2 (supplementary information) summarizes the characteristics of each study.

**Table 2:** Meta-analysis on the hallmarks of oral cancer in oral lichen planus samples.

					Pooled Data		Heterogeneity	
Meta-Analyses	No. of Studies *	No. of Cases *	Stat. Model	Wt	ES (95%CI)	*p-Value*	*P_het_*	*I^2^* *(%)*
**Hallmark 1: Sustaining proliferative signaling**								
Differential expression in OLP								
Subgroup analysis by role								
Oncogenic (pro-proliferative)	35	1011	REM	D-L	PP = 65.48% (51.87–78.02)	—	<0.001	94.5
Magnitude of association (OSCC vs. OLP)								
Subgroup analysis by role								
Oncogenic (pro-proliferative)	18	934	REM	D-L	OR = 4.39 (2.22–8.71)	<0.001	0.001	58.5
Magnitude of association (OLP vs. healthy controls)								
Subgroup analysis by role								
Oncogenic (pro-proliferative)	20	932	REM	D-L	OR = 2.90 (1.27–6.65)	0.01	<0.001	71.9
Magnitude of association (OSCC vs. healthy controls)								
Subgroup analysis by role								
Oncogenic (pro-proliferative)	9	378	REM	D-L	OR = 7.50 (2.58–21.73)	<0.001	0.05	48.4
**Hallmark 2: Evading growth suppressors**								
Differential expression in OLP								
Subgroup analysis by role								
Protector (growth suppressor)	36	1096	REM	D-L	PP = 63.15% (52.26–73.45)	—	<0.001	91.9
Magnitude of association (OSCC vs. OLP)								
Subgroup analysis by role								
Protector (growth suppressor)	18	1278	REM	D-L	OR = 2.16 (1.26–3.69)	0.005	0.009	50.8
Magnitude of association (OLP vs. healthy controls)								
Subgroup analysis by role								
Protector (growth suppressor)	25	1034	REM	D-L	OR = 11.43 (6.89–18.95)	<0.001	0.30	11.4
Magnitude of association (OSCC vs. healthy controls)								
Subgroup analysis by role								
Protector (growth suppressor)	11	577	REM	D-L	OR = 19.18 (8.25–44.61)	<0.001	0.74	0.0
**Hallmark 3: Resisting cell death**								
Differential expression in OLP								
Subgroup analysis by role						0.41 **		
Oncogenic (anti-apoptotic)	22	537	REM	D-L	PP = 55.93% (35.99–75.01)		<0.001	95.0
Protector (pro-apoptotic)	18	636	REM	D-L	PP = 64.92% (55.15–74.14)		<0.001	83.8
Magnitude of association (OSCC vs. OLP)								
Subgroup analysis by role						0.18 **		
Oncogenic (anti-apoptotic)	12	657	REM	D-L	OR = 2.34 (1.16–4.70)	0.02	0.09	39.1
Protector (pro-apoptotic)	5	281	REM	D-L	OR = 0.90 (0.27–3.03)	0.87	0.05	57.0
Magnitude of association (OLP vs. healthy controls)								
Subgroup analysis by role						0.73 **		
Oncogenic (anti-apoptotic)	14	444	REM	D-L	OR = 3.95 (1.07–14.63)	0.04	<0.001	72.2
Protector (pro-apoptotic)	12	600	REM	D-L	OR = 5.25 (2.07–13.31)	<0.001	0.001	66.8
Magnitude of association (OSCC vs. healthy controls)								
Subgroup analysis by role						0.08 **		
Oncogenic (anti-apoptotic)	8	331	REM	D-L	OR = 8.16 (2.19–30.35)	0.002	0.04	52.6
Protector (pro-apoptotic)	3	138	REM	D-L	OR = 48.53 (10.52–223.82)	<0.001	0.63	0.0
**Hallmark 4: Enabling replicative immortality**								
Differential expression in OLP								
Subgroup analysis by role								
Oncogenic (pro-survival/immortalization)	1	96	—	—	PP = 41.67% (32.31–51.66)		—	—
Magnitude of association (OSCC vs. OLP)								
Subgroup analysis by role								
Oncogenic (pro-survival/immortalization)	1	102	—	—	OR = 18.14 (0.99–331.13)	0.051	—	—
Magnitude of association (OLP vs. healthy controls)								
Subgroup analysis by role								
Oncogenic (pro-survival/immortalization)	1	106	—	—	OR = 15.05 (0.86–264.32)	0.06	—	—
Magnitude of association (OSCC vs. healthy controls)								
Subgroup analysis by role								
Oncogenic	1	16	—	—	OR = 273.00 (4.80–15,515)	0.007	—	—
**Hallmark 5: Inducing angiogenesis**								
Differential expression in OLP								
Subgroup analysis by role								
Oncogenic (pro-angiogenic)	3	96	REM	D-L	PP = 94.76% (65.81–100)		<0.001	91.0
Magnitude of association (OSCC vs. OLP)								
Subgroup analysis by role								
Oncogenic (pro-angiogenic)	0	0	—	—	—	—	—	—
Magnitude of association (OLP vs. healthy controls)								
Subgroup analysis by role								
Oncogenic (pro-angiogenic)	1	46	—	—	OR = 2.40 (0.62–9.27)	0.20	—	—
Magnitude of association (OSCC vs. healthy controls)								
Subgroup analysis by role								
Oncogenic (pro-angiogenic)	0	0	—	—	—	—	—	—
**Hallmark 6: Activating invasion and metastasis**								
Differential expression in OLP								
Subgroup analysis by role						0.52 **		
Oncogenic (pro-invasive)	21	914	REM	D-L	PP = 69.76% (55.72–82.29)		<0.001	94.2
Protector (anti-invasive)	2	57	REM	D-L	PP = 86.59% (29.86–100)		<0.001	95.3
Magnitude of association (OSCC vs. OLP)								
Subgroup analysis by role						0.04 **		
Oncogenic (pro-invasive)	14	911	REM	D-L	OR = 6.95 (3.20–15.10)	<0.001	0.08	37.5
Protector (anti-invasive)	1	42	—	—	OR = 1.38 (0.37–5.15)	0.64	—	—
Magnitude of association (OLP vs. healthy controls)								
Subgroup analysis by role						0.89 **		
Oncogenic (pro-invasive)	17	954	REM	D-L	OR = 13.50 (5.12–35.59)	<0.001	<0.001	66.0
Protector (anti-invasive)	2	78	REM	D-L	OR = 15.59 (2.58–93.99)	0.003	0.91	0.0
Magnitude of association (OSCC vs. healthy controls)								
Subgroup analysis by role						0.80 **		
Oncogenic (pro-invasive)	11	328	REM	D-L	OR = 28.04 (8.71–90.28)	<0.001	0.02	51.6
Protector (anti-invasive)	1	26	—	—	OR = 20.00 (1.97–203.32)	0.01	—	—
**Hallmark 7: Avoiding immune destruction**								
Differential expression in OLP								
Subgroup analysis by role								
Oncogenic (anti-tumor arrest)	4	186	REM	D-L	PP = 77.96% (51.96–95.96)		<0.001	92.8
Magnitude of association (OSCC vs. OLP)								
Subgroup analysis by role								
Oncogenic (anti-tumor arrest)	—	—	—	—	—		—	—
Magnitude of association (OLP vs. healthy controls)								
Subgroup analysis by role								
Oncogenic (anti-tumor arrest)	2	140	REM	D-L	OR = 107.92 (13.63–843.45)	<0.001	0.96	0.0
Magnitude of association (OSCC vs. healthy controls)								
Subgroup analysis by role								
Oncogenic (anti-tumor arrest)	0	0	—	—	—	—	—	—
**Hallmark 8: Deregulating cellular energetics**								
Differential expression in OLP								
Subgroup analysis by role								
Oncogenic (enhancing tumor acidosis)	1	23	—	—	PP = 69.57% (49.13–84.40)		—	—
Magnitude of association (OSCC vs. OLP)								
Subgroup analysis by role								
Oncogenic (enhancing tumor acidosis)	0	0	—	—	—	—	—	—
Magnitude of association (OLP vs. healthy controls)								
Subgroup analysis by role								
Oncogenic (enhancing tumor acidosis)	1	30	—	—	OR = 33.00 (1.66–656.23)	0.02	—	—
Magnitude of association (OSCC vs. healthy controls)								
Subgroup analysis by role								
Oncogenic (enhancing tumor acidosis)	0	0	—	—	—	—	—	—
**Hallmark 9: Genome instability and mutation**								
Differential expression in OLP								
Subgroup analysis by role						0.39 **		
Oncogenic (DNA instability)	5	157	REM	D-L	PP = 48.44% (13.54–84.19)		<0.001	95.3
Protector (DNA damage repair)	2	79	REM	D-L	PP = 72.37% (32.96–98.42)		0.001	91.6
Magnitude of association (OSCC vs. OLP)								
Subgroup analysis by role								
Oncogenic (DNA instability)	—	—	—	—	—	—	—	—
Protector (DNA damage repair)	1	38	—	—	OR = 2.88 (0.14–60.81)	0.50	—	—
Magnitude of association (OLP vs. healthy controls)								
Subgroup analysis by role								
Oncogenic (DNA instability)	—	—	—	—	—	—	—	—
Protector (DNA damage repair)	1	91	—	—	OR = 0.28 (0.11–0.73)	0.009	—	—
Magnitude of association (OSCC vs. healthy controls)								
Subgroup analysis by role								
Oncogenic (DNA instability)	1	52	—	—	OR = 1653.0 (30.82–88,665)	<0.001	—	—
Protector (DNA damage repair)	0	0	—	—	—	—	—	—
**Hallmark 10: Tumor-promoting inflammation**								
Differential expression in OLP								
Subgroup analysis by role								
Oncogenic (pro-inflammatory)	29	1386	REM	D-L	PP = 83.10% (73.93–90.74)		<0.001	93.7
Magnitude of association (OSCC vs. OLP)								
Subgroup analysis by role								
Oncogenic (pro-inflammatory)	8	301	REM	D-L	OR = 2.40 (0.88–6.51)	0.09	0.06	49.2
Magnitude of association (OLP vs. healthy controls)								
Subgroup analysis by role								
Oncogenic (pro-inflammatory)	14	691	REM	D-L	OR = 7.50 (1.97–28.56)	0.003	<0.001	74.5
Magnitude of association (OSCC vs. healthy controls)								
Subgroup analysis by role								
Oncogenic (pro-inflammatory)	6	193	REM	D-L	OR = 15.24 (2.54–91.34)	0.003	0.02	62.8

Abbreviations: Stat., statistical; Wt, method of weighting; PP, pooled proportion; OR, odds ratio; CI, confidence intervals; REM, random-effects model; D-L, DerSimonian and Laird method; OLP, oral lichen planus; OSCC, oral squamous cell carcinoma. *—Note that more than one analysis unit was analyzed per study and patient. **—Test for between-subgroup differences.

## Data Availability

The original contributions presented in the study are included in the article/supplementary material, further inquiries can be directed to the corresponding authors.

## Supplementary Materials

The following supporting information can be downloaded at: https://www.mdpi.com/article/10.3390/cancers16152614/s1, Table S1. Search strategy for each database, number of results, and execution date; Table S2. Characteristics of analyzed studies; Table S3. Biomarkers roles and hallmarks of cancer; Table S4. Quality plot graphically representing the risk of bias the risk of bias in individual studies, critically appraising ten domains, using a method specifically designed for systematic reviews addressing questions of prevalence (developed by the Joanna Briggs Institute, University of Adelaide, South Australia). Green, low risk of potential bias; yellow, moderate; red, high; Figure S1. Forest plot graphically representing the differential expression of biomarkers on the hallmark of cancer sustaining proliferation -using pooled proportions as ES metric, expressed as percentage- among OLP patients. ES, effect size; CI, confidence interval; Random-effects model; Figure S2. Forest plot graphically representing the differential expression of biomarkers on the hallmark of evading growth suppressors -using pooled proportions as ES metric, expressed as percentage- among OLP patients. ES, effect size; CI, confidence interval; Random-effects model; Figure S3. Forest plot graphically representing the differential expression of biomarkers on the hallmark of resisting cell death -using pooled proportions as ES metric, expressed as percentage- among OLP patients. ES, effect size; CI, confidence interval; Random-effects model; Figure S4. Forest plot graphically representing the differential expression of biomarkers on the hallmark of enabling replicative immortality -using pooled proportions as ES metric, expressed as percentage- among OLP patients. ES, effect size; CI, confidence interval; Random-effects model; Figure S5. Forest plot graphically representing the differential expression of biomarkers on the hallmark of inducing angiogenesis -using pooled proportions as ES metric, expressed as percentage- among OLP patients. ES, effect size; CI, confidence interval; Random-effects model; Figure S6. Forest plot graphically representing the differential expression of biomarkers on the hallmark of activating invasion and metastasis -using pooled proportions as ES metric, expressed as percentage- among OLP patients. ES, effect size; CI, confidence interval; Random-effects model; Figure S7. Forest plot graphically representing the differential expression of biomarkers on the hallmark of avoiding immune destruction -using pooled proportions as ES metric, expressed as percentage- among OLP patients. ES, effect size; CI, confidence interval; Random-effects model; Figure S8. Forest plot graphically representing the differential expression of biomarkers on the hallmark of deregulating cellular energetics -using pooled proportions as ES metric, expressed as percentage- among OLP patients. ES, effect size; CI, confidence interval; Random-effects model. ES, effect size; CI, confidence interval; Random-effects model; Figure S9. Forest plot graphically representing the differential expression of biomarkers on the hallmark of genome instability and mutation -using pooled proportions as ES metric, expressed as percentage- among OLP patients. ES, effect size; CI, confidence interval; Random-effects model; Figure S10. Forest plot graphically representing the differential expression of biomarkers on the hallmark of Tumor promoting and inflammation -using pooled proportions as ES metric, expressed as percentage- among OLP patients. ES, effect size; CI, confidence interval; Random-effects model; Figure S11. Forest plot graphically representing the meta-analysis on the magnitude of association of the hallmark of cancer sustaining proliferation -using OR as effect size metric- in order to compare the differential expression of biomarkers of the hallmark sustaining proliferative signaling between oral cancer and OLP. OR, odds ratio; CI, confidence interval; Random-effects model; Figure S12. Forest plot graphically representing the meta-analysis on the magnitude of association -using OR as effect size metric- in order to compare the differential expression of biomarkers of the hallmark evading growth suppressors between oral cancer and OLP. OR, odds ratio; CI, confidence interval; Random-effects model; Figure S13. Forest plot graphically representing the meta-analysis on the magnitude of association -using OR as effect size metric- in order to compare the differential expression of biomarkers of the hallmark resisting cell death between oral cancer and OLP. OR, odds ratio; CI, confidence interval; Random-effects model; Figure S14. Forest plot graphically representing the meta-analysis on the magnitude of association -using OR as effect size metric- in order to compare the differential expression of biomarkers of the hallmark enabling replicative immortality between oral cancer and OLP. OR, odds ratio; CI, confidence interval; Random-effects model; Figure S15. Forest plot graphically representing the meta-analysis on the magnitude of association -using OR as effect size metric- in order to compare the differential expression of biomarkers of the hallmark activating invasion and metastasis between oral cancer and OLP. OR, odds ratio; CI, confidence interval; Random-effects model; Figure S16. Forest plot graphically representing the meta-analysis on the magnitude of association -using OR as effect size metric- in order to compare the differential expression of biomarkers of the hallmark genome instability and mutation between oral cancer and OLP. OR, odds ratio; CI, confidence interval; Random-effects model; Figure S17. Forest plot graphically representing the meta-analysis on the magnitude of association -using OR as effect size metric- in order to compare the differential expression of biomarkers of the hallmark tumor promoting and inflammation between OLP and oral cancer. OR, odds ratio; CI, confidence interval; Random-effects model; Figure S18. Forest plot graphically representing the meta-analysis of the magnitude of association -using OR as effect size metric- in order to compare the differential expression of biomarkers on the hallmark of cancer sustaining proliferation between OLP and healthy controls. OR, odds ratio; CI, confidence interval; Random-effects model; Figure S19. Forest plot graphically representing the meta-analysis of the magnitude of association -using OR as effect size metric- in order to compare the differential expression of biomarkers on the hallmark of evading growth suppressors between OLP and healthy controls. OR, odds ratio; CI, confidence interval; Random-effects model; Figure S20. Forest plot graphically representing the meta-analysis of the magnitude of association -using OR as effect size metric- in order to compare the differential expression of biomarkers on the hallmark of resisting cell death between OLP and healthy controls. OR, odds ratio; CI, confidence interval; Random-effects model; Figure S21. Forest plot graphically representing the meta-analysis of the magnitude of association -using OR as effect size metric- in order to compare the differential expression of biomarkers on the hallmark of enabling replicative immortality between OLP and healthy controls. OR, odds ratio; CI, confidence interval; Random-effects model; Figure S22. Forest plot graphically representing the meta-analysis of the magnitude of association -using OR as effect size metric- in order to compare the differential expression of biomarkers on the hallmark of inducing angiogenesis between OLP and healthy controls. OR, odds ratio; CI, confidence interval; Random-effects model; Figure S23. Forest plot graphically representing the meta-analysis of the magnitude of association -using OR as effect size metric- in order to compare the differential expression of biomarkers on the hallmark of activating invasion and metastasis between OLP and healthy controls. OR, odds ratio; CI, confidence interval; Random-effects model; Figure S24. Forest plot graphically representing the meta-analysis of the magnitude of association -using OR as effect size metric- in order to compare the differential expression of biomarkers on the hallmark of avoiding immune destruction between OLP and healthy controls. OR, odds ratio; CI, confidence interval; Random-effects model; Figure S25. Forest plot graphically representing the meta-analysis of the magnitude of association -using OR as effect size metric- in order to compare the differential expression of biomarkers on the hallmark of deregulating cellular energetics between OLP and healthy controls. OR, odds ratio; CI, confidence interval; Random-effects model; Figure S26. Forest plot graphically representing the meta-analysis of the magnitude of association -using OR as effect size metric- in order to compare the differential expression of biomarkers on the hallmark of genome instability and mutation between OLP and healthy controls. OR, odds ratio; CI, confidence interval; Random-effects model; Figure S27. Forest plot graphically representing the meta-analysis of the magnitude of association -using OR as effect size metric- in order to compare the differential expression of biomarkers on the hallmark of tumor promoting and inflammation between OLP and healthy controls. OR, odds ratio; CI, confidence interval; Random-effects model; Figure S28. Forest plot graphically representing the meta-analysis of the magnitude of association -using OR as effect size metric- in order to compare the differential expression of biomarkers on the hallmark of cancer sustaining proliferation between oral cancer and healthy controls. OR, odds ratio; CI, confidence interval; Random-effects model; Figure S29. Forest plot graphically representing the meta-analysis of the magnitude of association -using OR as effect size metric- in order to compare the differential expression of biomarkers on the hallmark of evading growth suppressors between oral cancer and healthy controls. OR, odds ratio; CI, confidence interval; Random-effects model; Figure S30. Forest plot graphically representing the meta-analysis of the magnitude of association -using OR as effect size metric- in order to compare the differential expression of biomarkers on the hallmark of resisting cell death between oral cancer and healthy controls. OR, odds ratio; CI, confidence interval; Random-effects model; Figure S31. Forest plot graphically representing the meta-analysis of the magnitude of association -using OR as effect size metric- in order to compare the differential expression of biomarkers on the hallmark of enabling replicative immortality between oral cancer and healthy controls. OR, odds ratio; CI, confidence interval; Random-effects model; Figure S32. Forest plot graphically representing the meta-analysis of the magnitude of association -using OR as effect size metric- in order to compare the differential expression of biomarkers on the hallmark of activating invasion and metastasis between oral cancer and healthy controls. OR, odds ratio; CI, confidence interval; Random-effects model; Figure S33. Forest plot graphically representing the meta-analysis of the magnitude of association -using OR as effect size metric- in order to compare the differential expression of biomarkers on the hallmark of genome instability and mutation between oral cancer and healthy controls. OR, odds ratio; CI, confidence interval; Random-effects model; Figure S34. Forest plot graphically representing the meta-analysis of the magnitude of association -using OR as effect size metric- in order to compare the differential expression of biomarkers on the tumor promoting inflammation between oral cancer and healthy controls. OR, odds ratio; CI, confidence interval; Random-effects model; Figure S35. A funnel plot of estimated transformed proportions against their standard errors, graphically representing the analysis of “small-study” effects on the differential expression of the hallmark sustaining proliferative signaling among OLP patients; Figure S36. A funnel plot of estimated transformed proportions against their standard errors, graphically representing the analysis of “small-study” effects on the differential expression of the hallmark evading growth suppressors among OLP patients; Figure S37. A funnel plot of estimated transformed proportions against their standard errors, graphically representing the analysis of “small-study” effects on the differential expression of the antiapoptotic biomarkers among OLP patients; Figure S38. A funnel plot of estimated transformed proportions against their standard errors, graphically representing the analysis of “small-study” effects on the differential expression of the anti-apoptotic biomarkers among OLP patients; Figure S39. A funnel plot of estimated transformed proportions against their standard errors, graphically representing the analysis of “small-study” effects on the differential expression of the hallmark activating invasion and metastasis among OLP patients; Figure S40. A funnel plot of estimated transformed proportions against their standard errors, graphically representing the analysis of “small-study” effects on the differential expression of the hallmark tumor promoting inflammation among OLP patients; List S1. List of included studies (n = 110); List S2. Lack of essential data (n = 72); List S3. Non-Immunohistochemistry technique (n = 245); List S4. Overlapping population (n = 9); List S5. No distinction between oral, cutaneous and genital lichen planus (n = 1).
