# Supplementary material for: A Molecular Hypothesis on Malignant Transformation of Oral Lichen Planus: A Systematic Review and Meta-Analysis of Cancer Hallmarks Expression in This Oral Potentially Malignant Disorder
Source: Cancers (Basel). 2024 Jul 23;16(15):2614. doi: 10.3390/cancers16152614 (PMC11311016; doi:10.3390/cancers16152614)
Supplement: Supplementary file 1 [file cancers-16-02614-s001.zip › cancers-3086352-supplementary.pdf]

Supplementary information to the manuscript

A molecular hypothesis on malignant transformation of oral lichen planus: a systematic review and meta-analysis of cancer hallmarks expression in this oral potentially malignant disorder

Keim-del Pino C[a], Ramos-García P[a*], González-Moles MA[a*],

a- School of Dentistry, University of Granada. Biohealth Research Institute, Ibs.Granada, Spain

Corresponding Authors: *Miguel Ángel González-Moles, Pablo Ramos-Garcia

Oral Medicine Department, School of Dentistry, University of Granada,Granada, Paseo de Cartuja s/n, 18071 Granada, Spain.

Tel.:+34958243804; fax:+34958240908; E-mail: magonzal@ugr.es; pabloramos@ugr.es

Table of contents

1. Search strategy....4

2. Characteristics of analyzed studies....5

3. Differential expression of the hallmarks of cancer in patients with OLP 15

3.1 Hallmark Sustaining proliferative signaling 15

3.2 Hallmark Evading growth suppressors 16

3.3 Hallmark Resisting cell death 17

3.4 Hallmark Enabling replicative immortality 18

3.5 Hallmark Inducing angiogenesis....19

3.6 Hallmark Activating invasion and metastasis....20

3.7 Hallmark Avoiding immune destruction....21

3.8 Hallmark Deregulating cellular energetics....22

3.9 Hallmark Genome instability and mutation 23

3.10 Hallmark Tumor promoting and inflammation....24

4. Magnitude of association between oral cancer and OLP. 25

4.1 Hallmark Sustaining proliferative signaling 25

4.2 Hallmark Evading growth suppressors 26

4.3 Hallmark Resisting cell death 27

4.4 Hallmark Enabling replicative immortality 28

4.5 Hallmark Activating invasion and metastasis....29

4.6 Hallmark Genome instability and mutation....30

4.7 Hallmark Tumor promoting and inflammation....31

5. Magnitude of association between OLP and healthy controls. 32

5.1 Hallmark Sustaining proliferative signaling 32

5.2 Hallmark Evading growth suppressors 33

5.3 Hallmark Resisting cell death 34

5.4 Hallmark Enabling replicative immortality 35

5.5 Hallmark Inducing angiogenesis....36

5.6 Hallmark Activating invasion and metastasis....37

5.7 Hallmark Avoiding immune destruction....38

5.8 Hallmark Deregulating cellular energetics....39

5.9 Hallmark Genome instability and mutation 40

5.10 Hallmark Tumor promoting and inflammation....41

6. Magnitude of association between OLP and healthy controls. 42
6.1 Hallmark Sustaining proliferative signaling 42
6.2 Hallmark Evading growth suppressors 43
6.3 Hallmark Resisting cell death 44
6.4 Hallmark Enabling replicative immortality 45
6.5 Hallmark Activating invasion and metastasis 46
6.6 Hallmark Genome instability and mutation 47
6.7 Hallmark Tumor promoting and inflammation 48
7. Biomarkers roles and hallmarks of cancer 49
8. Risk of bias analysis. 67
9. Analysis of small-study effects. 73
9.1 Hallmark Sustaining proliferative signaling 73
9.2 Hallmark Evading growth suppressors 74
9.3 Hallmark Anti-apoptotic biomarkers 75
9.4 Hallmark Pro-apoptotic biomarkers 76
9.5 Hallmark Activating invasion and metastasis 77
9.6 Hallmark Tumor promoting and inflammation 78
10. List of included studies. 79
11. List of excluded studies with reasons. 90

1. Search strategy

Table S1. Search strategy for each database, number of results, and execution date.

**Database**	**Query/Search Strategy**	**Results/ Items found**	**Search time limits**
PubMed	("Lichen Planus, Oral"[MeSH] or "oral lichen planus"[All Fields] or "olp"[All Fields]) and (malign* or premalign* or “potentially malignant disorder” or “precancer” or “cancer"[All Fields] or "Carcinoma, Squamous Cell"[MeSH] or "squamous cell carcinoma"[All Fields] or "oscc"[All Fields] or “transformation” [All Fields] or "risk"[All Fields] or "progression"[All Fields])	1626	October, 2023
Embase	('oral lichen planus'/exp OR 'oral lichen planus' OR 'olp') AND (‘malign*’ OR ‘premalign*’ OR 'potentially malignant disorder' OR 'precancer'/exp OR 'precancer' OR 'cancer'/exp OR 'cancer' OR 'squamous cell carcinoma'/exp OR 'squamous cell carcinoma' OR 'oscc' OR 'transformation'/exp OR 'transformation' OR 'risk'/exp OR 'risk' OR 'progression')	2388	October, 2023
Web of Science	TS=(oral lichen planus OR olp) AND TS=(malign* or premalign* or potentially malignant disorder or precancer or cancer or squamous cell carcinoma or oscc or transformation or risk or progression)	2209	October, 2023
Scopus	TITLE-ABS-KEY (("oral lichen planus" OR "olp") AND ("malign*" OR "premalign*" OR "potentially malignant disorder" OR "precancer" OR "cancer" OR "squamous cell carcinoma" OR "oscc" OR "transformation" OR "risk" OR "progression"))	1778	October, 2023
Total			8001

2. Table S2. Characteristics of analyzed studies

Study (year)	Country	Publication LanguAge	Recruitment period	Study design	Follow up period (mean; range) Months (min/max)	OLP Sample size	Sex; M (%)/ F; n; Age; y (mean±SD)	Tobacco; n (%)	Alcohol; n (%)	Location of lesions n (M)	OLP clinical lesions									Healthy control group; n	Oral cancer comparison group; n	Biomarkers expression analysis				
											Red; n	White; n	Papular;n	Reticular; n	Erosive; n	Atrophic; n	Bullous; n	Plaque; n	Other; n			Methods	Antibody (nature; dilution; incubation time; temperature)	IHC pattern	IHC cutoff point (%)	Biomarker positivity; n(%)
Pariyawathee et al (2020)	Thailand	English	2015-2017	Retrospective	NR	20	Sex=M= 7 (35%)<br>F=13<br>Age= (47.20±10.85)	NR	NR	Buccal mucosa=16<br>Other =4	10	10	NR	NR	10	10	NR	NR	NR	Yes (20)	Yes (20)	Immunohistochemistry	Anti-CD146 monoclonal antibody; 45 minutes; room temperature	Cell membrane	NR	CD146 =20; (100%)
Kitkhajornkiat et al (2020)	Thailand	English	NR	Retrospective	NR	30	Sex=M= 5 (16.7%)<br>F=25<br>Age=NR	NR	NR	Buccal mucosa=30	15	15	NR	NR	15	NR	NR	NR	NR	Yes (10)	Yes (10)	Immunohistochemistry	4ºC; overnight	Cytoplasm and nucleus	Yes (10%)	Capthepsin – L=30; (100%)
Babiuch et al (2020)	Poland	English	2011-2015	Retrospective	NR	15	Sex=M=6 (40%)<br>F=7<br>Age=60	Yes (13)	Yes (13)	NR	NR	NR	NR	NR	NR	NR	NR	NR	NR	Yes (7)	Yes (14)	Immunohistochemistry	Anti-TNF- polyclonal antibdoy ; room temperature; 60 minutes	NR	Yes (5%)	TNF-α= 15; (100%)
Babiuch et al. (2020)	Poland	English	2011-2015	Retrospective	NR	15	Sex=M=6 (40%)<br>F=7<br>Age=60	Yes (13)	Yes (13)	NR	NR	NR	NR	NR	NR	NR	NR	NR	NR	Yes (7)	Yes (14)	Immunohistochemistry	Anti-IL-1 polyclonal antibody; room temperature; 30 minutes	NR	Yes (5%)	IL-1α=12 (80%)
Babiuch et al (2020)	Poland	English	2011-2015	Retrospective	NR	15	Sex=M=6 (40%)<br>F=7<br>Age=60	Yes (13)	Yes (13)	NR	NR	NR	NR	NR	NR	NR	NR	NR	NR	Yes (7)	Yes (14)	Immunohistochemistry	Anti-IL-6 monoclonal antibdoy; room temperature; 30 minutes	NR	Yes (5%)	IL-6= 12 (80%)
Babiuch et al (2020)	Poland	English	2011-2015	Retrospective	NR	15	Sex=M=6 (40%)<br>F=7<br>Age=60	Yes (13)	Yes (13)	NR	NR	NR	NR	NR	NR	NR	NR	NR	NR	Yes (7)	Yes (14)	Immunohistochemistry	Anti-IL-8 monoclonal antibody; room temperature; 60 minutes	NR	Yes (5%)	IL-8= 8; (53.4%)
Angelin and Nair (2020)	India	Englsih	NR	Retrospective	NR	20	Sex=NR<br>Age=45.8	NR	NR	NR	NR	NR	NR	NR	NR	NR	NR	NR	NR	Yes (20)	Yes (20)	Immunohistochemistry	Polyclonal antibody; room temperature; 30 minutes	NR	NR	Surviving=9 (45%)
Akama et al (2020)	Brazil	English	NR	Longitudinal; restrospective	(3 months- 24 months)	59	Sex=NR<br>Age=NR	NR	NR	Buccal mucosa =50	21	NR	NR	NR	21	NR	NR	NR	Ulcerative=7	Yes<br>(26)	Yes<br>(20)	Immunohistochemistry	NR	Cytoplasm	NR	laminin-332 =21; (35.59%)
Squarzanti et al (2019)	Italy	English	2002-2014	Retrospective	5-11 years	28	Sex= M=13 (46;4%)<br>F=15<br>Age= 54	NR	NR	Buccal mucosa=24; Gingiva=1; Lip=1; Retromolar trygone=1; Tongue=1	18	1	NR	16	1	NR	NR	2	Mixed=9	Yes (10)	Yes (10)	Immunohistochemistry	Monoclonal anti-OPN antibody clone Mab53; 1:200; overnight; 4ºC	Cytoplasm	NR	OPN=19; (67.85%)
Squarzanti et al (2019)	Italy	English	2002-2014	Retrospective	5-11 years	28	Sex= M=13 (46;4%)<br>F=15<br>Age= 54	NR	NR	Buccal mucosa= 24 Gingiva= 1 Lip=1 Retromolar trygone= 1 Tongue=1	18	1	NR	16	1	NR	NR	2	mixed (9)	Yes (10)	Yes (10)	Immunohistochemistry	Monoclonal antibody MCM7 clone 47DC141; 1:300; overnight; 4ºC	NR	Yes (10%)	MCM7=15; (53.57%)
Squarzanti et al (2019)	Italy	English	2002-2014	Retrospective	5-11 years	28	Sex= M=13 (46;4%)<br>F=15<br>Age= 54	NR	NR	Buccal mucosa= 24 Gingiva= 1 Lip=1<br>Retromolar trygone= 1 Tongue=1	18	1	NR	16	1	NR	NR	2	mixed (9)	Yes (10)	Yes (10)	Immunohistochemistry	Monoclonal antibody MIB-1	Nucleous	Yes (10%)	Ki67=14; (50%)
Squarzanti et al (2019)	Italy	English	2002-2014	Retrospective	5-11 years	28	Sex= M=13 (46;4%)<br>F=15<br>Age= 54	NR	NR	Buccal mucosa= 24 Gingiva= 1 Lip=1<br>Retromolar trygone= 1 Tongue=1	18	1	NR	16	1	NR	NR	2	mixed (9)	Yes (10)	Yes (10)	Immunohistochemistry	Monoclonal antibody Clone DO-7	Nucleous	Yes (10%)	P53=23; 82.14%)
Squarzanti et al (2019)	Italy	English	2002-2014	Retrospective	5-11 years	28	Sex= M=13 (46;4%)<br>F=15<br>Age= 54	NR	NR	Buccal mucosa= 24 Gingiva= 1 Lip=1<br>Retromolar trygone= 1 Tongue=1	18	1	NR	16	1	NR	NR	2	mixed (9)	Yes (10)	Yes (10)	Immunohistochemistry	Monoclonal antibody Clone 124	NR	Yes (10%)	Bcl2=15; (53.57%)
Squarzanti et al (2019)	Italy	English	2002-2014	Retrospective	5-11 years	28	Sex= M=13 (46;4%)<br>F=15<br>Age= 54	NR	NR	Buccal mucosa= 24 Gingiva= 1 Lip=1<br>Retromolar trygone= 1 Tongue=1	18	1	NR	16	1	NR	NR	2	mixed (9)	Yes (10)	Yes (10)	Immunohistochemistry	Polyclonal anti-53BP1; 1:1000; 60 minutes; room temperature	NR	NR	53BP1=25; (89.28%)
Squarzanti et al (2019)	Italy	English	2002-2014	Retrospective	5-11 years	28	Sex= M=13 (46;4%)<br>F=15<br>Age= 54	NR	NR	Buccal mucosa= 24 Gingiva= 1 Lip=1<br>Retromolar trygone= 1 Tongue=1	18	1	NR	16	1	NR	NR	2	mixed (9)	Yes (10)	Yes (10)	Immunohistochemistry	Monoclonal antibody Clone C8/144 B	NR	Yes (30%)	CD8=19; (67.86%)
Squarzanti et al (2019)	Italy	English	2002-2014	Retrospective	5-11 years	28	Sex= M=13 (46;4%)<br>F=15<br>Age= 54	NR	NR	Buccal mucosa= 24 Gingiva= 1 Lip=1<br>Retromolar trygone= 1 Tongue=1	18	1	NR	16	1	NR	NR	2	mixed (9)	Yes (10)	Yes (10)	Immunohistochemistry	Monoclonal antibody Clone 4B12	Lymphotytic infiltration	Yes (30%)	CD4=25 (89.28%)
Agarwal et al (2019)	India	English	NR	Retrospective	5 years	30	Sex= M=23(76;67%)<br>F= 7<br>Age= 43;03	NR	NR	Buccal mucosa=29 Lateral border of the tongue=1	6	24	NR	24	6	NR	NR	NR	NR	Yes (10)	NR	Immunohistochemistry	1:20; 180 minutes; room temperature	NR	NR	MMP-2= 30; (100%)
Agarwal et al (2019)	India	English	NR	Retrospective	5 Years	30	Sex= M=23(76;67%)<br>F= 7<br>Age= 43;03	4	NR	Buccal mucosa= 29<br>Lateral border of the tongue= 1	6	24	NR	24	6	NR	NR	NR	NR	Yes (10)	NR	Immunohistochemistry	1:20 180 minutes; room temperature	NR	NR	TIMP-2=30; (100%)
Zhang et al (2018)	China	English	July 2012-December 2015.	Retrospective	NR	117	Sex= M= 45 (38.46%)<br>F=72<br>Age= 52	NR	NR	NR	65	52	NR	52	41	24	NR	NR	NR	Yes (110)	NR	Immunohistochemistry	1:150; room temperature; 120 minutes.	NR	NR	Periostin=56; (47.86%)
Zargaran et al (2018)	Iran	English	NR	Retrospective	NR	20	Sex=M=5 (25%)<br>F=15<br>Age= 47.84± 1.00	NR	NR	Tongue= 2<br>Buccal mucosa= 16<br>Gingiva=2	NR	NR	NR	NR	NR	NR	NR	NR	NR	Yes (15)	Yes (20)	Immunohistochemistry	Monoclonal anti-human phagocytic glycoprotein-I; 60 minutes.	Cell membrane	NR	CD44=20; (100%)
Zargaran et al (2018)	Iran	English	NR	Retrospective	NR	20	Sex=M=5 (25%)<br>F=15<br>Age= 47.84± 1.00	NR	NR	Tongue= 2<br>Buccal mucosa= 16<br>Gingiva=2	NR	NR	NR	NR	NR	NR	NR	NR	NR	Yes (15)	Yes (20)	Immunohistochemistry	Monoclonal anti-human Beta-Catenin; 60 minutes	Cytoplasm and Cell membrane	NR	βCATENIN 20; (100%)
Shiva et al (2018)	Iran	English	NR	Retrospective	NR	32	Sex= M=15 (53.1%)<br>F=17<br>Age= 46±0.81	NR	NR	Gingiva= 13<br>Buccal mucosa= 14<br>Labial=4<br>Floor=1	16	16	NR	NR	16	NR	NR	NR	NR	Yes (8)	NR	Immunohistochemistry	Monoclonal antibody; Clone Do-7; 60 minutes	Cytoplasm and nucleus	Yes (10%)	P53 32; (100%)
Sheelam et al (2018)	India	English	NR	Retrospective	NR	10	NR	NR	NR	NR	NR	NR	NR	NR	NR	NR	NR	NR	NR	Yes (10)	Yes (30)	Immunohistochemistry	NR	Nucleus	Yes (5%)	PCNA 6; (60%)

Perez et al (2018)	Argentina	English	NR	Retrospective	NR	23	NR	NR	NR	NR	14	9	NR	9	14	NR	NR	NR	NR	Yes (7)	NR	Immunohistochemistry	Overnight	Cell membrane	NR	carbonic anhydrase IX= 16; (69.57%)
Enomoto et al (2018)	Japan	English	2014-2016	Retrospective	16 months (3-84 months).	123	Sex= M=25 (20.33%)<br>F=98<br>Age= 60 (23-88)	NR	NR	Buccal mucosa=104<br>Other =19	NR	NR	NR	NR	NR	NR	NR	NR	NR	NR	NR	Immunohistochemistry	Monoclonal antibody clone C8/144B	NR	NR	CD8= 66; (53.66%)
Enomoto et al (2018)	Japan	English	2014-2016	Retrospective	16 months (3-84 months).	123	Sex= M=25 (20.33%)<br>F=98<br>Age=60 (23-88)	NR	NR	Buccal mucosa= 104 Other= 19	NR	NR	NR	NR	NR	NR	NR	NR	NR	NR	NR	Immunohistochemistry	Monoclonal antibody clone H-210; 20 minutes; 105ºC	NR	NR	T-bet+= 76; (61.79%)
Enomoto et al (2018)	Japan	English	2014-2016	Retrospective	16 months (3-84 months).	123	Sex= M=25 (20.33%)<br>F=98<br>Age=60 (23-88)	NR	NR	Buccal mucosa= 104 Other= 19	NR	NR	NR	NR	NR	NR	NR	NR	NR	NR	NR	Immunohistochemistry	Momoclonal antibody clone 236A/E7; 20 minutes; 105ºC	NR	NR	FoxP3+ =60; (48.78%)
Enomoto et al (2018)	Japan	English	2014-2016	Retrospective	16 months (3-84 months).	123	Sex= M=25 (20.33%)<br>F=98<br>Age=60 (23-88)	NR	NR	Buccal mucosa= 104 Other= 19	NR	NR	NR	NR	NR	NR	NR	NR	NR	NR	NR	Immunohistochemistry	NR	NR	NR	CD4 70; (56.91%)
Danielsson et al (2018)	Sweden	English	NR	Retrospective	NR	79	Sex= M=26 (33%)<br>F=53<br>Age= 57 (21-89)	NR	NR	Buccal mucosa= 57<br>Gingiva=11<br>Tongue= 3<br>Palate=2<br>Other=6	NR	NR	NR	NR	NR	NR	NR	NR	NR	Yes (15)	NR	Immunohistochemistry	1:200	NR	Yes (0%)	P16 79; (100%)
Zolfaghari Saravi et al (2017)	Iran	English	NR	Retrospective	NR	52	Sex= M=11 (21.1%) F= 41<br>Age= 45.8±11.7	NR	NR	Tongue=6<br>Buccal mucosa=40<br>Gingiva=3<br>Lip= 1	25	27	NR	27	25	NR	NR	NR	NR	Yes (60)	NR	Immunohistochemistry	Monoclonal Antibody; 1:50.	NR	Yes (10%)	VEGFR-3 52; (100%)
Satelur et al (2017)	India	English	September 2016-October 2016	Retrospective	NR	50	NR	NR	NR	NR	NR	NR	NR	NR	NR	NR	NR	NR	NR	Yes (10)	Yes (10)	Immunohistochemistry	30 minutes; room temperature	Cytoplasm	NR	cathepsin-B 47; (94%)
Liu et al (2017)	China	English	NR	Prospective	NR	10	Sex= M=3 (30%) F=7<br>Age= F= 54 (42-68)<br>M=49 (43-65)	NR	NR	NR	NR	NR	NR	NR	NR	NR	NR	NR	NR	Yes (3)	Yes (4)	Immunohistochemistry	Monoclonal antibody; 4ºC; overnight.	NR	NR	E-cadherine 10; (100%)
Liu et al (2017)	China	English	NR	Prospective	NR	10	Sex= M=3 (30%) F=7<br>Age= F= 54 (42-68)<br>M=49 (43-65)	NR	NR	NR	NR	NR	NR	NR	NR	NR	NR	NR	NR	Yes (3)	Yes (4)	Immunohistochemistry	Monoclonal antibody; 4ºC; overnight.	NR	NR	Vimentin 10; (100%)
Liu et al (2017)	China	English	NR	Prospective	NR	10	Sex= M=3 (30%) F=7<br>Age= F= 54 (42-68)<br>M=49 (43-65)	NR	NR	NR	NR	NR	NR	NR	NR	NR	NR	NR	NR	Yes (3)	Yes (4)	Immunohistochemistry	Monoclonal antibody; 4ºC overnight.	NR	NR	CK19 10; (100%)
Liu et al (2017)	China	English	NR	Prospective	NR	10	Sex= M=3 (30%) F=7<br>Age= F= 54 (42-68)<br>M=49 (43-65)	NR	NR	NR	NR	NR	NR	NR	NR	NR	NR	NR	NR	Yes (3)	Yes (4)	Immunohistochemistry	Monoclonal antibody; 4ºC overnight.	NR	NR	β1 integrin 10; (100%)
Liu et al (2017)	China	English	NR	Prospective	NR	10	Sex= M=3 (30%) F=7<br>Age= F= 54 (42-68)<br>M=49 (43-65)	NR	NR	NR	NR	.NR	NR	NR	NR	NR	NR	NR	NR	Yes (3)	Yes (4)	Immunohistochemistry	Monoclonal antibody; 4ºC overnight.	NR	NR	Nestin 8; (80%)
Jaafari-Ashkavandi and Aslani (2017)	Iran	English	1998-2005	Retrospective	NR	24	Sex= M=11(45.83%)<br>F=13<br>Age= 45.2 ± 14	NR	NR	NR	NR	NR	NR	NR	NR	NR	NR	NR	NR	NR	Yes (23)	Immunohistochemistry	Polyclonal antibody; 1:200; 60 minutes.	Cytoplasm and Cell membrane	Yes (10%)	caveolin-1 14; (58.33%)
Hadzi-Mihailovic et al (2017)	Serbia	English	NR	Retrospective	NR	40	Sex= M=12 (30%)<br>F=28 (70%)<br>Age= 58.3 (33-81).	Yes (15)	Yes (11)	NR	29	11	NR	8	28	NR	1	3	NR	Yes (13)	Yes (12)	Immunohistochemistry	Monoclonal antibody Do7; 1:50; room temperature; 45 minutes.	Nucleus	Yes (1%)	P53=32; (80%)
Ghallab et al (2017)	Egypt	English	May 2015 to December 2015.	Retrospective	NR	30	Sex= F=19<br>M=11 (36.66%)<br>Age= 9 (46-69)	NR	NR	Buccal mucosa=27 Lip=15 Tongue= 21 Gingiva=12 Palate= 6	20	10	NR	10	10	10	NR	NR	NR	Yes (30)	NR	Immunohistochemistry	Polyclonal antibody; room temperature; 30 minutes	Nucleus	NR	Cyclin-D1= 30; (100%)
Bombeccari et al (2017)	Italy	English	March 2001-November 2015	Prospective	19-128 Months<br>Mean= 89.7 (SD=13.9)	18	Sex= F=12 M= 6 (33%)<br>Age= F= 65 (60-71) M= 62 (55-68)	NR	NR	Buccal mucosa= 6 Tongue=10 Gingiva=2	12	9	NR	NR	NR	NR	NR	NR	NR	NR	Yes (18)	Immunohistochemistry	Monoclonal antibody; 1:100; 12 hours;	NR	NR	Cytokeratin-19 =6; (33.33%)
Basheer et al (2017)	India	English	NR	Retrospective	NR	10	Sex= F=7 M=3 (30%)<br>Age= NR	NR	NR	Multifocal=10	4	6	NR	NR	NR	NR	NR	NR	NR	Yes (10)	NR	Immunohistochemistry	Monoclonal antibody clone EP288OY	Nucleus	Yes (5%)	Survivin=1; (10%)
Basheer et al (2017)	India	English	NR	Retrospective	NR	10	Sex= F=7 M=3 (30%)<br>Age= NR	NR	NR	Multifocal=10	4	6	NR	NR	NR	NR	NR	NR	NR	Yes (10)	NR	Immunohistochemistry	Monoclonal antibody Clone D07	Nucleus	Yes (5%)	P53= 4; (40%)
Siponen M et al (2016)	Finland	English	2001-2012	Retrospective	NR	25	Sex=F= 14<br>M=11 (44%)<br>Age= 53 (9-88)	NR	NR	Buccal mucosa=20 Gingiva=2 Palate=2 Tongue=1	NR	NR	NR	NR	NR	NR	NR	NR	8	Yes (14)	NR	Immunohistochemistry	Monoclonal antibody clone E-7; room temperature; 20 minutes.	NR	Yes (5%)	cathepsin-k 18; (72%)
Miri-Moghaddam M and Kadeh H (2016)	Iran	English	NR	Retrospective	10 years	26	Sex= F= 17<br>M= 6 (23.1%)<br>Age= 42.86 (12-80)	NR	NR	Buccal mucosa= 20 Tongue= 5 Lip=1	9	17	NR	17	9	NR	NR	NR	NR	Yes (20)	NR	Immunohistochemistry	Monoclonal antibody; 60 minutes; room temperature	Cytoplasm	Yes (1%)	MMP-10 21; 84.61%
Sridevi et al (2015)	India	English	NR	Retrospective	NR	10	Sex= F= 6 M=4 (40%)<br>Age= 20-60<br>(36.00±8.30)	NR	NR	NR	4	6	NR	6	2	2	NR	NR	NR	Yes (10)	Yes (10)	Immunohistochemistry	1:200; room temperature; 24 hours.	Cell membrane	Yes (10%)	E-cadherin 10; (100%)
Siponen et al (2015)	Finland	English	1990-2001	Retrospective	NR	55	NR	NR	NR	NR	19	36	NR	NR	NR	NR	NR	NR	NR	Yes (23)	NR	Immunohistochemistry	Overnight; 4ºC; 1:30	Cell membrane	NR	Hyaluronan =55; (100%)
Siponen et al (2015)	Finland	English	1990-2001	Retrospective	NR	55	NR	NR	NR	NR	19	36	NR	NR	NR	NR	NR	NR	NR	Yes (23)	NR	Immunohistochemistry	Room temperature; 1 hour; 1:150	Cell membrane	NR	CD44= 49; (89.09%)

Siponen et al (2015)	Finland	English	1990-2001	Retrospective	NR	55	NR	NR	NR	NR	19	36	NR	NR	NR	NR	NR	NR	NR	Yes (23)	NR	Immunohistochemistry	Overnight; 4ºC; 1:75	Cell membrane and Cytoplasm	NR	HAS 1= 53; (96.36%)
Siponen et al (2015)	Finland	English	1990-2001	Retrospective	NR	55	NR	NR	NR	NR	19	36	NR	NR	NR	NR	NR	NR	NR	Yes (23)	NR	Immunohistochemistry	Overnight; 4ºC; 1:120	Cell membrane and Cytoplasm	NR	HAS 2= 52; (94.55%)
Siponen et al (2015)	Finland	English	1990-2001	Retrospective	NR	49	NR	NR	NR	NR	19	36	NR	NR	NR	NR	NR	NR	NR	Yes (23)	NR	Immunohistochemistry	Polyclonal antibody ; Overnight; 4ºC; 1:100	Cell membrane and Cytoplasm	NR	HYAL 1 =48; (97.96%)
Siponen et al (2015)	Finland	English	1990-2001	Retrospective	NR	49	NR	NR	NR	NR	19	36	NR	NR	NR	NR	NR	NR	NR	Yes (23)	NR	Immunohistochemistry	Polyclonal antibody; Overnight; 4ºC; 1:100	Cell membrane and Cytoplasm	NR	HYAL 2 =43; (87.76%)
Shailaja G et al (2015)	India	English	NR	Retrospective	NR	30	Sex= F= 16 (53.3%) M= 14 (46.6%) Age= 40.1 (30-52)	NR	NR	NR	NR	NR	NR	NR	NR	NR	NR	NR	NR	Yes (10)	NR	Immunohistochemistry	Monoclonal antibody; MIB-1 1:70.000; 1hour.	Nucleus	Yes (5%)	Ki67= 22; (73.3%)
Shailaja G et al (2015)	India	English	NR	Retrospective	NR	30	Sex= F= 16 (53.3%) M= 14 (46.6%) Age= 40.1 (30-52)	NR	NR	NR	NR	NR	NR	NR	NR	NR	NR	NR	NR	Yes (10)	NR	Immunohistochemistry	Monoclonal antibody; Clone DO7; 1:70.000; 1 hour.	Nucleus	Yes (5%)	P53 21(70%)
Shailaja G et al (2015)	India	English	NR	Retrospective	NR	30	Sex= F= 16 (53.3%) M= 14 (46.6%) Age= 40.1 (30-52)	NR	NR	NR	NR	NR	NR	NR	NR	NR	NR	NR	NR	Yes (10)	NR	Immunohistochemistry	Monoclonal antibody; Clone 124. 1:70.000; 1 hour.	Cytoplasm	Yes (5%)	Bcl2= 6 (20%)
Shailaja G et al (2015)	India	English	NR	Retrospective	NR	30	Sex= F= 16 (53.3%) M= 14 (46.6%) Age= 40.1 (30-52)	NR	NR	NR	NR	NR	NR	NR	NR	NR	NR	NR	NR	Yes (10)	NR	Immunohistochemistry	Monoclonal primary antibody 1:70.000; 1 hour.	Cytoplasm	Yes (5%)	Bax =1; (56.67%)
Pigatti et al (2015)	Brazil	English	2006-2011	Retrospective	NR	14	Sex=F=4 (28.57%) M=10 (71.43%) Age= 50.5 (32-76)	NO	NO	Buccal mucosa =5 Gingiva =4 Tongue=5	NR	NR	NR	NR	NR	NR	NR	NR	NR	Yes (9)	NR	Immunohistochemistry	Monoclonal antibody clone 124; 1:200	NR	Yes (10%)	Bcl2= 1 7.14%)
Pigatti et al (2015)	Brazil	English	2006-2011	Retrospective	NR	14	Sex=F=4 (28.57%) M=10 (71.43%) Age= 50.5 (32-76)	NO	NO	Buccal mucosa =5 Gingiva =4 Tongue=5	NR	NR	NR	NR	NR	NR	NR	NR	NR	Yes (9)	NR	Immunohistochemistry	Monoclonal antibody clone MIB; 1:200	NR	Yes (10%)	Ki67 14 (100%)
Li et al (2015)	China	English	NR	Retrospective	NR	51	Sex= F= 29 M=22 (43.13%) Age= 53.34 (36-72)	NR	NR	NR	27	24	NR	24	27	27	NR	NR	NR	Yes (40)	NR	Immunohistochemistry	Polyclonal antibody; 4ºC; overnight 1:75	Cytoplasm	Yes (5%)	hMSH2 27/51 (52;94%)
Kouhsoltani M et al (2015)	Iran	English	NR	Retrospective	NR	40	NR	NR	NR	NR	20	20	NR	20	20	NR	NR	NR	NR	NO	Yes (20)	Immunohistochemistry	1:800; 20 minutes; room temperature	Cell membrane	Yes (0%)	erbB-2 17/40 (42.5%)
Hu X-S et al (2015)	China	Chinese	2009-2010	Retrospective	NR	53	Sex=M=13 (24.52%) F=40 Age= 42. 3(14-65)	NR	NR	NR	NR	53	NR	NR	NR	NR	NR	NR	NR	Yes (18)	Yes (45)	Immunohistochemistry	Monoclonal antibody clone D14E12; overnight; 4ºC; 1:800	Nucleus and Cytoplasm	Yes (25%)	P38 MAPK 8/11 (72.72%)
Goel et al (2015)	India	English	NR	Retrospective	NR	60	Sex= M=32 (53.3%) F=28 Age = NR (19-69)	NR	NR	NR	43	17	NR	17	43	NR	NR	NR	NR	Yes (!0)	Yes (10	Immunohistochemistry	Polyclonal antibody clone SC260; overnight; room temperature	Nucleus and Cytoplasm	NR	CDK4 48/60 (80%)
Goel et al (2015)	India	English	NR	Retrospective	NR	60	Sex= M=32 (53.3%) F=28 Age = NR (19-69)	NR	NR	NR	43	17	NR	17	43	NR	NR	NR	NR	Yes (!0)	Yes (!0)	Immunohistochemistry	Monoclonal antibody clone DB152 R; overnight; room temperature	Nucleus and Cytoplasm	NR	P16 41/60 (68.33%)
Du and Li (2015)	China	English	2005-2012	Retrospective	NR	52	Sex= M= 28 (53.84%) F= 24 Age= 52.6 (38-73)	NR	NR	NR	NR	NR	NR	NR	NR	NR	NR	NR	NR	Yes (41)	NR	Immunohistochemistry	Monoclonal antibody clone BA0474; 1:200; 30 minutes; 37ºC.	Cell membrane and Cytoplasm	NR	E-cadherin (27/52) 51.92%
Baghaei et al (2015)	Iran	English	NR	Retrospective	NR	24	Sex= M=11 (45.83%) F=13 (54.16%) Age= 34.95±13.13	NR	NR	Gingiva=9 Buccal mucosa=10 Tongue=2 Floor of the mouth=3 Alveolar ridge=0	NR	NR	NR	NR	NR	NR	NR	NR	NR	Yes (24)	Yes (24)	Immunohistochemistry	Monoclonal antibody clone PM 354; Room temperature; 30 minutes.	NR	Yes (10%)	P21 24/24 (100%)
Arreaza et al (2015)	Venezuela	English	NR	Retrospective	NR	31	Sex= F= 26 (84.6%) M=19.3% Age= 59.5 ±11.7	NR	NR	Buccal mucosa = 16 Lip=3 Alveolar ridge =1 Palate=3 Tongue =3 Gingiva =2 Other=3	NR	NR	NR	NR	NR	NR	NR	NR	NR	NR	NR	Immunohistochemistry	Monoclonal antibody clone Do-7; 1:50; 30 minutes.	Nucleus	Yes (30%)	P53 24/31 (77.4.%)
Salehinejad et al (2014)	Iran	English	NR	Retrospective	NR	15	NR	NR	NR	NR	NR	NR	NR	NR	NR	NR	NR	NR	NR	Yes (8)	Yes (45)	Immunohistochemistry	Monoclonal antibody NCL-p16-432; 1:40;	Cytoplasm	Yes (10%)	P16 4/15 (26.67%)
Rivarola de Gutierrez et al (2014)	Argentina	English	NR	Retrospective	NR	31	Sex= F= 30 (90.9%) M=3 (9.09%) Age= 40-85 (61.6)	Yes (5)	NO	Buccal mucosa =27 Tongue =3 Palate =3	49	20	NR	NR	37	12	NR	20	NR	Yes (4)	NR	Immunohistochemistry	Monoclonal antibody clone 34βB4; overnight; 4ºC; 1:100	NR	NR	Cytokeratin-1 19/31 (61.29%)
Rivarola de Gutierrez et al (2014)	Argentina	English	NR	Retrospective	NR	31	Sex F= 30 (90.9%) M=3 (9.09%) Age= 40-85 (61.6)	Yes (6)	No	Buccal mucosa =27 Tongue =3 Palate =3	49	20	NR	NR	37	12	NR	20	NR	Yes (4)	NR	Immunohistochemistry	Monoclonal antibody NCL-CK13 clone KS-1A3; overnight; 4ºC; 1:200	NR	NR	Cytokeratin-13 31/31 (100%)
Rivarola de Gutierrez et al (2014)	Argentina	English	NR	Retrospective	NR	32	Sex F= 30 (90.9%) M=3 (9.09%) Age= 40-85 (61.6)	Yes (5)	No	Buccal mucosa =27 Tongue =3 Palate =3	49	20	NR	NR	37	12	NR	20	NR	Yes (4)	NR	Immunohistochemistry	Monoclonal antibody; cloneLL002; overnight; 4ºC; 1:100	NR	NR	Cytokeratin-14 31/32 (96.88%)
Redder et al (2014)	India	English	NR	Retrospective	NR	64	NR	NR	NR	NR	NR	NR	NR	NR	NR	NR	NR	NR	NR	NR	NR	Immunohistochemistry	NR	NR	Yes (5%)	PCNA 64/64 (100%)
Cortés-Ramírez et aL (2014)	Spain	English	January 2006-june 2008	Retrospective	43.5 months (20-78 months)	44	Sex= F=30 (60.2%) M=14 (31.8%) Age= 56.4 (31-82)	NR	NR	NR	NR	NR	NR	NR	NR	NR	NR	NR	NR	Yes	Yes	Immunohistochemistry	Monoclonal antibody clone 31G7: 1 hour; room temperature	Membrane and Cytoplasm	Yes (0%)	EGFR 35/44 (79;54%)
Arreaza et al (2014)	Venezuela	English	2003-2012	Retrospective	. NR	65	NR	NR	NR	NR	NR	NR	NR	NR	NR	NR	NR	NR	NR	NR	NR	Immunohistochemistry	1:50; 30 minutes	NR	Yes (1%)	Bcl-2 56/65 (86;15%)

Arreaza et al (2014)	Venezuela	English	2003-2012	Retrospective	NR	65	NR	NR	NR	NR	NR	NR	NR	NR	NR	NR	NR	NR	NR	NR	NR	Immunohistochemistry	1:50; 30 minutes	NR	Yes (1%)	COX-2 43/65 (66;15%)
Xu et al (2013)	China	English	1985-2010	Retrospective	5 Years every six months 5.0 1.3-14.5	89	Sex= F=70 (78.7%) M=19 (21.3%) Age= 44.7 (9-74)	Yes= 15 (21.4%)	Yes= 17 (24.3%)	NR	NR	NR	NR	NR	NR	NR	NR	NR	NR	Yes (10)	Yes (6)	Immunohistochemistry	Monoclonal antibody ab52492; 1:250;	Cell membrane and Cytoplasm	NR	ALDH1 27/89 (30;33%)
Sun et al (2013)	China	English	1988 - 2012	Retrospective	56 months. 16-170 every 6 months	110	Sex= F=84 M=26 Age=46 (9-74)	Yes= 12 (10.9%)	Yes= 25 (22.72%)	NR	NR	NR	NR	NR	NR	NR	NR	NR	NR	Yes (10)	Yes (6)	Immunohistochemistry	Monoclonal antibody; 1:50	Cell membrane and Cytoplasm	Yes (5%)	CD133 37/110 (33.63%)
Prodromidis et al (2013)	Greece	English	2005-2008	Retrospective	NR	40	NR	NR	NR	NR	NR	NR	NR	NR	NR	NR	NR	NR	NR	Yes (9)	Yes (10)	Immunohistochemistry	Monoclonal antibody 4960 1:100	Nucleus and Cytoplasm	Yes (0%)	Akt 37/40 (92.5%)
Prodromidis et al (2013)	Greece	English	2005-2008	Retrospective	NR	39	NR	NR	NR	NR	NR	NR	NR	NR	NR	NR	NR	NR	NR	Yes (10)	Yes (10)	Immunohistochemistry	Monoclonal antibody 2976 1:100	Nucleus and Cytoplasm	Yes (0%)	mTOR 4/39 (10.25%)
Prodromidis et al (2013)	Greece	English	2005-2008	Retrospective	NR	40	NR	NR	NR	NR	NR	NR	NR	NR	NR	NR	NR	NR	NR	Yes (10)	Yes (10)	Immunohistochemistry	Monoclonal antibody 2211 1:100	Nucleus and Cytoplasm	Yes (0%)	Ribosomal S6 36/40 (33.64%)
Oliveira Alves et al (2013)	Brazil	English	NR	Retrospective	NR	65	NR	NR	NR	Buccal mucosa=65	NR	NR	NR	NR	NR	NR	NR	NR	NR	Yes (5)	Yes (19)	Immunohistochemistry	Monoclonal antibody clone DO-7; 1:50; 4ºC; 1H	Nucleus	Yes (5%)	P53 60/65 (92.31%)
Oliveira Alves et al (2013)	Brazil	English	NR	Retrospective	NR	65	NR	NR	NR	Buccal mucosa=65	NR	NR	NR	NR	NR	NR	NR	NR	NR	Yes (5)	Yes (19)	Immunohistochemistry	Monoclonal antibody Clone D-11; 1:50; 4ºC; 18H	Nucleus and Cytoplasm	Yes (5%)	SUMO-1 23/65 (35.38%)
Oliveira Alves et al (2013)	Brazil	English	NR	Retrospective	NR	65	NR	NR	NR	Buccal mucosa=65	NR	NR	NR	NR	NR	NR	NR	NR	NR	Yes (5)	Yes (19)	Immunohistochemistry	Monoclonal antibody clone SMP14; 1:50; 4ºC; 18H	Nucleus	Yes (5%)	MDM2 65/65 (100%)
Nafarzadeh et al (2013)	Iran	English	NR	Retrospective	NR	30	NR	NR	NR	NR	15	15	NR	15	15	NR	NR	NR	NR	Yes (20)	Yes (11)	Immunohistochemistry	1:100 ; overnight; 4ºC	NR	Yes (0%)	BAX 21/30 (70%)
Nafarzadeh et al (2013)	Iran	English	NR	Retrospective	NR	30	NR	NR	NR	NR	15	15	NR	15	15	NR	NR	NR	NR	Yes (20)	Yes (11)	Immunohistochemistry	1:100 ; overnight; 4ºC	NR	Yes (0%)	BCL2 0/30
Nafarzadeh et al (2013)	Iran	English	2005-2010	Retrospective	NR	30	Sex= F=21 (70%) M=9 (30%) Age= 45.23± 2.44	NR	NR	Buccal mucosa=22 Lip= 1 Tongue=4 Gingiva=2 Palate=1	6	24	NR	24	6	NR	NR	NR	NR	Yes (20)	NR	Immunohistochemistry	Monoclonal antibody ab55479; 60 minutes	Nucleus and Cytoplasm	Yes (0%)	Smad-3 30/30 (100%)
Ma et al (2013)	China	English	1990-2010	Retrospective	Min= 54 months (16-175)	96	Sex= F=74 (77.08%) M=22 (22.92%) Age= 46.4 (26-74)	Yes (17)	Yes (20)	NR	NR	NR	NR	NR	NR	NR	NR	NR	NR	Yes (10)	Yes (6)	Immunohistochemistry	Monoclonal antibody ab14389; 1:150	Nucleus	NR	Bmi-1 40/96 (41.67%)
Li et al (2013)	China	English	March 2007 and May 2011.	Retrospective	NR	56	Sex= F=3 M=53 (94.64%) Age= 30.7 (20-56)	Yes (56)	NR	Buccal mucosa=56	NR	NR	NR	NR	NR	NR	NR	NR	NR	Yes (10)	NR	Immunohistochemistry	Monoclonal antibody; 30 minutes	Cytoplasm	Yes (10%)	CXCL9 14/33 (42.42%)
Li and Cui (2013)	China	English	2009-2011	Retrospective	NR	33	Sex= F=15 (45.45%) M=18 (54.54%) Age= 20-65	NR	NR	NR	NR	NR	NR	NR	NR	NR	NR	NR	NR	Yes (10)	Yes (38)	Immunohistochemistry	NR	Cytoplasm and Cell membrane	Yes (10%)	Cox-2 14/33 (42.42%)
Li and Cui (2013)	China	English	2009-2011	Retrospective	NR	33	Sex= F=15 (45.45%) M=18 (54.54%) Age= 20-65	NR	NR	NR	NR	NR	NR	NR	NR	NR	NR	NR	NR	Yes (10)	Yes (38)	Immunohistochemistry	NR	Cytoplasm	Yes (10%)	MMP-7 14/33 (30.30%)
Zhao; M et al (2012)	China	Chinese	2005-2010	Retrospective	NR	20	NR	NR	NR	NR	10	10	NR	NR	NR	NR	NR	NR	NR	Yes (5)	Yes (20)	Immunohistochemistry	Monoclonal antibody; 1:50; 1 hour; room temperature	NR	Yes (5%)	EGFR 20/20 (100%)
Leyva-Huerta et al (2012)	Mexico	English	NR	Retrospective	NR	21	Sex= F= 16 (76.2%) M=5 (23.8%) Age=22 -74 (56+12.3).	Yes: 6 (28.6%)	Yes: 3 (14.3%)	Buccal mucosa =11 Lip= 8 Tongue=1 Palate= 1	17	4	NR	4	10	NR	NR	NR	NR	Yes (4)	Yes (16)	Immunohistochemistry	Monoclonal antibody NCI-953DO-7; room temperature; 40 minutes	Nucleus	Yes (5%)	P53 15/21 (71.43%)
Leyva-Huerta et al (2012)	Mexico	English	NR	Retrospective	NR	21	Sex= F= 16 (76.2%) M=5 (23.8%) Age=22 -74 (56+12.3).	Yes : 6 (28.6%)	Yes: 3 (14.3%)	Buccal mucosa =11 Lip= 8 Tongue=1 Palate= 1	17	4	NR	4	10	NR	NR	NR	NR	Yes (4)	Yes (16)	Immunohistochemistry	Monoclonal antibody mouse clone 124; room temperature 40 minutes	NR	Yes (5%)	Bcl2 0/21 (0%)
Danielsson et al (2012)	Sweden	English	NR	Retrospective	NR	18	Sex= F=14 (77.7%) M= 6 Age=61(43-79)	Yes (1)	NR	Buccal mucosa = 18	7	13	NR	NR	NR	NR	NR	NR	NR	Yes (18)	NR	Immunohistochemistry	Polyclonal antibody b 15191; 1:100	NR	NR	Cox 2=17/18 (94.4%)
Sudha VM and Hemavathy S (2011)	India	English	NR	Retrospective	NR	10	NR	NR	NR	NR	NR	NR	NR	NR	NR	NR	NR	NR	NR	NR	Yes (30)	Immunohistochemistry	1 hour; room temperature;	NR	Yes (10%)	Bcl2 10/10 (100%)

Poomsawat et al (2011)	Thailand	English	2001-2006	Retrospective	NR	23	Sex= M=6 F=17 (73.91%) Age=24-72	NR	NR	NR	NR	NR	NR	NR	NR	NR	NR	NR	NR	Yes (10)	NR	Immunohistochemistry	Polyclonal antibody SC260; 2 hours; room temperature; 1:50.	Nucleus	NR	CDK4 18/23 (78.26%)
Poomsawat et al (2011)	Thailand	English	2001-2006	Retrospective	NR	23	Sex= M=6 F=17 (73.91%) Age=24-72	NR	NR	NR	NR	NR	NR	NR	NR	NR	NR	NR	NR	Yes (10)	NR	Immunohistochemistry	1:10	Nucleus and Cytoplasm	NR	P16 15/23 (65.22%)
Poomsawat et al (2011)	Thailand	English	2001-2006	Retrospective	NR	23	Sex= M=6 F=17 (73.91%) Age=24-72	NR	NR	NR	NR	NR	NR	NR	NR	NR	NR	NR	NR	Yes (10)	NR	Immunohistochemistry	Polyclonal antibody SC177; 2 hours; room temperature; 1:50	Nucleus	NR	CDK16 8/23 (34.78%)
Montebugnoli et al (2011)	Italy	English	NR	Retrospective	12 to 24 months (mean 16.8± 6 months)	56	Sex= F=42 M=14 Age= 28 -78 (56 ± 12)	Yes (11)	Yes (5)	Buccal mucosa= 53 Palate=3	19	37	NR	37	NR	19	NR	NR	NR	Yes (14)	NR	Immunohistochemistry	Monoclonal antibody E6H4	Nucleus and Cytoplasm	Yes (5%)	P16=36/56 (64.29%)
Martín-Ezquerra G et al (2011)	Spain	English	1989-2007	Retrospective	NR	13	Sex= M=7 F=6 Age= 70 (45-94)	Yes (6) 43%	Yes (1) 7%	NR	3	10	NR	10	3	NR	NR	NR	NR	Yes (14)	Yes (51)	Immunohistochemistry	1:50	Nucleus	Yes (5%)	CKS1B 0/13 (0%)
Krauss E et al (2011)	Germany	English	NR	Retrospective	NR	26	NR	NR	NR	NR	NR	. NR	NR	NR	NR	NR	NR	NR	NR	NR	NR	Immunohistochemistry	Monoclonal antibody 57B; 1:100	NR	NR	MAge-A 0/26 (0%)
Klosek et al (2011)	Poland	English	2007-2009	Retrospective	NR	34	Sex= F=24 M=10 Age= 61.5 ±8.2 (52 -68)	Yes (18)	NR	Buccal mucosa=23 Tongue=7 alveolar= 3 palate=1	NR	NR	NR	NR	NR	NR	NR	NR	NR	Yes (12)	NR	Immunohistochemistry	Polyclonal antibody; Overnight; 4ºC;1:50;	Membrane and Cytoplasm	NR	C-MET 24/34 (70.58%)
Zyada MM and Fikry HE (2010)	Egypt	English	January 2000- May 2007	Retrospective	2 years	43	Sex= F=26 M=14 Age= 58.5 (31-77)	NR	NR	Buccal mucosa= 23 tongue= 10 gingiva=5 lip=1 floor=1	24	19	NR	NR	NR	NR	NR	NR	NR	Yes (20)	NR	Immunohistochemistry	NR	Cell membrane	NR	Syndecan-1 13/40 (32.5%)
Shi; Y-J et al (2010)	China	Chinese	January 2003-May 2008	Retrospective	NR	30	Sex= M=9 F=21 Age=26-72 (48)	NR	NR	Buccal mucosa=12 Tongue=18	9	21	NR	NR	NR	NR	NR	NR	NR	Yes (15)	NR	Immunohistochemistry	1:200	Cytoplasm	Yes (10%)	Caspase- 8 29/30 (96.67%)
Shi; Y-J et al (2010)	China	Chinese	January 2003-May 2008	Retrospective	NR	30	Sex= M=9 F=21 Age=26-72 (48)	NR	NR	Buccal mucosa=12 Tongue=18	9	21	NR	NR	NR	NR	NR	NR	NR	Yes (15)	NR	Immunohistochemistry	Monoclonal antibody; 1:400	Cytoplasm and nucleus	Yes (10%)	NF-KBp65 28/30 (93.3%)
Shi; Y-J et al (2010)	China	Chinese	January 2003-May 2008	Retrospective	NR	30	Sex= M=9 F=21 Age=26-72 (48)	NR	NR	Buccal mucosa=12 Tongue=18	9	21	NR	NR	NR	NR	NR	NR	NR	Yes (15)	NR	Immunohistochemistry	Polyclonal antibody 1:400;	Cytoplasm and nucleus	Yes (10%)	RIP 26/30 (86.67%)
Shi P et al (2010)	China	English	1978-2007	Retrospective	5.1 (1.33-19.25 y)	110	Sex= F=84 (76.4) M=26 (23.6) Age=45.3 (12.3) 9-74	Yes (7)	Yes (9)	NR	NR	NR	NR	NR	NR	NR	NR	NR	NR	NR	Yes (9)	Immunohistochemistry	Monoclonal antibody D2-40 1:150; Overnight; 4ºC	Cell membrane and Cytoplasm	NR	Podoplanin 48/110 (43.63%)
Shi P et al (2010)	china	English	1978-2007	Retrospective	5.1 (1.33-19.25 y)	100	Sex= F=84 (76.4) M=26 (23.6) Age=45.3 (12.3) 9-74	Yes (7)	Yes (9)	NR	NR	NR	NR	NR	NR	NR	NR	NR	NR	NR	Yes (9)	Immunohistochemistry	Monoclonal antibody; 1:40; Overnight; 4ºC	Cell membrane and Cytoplasm	NR	ABCG2 23/100 (23%)
Martín-Ezquerra G et al (2010)	Spain	English	1989-2007	Retrospective	NR	13	Sex=M=7 (53.84%) F=6 (46.15%) Age=70(45-94)	Yes (6)	Yes (1)	NR	3	10	NR	10	3	NR	NR	NR	NR	Yes (14)	Yes (51)	Immunohistochemistry	NR	Nucleus	NR	C-myc 0/13 (0%)
Martín-Ezquerra G et al (2010)	Spain	English	1989-2007	Retrospective	NR	13	Sex=M=7 (53.84%) F=6 (46.15%) Age=70(45-94)	Yes (6)	Yes (1)	NR	3	10	NR	10	3	NR	NR	NR	NR	Yes (14)	Yes (3)	Immunohistochemistry	NR	Nucleus	NR	P53 2/!3(15.38%)
Martín-Ezquerra G et al (2010)	Spain	English	1989-2007	Retrospective	NR	13	Sex=M=7 (53.84%) F=6 (46.15%) Age=70(45-94)	Yes (6)	Yes (1)	NR	3	10	NR	10	3	NR	NR	NR	NR	Yes (14)	Yes (3)	Immunohistochemistry	NR	Nucleus	NR	Cyclin-D1 5/13 (38.46%)
Martín-Ezquerra G et al (2010)	Spain	English	1989-2007	Retrospective	NR	13	Sex=M=7 (53.84%) F=6 (46.15%) Age=70(45-94)	Yes (6)	Yes (1)	NR	3	10	NR	10	3	NR	NR	NR	NR	Yes (14)	Yes (51)	Immunohistochemistry	NR	Cytoplasm and membrane	NR	EGFR 8/13 (61.54%)
Martín-Ezquerra G et al (2010)	Spain	English	1989-2007	Retrospective	NR	13	Sex=M=7 (53.84%) F=6 (46.15%) Age=70(45-94)	Yes (6)	Yes(1)	NR	3	10	NR	10	3	NR	NR	NR	NR	Yes (14)	Yes (51)	Immunohistochemistry	NR	NR	NR	ErBb-2 (15.38%)
Hsieh PC et al (2010)	Taiwan	English	NR	Retrospective	NR	16	Sex= F=1 M=15 (93.75) Age= 46.7±16.5	NR	NR	Lip=2 Buccal mucosa =14	NR	NR	NR	NR	NR	NR	NR	NR	NR	Yes (9)	Yes (43)	Immunohistochemistry	1:150; 30 minutes	NR	Yes (10%)	BUBR1 16/16 (100%)
Danielsson et al (2010)	Sweden	English	2007-2008	Retrospective	NR	22	Sex=F=12 M=10 (45.45%) Age=F=60 (range 42–79 M=47 (range 34–59).	NR	NR	NR	18	4	NR	3	7	11	NR	1	NR	Yes (10)	Yes (8)	Immunohistochemistry	Monoclonal ab55478; 1:50	Nucleus	Yes (25%)	Smad-2 22/22 (100%)
Danielsson et al (2010)	Sweden	English	2007-2008	Retrospective	NR	22	Sex=F=12 M=10 (45.45%) Age=F=60 (range 42–79 M=47 (range 34–59).	NR	NR	NR	18	4	NR	3	7	11	NR	1	NR	Yes (10)	Yes (8)	Immunohistochemistry	Monoclonal ab55479; 1:200	Nucleus	Yes (25%)	Smad-3 20/22 (90.9%)
Danielsson et al (2010)	Sweden	English	2007-2008	Retrospective	NR	22	Sex=F=12 M=10 (45.45%) Age=F=60 (range 42–79 M=47 (range 34–59).	NR	NR	NR	18	4	NR	3	7	11	NR	1	NR	Yes (10)	Yes (8)	Immunohistochemistry	Monoclonal antibody ab49178 ; 1:40	Cytoplasm	Yes (25%)	Smad-4 11/22 (50%)
Danielsson et al (2010)	Sweden	English	2007-2008	Retrospective	NR	22	Sex=F=12 M=10 (45.45%) Age=F=60 (range 42–79 M=47 (range 34–59).	NR	NR	NR	18	4	NR	3	7	11	NR	1	NR	Yes (10)	Yes (8)	Immunohistochemistry	Monoclonal antibody ab5825; 1:100	Nucleus	Yes (25%)	Smad-7 22/22 (100%)
Cortés-Ramírez DA et al (2010)	Spain	English	January 2003 to June 2008	Retrospective	Mean=43.5 months; range= 20 to 78 months	44	Sex=F=30 (68.2%) M=14 (31.8%) Age=56.4 (31-82)	NR	NR	NR	18	26	NR	NR	NR	NR	NR	NR	NR	NR	NR	Immunohistochemistry	Monoclonal antibody (clon SP21)1:100; 1 hour; room temperature	Cytoplasm	Yes (20%)	Cox-2 44/44 (100%)

De Sousa FA et al (2009)	Brazil	English	NR	Retrospective	NR	24	NR	NR	NR	NR	NR	NR	NR	NR	NR	NR	NR	NR	NR	NR	NR	Immunohistochemistry	1:200	NR	Yes (5%)	Bax 12/24 (50%)
De Sousa FA et al (2009)	Brazil	English	NR	Retrospective	NR	24	NR	NR	NR	NR	NR	NR	NR	NR	NR	NR	.NR	NR	NR	NR	NR	Immunohistochemistry	Monoclonal antibody Clone 124; 1:50	NR	Yes (5%)	Bcl2 4/24 (16.67%)
Oluwadara O et al (2009)	USA	English	NR	Retrospective	NR	13	NR	NR	NR	NR	NR	NR	NR	NR	NR	NR	NR	NR	NR	Yes (6)	Yes (27)	Immunohistochemistry	1:250; 4ºC: overnight.	NR	Yes (10%)	LCK 13/13 (100%)
Oluwadara O et al (2009)	USA	English	NR	Retrospective	NR	13	NR	NR	NR	NR	NR	NR	NR	NR	NR	NR	NR	NR	NR	Yes (6)	Yes (27)	Immunohistochemistry	4ºC: overnight.	NR	Yes (10%)	SURVIVIN 8/13 (64.3%)
Oluwadara O et al (2009)	USA	English	NR	Retrospective	NR	13	NR	NR	NR	NR	NR	NR	NR	NR	NR	NR	NR	NR	NR	Yes (6)	Yes (27)	Immunohistochemistry	1:50; 4ºC: overnight.	NR	Yes (10%)	PI-3K 9/13 (69.23%)
Ogmundsdóttir HM et al (2009)	Iceland	English	1900-2000	Retrospective	2-12 years	7	Sex=F=6 M=1 (14.28%)<br>Age=63.42	Yes=2	NR	Buccal mucosa=3 tongue=2 gingiva=2	4	3	NR	NR	1	3	NR	NR	2	NR	NR	Immunohistochemistry	Monoclonal antibody DO-7 monoclonal anti-human	NR	NR	P53 6/7 (85.71%)
Ogmundsdóttir HM et al (2009)	Iceland	English	1987-1996	Retrospective	15.9 (12–22)	45	Sex= M=17 F=28<br>Age=52 (23-81)	NR	NR	NR	NR	NR	NR	NR	NR	NR	NR	NR	NR	NO	Yes (54)	Immunohistochemistry	Monoclonal antibody DO-7	Nucleus AND Cytoplasm	NR	P53=16/45 (35.5%)
Jacques CM et al (2009)	Brazil	English	NR	Retrospective	NR	17	Sex= F=15 (88.24%)<br>M=2<br>Age= 56.77	NR	NR	Buccal mucosa=13 lip=3 tongue=1	5	12	0	6	4	0	NR	6	NR	NR	NR	Immunohistochemistry	Monoclonal antibody M7002: 1:50;12H	NR	NR	Cytokeratin-10 16/17 (94.11%)
Jacques CM et al (2009)	Brazil	English	NR	Retrospective	NR	23	Sex= F= 21 M=2 Age= 56.77	NR	NR	Buccal mucosa=20 Gingiva=2 lip=3 tongue=1	7	16	0	8	5	1	NR	9	NR	NR	NR	Immunohistochemistry	Monoclonal antibody M7003; 1:25; 12H	NR	NR	Cytokeratin-14 23/23 (100%)
Jacques CM et al (2009)	Brazil	English	NR	Retrospective	NR	23	Sex= M=1 F=22<br>Age= 56.77	NR	NR	Buccal mucosa=17 Gingiva=2 lip=2 tongue=1	7	16	0	7	5	1	NR	10	NR	NR	NR	Immunohistochemistry	Monoclonal antibody NCL-LL002; 1:20; 12H	NR	NR	Cytokeratin-13 16/23(69.56%)
Jacques CM et al (2009)	Brazil	English	NR	Retrospective	NR	21	Sex= F=20 M=1<br>Age= 56.77	NR	NR	Buccal mucosa=16 Gingiva=2 lip=2 tongue=1	6	15	1	5	5	1	NR	9	NR	NR	NR	Immunohistochemistry	1:100; 12H	NR	NR	Cytokeratin-19 4/21 (19.04%)
Hadzi-Mihailovic et al (2009)	Serbia	English	2003-2006	Retrospective	NR	40	Sex=F=28 (70%) M=12 (30%);<br>Age=58.3 (33 - 81)	Yes (15)	Yes (11)	NR	29	11	NR	8	28	NR	1	3	NR	Yes (13)	Yes (12)	Immunohistochemistry	Monoclonal antibody clone 124; overnight; 4ºc; 1:100	NR	Yes Yes (5%)	Bcl-2 28/40 (70%)
González Moles MA et al (2009)	Spain	English	January 2001-December 2004	Retrospective	NR	50	Sex=F=32 (64%) M=18 (36%);<br>Age=55.1 (31–79)	Yes (10)	Yes(12)	Multiple=26	29	21	NR	NR	29	NR	NR	NR	NR	Yes (26)	No	Immunohistochemistry	4ºC; overnight	Cytoplasm AND Nucleus	Yes Yes (0%)	Substance-P 48/50 (96%)
González Moles MA et al (2009)	Spain	English	January 2001-December 2004	Retrospective	NR	50	Sex=F=32 (64%) M=18 (36%);<br>Age=55.1 (31–79)	Yes (10)	Yes(12)	NR	29	21	NR	NR	29	NR	NR	NR	NR	Yes (26)	No	Immunohistochemistry	4ºC; overnight	Cytoplasm AND Cell membrane	Yes (0%)	NK-IR 5/50 (10%)
González Moles MA et al (2009)	Spain	English	January 2001-December 2004	Retrospective	NR	50	Sex=F=32 (64%) M=18 (36%);<br>Age=55.1 (31–79)	Yes (10)	Yes(12)	NR	29	21	NR	NR	29	NR	NR	NR	NR	Yes (26)	No	Immunohistochemistry	Monoclonal antibody Mib-1 clone; 4ºC; overnight ;	Cytoplasm AND Nucleus	Yes (0%	Ki-67 44/50 (88%)
González Moles MA et al (2009)	Spain	English	January 2001-December 2004	Retrospective	NR	50	Sex=F=32 (64%) M=18 males (36%);<br>Age=55.1 (31–79)	Yes (10)	Yes(12)	NR	29	21	NR	NR	29	NR	NR	NR	NR	Yes (26)	no	Immunohistochemistry	4ºC; overnight	Cytoplasm AND Nucleus	Yes Yes (0%)	Caspase-3 25/50 (50%)
de Sousa; F A (2009)	Brazil	English	NR	Retrospective	NR	24	NR	NR	NR	NR	NR	NR	NR	NR	NR	NR	NR	NR	NR	no	Yes (24)	Immunohistochemistry	Monoclonal antibody PC10 clone; 1:300; 18 hours; 4ºC;	NR	Yes (5%)	PCNA 14/24 (58.3%)
de Sousa; F A (2009)	Brazil	English	NR	Retrospective	NR	24	NR	NR	NR	NR	NR	NR	NR	NR	NR	NR	NR	NR	NR	NO	Yes (24)	Immunohistochemistry	Monoclonal antibody DO-7 clone; 1:200;4ºC; 18 hourS	NR	Yes (5%)	P53 10/24 (41.67%)
de Sousa; F A (2009)	Brazil	English	NR	Retrospective	NR	24	NR	NR	NR	NR	NR	NR	NR	NR	NR	NR	NR	NR	NR	NO	Yes (24)	Immunohistochemistry	1:200; 4ºC; 18 hours	NR	Yes (5%)	Bax 12/24 (50%)
de Sousa; F A (2009)	Brazil	English	NR	Retrospective	NR	24	NR	NR	NR	NR	NR	NR	NR	NR	NR	NR	NR	NR	NR	NO	Yes (24)	Immunohistochemistry	Monoclonal antibody 124 clone; 1:50; 4ºC; 18 hours	NR	Yes (5%)	Bcl2 4/24 (16.67%)24

Gonzalez-Moles MA et al (2008)	Spain	English	January 2001- december 2004	Retrospective	NR	51	Sex= F=33 (64.7%) M= 18 (35.3%) Age= 55.4 (31–79).	Yes (10)	Yes (12)	Multiple=27	29	22	NR	NR	29	NR	NR	NR	NR	Yes (26)	NO	Immunohistochemistry	Monoclonal antibody DO7 clone; overnight; 4ºC//pAb 240 clone; 1:500	NR	Yes (0%	P53 8/51 (15.68%)
Gonzalez-Moles MA et al (2008)	Spain	English	January 2001- december 2004	Retrospective	NR	51	Sex= F=33 (64.7%) M= 18 (35.3%) Age= 55.4 (31–79).	Yes (10)	Yes (12)	Multiple=27	29	22	NR	NR	29	NR	NR	NR	NR	Yes (26)	NO	Immunohistochemistry	Monoclonal antibody Mib-1 clone; 4ºC; overnight	NR	Yes (0%)	Ki67 17/51 (33.3%)
Gonzalez-Moles MA et al (2008)	Spain	English	January 2001- december 2004	Retrospective	NR	51	Sex= F=33 (64.7%) M= 18 (35.3%) Age= 55.4 (31–79).	Yes (10)	Yes (12)	Multiple=27	29	22	NR	NR	29	NR	NR	NR	NR	Yes (26)	NO	Immunohistochemistry	Monoclonal antibody Clone C92; overnight; 4ºC	NR	Yes (0%)	Caspase-3 14/51 (27.45%)
Chen Y et al (2008)	China	English	1984-2005	Retrospective	NR	27	Sex= F=14 M=13 (48.14%) Age=47	NR	NR	NR	12	15	NR	NR	NR	NR	NR	NR	NR	Yes (11)	Yes (15)	Immunohistochemistry	Monoclonal antibody ab-4; overnight; 4ºc	Cytoplasm	NR	MMP2 20/27 (74.07%)
Chen Y et al (2008)	China	English	1984-2005	Retrospective	NR	27	Sex= F=14 M=13 (48.14%) Age=47	NR	NR	NR	12	15	NR	NR	NR	NR	NR	NR	NR	Yes (11)	Yes (15)	Immunohistochemistry	ab-9; overnight; 4ºc	Cytoplasm	NR	MMP9 20/27 (74.07%)
Chen Y et al (2008)	China	English	1984-2005	Retrospective	NR	27	Sex= F=14 M=13 (48.14%) Age=47	NR	NR	NR	12	15	NR	NR	NR	NR	NR	NR	NR	Yes (11)	Yes (15)	Immunohistochemistry	Monoclonal antibody ab-4 overnight; 4ºc	Cytoplasm	NR	MTI-MMP 22/27 (81.48%)
Chen Y et al (2008)	China	English	1984-2005	Retrospective	NR	27	Sex= F=14 M=13 (48.14%) Age=47	NR	NR	NR	12	15	NR	NR	NR	NR	NR	NR	NR	Yes (11)	Yes (15)	Immunohistochemistry	Monoclonal antibody ab-5 overnight; 4ºc	Cytoplasm	NR	TIMP-2 16/27 (29.62%)
Chen Y et al (2008)	China	English	1984-2005	Retrospective	NR	27	Sex= F=14 M=13 (48.14%) Age=47	NR	NR	NR	12	15	NR	NR	NR	NR	NR	NR	NR	Yes (11)	Yes (15)	Immunohistochemistry	overnight; 4ºc	Cytoplasm	NR	TGF-B1 24/27 (88.88%)
Bascones-Ilundain C et al (2008)	Spain	English	1999 -February 2003	Retrospective	NR	32	Sex= F=20 (62.5%) M=12 (37.5%) Age= 54.2 ± 12.4.	Yes (6)	Yes (8)	Buccal mucosa=19 Gingiva=5 Lip=5 Tongue=2 Unknown=1	18	14	NR	14	18	NR	NR	NR	NR	NR	NR	Immunohistochemistry	Polyclonal antibody; 500; 60 minutes	NR	Yes (0%)	Bax 22/32(68.75%)
Bascones-Ilundain C et al (2008)	Spain	English	1999 -February 2003	Retrospective	NR	32	Sex= F=20 (62.5%) M=12 (37.5%) Age= 54.2 ± 12.4.	Yes (6)	Yes (8)	Buccal mucosa =19 Gingiva=5 Lip=5 Tongue=2 Unknown=1	18	14	NR	14	18	NR	NR	NR	NR	NR	NR	Immunohistochemistry	Monoclonal antibody (clone c92–605); 1 : 1000; 60 minutes	NR	Yes (0%)	Caspase-3 21/32 (68.62%)
Yao X et al (2007)	China	Chinese	2011	Retrospective	NR	60	NR	NR	NR	NR	NR	NR	NR	NR	NR	NR	NR	NR	NR	NR	NR	Immunohistochemistry	NR	NR	NR	NF-KBp65 55/60 (91;67%)
Neppelberg E and Johannessen AC (2007)	Norway	English	1978 to 2003	Retrospective	6–225 (mean 91)	56	NR	NR	NR	NR	NR	NR	NR	NR	NR	NR	NR	NR	NR	Yes (7)	Yes (7)	Immunohistochemistry	Monoclonal antibody clone HECD-1; 1:1500	Cytoplasm	NR	E-cadherin 56/56 (100%)
Neppelberg E and Johannessen AC (2007)	Norway	English	1978 to 2003	Retrospective	6–225 (mean 91)	63	NR	NR	NR	NR	NR	NR	NR	NR	NR	NR	NR	NR	NR	Yes (7)	Yes (5)	Immunohistochemistry	Monoclonal Ab (mAb) Cox-2; clone 229 1:600	Cytoplasm	NR	Cox-2 63/63 (100%)
Thongprasom K et al (2006)	Thailand	English	NR	Retrospective	NR	18	Sex= F=14 M=4 Age= 39.11 ±12.3 (17-67)	NR	NR	Gingiva=2 Buccal mucosa= 16	18	0	NR	NR	12	6	NR	NR	NR	Yes (20)	no	Immunohistochemistry	Polyclonal antibody; 1:50; 4ºC: overnight	NR	NR	TNF-α 16/18 (88.88%)
González-Moles MA et al (2006)	Spain	English	January 2001- december 2004	Retrospective	NR	51	Sex= F= 33 (64.7%) M= 18 (35.3%) Age= 55.4 (31-79)	Yes (10)	Yes (12)	Multiple:27	29	NR	NR	NR	29	NR	NR	NR	NR	Yes (26)	no	Immunohistochemistry	Monoclonal antibody Do-7 clone4ºC: overnight	NR	Yes (0%)	P53 8/51 (15.68%)
González-Moles MA et al (2006)	Spain	English	January 2001- december 2004	Retrospective	NR	51	Sex= F= 33 (64.7%) M= 18 (35.3%) Age= 55.4 (31-79)	Yes (10)	Yes (12)	Multiple:27	29	NR	NR	NR	29	NR	NR	NR	NR	Yes (26)	No	Immunohistochemistry	1:200;4ºC: overnight	NR	Yes (0%)	P21 28/51 (54.9%)
González-Moles MA et al (2006)	Spain	English	January 2001- december 2004	Retrospective	NR	51	Sex= F= 33 (64.7%) M= 18 (35.3%) Age= 55.4 (31-79)	Yes (10)	Yes (12)	Multiple:27	29	NR	NR	NR	29	NR	NR	NR	NR	Yes (26)	No	Immunohistochemistry	Monoclonal antibody Mib-1;4ºC: overnight	NR	Yes (0%)	Ki67 17/51 (33.3%)
González-Moles MA et al (2006)	Spain	English	January 2001- december 2004	Retrospective	NR	51	Sex= F= 33 (64.7%) M= 18 (35.3%) Age= 55.4 (31-79)	Yes (10)	Yes (12)	Multiple:27	29	NR	NR	NR	29	NR	NR	NR	NR	Yes (26)	No	Immunohistochemistry	4ºC: overnight	NR	Yes (0%)	Bcl2 4/51 (7.84%)
González-Moles MA et al (2006)	Spain	English	January 2001- december 2004	Retrospective	NR	51	Sex= F= 33 (64.7%) M= 18 (35.3%) Age= 55.4 (31-79))	Yes (10)	Yes (12)	Multiple:27	29	NR	NR	NR	29	NR	NR	NR	NR	Yes (26)	No	Immunohistochemistry	4ºC: overnight	NR	Yes (0%)	Caspase-3 14/51 (27.45%)
Acay RR et al (2006)	Brazil	English	NR	Retrospective	NR	22	NR	NR	NR	NR	NR	NR	NR	NR	NR	NR	NR	NR	NR	no	no	Immunohistochemistry	Monoclonal antibody Do-7	Nucleus	NR	P53 20/22 (90.9%)
Acay RR et al (2006)	Brazil	English	NR	Retrospective	NR	22	NR	NR	NR	NR	NR	NR	NR	NR	NR	NR	NR	NR	NR	No	no	Immunohistochemistry	Monoclonal antibody Mib-1	Nucleus	NR	Ki67 21/22 (95.45%)
Lee JJ et al (2005)	Taiwan	English	NR	Retrospective	NR	56	Sex= F=30 M=26 (46.42%) Age=48 (15-78)	Yes (18)	Yes (10)	Buccal mucosa=38 Tongue=10 Gingiva=5 Palate=2 lip=1	NR	NR	NR	NR	NR	NR	NR	NR	NR	Yes (20)	Yes (38)	Immunohistochemistry	Monoclonal antibody Do-7; 1:100; overnight; 4ºC	Nucleus	Yes (5%)	P53 16/56 (28.57%)

Brunotto M et al (2005)	Argentina	English	NR	Retrospective	NR	8	Sex=M=4 F= 4 (50%)<br>Age= 23-75	NR	NR	Tongue=4 Buccal mucosa=4	NR	NR	NR	NR	NR	NR	NR	NR	NR	Yes (10)	Yes (3)	Immunohistochemistry	1:100; overnight; 4ºC	NR	NR	Cytokeratine 14 8/8 (100%)
Brunotto M et al (2005)	Argentina	English	NR	Retrospective	NR	8	Sex=M=4 F= 4 (50%)<br>Age= 23-75	NR	NR	Tongue=4 Buccal mucosa=4	NR	NR	NR	NR	NR	NR	NR	NR	NR	Yes (10)	Yes (3)	Immunohistochemistry	1:100overnight; 4ºC	NR	NR	P53 4/8 (45%)
Brunotto M et al (2005)	Argentina	English	NR	Retrospective	NR	8	Sex=M=4 F= 4 (50%)<br>Age= 23-75	NR	NR	Tongue=4 Buccal mucosa=4	NR	NR	NR	NR	NR	NR	NR	NR	NR	Yes (10)	Yes (3)	Immunohistochemistry	1:100overnight; 4ºC	NR	NR	P21 1/8 (10%)
Brunotto M et al (2005)	Argentina	English	NR	Retrospective	NR	8	Sex=M=4 F= 4 (50%)<br>Age= 23-75	NR	NR	Tongue=4 Buccal mucosa=4	NR	NR	NR	NR	NR	NR	NR	NR	NR	Yes (10)	Yes (3)	Immunohistochemistry	1:100overnight; 4ºC	NR	NR	Bcl2 4/8 (53%)
Bascones C et al (2005)	Spain	English	November 1999 and February 2003	Retrospective	NR	32	Sex= F=20 (62.5%)<br>M=12<br>Age=34-81	NR	NR	Buccal mucosa=19 Gingiva= 5 lip=5 Tongue=2	14	18	NR	18	NR	NR	NR	NR	NR	Yes (20)	NO	Immunohistochemistry	NR	Cytoplasm	Yes (0%)	Caspase-3 14/32 (43.75%)
Bascones C et al (2005)	Spain	English	November 1999 and February 2003	Retrospective	NR	32	Sex= F=20 (62.5%)<br>M=12<br>Age=34-81	NR	NR	Buccal mucosa=19 Gingiva= 5 lip=5 Tongue=2	14	18	NR	18	NR	NR	NR	NR	NR	Yes (20)	NO	Immunohistochemistry	Polyclonal antibody ; 1:500	NR	Yes (0%)	Bax 22/32 (68.75%)
Bascones C et al (2005)	Spain	English	November 1999 and February 2003	Retrospective	NR	32	Sex= F=:20 M=12<br>Age= 34—81	NR	NR	Buccal mucosa=19 Gingiva= 5 lip=5 Tongue=2	14	18	NR	18	NR	NR	NR	NR	NR	Yes (20)	NO	Immunohistochemistry	NR	Nucleus	NR	P21 19/32 (59.38%)
Shen L et al (2004)	China	Chinese	January 2000-December 2003	Retrospective	NR	50	Sex=M=14 F=36 Age= 21-71 (43)	NR	NR	Tongue=26 Buccal mucosa= 20 lip=4	20	30	NR	NR	NR	NR	NR	NR	NR	Yes (10)	No	Immunohistochemistry	NR	Cell membrane and Cytoplasm	Yes (5%)	Fas D 44/50 (88%)
Shen L et al (2004)	China	Chinese	January 2000-December 2003	Retrospective	NR	50	Sex=M=14 F=36 Age= 21-71 (43)	NR	NR	Tongue=26 Buccal mucosa= 20 lip=4	20	30	NR	NR	NR	NR	NR	NR	NR	Yes (10)	No	Immunohistochemistry	Polyclonal antibody	Cell membrane and Cytoplasm	Yes (5%)	Fas L 25/50 (50%)
Shen L et al (2004)	China	Chinese	January 2000-December 2003	Retrospective	NR	50	Sex=M=14 F=36 Age= 21-71 (43)	NR	NR	Tongue=26 Buccal mucosa= 20 lip=4	20	30	NR	NR	NR	NR	NR	NR	NR	Yes (10)	no	Immunohistochemistry	Monoclonal antibody	Cytoplasm	Yes (5%)	Granzyme B 34/50 (68%)
Pirkić A et al (2004)	Croatia	English	NR	Retrospective	NR	30	Age= 48.1±12.7	NR	NR	NR	NR	NR	NR	NR	NR	NR	NR	NR	NR	NO8	NO	Immunohistochemistry	Monoclonal antibody; 1:50; 1 hour: room temperature	NR	NR	ErbB-2 30/30 (100%)
Fan Y et al (2004)	China	Chinese	NR	Retrospective	NR	18	NR	NR	NR	NR	10	8	NR	NR	NR	NR	NR	NR	NR	Yes (10)	Yes (22)	Immunohistochemistry	Polyclonal antibody; 1:100; 37ºC; 30 minutes.	NR	Yes (0%)	Bax 13/18n(72.2%)
Fan Y et al (2004)	China	Chinese	NR	Retrospective	NR	18	NR	NR	NR	NR	10	8	NR	NR	NR	NR	NR	NR	NR	Yes (10)	Yes (22)	Immunohistochemistry	Monoclonal antibody; 1:50; 37ºC; 30 minutes.	NR	Yes (0%)	Bcl2 3/18 (16.67%)
Bloor BK et al (2003)	United Kingdom	English	NR	Retrospective	NR	5	NR	NR	NR	Buccal mucosa=2 Tongue=1 Gingiva=2	NR	NR	NR	NR	NR	NR	NR	NR	NR	Yes (6)	Yes (7)	Immunohistochemistry	*Monoclonal antibody; 10 minutes; 4ºC*	NR	NR	K2e 0/0 (0%)
Bloor BK et al (2003)	united kingdom	English	NR	Retrospective	NR	5	NR	NR	NR	Buccal mucosa=2 Tongue=1 Gingiva=2	NR	NR	NR	NR	NR	NR	NR	NR	NR	Yes (6)	Yes (7)	Immunohistochemistry	Monoclonal antibody LHK1; 1:10; 10 minutes; 4ºC	NR	NR	K1 5/5 (100%)
Bloor BK et al (2003)	united kingdom	English	NR	Retrospective	NR	5	NR	NR	NR	Buccal mucosa=2 Tongue=1 Gingiva=2	NR	NR	NR	NR	NR	NR	NR	NR	NR	Yes (6)	Yes (7)	Immunohistochemistry	Monoclonal antibody rkse 60; 1:10;10 minutes; 4ºc	NR	NR	K10 5/5 (100%)
Taniguchi Y et al (2002)	japan	English	1990-1999	Retrospective	NR	44	Sex= F=26 M=17 Age= 54.7 ± 7.8 (26 –72).	.	.	Buccal mucosa= 38 lip=2 Palate=1 Tongue=1	19	25	NR	25	NR	19	NR	NR	NR	Yes (10)	.	Immunohistochemistry	Monoclonal antibody DO-7; 1:100	NR	Yes (5%)	P53 28/44 (63.63%)
Tanda N et al (2000)	japan	English	NR	Retrospective	NR	10	Sex= F=9 (90%) M=1 (10%)<br>Age= 59	NR	NR	NR	NR	NR	NR	NR	NR	NR	NR	NR	NR	Yes (9)	no	Immunohistochemistry	Anti-wild type p53 Monoclonal antibody (mAb) Ab-5	Nucleus	NR	P53=10/10 (100%)
Tanda N et al (2000)	japan	English	NR	Retrospective	NR	10	Sex= F=9 (90%) M=1 (10%)<br>Age= 59	NR	NR	NR	NR	NR	NR	NR	NR	NR	NR	NR	NR	Yes (9)	No	Immunohistochemistry	Monoclonal antibody clone 187	Nucleus	NR	P21=10/10 (100%)
Tanda N et al (2000)	Japan	English	NR	Retrospective	NR	10	Sex= F=9 (90%) M= 1 (10%)<br>Age= 59	NR	NR	NR	NR	NR	NR	NR	NR	NR	NR	NR	NR	Yes (9)	NR	Immunohistochemistry	Monoclonal antibody clone IF2	nuclei	NR	MDM2=10/10 (100%)

Tanda N et al (2000)	Japan	English	NR	Retrospective	NR	10	Sex= F=9 (90%) M= 1 (10%)<br>Age= 59	NR	NR	NR	NR	NR	NR	NR	NR	NR	NR	NR	NR	Yes (9)	NR	Immunohistochemistry	Monoclonal antibody clone 124	nuclei	NR	Bcl2=10/10 (100%)
Nagao Y et al (2000)	Japan	English	April 1994 and October 1998	Retrospective	NR	31	Sex= F=25 M=6<br>Age= 61.5	NR	NR	NR	NR	NR	NR	NR	NR	NR	NR	NR	NR	NR	NR	Immunohistochemistry	Monoclonal antibody	NR	NR	CD45=15/31 (48.38%)
Nagao Y et al (2000)	Japan	English	April 1994 and October 1998	Retrospective	NR	31	Sex= F=25 M=6<br>Age= 61.5	NR	NR	NR	NR	NR	NR	NR	NR	NR	NR	NR	NR	NR	NR	Immunohistochemistry	NR	NR	NR	CD20=15/31 (48.38%)
Ali A et al (2000)	United Kingdom	English	NR	Retrospective	NR	6	NR	NR	NR	NR	NR	NR	NR	NR	NR	NR	NR	NR	NR	NR	NR	Immunohistochemistry	Monoclonal antibody; 1:10; 45 min.	Cell membrane and Cytoplasm	NR	5T4 oncofoetal antigen 6/6 (100%)
van der Velden LA et al (1999)	The Netherlands	English	NR	Retrospective	4–5 years.	3	NR	NR	NR	NR	NR	NR	NR	NR	NR	NR	NR	NR	NR	NO	Yes (103)	Immunohistochemistry	Monoclonal antibody RV202	NR	Yes (25%)	VIMENTIN 3/3 (100%)
Girod SC et al (1998)	Germany	English	1986-1991	Retrospective	4–5 years.	3	NR	NR	NR	NR	NR	NR	NR	NR	NR	NR	NR	NR	NR	NO	Yes (103)	Immunohistochemistry	Monoclonal antibody DO7; 30; overnight 4ºC	NR	NR	P53 wild type 15/23 (65.21%)
Girod SC et al (1998)	Germany	English	1986-1991	Retrospective	4–5 years.	3	NR	NR	NR	NR	NR	NR	NR	NR	NR	NR	NR	NR	NR	NO	Yes (103)	Immunohistochemistry	Monoclonal antibody PAb 1801; 200; OVERNIGHT; 4ºC	NR	NR	P53 mutant type 4/23 (17.39%)
Girod SC et al (1998)	Germany	English	1986-1991	Retrospective	4–5 years.	3	NR	NR	NR	NR	NR	NR	NR	NR	NR	NR	NR	NR	NR	NO	Yes (103)	Immunohistochemistry	Monoclonal antibody IF-2; room temperature	NR	NR	MDM2 8/23 (34.78%)
Girod SC et al (1998)	Germany	English	1986-1991.	Retrospective	4–5 years.	3	NR	NR	NR	NR	NR	NR	NR	NR	NR	NR	NR	NR	NR	NO	Yes (103)	Immunohistochemistry	Monoclonal antibody RB Ab 1 room temperature	NR	NR	RB 22/23 (95.65%)
Yadav M et al (1997)	Malasya	English	NR	Retrospective	NR	NR	NR	NR	NR	Buccal mucosa=9	NR	NR	NR	NR	NR	NR	NR	NR	NR	Yes (7)	Yes (51)	Immunohistochemistry	Monoclonal antibody 6A5G3 1:40; 4ºC overnight	NR	NR	HHV-6- encoded antigens 9/9 (100%)
Muraki Y et al (1997)	Japan	English	NR	Retrospective	NR	20	NR	Tob .	Alch .	gingival	NR	NR	NR	NR	NR	NR	NR	NR	NR	Yes (20)	Yes (55)	Immunohistochemistry	Polyclonal antibody; 1:200; 20 minutes; 20-22ºC	NR	NR	Fas D 18/20 (90%)
Zhang L et al (1996)	Canada	English	NR	Retrospective	NR	18	NR	Yes (13)	NR	NR	NR	NR	NR	NR	NR	NR	NR	NR	NR	Yes (11)	NR	Immunohistochemistry	Monoclonal antibody DO-7;	NR	Yes (5%)	P53 9/18 (50%)
Younes F et al (1996)	United Kingdom	English	NR	Retrospective	NR	8	NR	NR	NR	NR	NR	NR	NR	NR	NR	NR	NR	NR	NR	NR	NR	Immunohistochemistry	1:50; 4ºC; 10 minutes.	NR	NR	TNF 8/8 (100%)
Martínez-Lara I et al (1996)	Spain	English	NR	Retrospective<br>.	NR	5	NR	NR	NR	NR	NR	NR	NR	NR	NR	NR	NR	NR	NR	Yes (10)	Yes (10)	Immunohistochemistry	Monoclonal antibody PC10	NR	NR	PCNA 4/5 (80%)
Kilpi A et al (1996)	Australia	English	NR	Retrospective<br>.	NR	32	NR	NR	NR	NR	NR	NR	NR	NR	NR	NR	NR	NR	NR	Yes (14)	Yes (9)	Immunohistochemistry	Monoclonal antibody; 1:40; 12 hours to 15 hours; 4°C	NR	NR	erbB-2 8/32 (25%)
Girod SC et al (1995)	Germany	English	NR	Retrospective	NR	14	NR	NR	NR	NR	NR	NR	NR	NR	NR	NR	NR	NR	NR	Yes (2)	Yes (110)	Immunohistochemistry	Monoclonal antibody if-2; 1:75; 4ºc; overnight.	Nuclear	NR	MDM2 5/14 (35.71%)
Girod SC et al (1995)	Germany	English	NR	Retrospective	NR	14	NR	NR	NR	NR	NR	NR	NR	NR	NR	NR	NR	NR	NR	Yes(2)	Yes (110)	Immunohistochemistry	Monoclonal antibody 1801; 1:30; 4ºc; overnight	nuclear	NR	P53 9/14 (64.29%)
Girod SC et al (1994)	Germany	English	NR	Retrospective	NR	21	NR	NR	NR	NR	NR	NR	NR	NR	NR	NR	NR	NR	NR	Yes (17)	Yes (144)	Immunohistochemistry	Monoclonal antibody Do7; 1:100; 12 hours; 4ºc	NR	NR	P53 4/21 (19.08%)
Favia G et al (1994)	Italy	Italian	1984-1991	Retrospective	NR	11	Sex= M=7 (63.63%) F=4 (36.37%)<br>Age= 62.54 (40-74)	NR	NR	Multicentric=4 Buccal mucosa=5 Gingiva= 5 Palate=3 Lip= 9 Tongue=18.	NR	NR	NR	NR	NR	NR	NR	NR	NR	NR	NR	Immunohistochemistry	NR	NR	NR	EGFR 11/11 (100%)
Tibin K Baby et al (2022)	India	English	NR	Retrospective	NR	30	Sex= F=23 M=7 Age= (24-65)	NR	NR	Buccal mucosa=30	15	15	NR	15	13	.	2	NR	NR	Yes (10)	NR	Immunohistochemistry	Monoclonal antibody anticox-2 clone SP21 1H room temperature	Cytoplasm and nuclear	NR	COX-2 30/30 (100%)
Radwan-Oczko; M et al (2022)	Poland	English	2015-2019	Retrospective	NR	25	Sex= M=21 (84%) F=4<br>Age= 57 (65.5 ±9.4)	NR	NR	Buccal mucosa=14	5	20	NR	NR	NR	NR	NR	NR	NR	NR	NR	Immunohistochemistry	Monoclonal antibody DO-7 (clone DO-7) overnight; 4ºC	NR	Yes (5%)	P53 7/25 (28%)

Radwan-Oczko; M et al (2022)	Poland	English	2015-2019	Retrospective	NR	25	Sex= M=21 (84%) F=4<br>Age= 57 (65.5 ±9.4)	NR	NR	Buccal mucosa=14	5	20	NR	NR	NR	NR	NR	NR	NR	NR	NR	Immunohistochemistry	Monoclonal antibody clone 1D6 overnight; 4ºC	NR	Yes (5%)	TOPO I 18/25 (72%)
Radwan-Oczko; M et al (2022)	Poland	English	2015-2019	Retrospective	NR	25	Sex= M=21 (84%) F=4<br>Age= 57 (65.5 ±9.4)	NR	NR	Buccal mucosa=14	5	20	NR	NR	NR	NR	NR	NR	NR	NR	NR	Immunohistochemistry	Monoclonal antibody clone JPB24 overnight; 4ºC	NR	Yes (5%)	HSP90 10/25 (40%)
Radwan-Oczko; M et al (2022)	Poland	English	2015-2019	Retrospective	NR	25	Sex= M=21 (84%) F=4<br>Age= 57 (65.5 ±9.4)	NR	NR	Buccal mucosa=14	5	20	NR	NR	NR	NR	NR	NR	NR	NR	NR	Immunohistochemistry	Monoclonal antibody clone NCH-38; overnight; 4ºC	NR	Yes (5%)	E-cadherine 15/25 (60%)
Wang Q-M et al (2022)	China	English	2013-2019	Retrospective	NR	48	NR	NR	NR	NR	20	28	NR	28	NR	20	NR	NR	NR	Yes (15)	Yes (46)	Immunohistochemistry	Polyclonal antibody 1:200 4ºC overnight	NR	NR	IL-27 48/48 (100%)
Celestina et al (2023)	India	English	NR	Retrospective	NR	30	Sex=NR<br>Age= 47 (16-73)	NR	NR	NR	NR	NR	NR	NR	NR	NR	NR	NR	NR	NR	NR	Immunohistochemistry	1:600; 45 minutes.	Nucleus and Cytoplasm	NR	Regucalcin 4/30 (13.33%)
Baddevithana et al (2023)	Sri Lanka	English	NR	Retrospective	NR	26	NR	NR	NR	NR	NR	NR	NR	NR	NR	NR	NR	NR	NR	Yes (0)	Yes (1)	Immunohistochemistry	Monoclonal antibody; Clone DCS-50 Sigma; 1:100; 2 hours; room temperature	NR	NR	P16 8/26 (29%)
Aghili et al (2023)	Iran	English	2011-2018	Retrospective	NR	23	Sex= M=42 F=57<br>Age= 50.7 (15 – 83)	NR	NR	NR	NR	NR	NR	NR	NR	NR	NR	NR	NR	Yes (16)	Yes (23)	Immunohistochemistry	Monoclonal antibody 32084: 1:500	Cytoplasm	Yes (10%)	PAXILLIN 2/23 (8%)
Costa Lago et al (2020)	Brazil	English	2007- 2017	Retrospective	NR	33	Sex= M=11 (33.3%)<br>F=22<br>Age=46.9	NO	NO	Buccal mucosa=25; Tongue=16; Palate=7; Gingiva=6	18	15	NR	15	17	NR	1	NR	NR	NR	NR	Immunohistochemistry	NR	Cell membrane	Yes (1%)	PD-L1 11/33 (33.33%)
Costa Lago et al (2020)	Brazil	English	2007 - 2017	Retrospective	NR	33	Sex= M=11 (33.3%)<br>F=22<br>Age=46.9	NO	NO	Buccal mucosa=25; Tongue=16; Palate=7; Gingiva=6	18	15	NR	15	17	NR	1	NR	NR	NR	NR	Immunohistochemistry	NR	Cell membrane and Cytoplasm	Yes (1%)	PD-L2 32/33 (96.96%)
Du et al (2011)	China	English	NR	Retrospective	NR	60	Sex= M= 17 (28.3%)<br>F= 43<br>Age= 44.8	NO	NO	NR	24	36	NR	36	24	NR	NR	NR	NR	Yes (10)	NR	Immunohistochemistry	Monoclonal antibody	NR	NR	PD-L1 50/60 (83.33%)
Du et al (2011)	China	English	NR	Retrospective	NR	60	Sex= M= 17 (28.3%)<br>F= 43<br>Age= 44.8	NO	NO	NR	24	36	NR	36	24	NR	NR	NR	NR	Yes (10)	NR	Immunohistochemistry	Monoclonal antibody	NR	NR	PD-L2 51/60 (85%)

3. Differential expression of the hallmarks of cancer in patients with OLP.

3.1 Hallmark Sustaining proliferative signaling

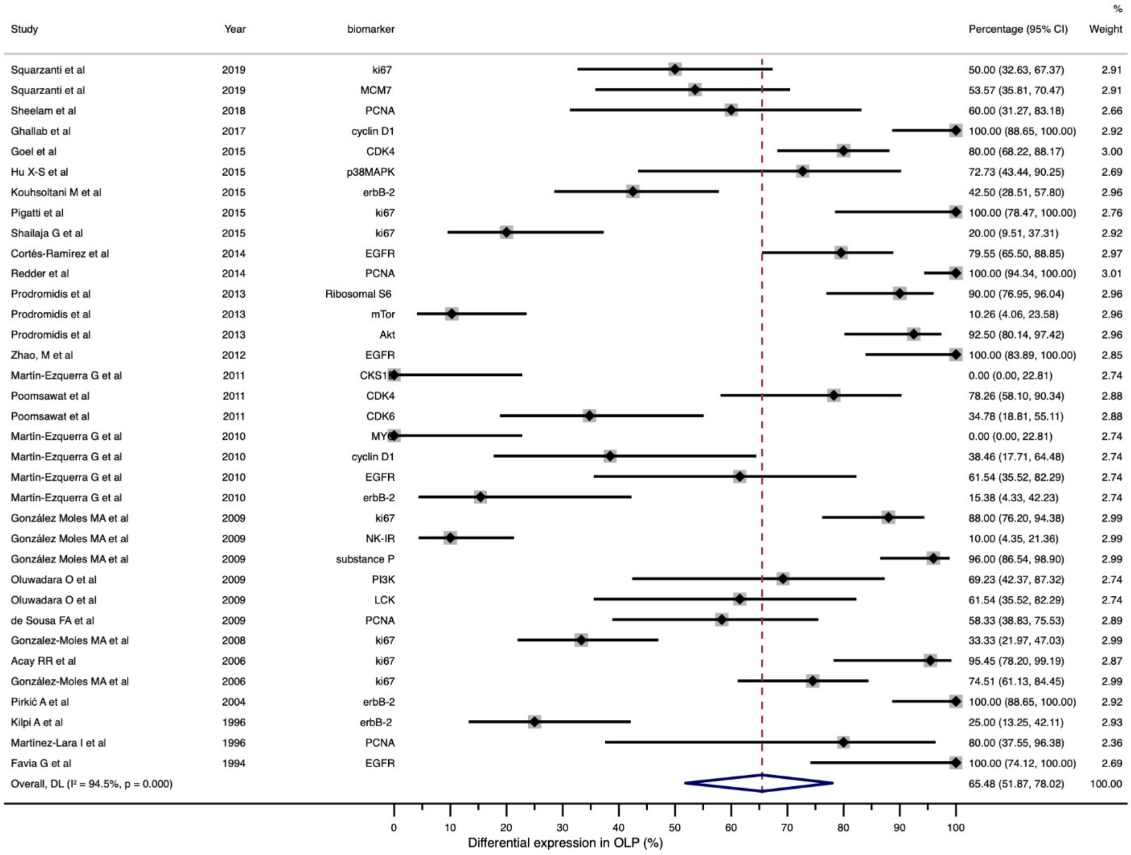

Figure S1. Forest plot graphically representing the differential expression of biomarkers on the hallmark of cancer sustaining proliferation -using pooled proportions as ES metric, expressed as percentage- among OLP patients. ES, effect size; CI, confidence interval; Random-effects model.

3.2 Hallmark Evading growth suppressors

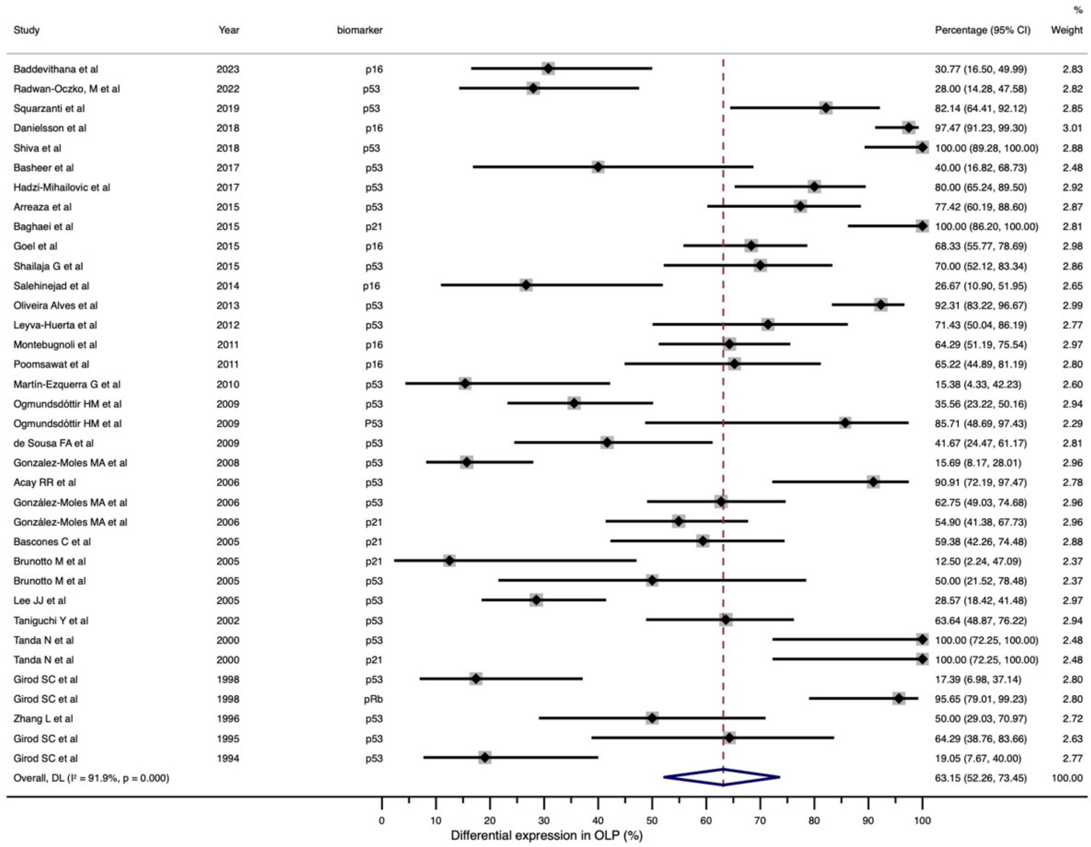

Figure S2. Forest plot graphically representing the differential expression of biomarkers on the hallmark of evading growth suppressors -using pooled proportions as ES metric, expressed as percentage- among OLP patients. ES, effect size; CI, confidence interval; Random-effects model.

3.3 Hallmark Resisting cell death

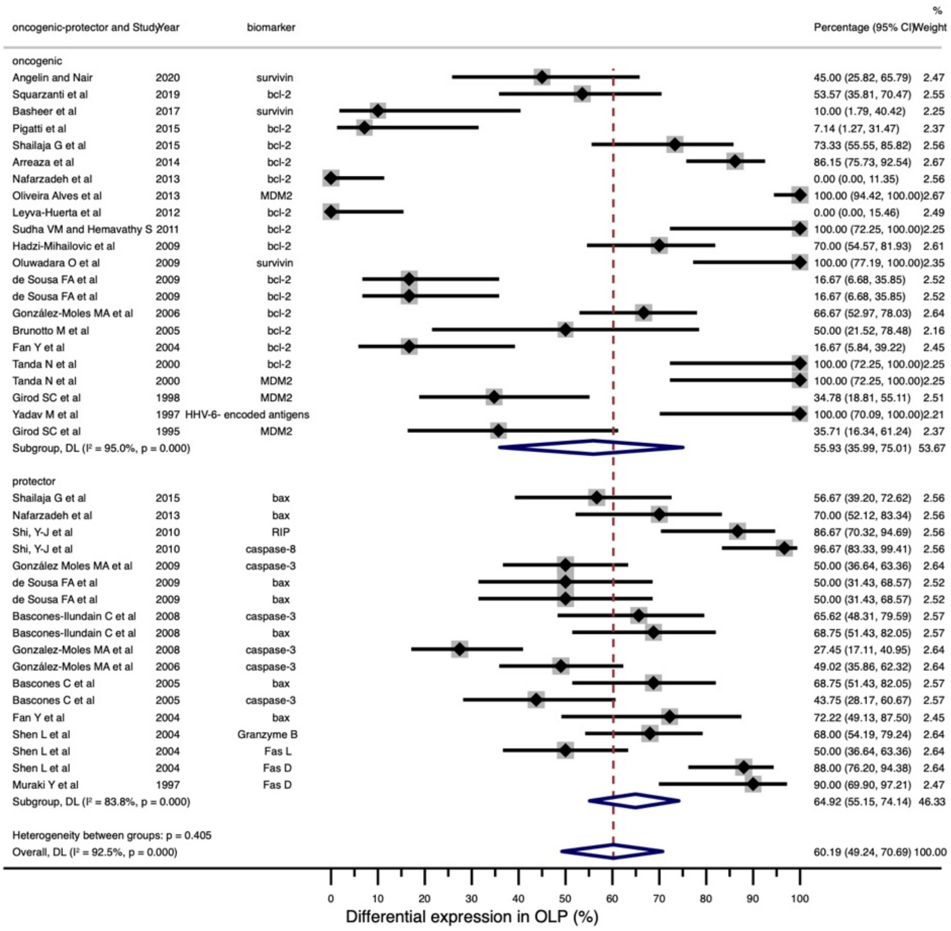

Figure S3. Forest plot graphically representing the differential expression of biomarkers on the hallmark of resisting cell death -using pooled proportions as ES metric, expressed as percentage- among OLP patients. ES, effect size; CI, confidence interval; Random-effects model.

3.4 Hallmark Enabling replicative immortality

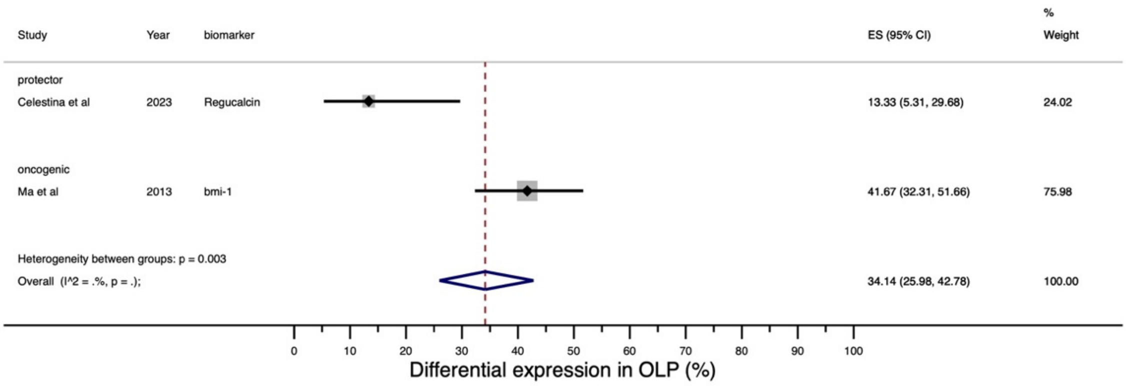

Figure S4. Forest plot graphically representing the differential expression of biomarkers on the hallmark of enabling replicative immortality -using pooled proportions as ES metric, expressed as percentage- among OLP patients. ES, effect size; CI, confidence interval; Random-effects model.

3.5 Hallmark Inducing angiogenesis

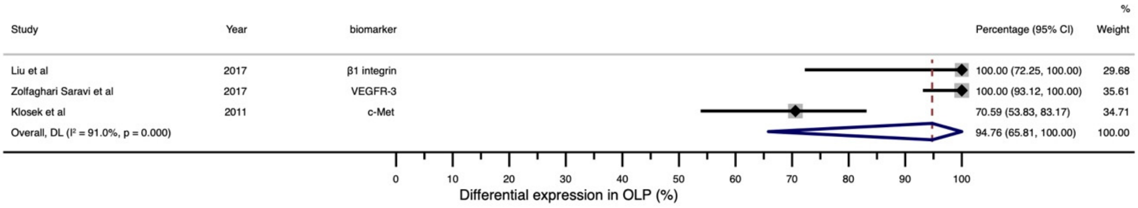

Figure S5. Forest plot graphically representing the differential expression of biomarkers on the hallmark of inducing angiogenesis -using pooled proportions as ES metric, expressed as percentage- among OLP patients. ES, effect size; CI, confidence interval; Random-effects model.

3.6 Hallmark Activating invasion and metastasis

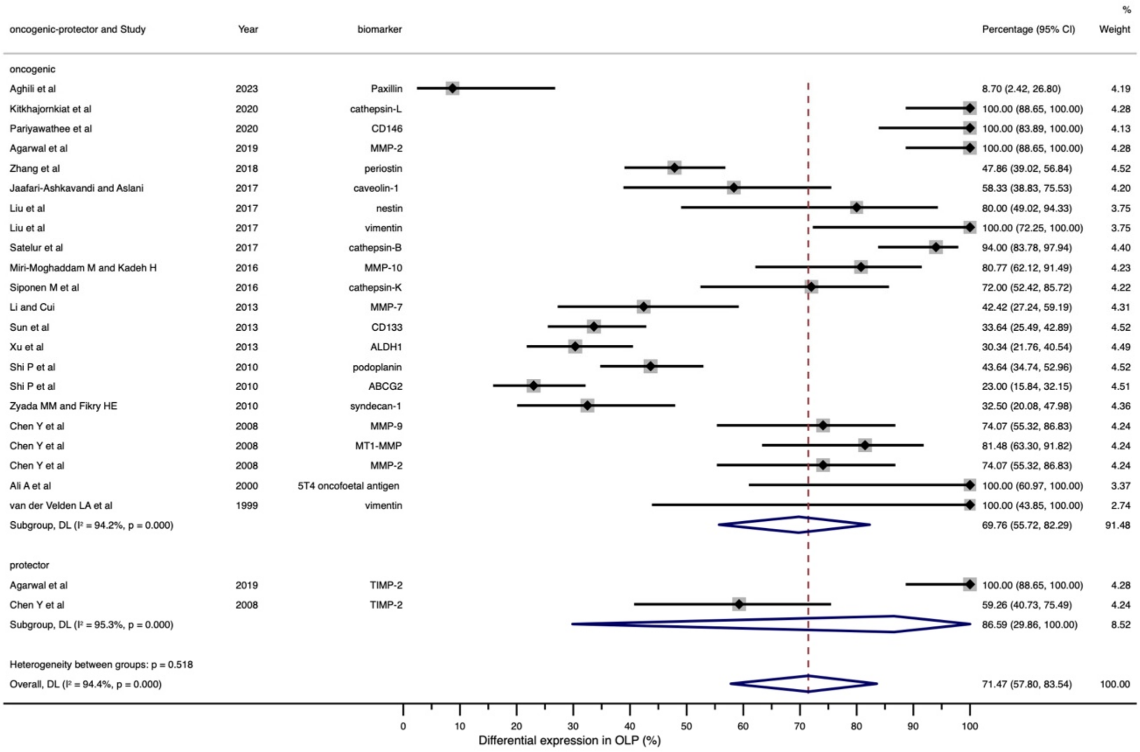

Figure S6. Forest plot graphically representing the differential expression of biomarkers on the hallmark of activating invasion and metastasis -using pooled proportions as ES metric, expressed as percentage- among OLP patients. ES, effect size; CI, confidence interval; Random-effects model.

3.7 Hallmark Avoiding immune destruction

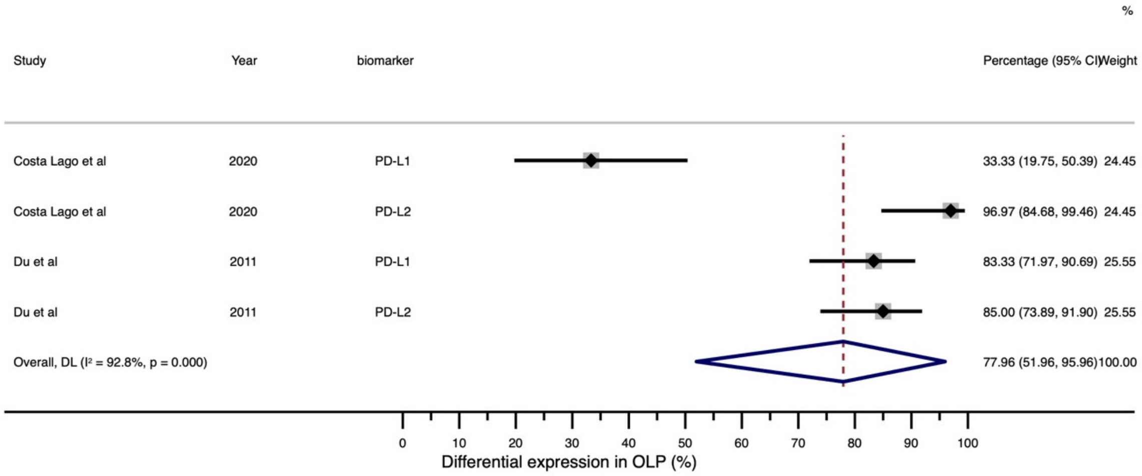

Figure S7. Forest plot graphically representing the differential expression of biomarkers on the hallmark of avoiding immune destruction -using pooled proportions as ES metric, expressed as percentage- among OLP patients. ES, effect size; CI, confidence interval; Random-effects model.

3.8 Hallmark Deregulating cellular energetics

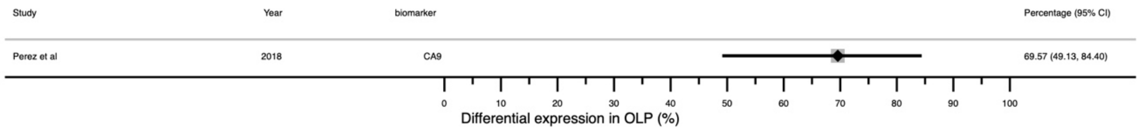

Figure S8. Forest plot graphically representing the differential expression of biomarkers on the hallmark of deregulating cellular energetics -using pooled proportions as ES metric, expressed as percentage- among OLP patients. ES, effect size; CI, confidence interval; Random-effects model. ES, effect size; CI, confidence interval; Random-effects model.

3.9 Hallmark Genome instability and mutation

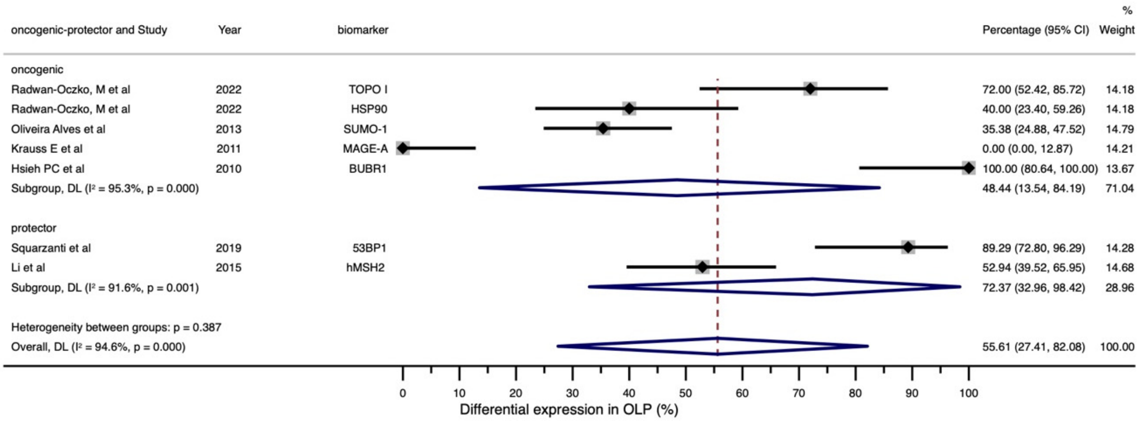

Figure S9. Forest plot graphically representing the differential expression of biomarkers on the hallmark of genome instability and mutation -using pooled proportions as ES metric, expressed as percentage- among OLP patients. ES, effect size; CI, confidence interval; Random-effects model.

3.10 Hallmark Tumor promoting and inflammation

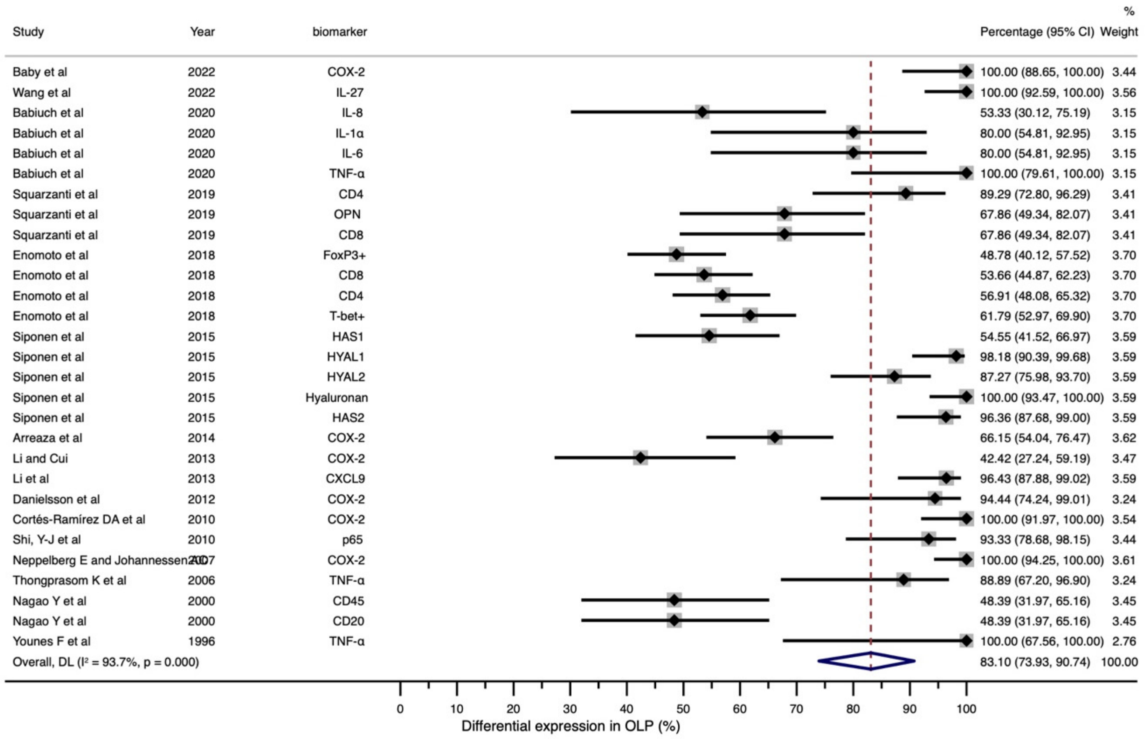

Figure S10. Forest plot graphically representing the differential expression of biomarkers on the hallmark of Tumor promoting and inflammation -using pooled proportions as ES metric, expressed as percentage- among OLP patients. ES, effect size; CI, confidence interval; Random-effects model.

4. Magnitude of association between oral cancer and OLP

4.1 Hallmark Sustaining proliferative signaling

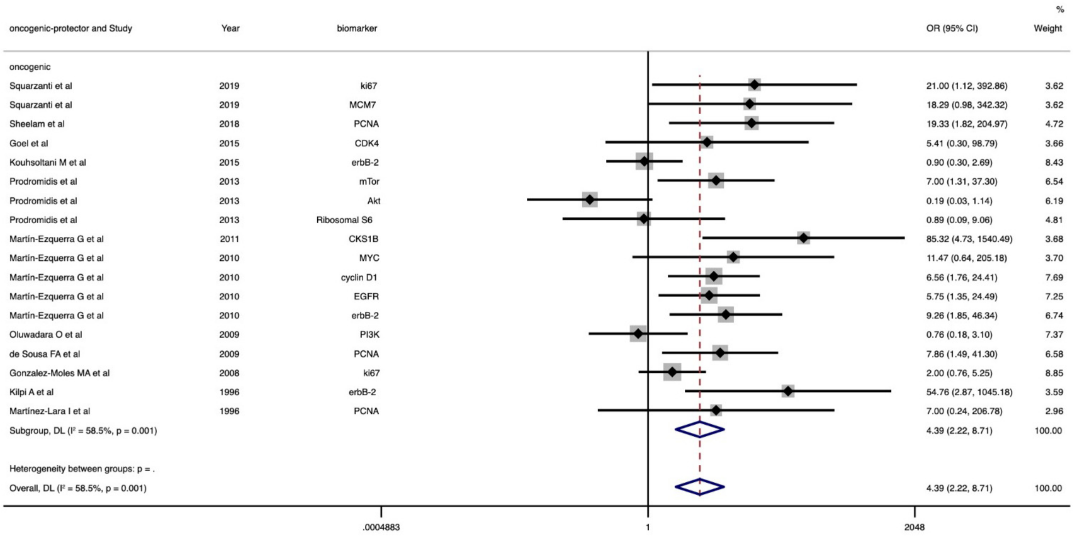

Figure S11. Forest plot graphically representing the meta-analysis on the magnitude of association of the hallmark of cancer sustaining proliferation -using OR as effect size metric- in order to compare the differential expression of biomarkers of the hallmark sustaining proliferative signaling between oral cancer and OLP. OR, odds ratio; CI, confidence interval; Random-effects model.

4.2 Hallmark Evading growth suppressors

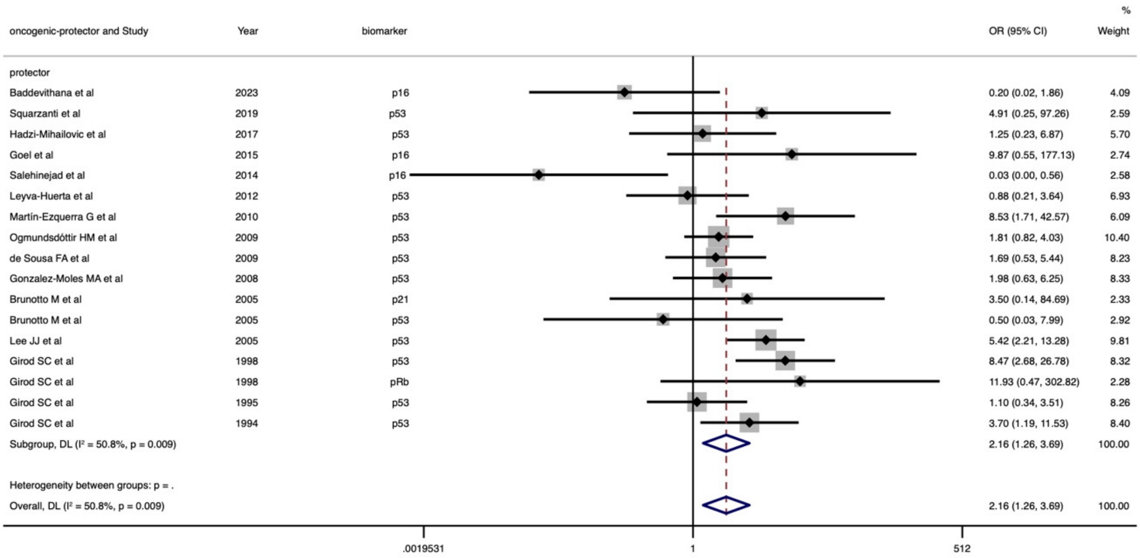

Figure S12. Forest plot graphically representing the meta-analysis on the magnitude of association -using OR as effect size metric- in order to compare the differential expression of biomarkers of the hallmark evading growth suppressors between oral cancer and OLP. OR, odds ratio; CI, confidence interval; Random-effects model.

4.3 Hallmark Resisting cell death

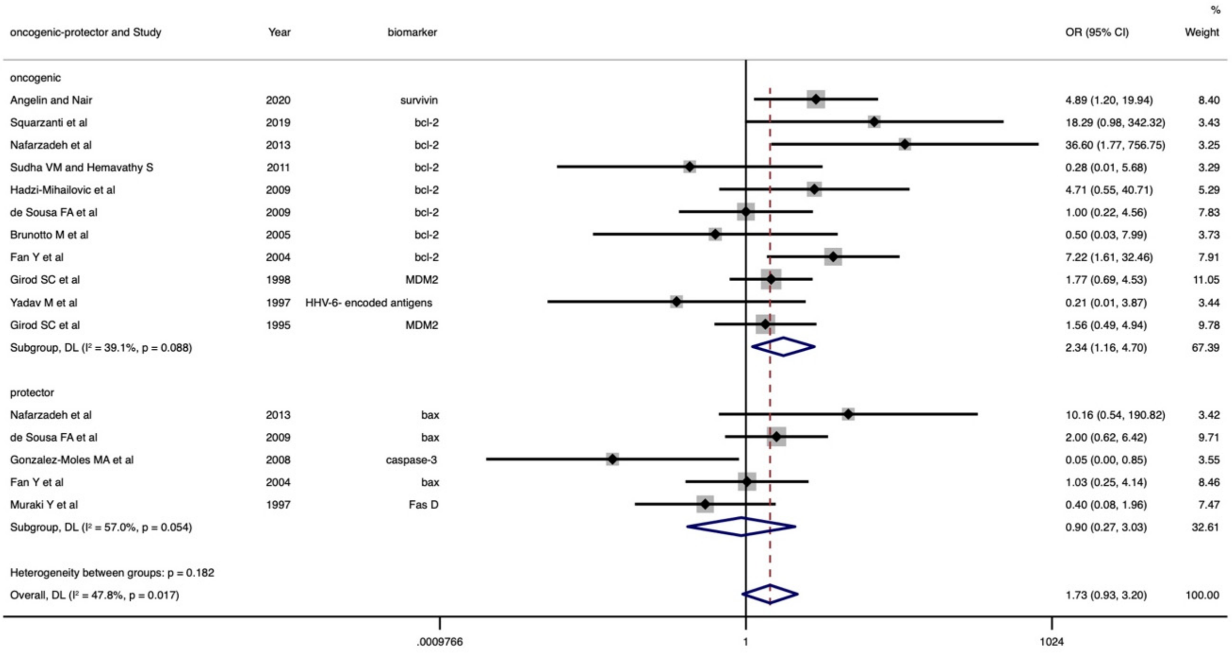

Figure S13. Forest plot graphically representing the meta-analysis on the magnitude of association -using OR as effect size metric- in order to compare the differential expression of biomarkers of the hallmark resisting cell death between oral cancer and OLP. OR, odds ratio; CI, confidence interval; Random-effects model.

4.4 Hallmark Enabling replicative immortality

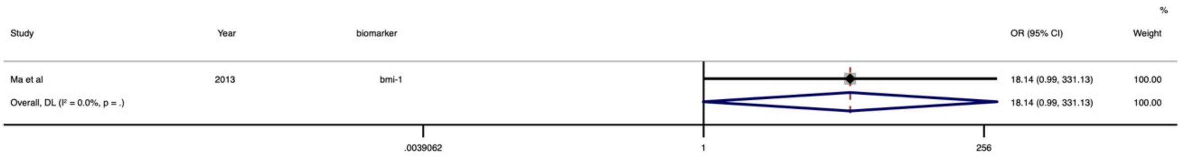

Figure S14. Forest plot graphically representing the meta-analysis on the magnitude of association -using OR as effect size metric- in order to compare the differential expression of biomarkers of the hallmark enabling replicative immortality between oral cancer and OLP. OR, odds ratio; CI, confidence interval; Random-effects model.

4.5 Hallmark Activating invasion and metastasis

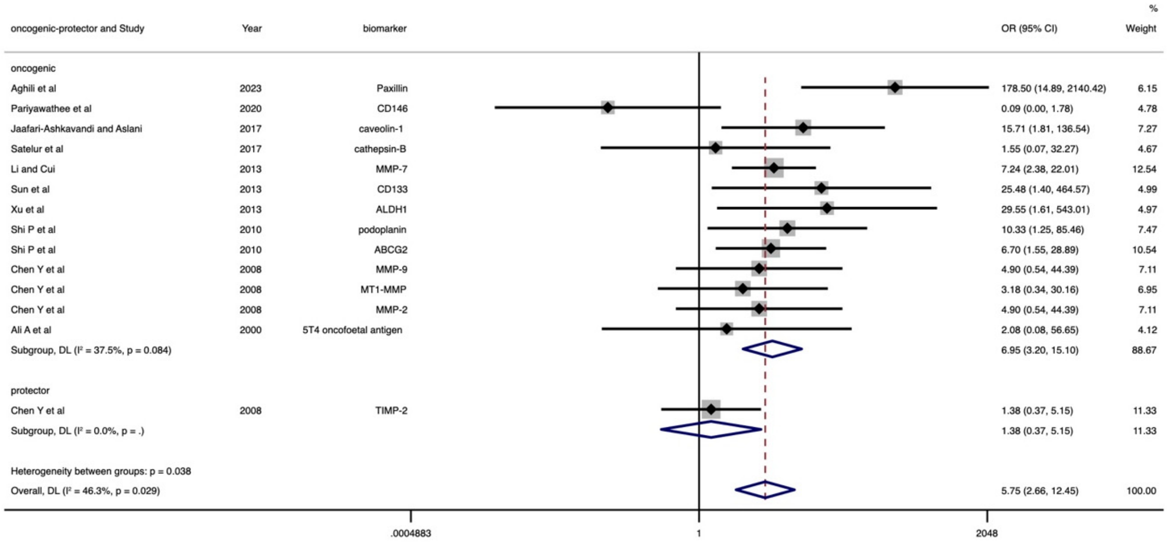

Figure S15. Forest plot graphically representing the meta-analysis on the magnitude of association -using OR as effect size metric- in order to compare the differential expression of biomarkers of the hallmark activating invasion and metastasis between oral cancer and OLP. OR, odds ratio; CI, confidence interval; Random-effects model.

4.6 Hallmark Genome instability and mutation

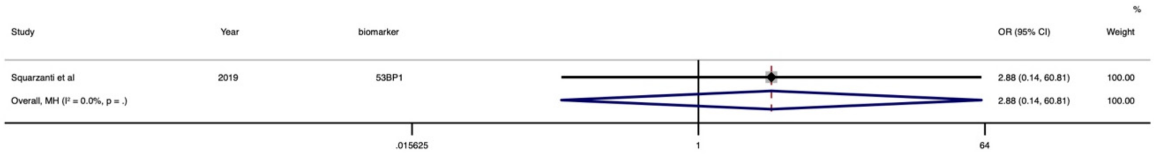

Figure S16. Forest plot graphically representing the meta-analysis on the magnitude of association -using OR as effect size metric- in order to compare the differential expression of biomarkers of the hallmark genome instability and mutation between oral cancer and OLP. OR, odds ratio; CI, confidence interval; Random-effects model.

4.7 Hallmark Tumor promoting and inflammation

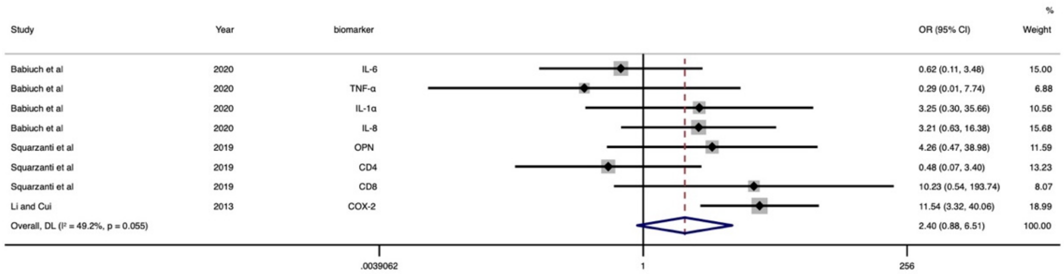

Figure S17. Forest plot graphically representing the meta-analysis on the magnitude of association -using OR as effect size metric- in order to compare the differential expression of biomarkers of the hallmark tumor promoting and inflammation between OLP and oral cancer. OR, odds ratio; CI, confidence interval; Random-effects model.

5. Magnitude of association between OLP and healthy controls.

5.1Hallmark Sustaining proliferative signaling

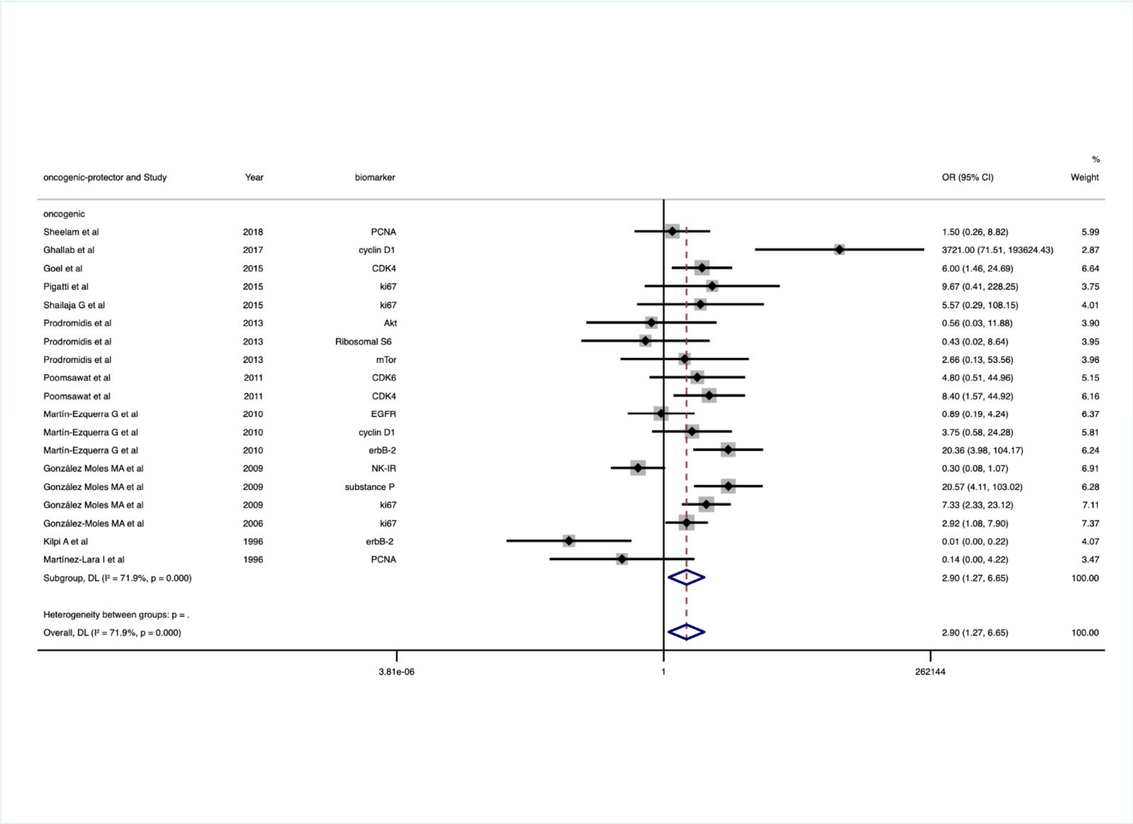

Figure S18. Forest plot graphically representing the meta-analysis of the magnitude of association -using OR as effect size metric- in order to compare the differential expression of biomarkers on the hallmark of cancer sustaining proliferation between OLP and healthy controls. OR, odds ratio; CI, confidence interval; Random-effects model.

5.2 Hallmark Evading growth suppressors

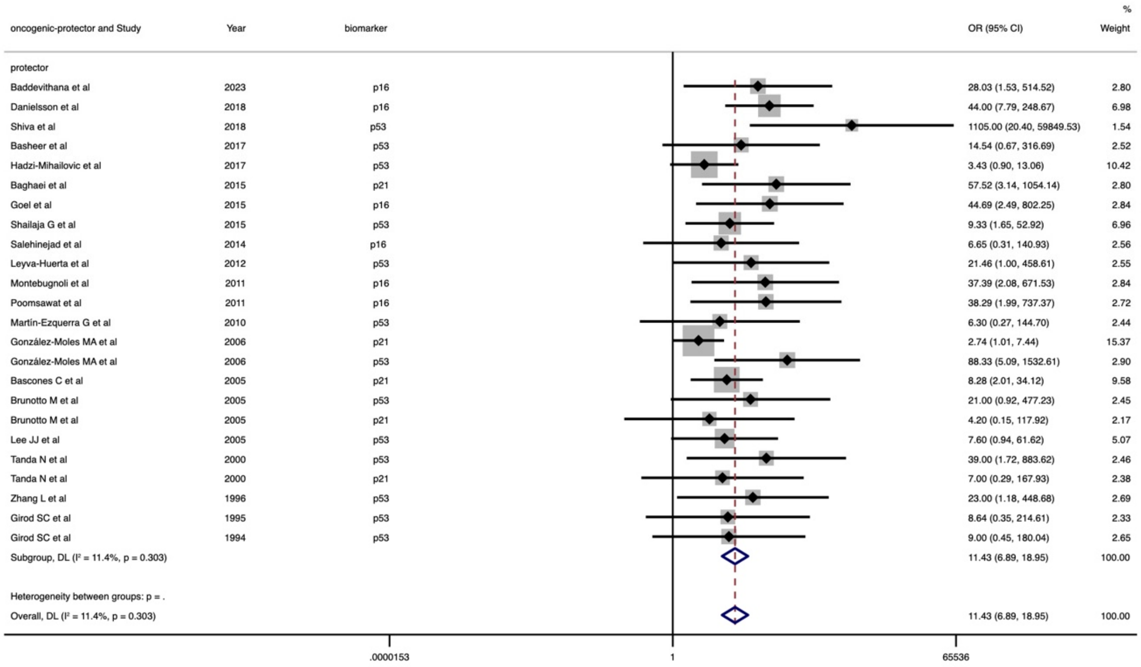

Figure S19. Forest plot graphically representing the meta-analysis of the magnitude of association -using OR as effect size metric- in order to compare the differential expression of biomarkers on the hallmark of evading growth suppressors between OLP and healthy controls. OR, odds ratio; CI, confidence interval; Random-effects model.

5.3 Hallmark Resisting cell death

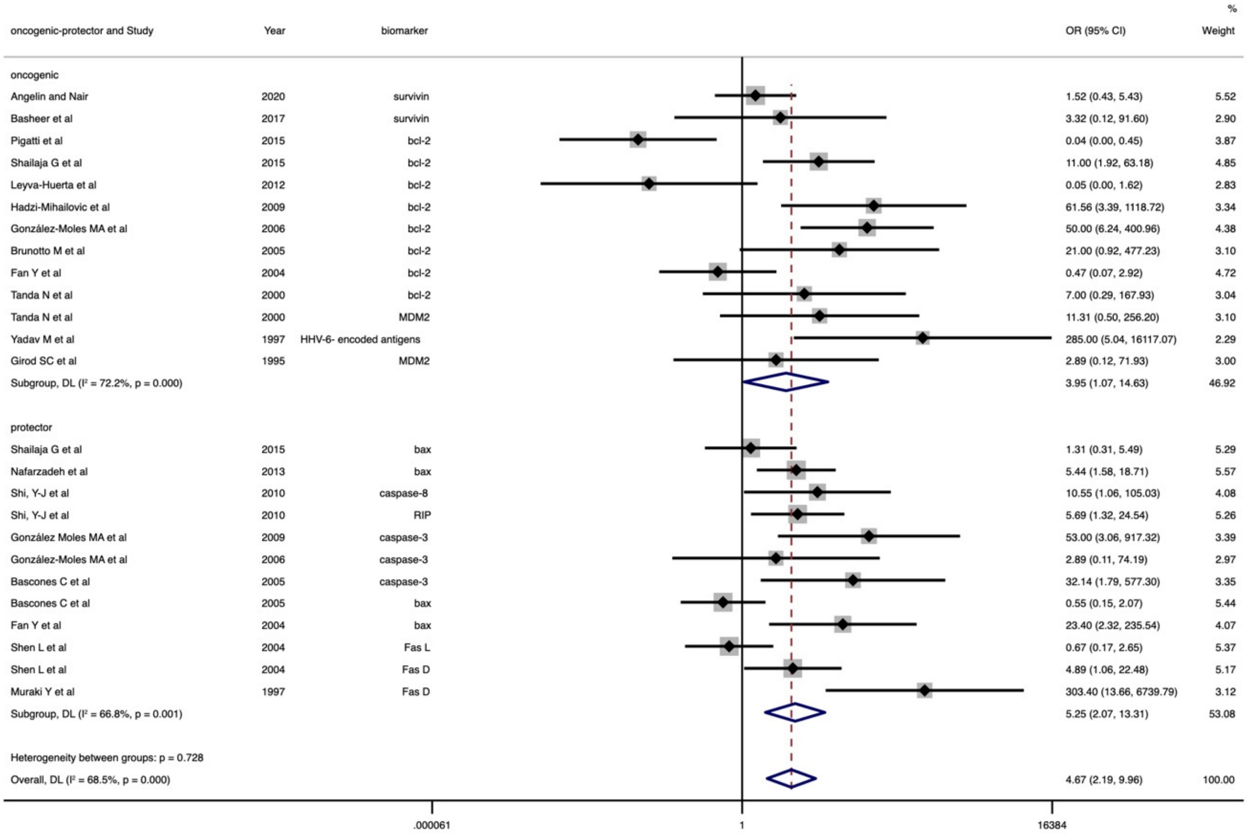

Figure S20. Forest plot graphically representing the meta-analysis of the magnitude of association -using OR as effect size metric- in order to compare the differential expression of biomarkers on the hallmark of resisting cell death between OLP and healthy controls. OR, odds ratio; CI, confidence interval; Random-effects model.

5.4 Hallmark Enabling replicative immortality

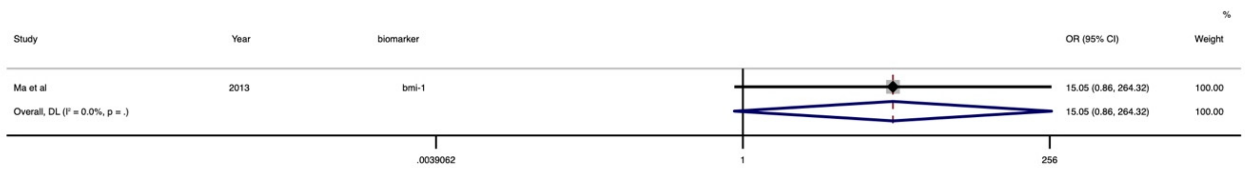

Figure S21. Forest plot graphically representing the meta-analysis of the magnitude of association -using OR as effect size metric- in order to compare the differential expression of biomarkers on the hallmark of enabling replicative immortality between OLP and healthy controls. OR, odds ratio; CI, confidence interval; Random-effects model.

5.5 Hallmark Inducing Angiogenesis

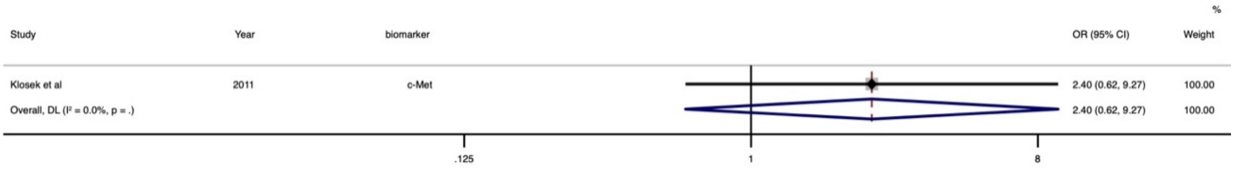

Figure S22. Forest plot graphically representing the meta-analysis of the magnitude of association -using OR as effect size metric- in order to compare the differential expression of biomarkers on the hallmark of inducing angiogenesis between OLP and healthy controls. OR, odds ratio; CI, confidence interval; Random-effects model.

5.6 Hallmark Activating invasion and metastasis

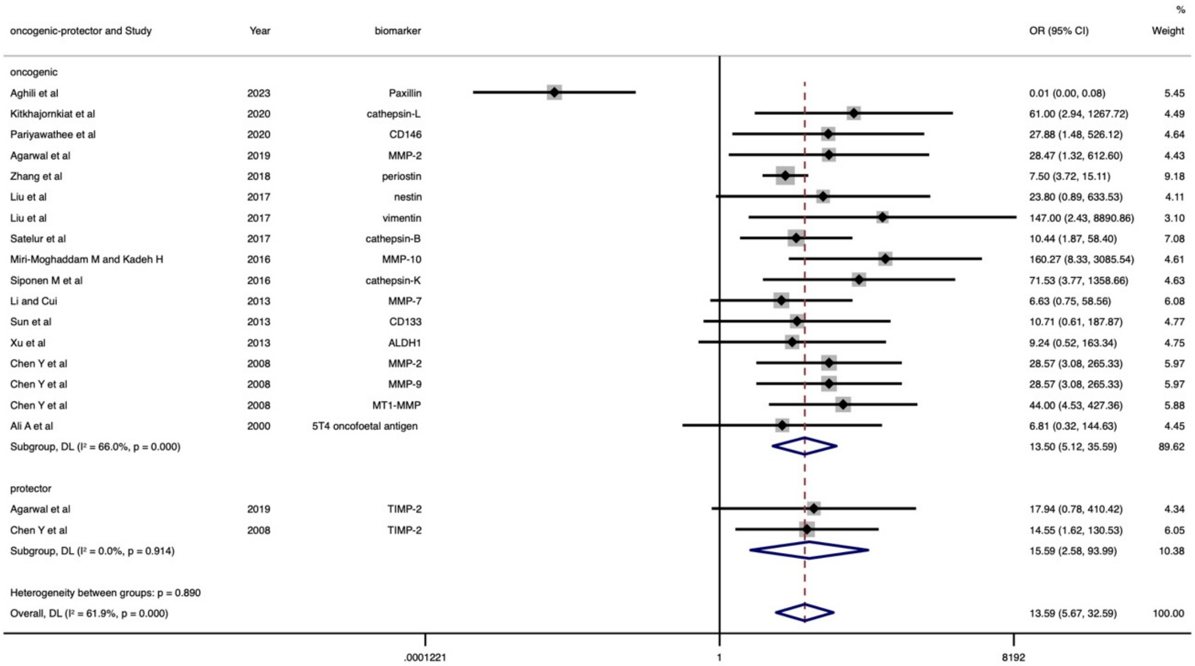

Figure S23. Forest plot graphically representing the meta-analysis of the magnitude of association -using OR as effect size metric- in order to compare the differential expression of biomarkers on the hallmark of activating invasion and metastasis between OLP and healthy controls. OR, odds ratio; CI, confidence interval; Random-effects model.

5.7 Hallmark Avoiding immune destruction

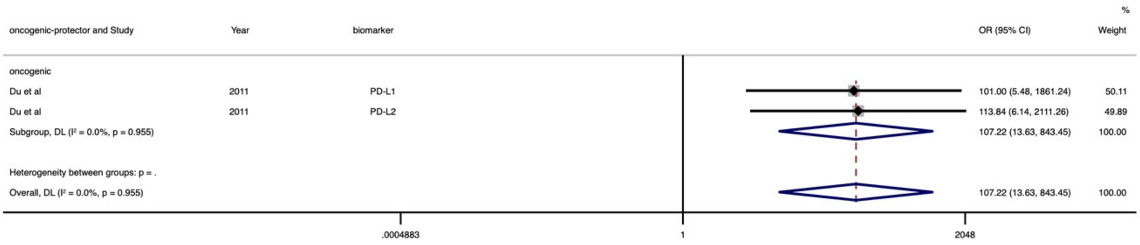

Figure S24. Forest plot graphically representing the meta-analysis of the magnitude of association -using OR as effect size metric- in order to compare the differential expression of biomarkers on the hallmark of avoiding immune destruction between OLP and healthy controls. OR, odds ratio; CI, confidence interval; Random-effects model.

5.8 Hallmark Deregulating cellular energetics

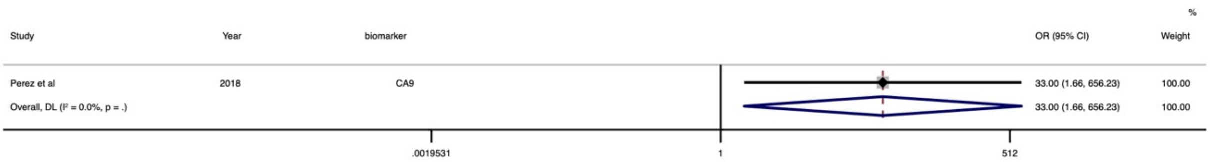

Figure S25. Forest plot graphically representing the meta-analysis of the magnitude of association -using OR as effect size metric- in order to compare the differential expression of biomarkers on the hallmark of deregulating cellular energetics between OLP and healthy controls. OR, odds ratio; CI, confidence interval; Random-effects model.

5.9 Hallmark Genome instability and mutation

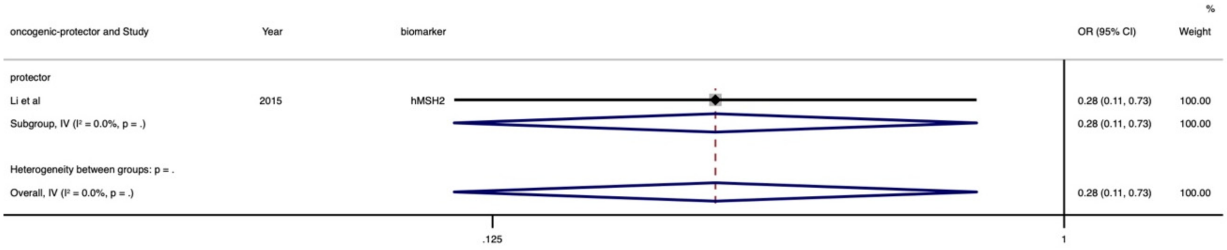

Figure S26. Forest plot graphically representing the meta-analysis of the magnitude of association -using OR as effect size metric- in order to compare the differential expression of biomarkers on the hallmark of genome instability and mutation between OLP and healthy controls. OR, odds ratio; CI, confidence interval; Random-effects model.

5.10 Hallmark Tumor promoting and inflammation

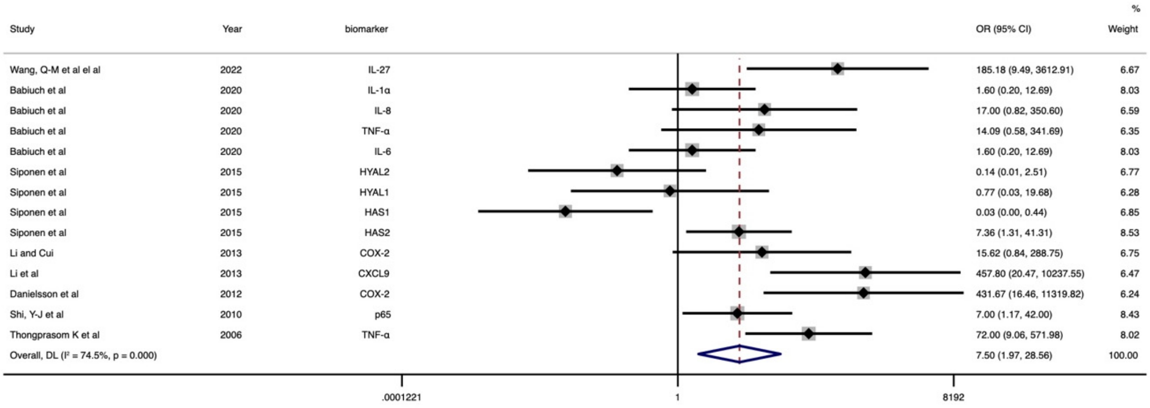

Figure S27. Forest plot graphically representing the meta-analysis of the magnitude of association -using OR as effect size metric- in order to compare the differential expression of biomarkers on the hallmark of tumor promoting and inflammation between OLP and healthy controls. OR, odds ratio; CI, confidence interval; Random-effects model.

6. Magnitude of association between oral cancer and healthy controls.

6.1 Hallmark Sustaining proliferative signaling

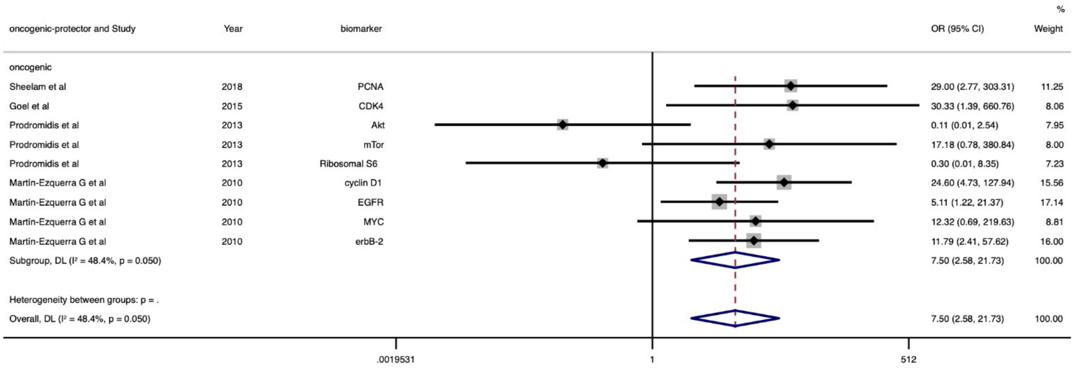

Figure S28. Forest plot graphically representing the meta-analysis of the magnitude of association -using OR as effect size metric- in order to compare the differential expression of biomarkers on the hallmark of cancer sustaining proliferation between oral cancer and healthy controls. OR, odds ratio; CI, confidence interval; Random-effects model.

6.2 Hallmark Evading growth suppressors

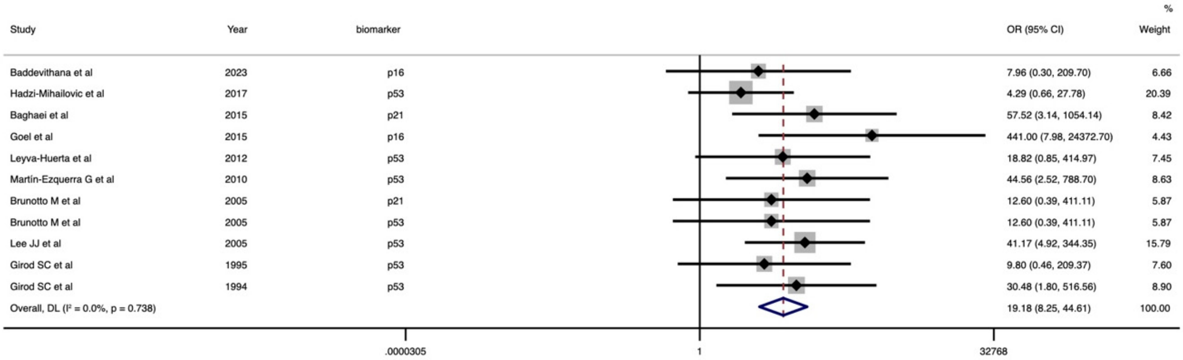

Figure S29. Forest plot graphically representing the meta-analysis of the magnitude of association -using OR as effect size metric- in order to compare the differential expression of biomarkers on the hallmark of evading growth suppressors between oral cancer and healthy controls. OR, odds ratio; CI, confidence interval; Random-effects model.

6.3 Hallmark Resisting cell death

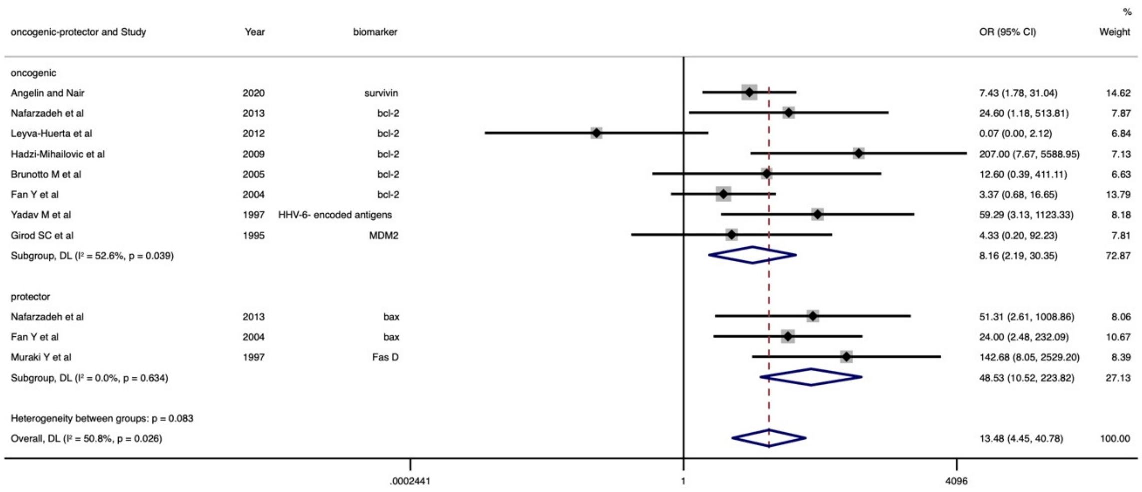

Figure S30. Forest plot graphically representing the meta-analysis of the magnitude of association -using OR as effect size metric- in order to compare the differential expression of biomarkers on the hallmark of resisting cell death between oral cancer and healthy controls. OR, odds ratio; CI, confidence interval; Random-effects model.

6.4 Hallmark Enabling replicative immortality

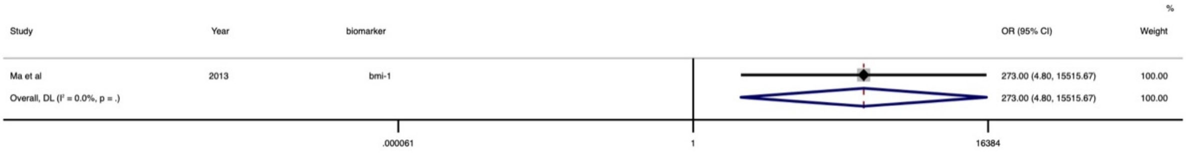

Figure S31. Forest plot graphically representing the meta-analysis of the magnitude of association -using OR as effect size metric- in order to compare the differential expression of biomarkers on the hallmark of enabling replicative immortality between oral cancer and healthy controls. OR, odds ratio; CI, confidence interval; Random-effects model.

6.5 Hallmark Activating invasion and metastasis

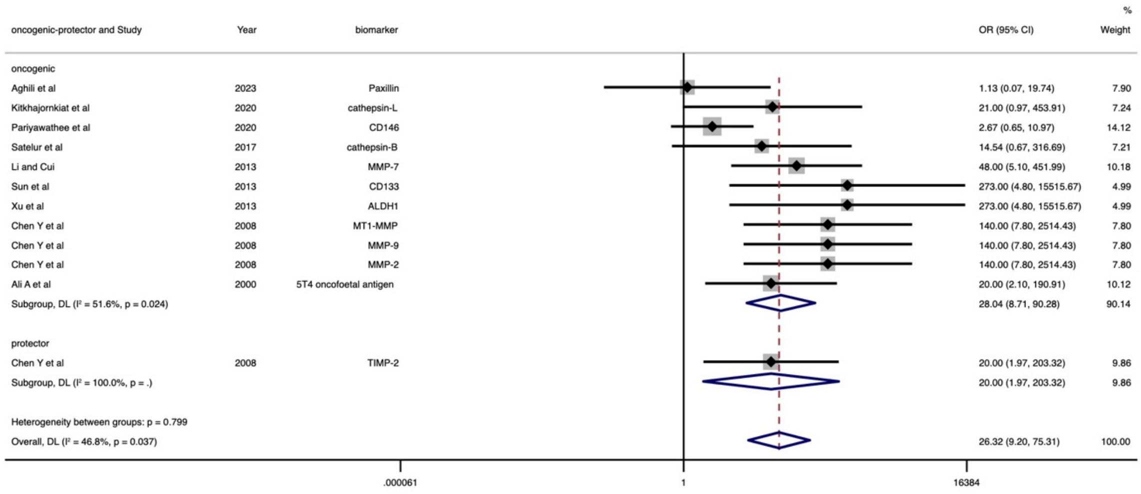

Figure S32. Forest plot graphically representing the meta-analysis of the magnitude of association -using OR as effect size metric- in order to compare the differential expression of biomarkers on the hallmark of activating invasion and metastasis between oral cancer and healthy controls. OR, odds ratio; CI, confidence interval; Random-effects model.

6.6 Hallmark Genome instability and mutation

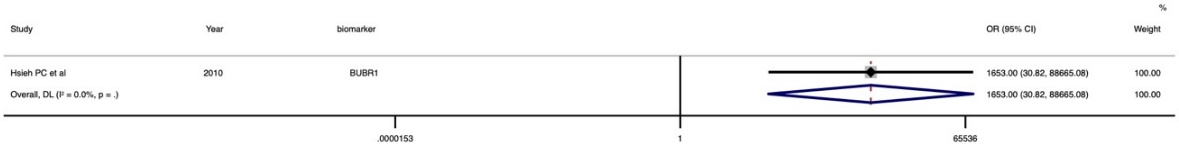

Figure S33. Forest plot graphically representing the meta-analysis of the magnitude of association -using OR as effect size metric- in order to compare the differential expression of biomarkers on the hallmark of genome instability and mutation between oral cancer and healthy controls. OR, odds ratio; CI, confidence interval; Random-effects model.

6.7 Hallmark Tumor promoting and inflammation

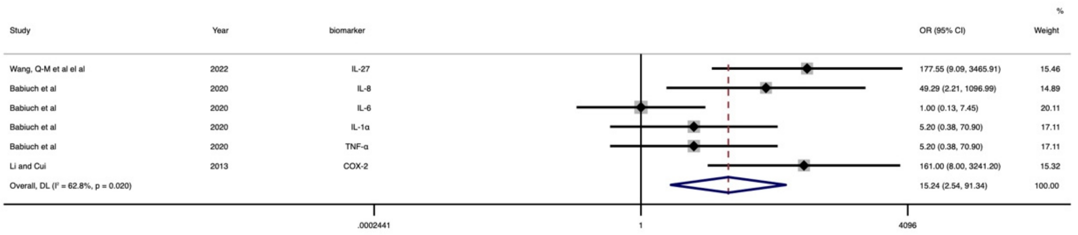

Figure S34. Forest plot graphically representing the meta-analysis of the magnitude of association -using OR as effect size metric- in order to compare the differential expression of biomarkers on the tumor promoting inflammation between oral cancer and healthy controls. OR, odds ratio; CI, confidence interval; Random-effects model.

7. Table S3. Biomarkers roles and hallmarks of cancer.

Biomarker	HM	oncogenic/protector	Function	HUGO	Additional source
Akt	1	proproliferative	**Cell proliferation activation.** It acts as a target and effector of the molecular signalling pathway phosphatidylinositol3-kinase (PI3K), an important pathway that stimulates cell proliferation.	✓	Prodromidis, Georgios, Nikolaos G. Nikitakis, and Alexandra Sklavounou. 2013. "Immunohistochemical Analysis of the Activation Status of the Akt/MTOR/PS6 Signaling Pathway in Oral Lichen Planus." *International Journal of Dentistry* 2013:743456. doi: 10.1155/2013/743456.
CDK4	1	proproliferative	**Cell cycle progression.** It activates progression from G1 to S phase of the cell cycle after forming complexes with Cyclin D1. Cyclin D1-CDK4 complexes phosphorylate retinoblastoma (pRb) proteins, leading to dissociation of the pRb-E2F complex and progression from G1 to S phase.	✓	Goel, Sinny, Nita Khurana, Akanksha Marvah, and Sunita Gupta. 2015. "Expression of Cdk4 and P16 in Oral Lichen Planus." *Journal of Oral and Maxillofacial Research* 6(2). doi: 10.5037/jomr.2015.6204.
CDK6	1	proproliferative	**Cell cycle progression.** It activates progression from G1 to S phase of the cell cycle after forming complexes with cyclin D1. Cyclin D1-CDK6 complexes phosphorylate retinoblastoma (pRb) proteins, leading to the dissociation of the pRb-E2F complex and progression from G1 to S phase.	✓	Poomsawat, Sopee, Waranun Buajeeb, Siribang-on Piboonniyom Khovidhunkit, and Jirapa Punyasingh. 2011. "Overexpression of Cdk4 and P16 in Oral Lichen Planus Supports the Concept of Premalignancy." *Journal of Oral Pathology & Medicine* 40(4):294–99. doi: 10.1111/j.1600-0714.2010.01001.x.
CKS1B	1	proproliferative	**Cell cycle progression** It associates with and activates cyclin-dependent kinase CDC28, and behaves as a promoter regulator of cell cycle progression from G1 to S phase.	✓	Martín-Ezquerra G, Salgado R, Toll A, Baró T, Mojal S, Yébenes M, Garcia-Muret MP, Solé F, Quitllet FA, Espinet B, Pujol RM. CDC28 protein kinase regulatory subunit 1B (CKS1B) expression and genetic status analysis in oral squamous cell carcinoma. Histol Histopathol. 2011 Jan;26(1):71-7. doi: 10.14670/HH-26.71. PMID: 21117028.
Cyclin D1	1	proproliferative	**Cell cycle progression** Activates progression from G1 to S phase of the cell cycle after forming complexes with CDKs4/6. Cyclin D1-CDK4/6 complexes phosphorylate retinoblastoma (pRb) proteins, leading to dissociation of the pRb-E2F complex and progression from G1 to S phase.	✓	Ghallab, Noha A., Rehab Fawzy Kasem, Safa Fathy Abd El-Ghani, and Olfat G. Shaker. 2017. "Gene Expression of MiRNA-138 and Cyclin D1 in Oral Lichen Planus." *Clinical Oral Investigations* 21(8):2481–91. doi: 10.1007/s00784-017-2091-5.
EGFR	1	proproliferative	**Cell cycle progression** Transmembrane glycoprotein with tyrosine kinase activity. Binding of its extracellular domains to their respective ligands (e.g., EGF) activates multiple molecular signalling pathways that stimulate cell proliferation (e.g., MAPK pathway).	✓	Cortes-Ramirez, D. A., M. J. Rodriguez-Tojo, J. C. Coca-Meneses, X. Marichalar-Mendia, and J. M. Aguirre-Urizar. 2014. "Epidermal Growth Factor Receptor Expression in Different Subtypes of Oral Lichenoid Disease." *Medicina Oral Patología Oral y Cirugia Bucal* e451--e458. doi: 10.4317/medoral.19452.

erbB-2	1	proproliferative	**Cell cycle progression**<br>ErbB2 is a receptor with tyrosine kinase activity, from the EGFR family (i.e., ErbB/HER family). Although it has no known ligand, its configuration remains activated, triggering multiple molecular signalling pathways that stimulate cell proliferation (e.g., MAPK pathway).	✓	Kouhsoltani, Maryam, Amirala Aghbali, Behrooz Shokoohi, and Ronak Ahmadzadeh. 2015. “Molecular Targeting of Her-2/Neu Protein Is Not Recommended as an Adjuvant Therapy in Oral Squamous Cell Carcinoma and Oral Lichen Planus.” *Advanced Pharmaceutical Bulletin* 5(Suppl 1):649–52. doi: 10.15171/apb.2015.088.
ki67	1	proproliferative	**Indicator of the total fraction of proliferating cells**<br>Well-known marker of proliferative activity in epithelial cells. It can be detected in the G1, S, G2 and M phases of the cell cycle, but not in the G0 phase.	✓	González-Moles, M. A., C. Bascones-Ilundain, J. A. Gil Montoya, I. Ruiz-Avila, M. Delgado-Rodríguez, and A. Bascones-Martínez. 2006. “Cell Cycle Regulating Mechanisms in Oral Lichen Planus: Molecular Bases in Epithelium Predisposed to Malignant Transformation.” *Archives of Oral Biology* 51(12):1093–1103. doi: 10.1016/j.archoralbio.2006.06.007.
LCK	1	proproliferative	**STAT signalling activator**<br>Activator of the STAT protein, a well-known transcription factor of the JAK-STAT signalling pathway that translocates to the cell nucleus to activate the expression of genes involved in cell proliferation.	✓	Oluwadara, Oluwadayo, Luca Giacomelli, Russell Christensen, George Kossan, Raisa Avezova, and Francesco Chiappelli. 2009. “LCK, Survivin and PI-3K in the Molecular Biomarker Profiling of Oral Lichen Planus and Oral Squamous Cell Carcinoma.” *Bioinformation* 4(6):249–57. doi: 10.6026/97320630004248.
MCM7	1	proproliferative	**Iniciation of eukaryotic genome replication**<br>Cell cycle regulator of late G1 phase and early S phase who orchestrates the initiation of DNA replication and consequently cell proliferation through cell cycle progression.	✓	Squarzanti, Diletta Francesca, Tiziana Cena, Rita Sorrentino, Mario Migliario, Annalisa Chiocchetti, Lia Rimondini, Barbara Azzimonti, and Guido Valente. 2019. “Implications on Pathogenesis and Risk of Oral Lichen Planus Neoplastic Transformation: An Ex-Vivo Retrospective Immunohistochemical Study.” *Histology and Histopathology* 34(9):1015–24. doi: 10.14670/HH-18-104.
mTOR	1	proproliferative	**Cell proliferation activation**<br>Popular member of the PI3K molecular signalling pathway, which, when activated by Akt, controls cell proliferation processes, such as translation of pro-proliferative protein mRNA (e.g. cyclin D1) to ribosomes for further synthesis.	✓	Prodromidis, Georgios, Nikolaos G. Nikitakis, and Alexandra Sklavounou. 2013. “Immunohistochemical Analysis of the Activation Status of the Akt/MTOR/PS6 Signaling Pathway in Oral Lichen Planus.” *International Journal of Dentistry* 2013:743456. doi: 10.1155/2013/743456.
MYC	1	proproliferative	**Activation of cell cycle progression**<br>Renowned transcription factor that binds to DNA and facilitates the transcription of mitogenic signal-mediated genes involved in cell proliferation processes.	✓	Martín-Ezquerra, G., R. Salgado, A. Toll, M. Gilaberte, T. Baró, F. Alameda Quitllet, M. Yébenes, F. Solé, M. Garcia-Muret, B. Espinet, and R. M. Pujol. 2010. “Multiple Genetic Copy Number Alterations in Oral Squamous Cell Carcinoma: Study of MYC , TP53 , CCDN1, EGFR and ERBB2 Status in Primary and Metastatic Tumours.” *British Journal of Dermatology* 163(5):1028–35. doi: 10.1111/j.1365-2133.2010.09947.x.
NK-IR	1	proproliferative	**Cell proliferation and tumour growth**<br>The NK-1R receptor, upon binding to the neuropeptide substance P, activates members of the mitogen-activated protein kinase (MAPK) cascade, including extracellular signal-	✓	González Moles, M. A., F. Esteban, I. Ruiz-Ávila, J. A. Gil Montoya, S. Brener, A. Bascones-Martínez, and M. Muñoz. 2009. “A Role for the Substance P/NK-1 Receptor Complex in Cell Proliferation and Apoptosis in Oral Lichen Planus.” *Oral Diseases* 15(2):162–69. doi: 10.1111/j.1601-0825.2008.01504.x.

			regulated kinases 1 and 2 (ERK 1 ⁄ 2), which move to the nucleus to induce cell proliferation.		
p38MAPK	1	proproliferative	**Cell proliferation and tumour growth** Member of the MAPK molecular signalling pathway that regulates cell proliferation through the progression of cell cycle phases G1/S and G2/M.	✓	Hu, X. S., Y. H. Huang, X. S. Liu, and H. Hua. 2016. “[Expression and Significance of P38 Mitogen-Activated Protein Kinase in Oral Lichen Planus and Oral Squamous Cell Cacinoma].” *Beijing Da Xue Xue Bao. Yi Xue Ban = Journal of Peking University. Health Sciences* 48(2):310–15.
PCNA	1	proproliferative	**Indicator of cell cycle progression** The PCNA (proliferating cell nuclear antigen) protein is used as a proliferation marker. It is located in the cell nucleus and promotes DNA synthesis, which is why it is most marked in the G1 and S phases of the cell cycle.	✓	Martínez-Lara, I., M. A. González-Moles, I. Ruiz-Avila, M. Bravo, M. C. Ramos, and J. A. Fernández-Martínez. 1996. “Proliferating Cell Nuclear Antigen (PCNA) as a Marker of Dysplasia in Oral Mucosa.” *Acta Stomatologica Belgica* 93(1):29–32.
PI3K	1	proproliferative	**Cell proliferation activation** Protein phosphoinositol 3-kinase or PI3K is a family of enzymes that regulate the important pathway PI3K-Akt, which stimulates cell proliferation.	✓	Oluwadara, Oluwadayo, Luca Giacomelli, Russell Christensen, George Kossan, Raisa Avezova, and Francesco Chiappelli. 2009. “LCK, Survivin and PI-3K in the Molecular Biomarker Profiling of Oral Lichen Planus and Oral Squamous Cell Carcinoma.” *Bioinformation* 4(6):249–57. doi: 10.6026/97320630004248.
ribosomal S6	1	proproliferative	**Cell proliferation activation** Ribosomal protein S6 (pS6) is a target upon mTor activation and is used as a reliable surrogate marker of mTor activity.	✓	Prodromidis, Georgios, Nikolaos G. Nikitakis, and Alexandra Sklavounou. 2013. “Immunohistochemical Analysis of the Activation Status of the Akt/MTOR/PS6 Signaling Pathway in Oral Lichen Planus.” *International Journal of Dentistry* 2013:743456. doi: 10.1155/2013/743456.
substance P	1	Pro-proliferative	**Cell proliferation activation** Member of the tachykinin family of neuropeptides. It exerts its action by binding to the NK-1R receptor which activates members of the mitogen-activated protein kinase (MAPK) cascade, including extracellular signal-regulated kinases 1 and 2 (ERK 1 ⁄ 2), which move into the nucleus to induce cell proliferation.	✓	González Moles, M. A., F. Esteban, I. Ruiz-Ávila, J. A. Gil Montoya, S. Brener, A. Bascones-Martínez, and M. Muñoz. 2009. “A Role for the Substance P/NK-1 Receptor Complex in Cell Proliferation and Apoptosis in Oral Lichen Planus.” *Oral Diseases* 15(2):162–69. doi: 10.1111/j.1601-0825.2008.01504.x.
p16	2	protector	**Cell cycle control** It is a negative regulator of cell proliferation by inhibiting the formation of cyclin D1-CDK4/6 complexes, thereby blocking cell cycle progression.	✓	Montebugnoli, L., M. Venturi, D. B. Gissi, E. Leonardi, A. Farnedi, and Maria P. Foschini. 2011. “Immunohistochemical Expression of P16INK4A Protein in Oral Lichen Planus.” *Oral Surgery, Oral Medicine, Oral Pathology, Oral Radiology, and Endodontology* 112(2):222–27. doi: 10.1016/j.tripleo.2011.02.029.
p21	2	protector	**Cell cycle control** It is a negative regulator of cell proliferation by inhibiting the formation of cyclin E-CDK2 and cyclin D1-CDK4/6 complexes, thereby blocking cell cycle progression.	✓	Brunotto, Mabel, Ana María Zárate, Adriana Cismondi, María del Carmen Fernández, and Rita Inés Noher de Halac. 2005. “Valuation of Exfoliative Cytology as Prediction Factor in Oral Mucosa Lesions.” *Medicina Oral, Patologia Oral y Cirugia Bucal* 10 Suppl 2:E92--102.

p53	2	protector	**Cell cycle control, DNA repair and apoptosis**<br>It is a key tumour suppressor, known as the "guardian of the genome", stops the cell cycle and inhibits the proliferation of cells with damaged DNA, DNA repair and promotes apoptosis in cases of irreversible damage.	✓	Brunotto, Mabel, Ana María Zárate, Adriana Cismondi, María del Carmen Fernández, and Rita Inés Noher de Halac. 2005. "Valuation of Exfoliative Cytology as Prediction Factor in Oral Mucosa Lesions." *Medicina Oral, Patologia Oral y Cirugia Bucal* 10 Suppl 2:E92--102.
pRb	2	protector	**Tumoral supression**<br>It is a key tumour suppressor, inhibits cell proliferation by arresting the cell cycle in G1, essentially by sequestering E2F transcription factors, preventing activation of target genes involved in proliferation.	✓	Girod, S. C., P. Pfeiffer, J. Ries, and H. D. Pape. 1998. "Proliferative Activity and Loss of Function of Tumour Suppressor Genes as 'biomarkers' in Diagnosis and Prognosis of Benign and Preneoplastic Oral Lesions and Oral Squamous Cell Carcinoma." *The British Journal of Oral & Maxillofacial Surgery* 36(4):252–60. doi: 10.1016/s0266-4356(98)90708-2.
bax	3	Protector (pro-apoptotic)	**Pro-apoptotic**<br>It is an activator of apoptosis. It interacts with the mitochondrial voltage dependent anionic channel increasing its aperture leading to the permeabilization of the outer mitochondrial membrane hence to the lost of the membrane potential and the release of cytochrome C and other mitochondrial proapoptotic factors.	✓	Kvansakul M, Caria S, Hinds MG. The Bcl-2 Family in Host-Virus Interactions. Viruses. 2017 Oct 6;9(10):290. doi: 10.3390/v9100290. PMID: 28984827; PMCID: PMC5691641.
bcl-2	3	Oncogenic (anti-apoptotic)	**Anti-apoptotic**<br>It is the main molecule that actively<br><br>suppresses apoptosis throughout the intrinsic apoptosis pathway preventing the release of cytochrome c by means of inhibiting Bax insertion in the mitochondria or by inhibiting directly or indirectly Bax-channel activity.	✓	Kvansakul M, Caria S, Hinds MG. The Bcl-2 Family in Host-Virus Interactions. Viruses. 2017 Oct 6;9(10):290. doi: 10.3390/v9100290. PMID: 28984827; PMCID: PMC5691641.
caspase-3	3	Protector (pro-apoptotic)	**Pro-apoptotic**<br>It is processed and activated by caspases 8. It is essential for apoptotic chromatin condensation and DNA fragmentation.	✓	Porter, A. G., & Jänicke, R. U. (1999). Emerging roles of caspase-3 in apoptosis. *Cell death & differentiation*, *6*(2), 99-104.
caspase-8	3	Protector (pro-apoptotic)	**Pro-apoptotic**<br>It is involved in the initiation of extrinsic apoptosis execution. Being activated by surface cell death receptor signals, it leads to a series of BID protein modifications that will serve as a ligand to promote Bax activation.	✓	Tummers, B., & Green, D. R. (2017). Caspase-8: regulating life and death. *Immunological reviews*, *277*(1), 76-89.
Fas L	3	Protector (pro-apoptotic)	**Pro-apoptotic**	✓	Lord, S. J., Rajotte, R. V., Korbutt, G. S., & Bleackley, R. C. (2003). Granzyme B: a natural born killer. *Immunological reviews*, *193*(1), 31-38.

			It approaches the mechanism of Cytotoxic T lymphocytes-mediated target cell lysis by receptor. On activated CTLs, it engages the Fas receptor on target cells, causing them to undergo apoptotic cell death.		
Granzyme B	3	Protector (pro-apoptotic)	**Pro-apoptotic**<br>It is a cytolytic effector molecule delivered in the major mechanism for CTL-mediated destruction of target cells which allows a rapid induction of DNA fragmentation.	✓	Lord, S. J., Rajotte, R. V., Korbutt, G. S., & Bleackley, R. C. (2003). Granzyme B: a natural born killer. *Immunological reviews*, *193*(1), 31-38.
HHV-6- encoded antigens	3	Oncogenic (anti-apoptotic)	**Anti-apoptotic**<br>It is a ubiquitous virus capable of preventing apoptosis of T-lymphocytes by inhibiting p53 nuclear localization as an indirect mechanism which limits its ability to promote apoptosis.	✓	Eliassen, E., Lum, E., Pritchett, J., Ongradi, J., Krueger, G., Crawford, J. R., ... & Hudnall, S. D. (2018). Human herpesvirus 6 and malignancy: a review. *Frontiers in oncology*, *8*, 512.
MDM2	3	Oncogenic (anti-apoptotic)	**Anti-apoptotic**<br>This oncoprotein plays a central role in the regulatory process of p53. MDM2 protein binds to p53 and blocks its activity as a tumour suppressor and promotes its degradation. As MDM2 levels increase it results in the inhibition of p53 transcriptional activity and the degradation of p53 protein avoiding its activation as a transcription factor so the start of cell-cycle arrest or apoptotic functions do not succeed.	✓	Freedman, D. A., Wu, L., & Levine, A. J. (1999). Functions of the MDM2 oncoprotein. *Cellular and Molecular Life Sciences CMLS*, *55*, 96-107.
RIP	3	Protector (pro-apoptotic)	**Pro-apoptotic**<br>It has the capability to activate the transcription factor NFκB and induce apoptosis. It has also been reported to enhance caspases activity.	✓	Yu, P. W., Huang, B. C., Shen, M., Quast, J., Chan, E., Xu, X., ... & Luo, Y. (1999). Identification of RIP3, a RIP-like kinase that activates apoptosis and NFκB. *Current biology*, *9*(10), 539-542.
survivin	3	Oncogenic (anti-apoptotic)	**Anti-apoptotic**<br>A critical cancer-specific protein whose expression in tissues stimulates T cells. It belongs to the family of apoptosis inhibitors and is currently a key molecular target in cancer therapy..	✓	Oluwadara, Oluwadayo, Luca Giacomelli, Russell Christensen, George Kossan, Raisa Avezova, and Francesco Chiappelli. 2009. “LCK, Survivin and PI-3K in the Molecular Biomarker Profiling of Oral Lichen Planus and Oral Squamous Cell Carcinoma.” *Bioinformation* 4(6):249–57. doi: 10.6026/97320630004248.
Bmi-1	4	Oncogenic	**Cell inmortality**<br>Member of the complex PCR1 (Polycomb repressive complex 1). It is implied in the gene silencing by regulating chromatin structure and it is essential for the maintenance and	✓	Liu J, Cao L, Chen J, Song S, Lee IH, Quijano C, Liu H, Keyvanfar K, Chen H, Cao LY, Ahn BH, Kumar NG, Rovira II, Xu XL, van Lohuizen M, Motoyama N, Deng CX, Finkel T. Bmi1 regulates mitochondrial function and the DNA damage response pathway. Nature. 2009 May 21;459(7245):387-392. doi: 10.1038/nature08040. Epub 2009 Apr 29. PMID: 19404261; PMCID: PMC4721521.

			self-renewal implicated in mediating cellular senescence.		
Regucalcin	4	Protector	**Cell inmortality**<br>It is a calcium-binding protein which plays the role as a suppressor protein for cell signaling systems. It can inhibit nuclear protein kinase, protein phosphatase, and deoxyribonucleic acid and ribonucleic acid synthesis avoiding cell proliferation of damage cells.	✓	Ghanem NZ, Yamaguchi M. Regucalcin downregulation in human cancer. Life Sci. 2024 Jan 19;340:122448. doi: 10.1016/j.lfs.2024.122448. Epub ahead of print. PMID: 38246519.
c-Met	5	oncogenic	**Formation of new blood vessels**<br>It is a surface receptor tyrosine kinase which triggers the formation of new blood vessels that supply nutrients to the tumor and allows it to grow and to spread throughout multiple signaling pathways directly, by motogenic or morphogenic effects, or indirectly by the regulation of other angiogenic factors, turning into the stimulation of endothelial cells.	✓	You WK, McDonald DM. The hepatocyte growth factor/c-Met signaling pathway as a therapeutic target to inhibit angiogenesis. BMB Rep. 2008 Dec 31;41(12):833-9. doi: 10.5483/bmbrep.2008.41.12.833. PMID: 19123972; PMCID: PMC4417610.
VEGFR-3	5	oncogenic	**Endothelial regulation**<br>Receptor for *Vascular Endothelial Growth Factor with tyrosine kinase activity.* It is an important protein not only involved in vasculogenesis and angiogenesis but mainly in lymphangiogenesis.	✓	Witmer AN, van Blijswijk BC, Dai J, Hofman P, Partanen TA, Vrensen GF, Schlingemann RO. VEGFR-3 in adult angiogenesis. J Pathol. 2001 Nov;195(4):490-7. doi: 10.1002/path.969. PMID: 11745682.
β1 integrin	5	oncogenic	**Extracellular matrix adhesion**<br>It supports since endothelial cell proliferation via a block of MMP production to their assemble or migration by the activation of the integrin adhesion receptors.	✓	Mettouchi, A., & Meneguzzi, G. (2006). Distinct roles of β1 integrins during angiogenesis. *European journal of cell biology*, *85*(3-4), 243-247
5T4 oncofoetal antigen	6	proinvasive	**Cell migration and invasion activation**<br>Its expression may influence several processes occurring during embryogenesis, where migration or motility of cells and adhesion cell-cell interactions are critical.	✓	Ali, A., J. Langdon, P. Stern, and M. Partridge. 2001. "The Pattern of Expression of the 5T4 Oncofoetal Antigen on Normal, Dysplastic and Malignant Oral Mucosa." *Oral Oncology* 37(1):57–64. doi: 10.1016/s1368-8375(00)00057-9.
ABCG2	6	proinvasive	**Stem cell phenotype and multidrug resistance**<br>It is involved in the maintenance of the phenotype of cancer stem cell populations and it is a transporter protein that promotes the efflux of toxins and drugs into the extracellular environment through regulation	✓	Shi, Peng, Wei Liu, Zeng-Tong Zhou, Qing-Bo He, and Wei-Wen Jiang. 2010. "Podoplanin and ABCG2: Malignant Transformation Risk Markers for Oral Lichen Planus." *Cancer Epidemiology, Biomarkers & Prevention* 19(3):844–49. doi: 10.1158/1055-9965.EPI-09-0699.

			of ATP hydrolysis related to the increase of migration.		
ALDH1	6	proinvasive	**Stem cell differentation**<br>It is an enzyme essential for detoxification of endogenous and exogenous aldehyde substrates through NAD(P)$^+$-dependent oxidation. It is involved in the regulation and maintenance of cancer stem cell phenotype. Cancer cell-acquired drug resistance is associated with the transcriptional activation of ALDH1 expression.	✓	Xu, Ziyuan, Zhengyu Shen, Linjun Shi, Hongying Sun, Wei Liu, and Zengtong Zhou. 2013. "Aldehyde Dehydrogenase 1 Expression Correlated with Malignant Potential of Oral Lichen Planus." *Annals of Diagnostic Pathology* 17(5):408–11. doi: 10.1016/j.anndiagpath.2013.04.008.
cathepsin-B	6	proinvasive	**Cell migration and invasion activation**<br>Lysosomal proteases which regulate extracellular matrix degradation and cell invasion by various mechanisms.	✓	Satelur, Krishnanand Prakash, Shiny Bopaiah, Radhika Manoj Bavle, and Prashant Ramachandra. 2017. "Role of Cathepsin B as a Marker of Malignant Transformation in Oral Lichen Planus: An Immunohistochemical Study." *Journal of Clinical and Diagnostic Research : JCDR* 11(8):ZC29--ZC32. doi: 10.7860/JCDR/2017/30740.10274.
cathepsin-K	6	proinvasive	**Cell migration and invasion activation**<br>Lysosomal proteases which regulate extracellular matrix degradation and cell invasion by various mechanisms.	✓	Siponen, Maria, Carolina Cavalcante Bitu, Ahmed Al-Samadi, Pentti Nieminen, and Tuula Salo. 2016. "Cathepsin K Expression Is Increased in Oral Lichen Planus." *Journal of Oral Pathology & Medicine* 45(10):758–65. doi: 10.1111/jop.12446.
cathepsin-L	6	proinvasive	**Cell migration and invasion activation**<br>Lysosomal proteases regulate extracellular matrix degradation and cell invasion by various mechanisms.	✓	Kitkhajornkiat, Athip, Sorasun Rungsiyanont, Sineepat Talungchit, Pimporn Jirawechwongsakul, and Patrayu Taebunpakul. 2020. "The Expression of Cathepsin L in Oral Lichen Planus." *Journal of Oral Biology and Craniofacial Research* 10(3):281–86. doi: 10.1016/j.jobcr.2020.06.003.
caveolin-1	6	proinvasive	**Cell migration and invasion activation**<br>A protein that generally forms caveolae, which are invaginations of the plasma membrane. Caveolae have one transmembrane and two cytoplasmic microdomains and are critical components for interactions between integrin receptors and intracellular signalling molecules. Associated with malignant transformation, metastasis and resistance to chemotherapeutic treatment.	✓	Jaafari-Ashkavandi, Zohreh, and Ehsan Aslani. 2017. "Caveolin-1 Expression in Oral Lichen Planus, Dysplastic Lesions and Squamous Cell Carcinoma." *Pathology, Research and Practice* 213(7):809–14. doi: 10.1016/j.prp.2017.03.006.
CD133	6	proinvasive	**Cell migration and invasion activation**<br>One of the prototypical cancer stem cell biomarkers, which among its multiple roles activates the canonical Wnt oncogenic signalling pathway. It has been associated with increased cell migration and metastasis.	✓	Sun, Lili, Jinqiu Feng, Lihua Ma, Wei Liu, and Zengtong Zhou. 2013. "CD133 Expression in Oral Lichen Planus Correlated with the Risk for Progression to Oral Squamous Cell Carcinoma." *Annals of Diagnostic Pathology* 17(6):486–89. doi: 10.1016/j.anndiagpath.2013.06.004.
CD146	6	proinvasive	**Cell adhesion**	✓	Shih, I. M. (1999). The role of CD146 (Mel-CAM) in biology and pathology. *The Journal of pathology*, *189*(1), 4-11.

			Glycoprotein recently renamed METCAM (metastasis-regulating CAM). It is a cell-cell adhesion protein, usually located at endothelial cell junctions.		
MMP-10	6	proinvasive	**Cell migration and invasion**<br>It promotes cell migration and invasion through its zinc-dependent endopeptidase activity, capable of digesting the extracellular matrix and basement membrane.	✓	Chen, Yu, Weiping Zhang, Ning Geng, Kun Tian, and Lester Jack Windsor. 2008. “MMPs, TIMP-2, and TGF-$β$1 in the Cancerization of Oral Lichen Planus.” *Head & Neck* 30(9):1237–45. doi: 10.1002/hed.20869.
MMP-2	6	proinvasive	**Cell migration and invasion**<br>It promotes cell migration and invasion through proteolysis and degradation of collagen types IV, V and XI of the extracellular matrix.	✓	Chen, Yu, Weiping Zhang, Ning Geng, Kun Tian, and Lester Jack Windsor. 2008. “MMPs, TIMP-2, and TGF-$β$1 in the Cancerization of Oral Lichen Planus.” *Head & Neck* 30(9):1237–45. doi: 10.1002/hed.20869.<br>Agarwal, Neha, Sunitha Carnelio, and Gabriel Rodrigues. 2019. “Immunohistochemical and Clinical Significance of Matrix Metalloproteinase-2 and Its Inhibitor in Oral Lichen Planus.” *Journal of Oral and Maxillofacial Pathology : JOMFP* 23(3):476. doi: 10.4103/jomfp.JOMFP_27_19.
MMP-7	6	proinvasive	**Cell migration and invasion**<br>It promotes cell migration and invasion through proteolysis and degradation of type IV collagen of the extracellular matrix.	✓	Chen, Yu, Weiping Zhang, Ning Geng, Kun Tian, and Lester Jack Windsor. 2008. “MMPs, TIMP-2, and TGF-$β$1 in the Cancerization of Oral Lichen Planus.” *Head & Neck* 30(9):1237–45. doi: 10.1002/hed.20869.<br>Li, Tie-Jun, and Jun Cui. 2013. “COX-2, MMP-7 Expression in Oral Lichen Planus and Oral Squamous Cell Carcinoma.” *Asian Pacific Journal of Tropical Medicine* 6(8):640–43. doi: 10.1016/S1995-7645(13)60110-8.
MMP-9	6	proinvasive	**Cell migration and invasion**<br>It promotes cell migration and invasion through proteolysis and degradation of basement membrane type IV collagen and extracellular matrix.	✓	Chen, Yu, Weiping Zhang, Ning Geng, Kun Tian, and Lester Jack Windsor. 2008. “MMPs, TIMP-2, and TGF-$β$1 in the Cancerization of Oral Lichen Planus.” *Head & Neck* 30(9):1237–45. doi: 10.1002/hed.20869.<br>Agarwal, Neha, Sunitha Carnelio, and Gabriel Rodrigues. 2019. “Immunohistochemical and Clinical Significance of Matrix Metalloproteinase-2 and Its Inhibitor in Oral Lichen Planus.” *Journal of Oral and Maxillofacial Pathology : JOMFP* 23(3):476. doi: 10.4103/jomfp.JOMFP_27_19.
MT1-MMP	6	proinvasive	**Cell migration and invasion**<br>It is a type I transmembrane proteinase whose promoter mechanism for cell invasion and motility is mediated by the proteolysis of extracellular matrix macromolecules.	✓	Itoh, Y., & Seiki, M. (2006). MT1-MMP: a potent modifier of pericellular microenvironment. *Journal of cellular physiology*, *206*(1), 1-8.
nestin	6	proinvasive	**EMT induction**<br>It is a is a cytoskeletal intermediate filament constituted by a single type VI protein. It is considered a key player in polarity maintenance in migrating cells. It also regulates focal adhesion dynamics. Cell invasion is due to the significant increase in	✓	Bernal A, Arranz L. Nestin-expressing progenitor cells: function, identity and therapeutic implications. Cell Mol Life Sci. 2018 Jun;75(12):2177-2195. doi: 10.1007/s00018-018-2794-z. Epub 2018 Mar 14. PMID: 29541793; PMCID: PMC5948302.

			phosphorylated focal adhesion kinase and the integrin-dependent matrix degradation.		
Paxillin	6	proinvasive	**EMT induction**<br>Protein associated with cell migration gain through EMT regulation. It plays essential roles during the formation of focal adhesions, which are necessary for the regulation of cell motility. Has been associated with the development of metastasis.	✓	Aghili, Seyedeh Sara, Razieh Zare, and Alireza Jahangirnia. 2023. “Evaluation of Paxillin Expression in Epithelial Dysplasia, Oral Squamous Cell Carcinoma, Lichen Planus with and without Dysplasia, and Hyperkeratosis: A Retrospective Cross-Sectional Study.” *Diagnostics (Basel, Switzerland)* 13(15). doi: 10.3390/diagnostics13152476.
periostin	6	proinvasive	**EMT induction**<br>A soluble extracellular matrix protein that plays a role in embryonic development. It is involved in cell migration gain through regulation of EMT. It has been associated with cell invasion and metastasis.	✓	Zhang, Zhi-Rui, Li-Ya Chen, Hong-Yan Qi, and Shao-Hua Sun. 2018. “Expression and Clinical Significance of Periostin in Oral Lichen Planus.” *Experimental and Therapeutic Medicine* 15(6):5141–47. doi: 10.3892/etm.2018.6029.
podoplanin	6	proinvasive	**EMT induction**<br>It is a transmembrane receptor glycoprotein whose expression is induced by tumor promoters such as TPA, RAS and Src, activating cell motility.	✓	Krishnan H, Rayes J, Miyashita T, Ishii G, Retzbach EP, Sheehan SA, Takemoto A, Chang YW, Yoneda K, Asai J, Jensen L, Chalise L, Natsume A, Goldberg GS. Podoplanin: An emerging cancer biomarker and therapeutic target. Cancer Sci. 2018 May;109(5):1292-1299. doi: 10.1111/cas.13580. PMID: 29575529; PMCID: PMC5980289.
syndecan-1	6	**Proinvasive**	**Cell adhesion**<br>It is required for the maintenance of epithelial junctions, such as adherens junctions, as has been linked to E-cadherin expression.	✓	Couchman JR. Syndecan-1 (CD138), Carcinomas and EMT. Int J Mol Sci. 2021 Apr 19;22(8):4227. doi: 10.3390/ijms22084227. PMID: 33921767; PMCID: PMC8072910.
TIMP-2	6	anti-invasive	**MMP inhibitor**<br>Endogenous inhibitor of the proteolytic activity of MMPs, associated with decreased cell migration and invasion.	✓	Agarwal, Neha, Sunitha Carnelio, and Gabriel Rodrigues. 2019. “Immunohistochemical and Clinical Significance of Matrix Metalloproteinase-2 and Its Inhibitor in Oral Lichen Planus.” *Journal of Oral and Maxillofacial Pathology : JOMFP* 23(3):476. doi: 10.4103/jomfp.JOMFP_27_19.
vimentin	6	pro-invasive	**EMT induction**<br>It is an intermediate structural filament protein required for mesenchymal cell migration who directly interacts with actin, β1 integrin and paxillin. Its filaments are centrally involved in cell extension formation and migration through fibronectin.	✓	Gilles, C., POLETTE, M., Piette, J., DELVIGNE, A. C., Thompson, E. W., FOIDART, J. M., & BIREMBAUT, P. (1996). Vimentin expression in cervical carcinomas: association with invasive and migratory potential. *The Journal of pathology*, *180*(2), 175-180.

PD-L1	7	Oncogenic	**Immunity response arrest**<br>Programmed death ligand 1 is a coinhibitory receptor that can be constitutively expressed or induced which is involved in the development of immune tolerance preventing excessive immune cell activity that can lead to tissue destruction and autoimmunity constituting an immune evasion mechanism	✓	Kythreotou, A., Siddique, A., Mauri, F. A., Bower, M., & Pinato, D. J. (2017). PD-L1. *Journal of clinical pathology*.
PD-L2	7	Oncogenic	**Immunity response arrest**<br>It allows to inhibit T-cell proliferation, cytokine production and cell adhesion. It triggers reverse signaling in dendritic cells leading to IL-12 production and activation of T cells.	✓	Marguerite Ghiotto, Laurent Gauthier, Nacer Serriari, Sonia Pastor, Alemseged Truneh, Jacques A. Nunès, Daniel Olive, PD-L1 and PD-L2 differ in their molecular mechanisms of interaction with PD-1, *International Immunology*, Volume 22, Issue 8, August 2010, Pages 651–660.
CA9	8	Oncogenic	**Maintenance of intracellular acidosis.**<br>Biomarker involved in the catalytic hydration of carbon dioxide to carbonic acid which creates an acidic microenvironment. This induces hypoxia therefore it allows cells to rapidly outgrow blood supply correlating with cell survival.	✓	Guan SS, Cheng CC, Ho AS, Wang CC, Luo TY, Liao TZ, Chang J, Wu CT, Liu SH. Sulfonamide derivative targeting carbonic anhydrase IX as a nuclear imaging probe for colorectal cancer detection in vivo. Oncotarget. 2015 Nov 3;6(34):36139-55. doi: 10.18632/oncotarget.5684. PMID: 26447758; PMCID: PMC4742167.
53BP1	9	Protector	**DNA damage repair**<br>Its function is based on the repair of double-strand breaks (DSBs) by promoting the joining of non-homologous ends. It controls 5' end resection, mediates DNA end synapsis and promotes the mobility of damaged chromatin.	✓	Zimmermann M, de Lange T. 53BP1: pro choice in DNA repair. Trends Cell Biol. 2014 Feb;24(2):108-17. doi: 10.1016/j.tcb.2013.09.003. Epub 2013 Oct 4. PMID: 24094932; PMCID: PMC3946699.
BUBR1	9	Protector	**DNA damage repair**<br>Its function is based on maintaining the high fidelity of mitotic chromosome segregation by preventing cells from initiating anaphase. If one or more kinetochores are not attached to the spindle, thereby helping to promote the establishment of stable kinetochore-microtubule junctions during prometaphase.	✓	Karess RE, Wassmann K, Rahmani Z. New insights into the role of BubR1 in mitosis and beyond. Int Rev Cell Mol Biol. 2013;306:223-73. doi: 10.1016/B978-0-12-407694-5.00006-7. PMID: 24016527.
hMSH2	9	Protector	**DNA damage repair**<br>It eliminates insertion and deletion loops resulting from primer slippage in repetitive DNA sequences and corrects mismatches of individual bases that escape polymerase correction, thus preventing the accumulation of spontaneous mutations and ensuring genome integrity and stability.	✓	Seifert M, Reichrath J. The role of the human DNA mismatch repair gene hMSH2 in DNA repair, cell cycle control and apoptosis: implications for pathogenesis, progression and therapy of cancer. J Mol Histol. 2006 Sep;37(5-7):301-7. doi: 10.1007/s10735-006-9062-5. Epub 2006 Nov 2. PMID: 17080293.

HSP90	9	Oncogenic	**DNA instability**<br>A molecular chaperone required for the maturation and activation of a number of key cellular proteins. It acts in the assembly of telomeric complexes.	✓	Jackson SE. Hsp90: structure and function. Top Curr Chem. 2013;328:155-240. doi: 10.1007/128_2012_356. PMID: 22955504.
MAGE-A	9	oncogenic	**DNA instability**<br>The melonoma-associated antigen gets activated while the epigenetic reprogramming occurs in tumors, this triggeres their aberrant expression.	✓	Sang, M., Lian, Y., Zhou, X., & Shan, B. (2011). MAGE-A family: attractive targets for cancer immunotherapy. *Vaccine*, *29*(47), 8496-8500.
SUMO-1	9	oncogenic	**DNA instability**<br>Responsible for the ubiquinization of proteins. Facilitates protein degradation by a complex protease called 26s2J proteasome.	✓	Melchior, F., & Hengst, L. (2002). SUMO-1 and p53. *Cell Cycle*, *1*(4), 243-247.
TOPO I	9	oncogenic	**DNA instability**<br>It mediates damaged DNA. It is involved in replication, transcription and DNA recombination. Has a catalytic mechanism involving nucleophilic attack of a phosphodiester bond in DNA by a catalytic tyrosyl residue.	✓	Pommier, Y. (2006). Topoisomerase I inhibitors: camptothecins and beyond. *Nature Reviews Cancer*, *6*(10), 789-802.
CD20	10	pro-inflammatory	**Inflammatory response**<br>CD20 is a transmembrane protein expressed on the surface of B lymphocytes, which plays an important role in the development and differentiation of these cells.	✓	Nagao, Y., M. Sata, M. Kage, T. Kameyama, and T. Ueno. 2000. “Histopathological and Immunohistochemical Study of Oral Lichen Planus-Associated HCV Infection.” *European Journal of Internal Medicine* 11(5):277–82. doi: 10.1016/s0953-6205(00)00107-2.
CD4	10	pro-inflammatory	**Inflammatory response**<br>A molecule that is expressed on the surface of some T cells, particularly CD4+ T cells from the lymphocyte infiltrate during the chronic inflammatory response in OLP.	✓	Enomoto, Ai, Eiichi Sato, Takashi Yasuda, Tatsuya Isomura, Toshitaka Nagao, and Daichi Chikazu. 2018. “Intraepithelial CD8+ Lymphocytes as a Predictive Diagnostic Biomarker for the Remission of Oral Lichen Planus.” *Human Pathology* 74:43–53. doi: 10.1016/j.humpath.2017.12.008.<br>Squarzanti, Diletta Francesca, Tiziana Cena, Rita Sorrentino, Mario Migliario, Annalisa Chiocchetti, Lia Rimondini, Barbara Azzimonti, and Guido Valente. 2019. “Implications on Pathogenesis and Risk of Oral Lichen Planus Neoplastic Transformation: An Ex-Vivo Retrospective Immunohistochemical Study.” *Histology and Histopathology* 34(9):1015–24. doi: 10.14670/HH-18-104.
CD45	10	pro-inflammatory	**Inflammatory response**<br>CD45 is a protein tyrosine phosphatase that is present in all leukocytes, with higher overexpression in lymphocytes, playing a relevant role in the regulation of the differentiation of these cells.	✓	Nagao, Y., M. Sata, M. Kage, T. Kameyama, and T. Ueno. 2000. “Histopathological and Immunohistochemical Study of Oral Lichen Planus-Associated HCV Infection.” *European Journal of Internal Medicine* 11(5):277–82. doi: 10.1016/s0953-6205(00)00107-2.

CD8	10	pro-inflammatory	**Inflammatory response**<br>A molecule that is expressed on the surface of some T cells, particularly CD8+ T cells in the lymphocyte infiltrate during the chronic inflammatory response in OLP.	✓	Enomoto, Ai, Eiichi Sato, Takashi Yasuda, Tatsuya Isomura, Toshitaka Nagao, and Daichi Chikazu. 2018. “Intraepithelial CD8+ Lymphocytes as a Predictive Diagnostic Biomarker for the Remission of Oral Lichen Planus.” *Human Pathology* 74:43–53. doi: 10.1016/j.humpath.2017.12.008.<br>Squarzanti, Diletta Francesca, Tiziana Cena, Rita Sorrentino, Mario Migliario, Annalisa Chiocchetti, Lia Rimondini, Barbara Azzimonti, and Guido Valente. 2019. “Implications on Pathogenesis and Risk of Oral Lichen Planus Neoplastic Transformation: An Ex-Vivo Retrospective Immunohistochemical Study.” *Histology and Histopathology* 34(9):1015–24. doi: 10.14670/HH-18-104.
COX-2	10	pro-inflammatory	**Inflammation and tumour growth promotion**<br>COX2 is the enzyme responsible for regulating the metabolism of arachidonic acid and its conversion to prostaglandin H2, a potent inducer of the inflammatory response, implicated in numerous related processes (pain, fever, vasodilatation, increased tissue permeability allowing the entry of leukocytes, among others). Numerous specific oncogenic actions have been attributed to COX2, such as activation of cell proliferation, inhibition of apoptosis and neoangiogenesis.	✓	Neppelberg, Evelyn, and Anne Christine Johannessen. 2007. “DNA Content, Cyclooxygenase-2 Expression and Loss of E-Cadherin Expression Do Not Predict Risk of Malignant Transformation in Oral Lichen Planus.” *European Archives of Oto-Rhino-Laryngology* 264(10):1223–30. doi: 10.1007/s00405-007-0346-5.
CXCL9	10	pro-inflammatory	**Chemotaxis and inflamation regulation**<br>CXCL9 is a chemokine activated by proinflammatory stimuli that can orchestrate innate and adaptive immune responses to control neutrophil and effector lymphocyte recruitment to foci of infection and inflammation.	✓	Li, Ning, Qiong Hu, Canhua Jiang, Feng Guo, Krishna Munnee, Xinchun Jian, Yanjia Hu, and Zhangui Tang. 2013. “Cys-X-Cys Ligand 9 Might Be an Immunological Factor in the Pathogenesis of Oral Submucous Fibrosis and Its Concomitant Oral Lichenoid Lesion.” *Clinical Oral Investigations* 17(4):1251–58. doi: 10.1007/s00784-012-0799-9.
FoxP3+	10	pro-inflammatory	**Tumour microenvironment regulator**<br>Specific marker of natural regulatory T cells (Tregs), which exert regulatory roles during the immune response.	✓	Enomoto, Ai, Eiichi Sato, Takashi Yasuda, Tatsuya Isomura, Toshitaka Nagao, and Daichi Chikazu. 2018. “Intraepithelial CD8+ Lymphocytes as a Predictive Diagnostic Biomarker for the Remission of Oral Lichen Planus.” *Human Pathology* 74:43–53. doi: 10.1016/j.humpath.2017.12.008.
HAS1	10	pro-inflammatory	**Inflammation and tumour growth promotion**<br>Hyaluronan synthase 1 responsible for cellular hyaluronan synthesis a pro-inflammatory molecule on its low-molecular-weight form.	✓	Siponen, Maria, Arja Kullaa, Pentti Nieminen, Tuula Salo, and Sanna Pasonen-Seppänen. 2015. “Altered Expression of Hyaluronan, HAS1-2, and HYAL1-2 in Oral Lichen Planus.” *Journal of Oral Pathology & Medicine* 44(6):401–9. doi: 10.1111/jop.12294.
HAS2	10	pro-inflammatory	**Inflammation and tumor progression**<br>Hyaluronan synthase 2 responsible for cellular hyaluronan synthesis a pro-inflammatory molecule on its low-molecular-weight form.	✓	Siponen, Maria, Arja Kullaa, Pentti Nieminen, Tuula Salo, and Sanna Pasonen-Seppänen. 2015. “Altered Expression of Hyaluronan, HAS1-2, and HYAL1-2 in Oral Lichen Planus.” *Journal of Oral Pathology & Medicine* 44(6):401–9. doi: 10.1111/jop.12294.

HYAL1	10	pro-inflammatory	**Inflammation and tumor progression**<br>It is an enzyme which regulates catabolism and molecular weight of Hyaluronan. It promotes the degradation of the high molecular weight Hyaluronan form with anti-inflammatory properties carrying out a pro-inflammatory role.	✓	Kaul A, Short WD, Wang X, Keswani SG. Hyaluronidases in Human Diseases. Int J Mol Sci. 2021 Mar 22;22(6):3204. doi: 10.3390/ijms22063204. PMID: 33809827; PMCID: PMC8004219
HYAL2	10	pro-inflammatory	**Inflammation and tumor progression**<br>It is an enzyme which regulates catabolism and molecular weight of Hyaluronan. It promotes the degradation of the high molecular weight Hyaluronan form with anti-inflammatory properties, carrying out a pro-inflammatory role.	✓	Kaul A, Short WD, Wang X, Keswani SG. Hyaluronidases in Human Diseases. Int J Mol Sci. 2021 Mar 22;22(6):3204. doi: 10.3390/ijms22063204. PMID: 33809827; PMCID: PMC8004219.
Hyaluronan	10	pro-inflammatory	**Inflammation and tumor progression**<br>Its low-molecular-weight HA (LMW-HA) form, lead to inflammation. It is able to activate macrophages and dendritic cells as well as stimulate transcription of inflammation-related genes such as TNF-α, IL-12, IL-1β and matrix metalloproteinases.	✓	Petrey, A. C., & de la Motte, C. A. (2014). Hyaluronan, a crucial regulator of inflammation. *Frontiers in immunology*, *5*, 101.
IL-1α	10	pro-inflammatory	**Proinflammatory**<br>Interleukin 1α is a multifunctional cytokine with proinflammatory activity, mainly produced by macrophages and neutrophils.	✓	Babiuch, Karolina, Beata Kuśnierz-Cabala, Barbara Kęsek, Krzysztof Okoń, Dagmara Darczuk, and Maria Chomyszyn-Gajewska. 2020b. "Evaluation of Proinflammatory, Nf-Kappab Dependent Cytokines: Il-1α, Il-6, Il-8, and TNF-α in Tissue Specimens and Saliva of Patients with Oral Squamous Cell Carcinoma and Oral Potentially Malignant Disorders." *Journal of Clinical Medicine* 9(3). doi: 10.3390/jcm9030867.
IL-27	10	pro-inflammatory	**Proinflammatory**<br>Interleukin-27 is a multifunctional cytokine that regulates T- and B-lymphocyte activity during inflammation.	✓	Wang, Qing-Mei, Xue-Ying Huang, and Wei-Qun Guan. 2022. "Expressions of Interleukin-27 in Oral Lichen Planus, Oral Leukoplakia, and Oral Squamous Cell Carcinoma." *Inflammation* 45(3):1023–38. doi: 10.1007/s10753-021-01599-5.
IL-6	10	pro-inflammatory	**Proinflammatory**<br>Interleukin-6 is a multifunctional cytokine with proinflammatory activity, released in response to TNFα and IL-1.	✓	Babiuch, Karolina, Beata Kuśnierz-Cabala, Barbara Kęsek, Krzysztof Okoń, Dagmara Darczuk, and Maria Chomyszyn-Gajewska. 2020b. "Evaluation of Proinflammatory, Nf-Kappab Dependent Cytokines: Il-1α, Il-6, Il-8, and TNF-α in Tissue Specimens and Saliva of Patients with Oral Squamous Cell Carcinoma and Oral Potentially Malignant Disorders." *Journal of Clinical Medicine* 9(3). doi: 10.3390/jcm9030867.
IL-8	10	pro-inflammatory	**Proinflammatory**<br>Interleukin-8 is a multifunctional cytokine with proinflammatory activity, considered a potent	✓	Babiuch, Karolina, Beata Kuśnierz-Cabala, Barbara Kęsek, Krzysztof Okoń, Dagmara Darczuk, and Maria Chomyszyn-Gajewska. 2020b. "Evaluation of Proinflammatory, Nf-Kappab Dependent Cytokines: Il-1α, Il-6, Il-8, and TNF-α in

			neutrophil chemotactic factor, amplifies local inflammation and stimulates angiogenesis.		Tissue Specimens and Saliva of Patients with Oral Squamous Cell Carcinoma and Oral Potentially Malignant Disorders.” *Journal of Clinical Medicine* 9(3). doi: 10.3390/jcm9030867.
OPN	10	pro-inflammatory	**Proinflammatory**<br>Osteopontin is a glycoprotein cytokine synthesised by immune cells that appears to exert a proinflammatory role following exposure to other inflammatory mediators, such as TNFα or IL-1β.	✓	Squarzanti, Diletta Francesca, Tiziana Cena, Rita Sorrentino, Mario Migliario, Annalisa Chiocchetti, Lia Rimondini, Barbara Azzimonti, and Guido Valente. 2019. “Implications on Pathogenesis and Risk of Oral Lichen Planus Neoplastic Transformation: An Ex-Vivo Retrospective Immunohistochemical Study.” *Histology and Histopathology* 34(9):1015–24. doi: 10.14670/HH-18-104.
p65	10	pro-inflammatory	**Pro-inflammatory**<br>It is a member of the transcription factorNF-κB which is critical for lymphocytes and macrophages. It has the ability to regulate the promoters of a variety of genes whose products, such as cytokines or adhesion molecules, are critical for inflammatory processes.	✓	Neurath, M. F., & Pettersson, S. (1997). Predominant role of NF-κB p65 in the pathogenesis of chronic intestinal inflammation. *Immunobiology*, *198*(1-3), 91-98.
T-bet+	10	pro-inflammatory	**Tumour microenvironment regulator**<br>T-Bet protein (T-box protein expressed in T cells) is involved during the inflammatory response, inducing the differentiation of T helper 1 (Th1) cells, accelerator cells of the cytotoxic immune response.	✓	Enomoto, Ai, Eiichi Sato, Takashi Yasuda, Tatsuya Isomura, Toshitaka Nagao, and Daichi Chikazu. 2018. “Intraepithelial CD8+ Lymphocytes as a Predictive Diagnostic Biomarker for the Remission of Oral Lichen Planus.” *Human Pathology* 74:43–53. doi: 10.1016/j.humpath.2017.12.008.
TNF-α	10	pro-inflammatory	**Proinflammatory**<br>A multifunctional cytokine released by immune cells that plays an important role during inflammation and angiogenesis, among other processes.	✓	Babiuch, Karolina, Beata Kuśnierz-Cabala, Barbara Kęsek, Krzysztof Okoń, Dagmara Darczuk, and Maria Chomyszyn-Gajewska. 2020b. “Evaluation of Proinflammatory, Nf-Kappab Dependent Cytokines: Il-1α, Il-6, Il-8, and TNF-α in Tissue Specimens and Saliva of Patients with Oral Squamous Cell Carcinoma and Oral Potentially Malignant Disorders.” *Journal of Clinical Medicine* 9(3). doi: 10.3390/jcm9030867.
CD44	U	Unespecified	A transmembrane glycoprotein involved in interactions between cells, adhesion and migration. It is a receptor for many ligands such as hyaluronic acid, osteopontine, collagens and matrix metalloproteinases.	✓	Xu, H., Niu, M., Yuan, X., Wu, K., & Liu, A. (2020). CD44 as a tumor biomarker and therapeutic target. *Experimental Hematology & Oncology*, *9*(1), 1-14.
CK1	U	Unespecified	It is involved mainly in modulating a variety of cellular functions specially immune response, inflammation, DNA damaging and apoptosis throughout different mechanism as phosphorylation by inhibitory autophosphorylation, phosphorylation by other cellular protein kinases, and interaction	✓	Knippschild, U., Krüger, M., Richter, J., Xu, P., García-Reyes, B., Peifer, C., ... & Bischof, J. (2014). The CK1 family: contribution to cellular stress response and its role in carcinogenesis. *Frontiers in oncology*, *4*, 96.

			with cellular proteins or subcellular sequestration.		
CK10	U	Unespecified	It is an intermediate filament protein expressed in stratified epithelial tissues. Its function encompasses providing structural support and stability to keratinocytes.<br>It is involved in the process of keratinocyte differentiation.	✓	Uhlig, R., Abboud, M., Gorbokon, N., Lennartz, M., Rico, S. D., Kind, S., ... & Menz, A. (2022). Cytokeratin 10 (CK10) expression in cancer: A tissue microarray study on 11,021 tumors. *Annals of Diagnostic Pathology*, *60*, 152029.
CK13	U	Unespecified	It is an intermediate filament protein primarily found in epithelial tissues, in stratified squamous epithelia. Its functions involves providing structural support and mechanical strength to cells, contributing to the maintenance of tissue integrity and regulating cell shape, adhesion, and migration processes. It is also implicated in epithelial differentiation and wound healing mechanisms.	✓	Lennartz, M., Ullmann, V. S., Gorbokon, N., Uhlig, R., Rico, S. D., Kind, S., ... & Menz, A. (2023). Cytokeratin 13 (CK13) expression in cancer: a tissue microarray study on 10,439 tumors. *APMIS*, *131*(2), 77-91.
CK14	U	Unespecified	It is an intermediate filament protein, predominantly expressed in basal epithelial cells, providing structural integrity and resilience to various tissues, notably in stratified epithelia. Its function includes anchoring epithelial cells to the basement membrane and regulating cell proliferation and differentiation processes.	✓	Xue, L. Y., Zou, S. M., Zheng, S., Xie, Y. Q., Wen, P., Liu, X. Y., ... & Lü, N. (2010). Expression of fascin and CK14 in different histological types of cancer and its differential diagnostic significance. *Zhonghua zhong liu za zhi [Chinese journal of oncology]*, *32*(11), 838-844.
CK19	U	Unespecified	It is a type I intermediate filament protein. Its functions encompass providing structural support and stability to cells, particularly during cell division and migration processes. It is involved in the formation and maintenance of cellular junctions and contributes to epithelial tissue integrity and repair.	✓	Jain, R., Fischer, S., Serra, S., & Chetty, R. (2010). The use of cytokeratin 19 (CK19) immunohistochemistry in lesions of the pancreas, gastrointestinal tract, and liver. *Applied Immunohistochemistry & Molecular Morphology*, *18*(1), 9-15.
CK2e	U	Unespecified	It is a type II intermediate filament protein primarily expressed in stratified epithelial tissues. It provides structural stability and integrity to epithelial cells, specifically in the context of keratinocyte differentiation. Additionally, it contributes to the regulation of cell proliferation and differentiation processes.	✓	Collin, C., Moll, R., Kubicka, S., Ouhayoun, J. P., & Franke, W. W. (1992). Characterization of human cytokeratin 2, an epidermal cytoskeletal protein synthesized late during differentiation. *Experimental cell research*, *202*(1), 132-141.
E-cadherin	U	Unespecified	It is a cell adhesion molecule which mediates calcium-dependent cell-cell adhesion. It promotes cell-cell adhesion and maintaining	✓	Van Roy, F., & Berx, G. (2008). The cell-cell adhesion molecule E-cadherin. *Cellular and molecular life sciences*, *65*, 3756-3788.

			tissue integrity by forming adherens junctions between adjacent cells. It regulates cell differentiation and migration during embryonic development. Additionally, it suppresses tumor invasion and metastasis by promoting epithelial cohesion and inhibiting epithelial-mesenchymal transition.		
laminin-332	U	Unespecified	It represents an epithelial-basement membrane specific laminin variant crucial for facilitating epithelial cell adhesion to the basement membrane. Its role extends to pivotal functions in the nucleation and sustenance of anchoring structures. Moreover, its proteolytic cleavages can induce disparate cellular events contingent upon their extent.	✓	Marinkovich, M. P. (2007). Laminin 332 in squamous-cell carcinoma. *Nature Reviews Cancer*, *7*(5), 370-380.
β-catenin	U	Unespecified	It is an integral structural component of cadherin-based adherens junctions which regulates Wnt signalling. In the absence of a Wnt signal, β-catenin is phosphorylated and degraded in a complex of proteins accumulated in the nucleus, where it interacts with transcription factors, leading to erroneous homeostatic processes	✓	Valenta, T., Hausmann, G., & Basler, K. (2012). The many faces and functions of β-catenin. *The EMBO journal*, *31*(12), 2714-2736.
Smad-2	U	Unespecified	TGF-β signals' transduction is done by two types of receptors with serine/ threonine kinase activity (TGF-β RII, TGF-β RI). Upon the binding of TGF-β to TGF-βRII, TGF-βRI is activated by phosphorylation. TGF-βRI in turn phosphorylates Smad2 and Smad3 forming a hetero-oligomer with Smad4, resulting a Smad complex which interacts through other DNA-binding proteins with DNA, regulating transcription of the target genes to lead the regulation of cellular proliferation.	✓	*Miyaki, M., & Kuroki, T. (2003). Role of Smad4 (DPC4) inactivation in human cancer. Biochemical and biophysical research communications, 306(4), 799-804*
Smad-3	U	Unespecified	TGF-β signals' transduction is done by two types of receptors with serine/ threonine kinase activity (TGF-β RII, TGF-β RI). Upon the binding of TGF-β to TGF-βRII, TGF-βRI is activated by phosphorylation. TGF-βRI in turn phosphorylates Smad2 and Smad3 forming a hetero-oligomer with Smad4, resulting a Smad	✓	*Miyaki, M., & Kuroki, T. (2003). Role of Smad4 (DPC4) inactivation in human cancer. Biochemical and biophysical research communications, 306(4), 799-804*

			complex which interacts through other DNA-binding proteins with DNA, regulating transcription of the target genes to lead the regulation of cellular proliferation.		
Smad-4	U	Unespecified	TGF-β signals' transduction is done by two types of receptors with serine/ threonine kinase activity (TGF-β RII, TGF-β RI). Upon the binding of TGF-β to TGF-βRII, TGF-βRI is activated by phosphorylation. TGF-βRI in turn phosphorylates Smad2 and Smad3 forming a hetero-oligomer with Smad4, resulting a Smad complex which interacts through other DNA-binding proteins with DNA, regulating transcription of the target genes to lead the regulation of cellular proliferation.	✓	*Miyaki, M., & Kuroki, T. (2003). Role of Smad4 (DPC4) inactivation in human cancer. Biochemical and biophysical research communications, 306(4), 799-804*
Smad-7	U	Unespecified	It regulates TGF-β signalling. It is an inhibitory Smad that lacks the conserved MH1 domain capable of blocking the phosphorylation of R-Smads.	✓	Yan, X., Liu, Z., & Chen, Y. (2009). Regulation of TGF-β signaling by Smad7. *Acta Biochim Biophys Sin*, *41*(4), 263-272.
TGF-β1	U	Unespecified	It is a cytokine able to inhibit epithelial cell proliferation by arresting the progression of the cell cycle at the GI-to-S phase transition. It can lead different pathways such as the Smad-mediated transcriptional regulation.	✓	Mazars, P., Barboule, N., Baldin, V., Vidal, S., Ducommun, B., & Valette, A. (1995). Effects of TGF-β1 (transforming growth factor-β1) on the cell cycle regulation of human breast adenocarcinoma (MCF-7) cells. *FEBS letters*, *362*(3), 295-300.

8. Risk of bias analysis.

Table S4.Quality plot graphically representing the risk of bias the risk of bias in individual studies, critically appraising ten domains, using a method specifically designed for systematic reviews addressing questions of prevalence (developed by the Joanna Briggs Institute, University of Adelaide, South Australia). Green, low risk of potential bias; yellow, moderate; red, high**.**

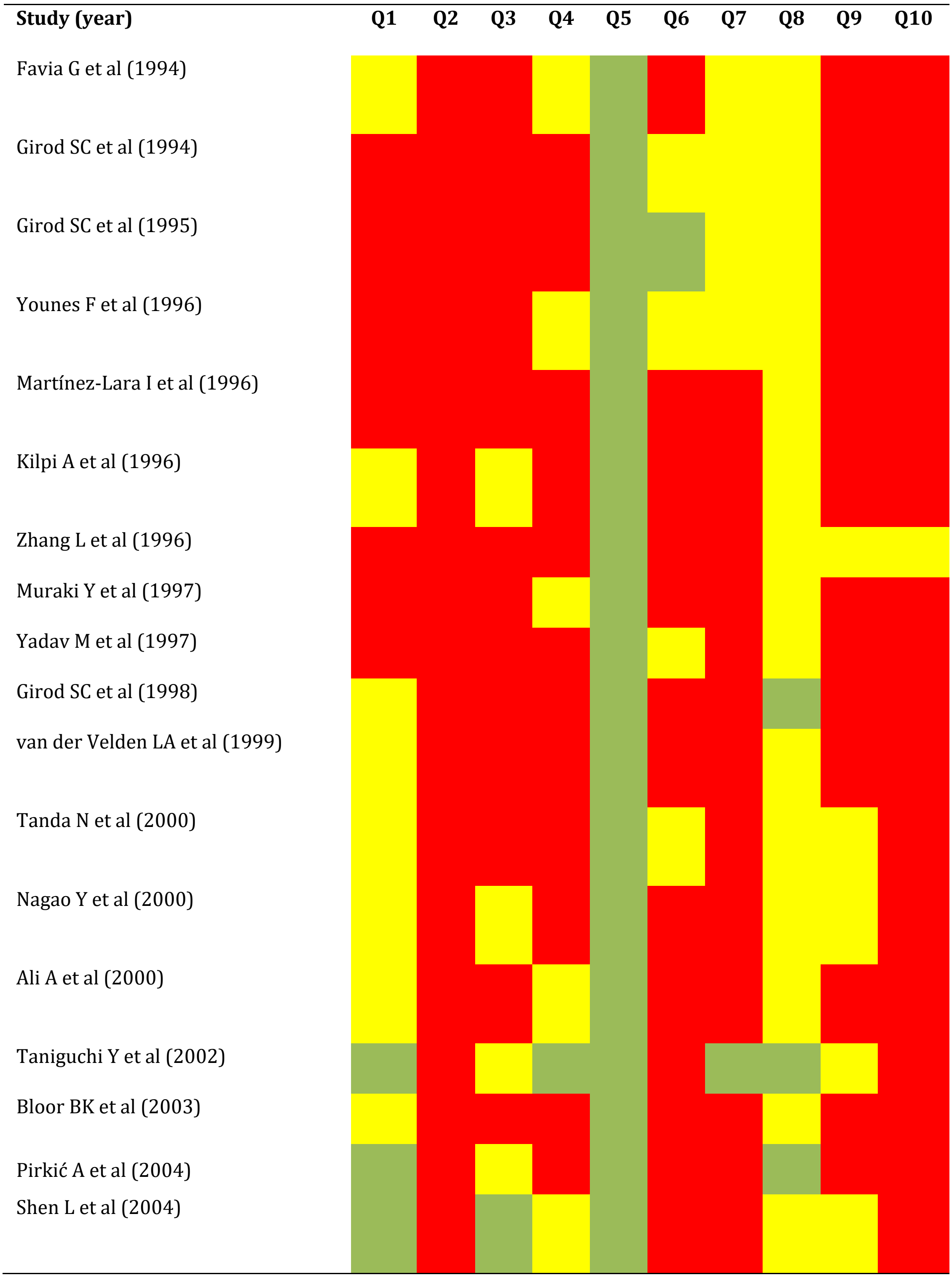

Fan Y et al (2004)
Lee JJ et al (2005)
Brunotto M et al (2005)
Bascones C et al (2005)
Thongprasom K et al (2006)
Acay RR et al (2006)
González-Moles MA et al (2006)
Neppelberg E and Johannessen AC (2007)
Bascones-Ilundain C et al (2008)
Gonzalez-Moles MA et al (2008)
Chen Y et al (2008)
González Moles MA et al (2009)
de Sousa FA et al (2009)
Ogmundsdóttir HM et al (2009a)
Jacques CM et al (2009)
Hadzi-Mihailovic et al (2009)
de Sousa FA et al (2009)
Oluwadara O et al (2009)
Ogmundsdóttir HM et al (2009b)
Danielsson et al (2010)
Shi, Y-J et al (2010)
Hsieh PC et al (2010)

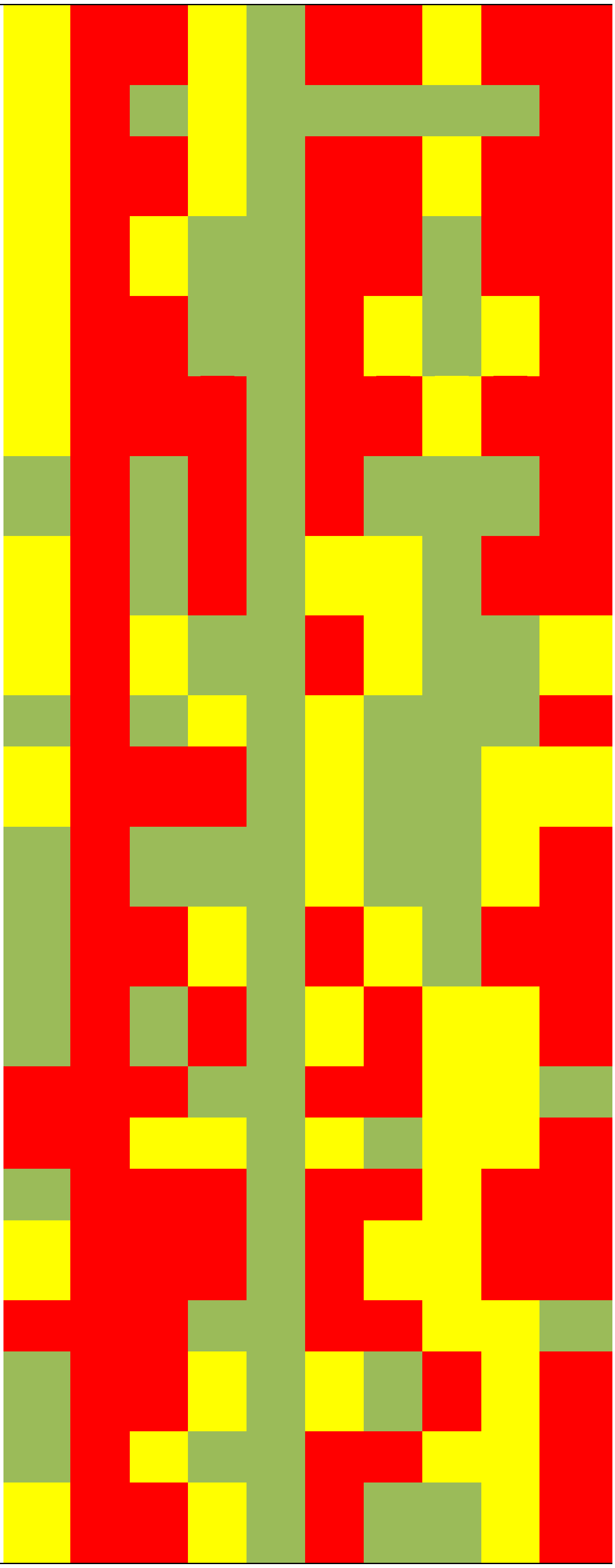

Cortés-Ramírez DA et al (2010)

Zyada MM and Fikry HE (2010)

Martín-Ezquerra G et al (2010)

Shi P et al (2010)

Du et al (2011)

Kłosek et al (2011)

Montebugnoli et al (2011)

Poomsawat et al (2011)

Sudha VM and Hemavathy S (2011)

Krauss E et al (2011)

Martín-Ezquerra G et al (2011)

Danielsson et al (2012)

Leyva-Huerta et al (2012)

Zhao, M et al (2012)

Nafarzadeh et al (2013)

Sun et al (2013)

Li and Cui (2013)

Nafarzadeh et al (2013)

Xu et al (2013)

Ma et al (2013)

Oliveira Alves et al (2013)

Prodromidis et al (2013)

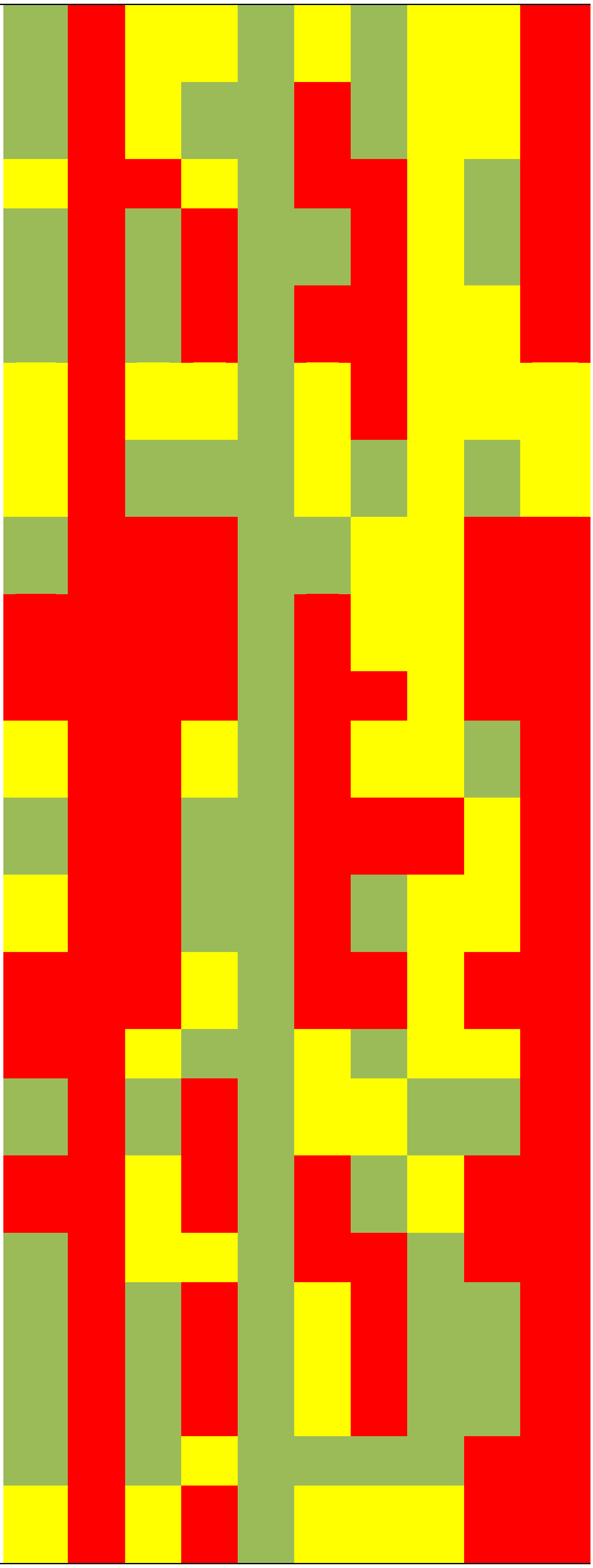

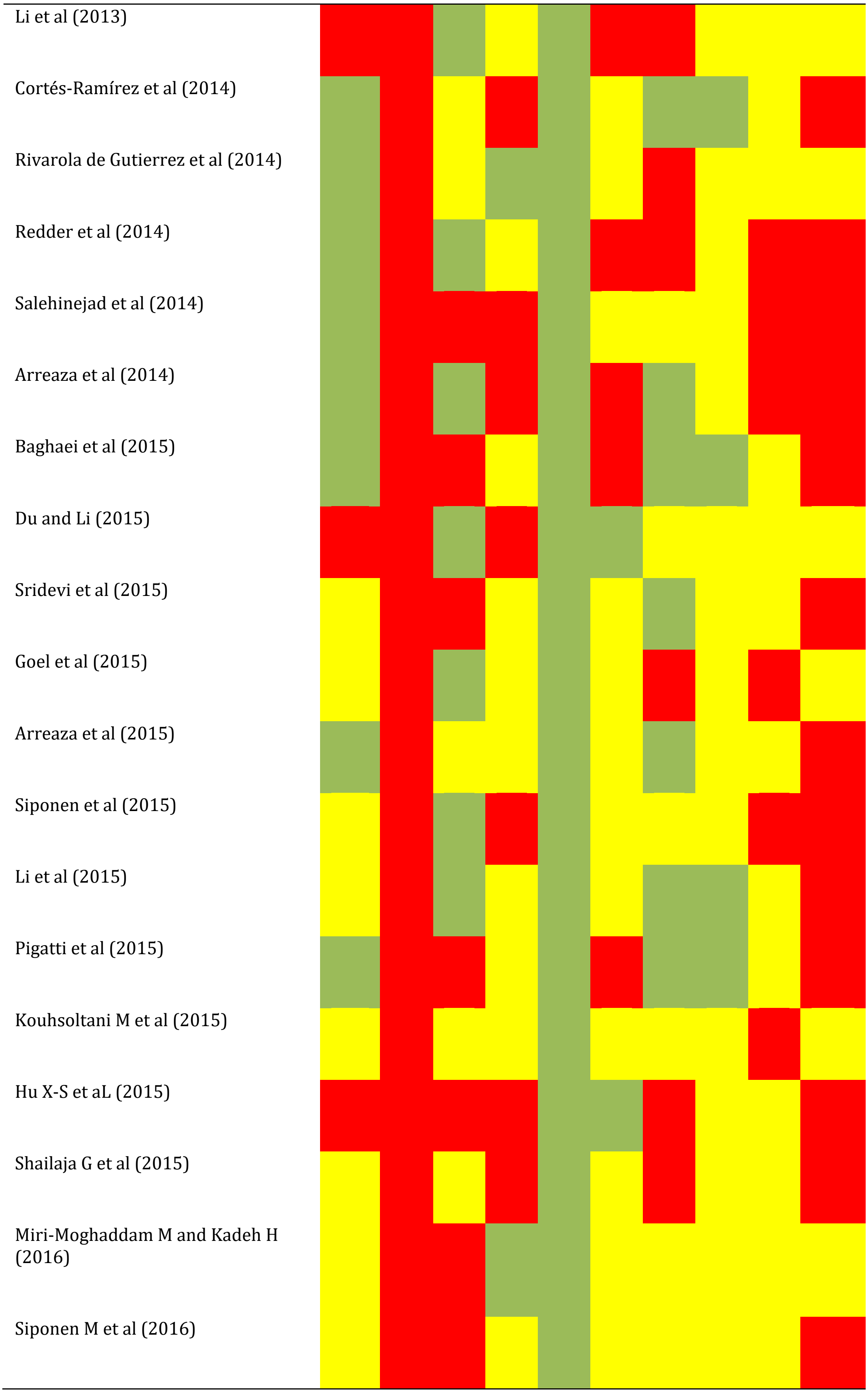
Li et al (2013)
Cortés-Ramírez et al (2014)
Rivarola de Gutierrez et al (2014)
Redder et al (2014)
Salehinejad et al (2014)
Arreaza et al (2014)
Baghaei et al (2015)
Du and Li (2015)
Sridevi et al (2015)
Goel et al (2015)
Arreaza et al (2015)
Siponen et al (2015)
Li et al (2015)
Pigatti et al (2015)
Kouhsoltani M et al (2015)
Hu X-S et aL (2015)
Shailaja G et al (2015)
Miri-Moghaddam M and Kadeh H (2016)
Siponen M et al (2016)

Liu et al (2017)										
Ghallab et al (2017)										
Basheer et al (2017)										
Hadzi-Mihailovic et al (2017)										
Bombeccari et al (2017)										
Satelur et al (2017)										
Jaafari-Ashkavandi and Aslani (2017)										
Zolfaghari Saravi et al (2017)										
Perez et al (2018)										
Zargaran et al (2018)										
Danielsson et al (2018)										
Sheelam et al (2018)										
Enomoto et al (2018)										
Zhang et al (2018)										
Shiva et al (2018)										
Agarwal et al (2019)										
Squarzanti et al (2019)										
Costa Lago et al (2020)										
Akama et al (2020)										
Kitkhajornkiat et al (2020)										

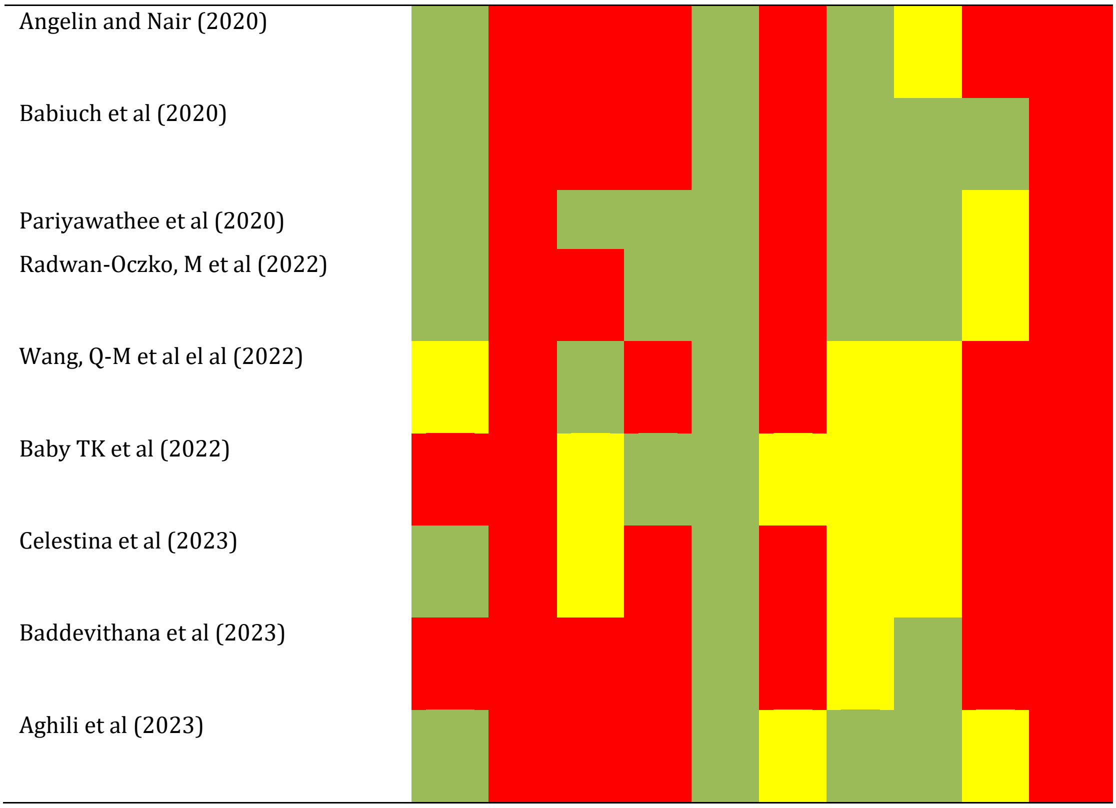
Angelin and Nair (2020)
Babiuch et al (2020)
Pariyawathee et al (2020)
Radwan-Oczko, M et al (2022)
Wang, Q-M et al el al (2022)
Baby TK et al (2022)
Celestina et al (2023)
Baddevithana et al (2023)
Aghili et al (2023)

9. Analysis of small-study effects

9.1 Hallmark 1 Sustaining proliferative signaling

Figure S35. A funnel plot of estimated transformed proportions against their standard errors, graphically representing the analysis of "small-study" effects on the differential expression of the hallmark sustaining proliferative signaling among OLP patients.

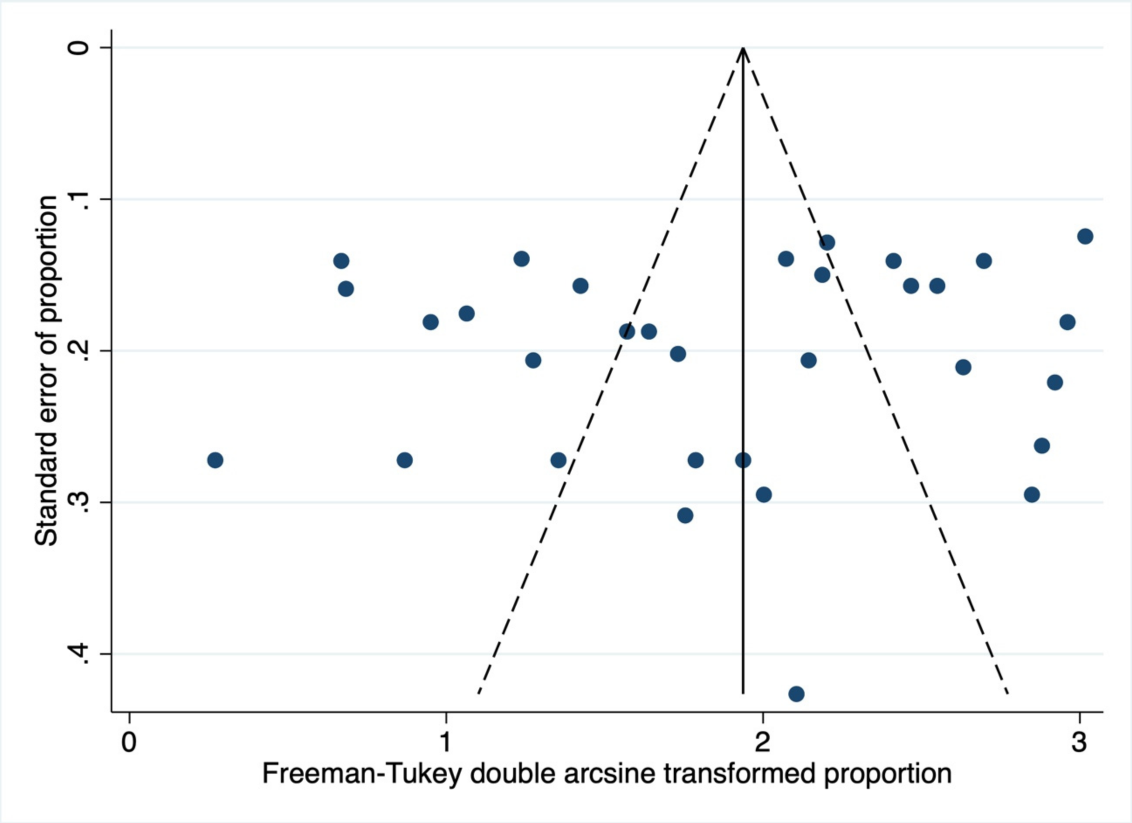

The black vertical line corresponds to the pooled estimated transformed prevalence. The two diagonal intermittent lines represent the pseudo-95% confidence interval. The blue circles represent the published studies.

9.2 Hallmark 2 Evading growth suppressors

Figure S36. A funnel plot of estimated transformed proportions against their standard errors, graphically representing the analysis of "small-study" effects on the differential expression of the hallmark evading growth suppressors among OLP patients.

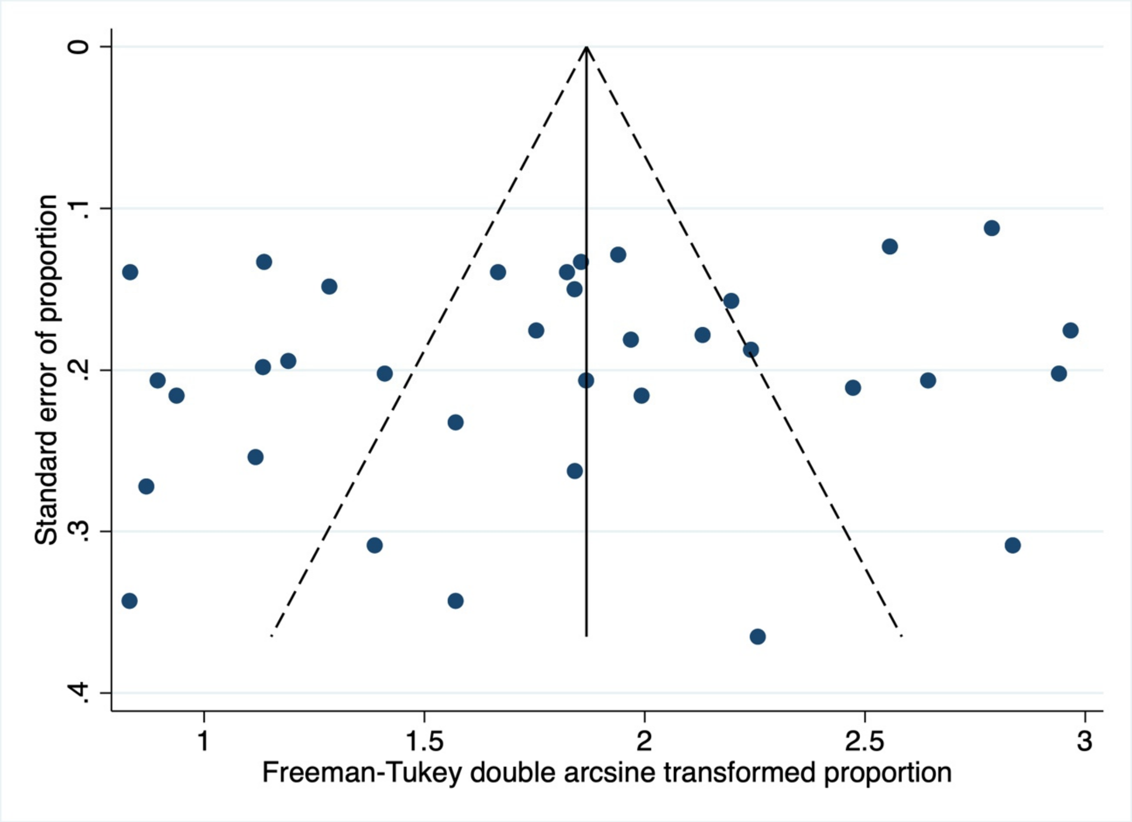

The black vertical line corresponds to the pooled estimated transformed prevalence. The two diagonal intermittent lines represent the pseudo-95% confidence interval. The blue circles represent the published studies.

9.3 Hallmark 3 anti-apoptotic biomarkers

Figure S37. A funnel plot of estimated transformed proportions against their standard errors, graphically representing the analysis of “small-study” effects on the differential expression of the antiapoptotic biomarkers among OLP patients.

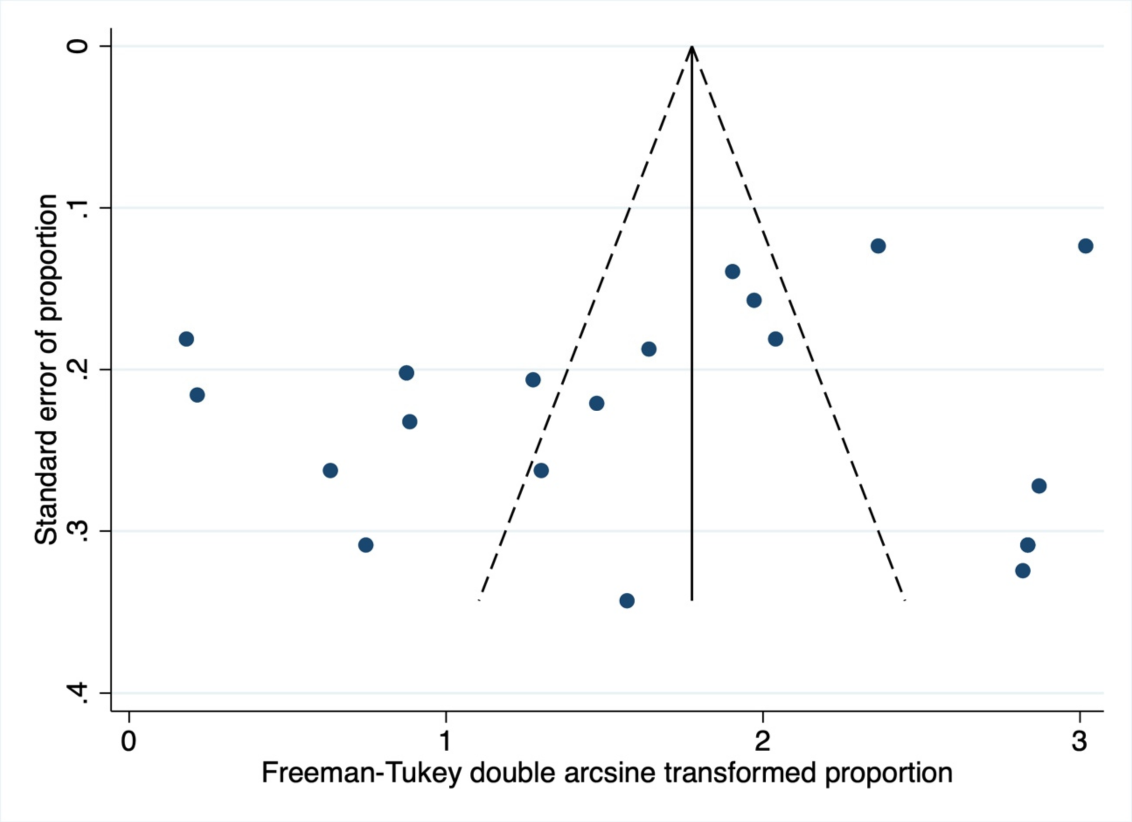

The black vertical line corresponds to the pooled estimated transformed prevalence. The two diagonal intermittent lines represent the pseudo-95% confidence interval. The blue circles represent the published studies.

9.4 Hallmark 3 pro-apoptotic biomarkers

Figure S38. A funnel plot of estimated transformed proportions against their standard errors, graphically representing the analysis of "small-study" effects on the differential expression of the anti-apoptotic biomarkers among OLP patients.

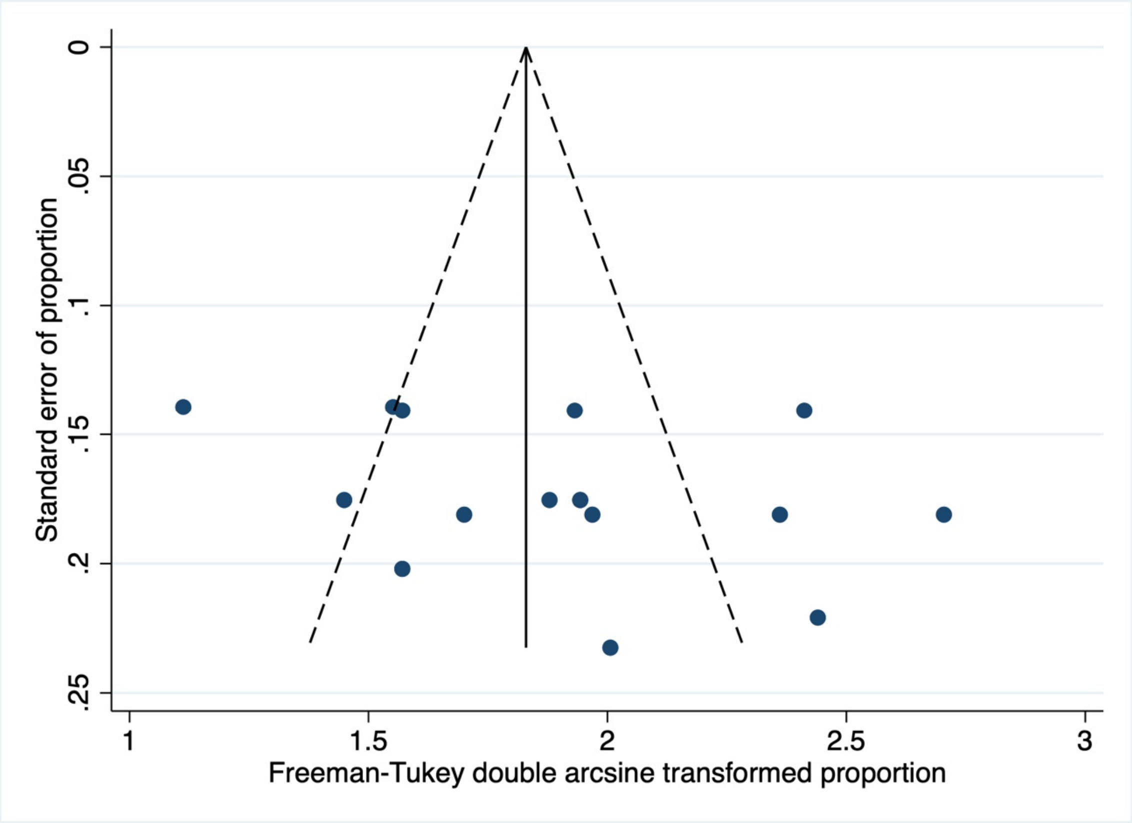

The black vertical line corresponds to the pooled estimated transformed prevalence. The two diagonal intermittent lines represent the pseudo-95% confidence interval. The blue circles represent the published studies.

9.5 Hallmark 6 activating invasion and metastasis

Figure S39. A funnel plot of estimated transformed proportions against their standard errors, graphically representing the analysis of "small-study" effects on the differential expression of the hallmark **activating invasion and metastasis** among OLP patients.

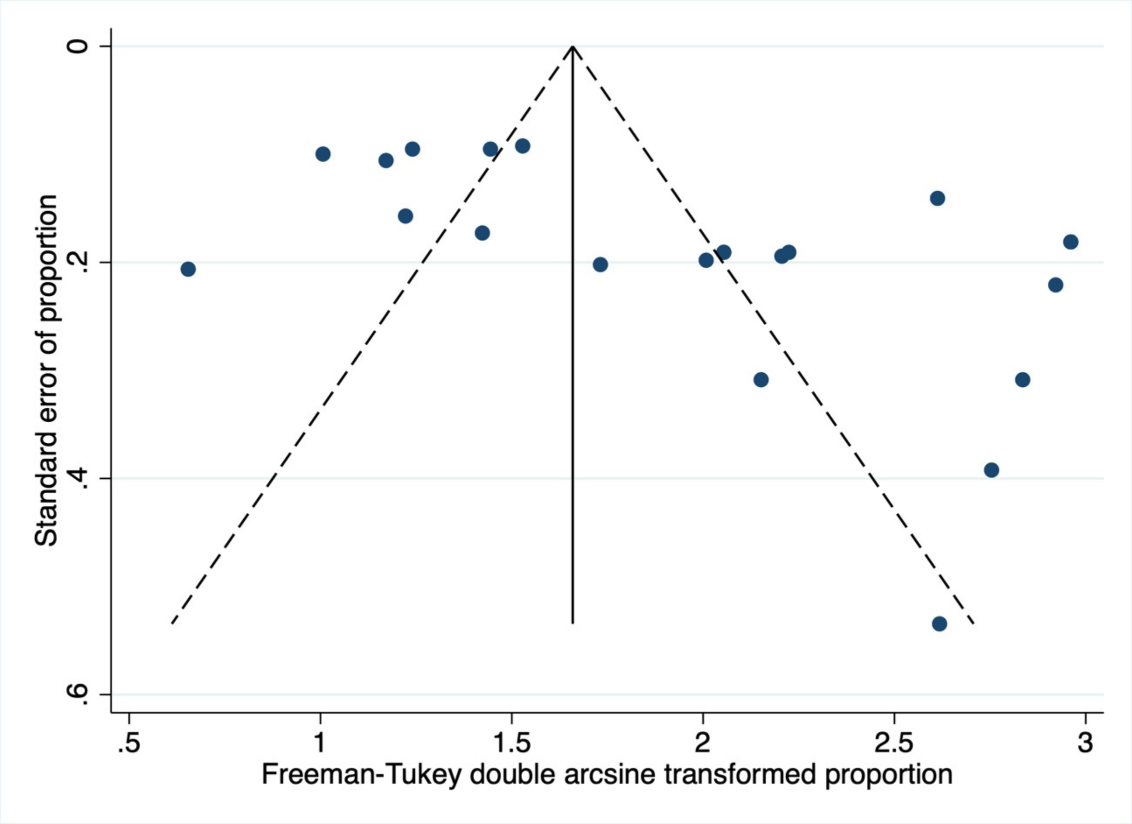

The black vertical line corresponds to the pooled estimated transformed prevalence. The two diagonal intermittent lines represent the pseudo-95% confidence interval. The blue circles represent the published studies.

9.6 Hallmark 10-tumor promoting inflammation

Figure S40. A funnel plot of estimated transformed proportions against their standard errors, graphically representing the analysis of “small-study” effects on the differential expression of the hallmark tumor promoting inflammation among OLP patients.

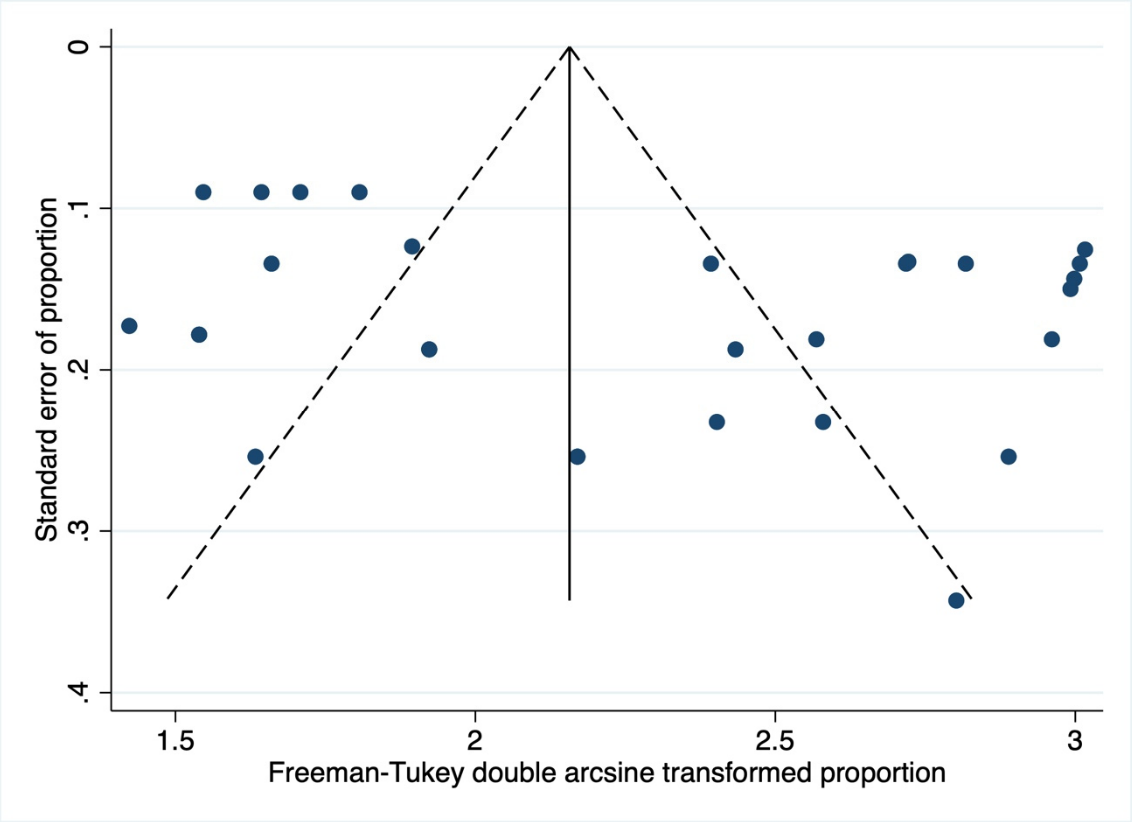

The black vertical line corresponds to the pooled estimated transformed prevalence. The two diagonal intermittent lines represent the pseudo-95% confidence interval. The blue circles represent the published studies.

10. List S1. List of included studies (n = 110).

Acay, Renata Rodrigues, Claudia Ronca Felizzola, Ney Soares de Araújo, and Suzana Orsini Machado de Sousa. 2006. “Evaluation of Proliferative Potential in Oral Lichen Planus and Oral Lichenoid Lesions Using Immunohistochemical Expression of P53 and Ki67.” *Oral Oncology* 42(5):475–80. doi: 10.1016/j.oraloncology.2005.09.012.

Agarwal, Neha, Sunitha Carnelio, and Gabriel Rodrigues. 2019. “Immunohistochemical and Clinical Significance of Matrix Metalloproteinase-2 and Its Inhibitor in Oral Lichen Planus.” *Journal of Oral and Maxillofacial Pathology : JOMFP* 23(3):476. doi: 10.4103/jomfp.JOMFP_27_19.

Aghili, Seyedeh Sara, Razieh Zare, and Alireza Jahangirnia. 2023. “Evaluation of Paxillin Expression in Epithelial Dysplasia, Oral Squamous Cell Carcinoma, Lichen Planus with and without Dysplasia, and Hyperkeratosis: A Retrospective Cross-Sectional Study.” *Diagnostics (Basel, Switzerland)* 13(15). doi: 10.3390/diagnostics13152476.

Akama, Mayara Sayuri Kamimura, Lucas Ribeiro Teixeira, Lara Maria Alencar Ramos Innocentini, Camila de Barros Gallo, Tiago Novaes Pinheiro, Alfredo Ribeiro-Silva, and Ana Carolina Fragoso Motta. 2021. “Laminin-332 Expression in Oral Lichen Planus: Preliminary Results of a Cross-Sectional Study.” *Oral Diseases* 27(4):942–46. doi: 10.1111/odi.13612.

Ali, A., J. Langdon, P. Stern, and M. Partridge. 2001. “The Pattern of Expression of the 5T4 Oncofoetal Antigen on Normal, Dysplastic and Malignant Oral Mucosa.” *Oral Oncology* 37(1):57–64. doi: 10.1016/s1368-8375(00)00057-9.

Angelin, D., and Bindu J. Nair. 2020. “Comparative Evaluation of Survivin Expression in Leukoplakia, Lichen Planus, and Oral Squamous Cell Carcinoma: An Immunohistochemical Study.” *Journal of Cancer Research and Therapeutics* 16(3):569–74. doi: 10.4103/jcrt.JCRT_421_19.

Arreaza, A., H. Rivera, and M. Correnti. 2015. “P53 Expression in Oral Lichenoid Lesions and Oral Lichen Planus.” *General Dentistry* 63(1):69–72.

Arreaza, Alven J., Helen Rivera, and María Correnti. 2014. “Expression of COX-2 and Bcl-2 in Oral Lichen Planus Lesions and Lichenoid Reactions.” *Ecancermedicalscience* 8:411. doi: 10.3332/ecancer.2014.411.

Babiuch, Karolina, Beata Kuśnierz-Cabala, Barbara Kęsek, Krzysztof Okoń, Dagmara Darczuk, and Maria Chomyszyn-Gajewska. 2020a. “Evaluation of Proinflammatory, NF-KappaB Dependent Cytokines: IL-1$α$, IL-6, IL-8, and TNF-$α$ in Tissue Specimens and Saliva of Patients with Oral Squamous Cell Carcinoma and Oral Potentially Malignant Disorders.” *Journal of Clinical Medicine* 9(3). doi: 10.3390/jcm9030867.

Babiuch, Karolina, Beata Kuśnierz-Cabala, Barbara Kęsek, Krzysztof Okoń, Dagmara Darczuk, and Maria Chomyszyn-Gajewska. 2020b. “Evaluation of Proinflammatory, Nf-Kappab Dependent Cytokines: Il-1α, Il-6, Il-8, and TNF-α in Tissue Specimens and Saliva of Patients with Oral Squamous Cell Carcinoma and Oral Potentially Malignant Disorders.”

Journal of Clinical Medicine 9(3). doi: 10.3390/jcm9030867.

Baddevithana, Asinsala K., Ruwan D. Jayasinghe, Wanninayake M. Tilakaratne, Rasika P. Illeperuma, and Bogahawatte S. M. S. Siriwardena. n.d. "Expression of Human Papillomavirus and the P16 Gene in Oral Potentially Malignant Disorders (OPMD): A Comparative Study With Oral Squamous Cell Carcinoma." *Applied Immunohistochemistry & Molecular Morphology : AIMM* 31(5):331–38. doi: 10.1097/PAI.0000000000001124.

Baghaei, Fahimeh, Setareh Shojaei, Noushin Afshar-Moghaddam, Massoumeh Zargaran, Verisheh Rastin, Mohsen Nasr, and Abbas Moghimbeigi. 2015. "Study of P21 Expression in Oral Lichen Planus and Oral Squamous Cell Carcinoma by Immunohistochemical Technique." *Journal of Dentistry (Shiraz, Iran)* 16(3):156–61.

Bascones-Ilundain, C., M. Á. González-Moles, J. Campo-Trapero, J. A. Gil-Montoya, G. C. Esparza-Gómez, J. Cano-Sánchez, and A. Bascones-Martínez. 2008. "No Differences in Caspase-3 and Bax Expression in Atrophic-erosive vs. Reticular Oral Lichen Planus." *Journal of the European Academy of Dermatology and Venereology* 22(2):204–12. doi: 10.1111/j.1468-3083.2007.02387.x.

Bascones, C., M. A. Gonzalez-Moles, G. Esparza, M. Bravo, A. Acevedo, J. A. Gil-Montoya, and A. Bascones. 2005. "Apoptosis and Cell Cycle Arrest in Oral Lichen Planus." *Archives of Oral Biology* 50(10):873–81. doi: 10.1016/j.archoralbio.2005.02.005.

Basheer, Shaini, P. M. Shameena, S. Sudha, Sujatha Varma, S. Vidyanath, and Aniruddha Varekar. 2017. "Expression of Survivin and P53 in Oral Lichen Planus, Lichenoid Reaction and Lichenoid Dysplasia: An Immunohistochemical Study." *Journal of Oral and Maxillofacial Pathology : JOMFP* 21(3):456–57. doi: 10.4103/jomfp.JOMFP_39_15.

Bloor, Balvinder K., Nicholas Tidman, Irene M. Leigh, Edward Odell, Bilal Dogan, Uwe Wollina, Lucy Ghali, and Ahmad Waseem. 2003. "Expression of Keratin K2e in Cutaneous and Oral Lesions: Association with Keratinocyte Activation, Proliferation, and Keratinization." *The American Journal of Pathology* 162(3):963–75. doi: 10.1016/S0002-9440(10)63891-6.

Bombeccari, Gian Paolo, Aldo Bruno Giannì, and Francesco Spadari. 2017. "Immunoexpression of Cytokeratin-19 in the Oral Lichen Planus and Related Oral Squamous Cell Carcinoma." *Annali Di Stomatologia* 8(3):104–9. doi: 10.11138/ads/2017.8.3.104.

Brunotto, Mabel, Ana María Zárate, Adriana Cismondi, María del Carmen Fernández, and Rita Inés Noher de Halac. 2005. "Valuation of Exfoliative Cytology as Prediction Factor in Oral Mucosa Lesions." *Medicina Oral, Patologia Oral y Cirugia Bucal* 10 Suppl 2:E92--102.

Chen, Yu, Weiping Zhang, Ning Geng, Kun Tian, and Lester Jack Windsor. 2008. "MMPs, TIMP-2, and TGF-$β$1 in the Cancerization of Oral Lichen Planus." *Head & Neck* 30(9):1237–45. doi: 10.1002/hed.20869.

Cortes-Ramirez, D. A., M. J. Rodriguez-Tojo, J. C. Coca-Meneses, X. Marichalar-Mendia, and J. M. Aguirre-Urizar. 2014. "Epidermal Growth Factor Receptor Expression in Different Subtypes of Oral Lichenoid Disease." *Medicina Oral Patología Oral y Cirugia Bucal* e451--

e458. doi: 10.4317/medoral.19452.

Cortés-Ramírez, Dionisio A., María J. Rodríguez-Tojo, María L. Gainza-Cirauqui, Rafael Martínez-Conde, and José M. Aguirre-Urizar. 2010. “Overexpression of Cyclooxygenase-2 as a Biomarker in Different Subtypes of the Oral Lichenoid Disease.” *Oral Surgery, Oral Medicine, Oral Pathology, Oral Radiology, and Endodontology* 110(6):738–43. doi: 10.1016/j.tripleo.2010.08.005.

Costa, Nádia Lago, Julie Ane Maria Gonçalves, Sara Lia Gonçalves de Lima, José Alcides Almeida de Arruda, Ana Carolina Campos Miranda, Ricardo Alves Mesquita, Éricka Janine Dantas da Silveira, and Aline Carvalho Batista. 2020. “Evaluation of PD-L1, PD-L2, PD-1 and Cytotoxic Immune Response in Oral Lichen Planus.” *Oral Diseases* 26(6):1246–54. doi: 10.1111/odi.13344.

Danielsson, K., M. Ebrahimi, Y. B. Wahlin, K. Nylander, and L. Boldrup. 2012. “Increased Levels of COX-2 in Oral Lichen Planus Supports an Autoimmune Cause of the Disease.” *Journal of the European Academy of Dermatology and Venereology : JEADV* 26(11):1415–19. doi: 10.1111/j.1468-3083.2011.04306.x.

Danielsson, K., J. Olah, R. Zohori-Zangeneh, E. Nylander, and M. Ebrahimi. 2018. “Increased Expression of P16 in Both Oral and Genital Lichen Planus.” *Medicina Oral, Patologia Oral y Cirugia Bucal* 23(4):e449--e453. doi: 10.4317/medoral.22432.

Danielsson, K., Y. B. Wahlin, P. J. Coates, and K. Nylander. 2010. “Increased Expression of Smad Proteins, and in Particular Smad3, in Oral Lichen Planus Compared to Normal Oral Mucosa.” *Journal of Oral Pathology & Medicine : Official Publication of the International Association of Oral Pathologists and the American Academy of Oral Pathology* 39(8):639–44. doi: 10.1111/j.1600-0714.2010.00902.x.

Du, Guan-Huan, Xiao-Peng Qin, Qin Li, Yong-Mei Zhou, Xue-Min Shen, and Guo-Yao Tang. 2011. “The High Expression Level of Programmed Death-1 Ligand 2 in Oral Lichen Planus and the Possible Costimulatory Effect on Human T Cells.” *Journal of Oral Pathology & Medicine* 40(7):525–32. doi: 10.1111/j.1600-0714.2011.01035.x.

DU, YONG, and HAOBO LI. 2015. “Expression of E-Cadherin in Oral Lichen Planus.” *Experimental and Therapeutic Medicine* 10(4):1544–48. doi: 10.3892/etm.2015.2654.

Enomoto, Ai, Eiichi Sato, Takashi Yasuda, Tatsuya Isomura, Toshitaka Nagao, and Daichi Chikazu. 2018. “Intraepithelial CD8+ Lymphocytes as a Predictive Diagnostic Biomarker for the Remission of Oral Lichen Planus.” *Human Pathology* 74:43–53. doi: 10.1016/j.humpath.2017.12.008.

Fan, Yuan, Zhen Zhan, Tao Peng, Xiao-ling Song, and Zhen-qing Feng. 2004. “[The Expression of Apoptosis-Associated Proteins Bcl-2, Bax in Oral Leukoplakia and Lichen Planus].” *Shanghai Kou Qiang Yi Xue = Shanghai Journal of Stomatology* 13(6):497–501.

Favia, G., M. Corsalini, M. Iacobellis, and E. Maiorano. 1994. “[Squamous Cell Carcinoma in Oral Lichen Ruber Planus. A Clinico-Pathological and Immunohistochemical Study of 11

Cases].” *Minerva Stomatologica* 43(10):479–91.

Ghallab, Noha A., Rehab Fawzy Kasem, Safa Fathy Abd El-Ghani, and Olfat G. Shaker. 2017. “Gene Expression of MiRNA-138 and Cyclin D1 in Oral Lichen Planus.” *Clinical Oral Investigations* 21(8):2481–91. doi: 10.1007/s00784-017-2091-5.

Girod, S. C., D. Cesarz, U. Fischer, and G. R. Krueger. 1995. “Detection of P53 and MDM2 Protein Expression in Head and Neck Carcinogenesis.” *Anticancer Research* 15(4):1453–57.

Girod, S. C., H. D. Pape, and G. R. Krueger. 1994. “P53 and PCNA Expression in Carcinogenesis of the Oropharyngeal Mucosa.” *European Journal of Cancer. Part B, Oral Oncology* 30B(6):419–23. doi: 10.1016/0964-1955(94)90023-x.

Girod, S. C., P. Pfeiffer, J. Ries, and H. D. Pape. 1998. “Proliferative Activity and Loss of Function of Tumour Suppressor Genes as ‘biomarkers’ in Diagnosis and Prognosis of Benign and Preneoplastic Oral Lesions and Oral Squamous Cell Carcinoma.” *The British Journal of Oral & Maxillofacial Surgery* 36(4):252–60. doi: 10.1016/s0266-4356(98)90708-2.

Goel, Sinny, Nita Khurana, Akanksha Marvah, and Sunita Gupta. 2015. “Expression of Cdk4 and P16 in Oral Lichen Planus.” *Journal of Oral and Maxillofacial Research* 6(2). doi: 10.5037/jomr.2015.6204.

González-Moles, M. A., C. Bascones-Ilundain, J. A. Gil Montoya, I. Ruiz-Avila, M. Delgado-Rodríguez, and A. Bascones-Martínez. 2006. “Cell Cycle Regulating Mechanisms in Oral Lichen Planus: Molecular Bases in Epithelium Predisposed to Malignant Transformation.” *Archives of Oral Biology* 51(12):1093–1103. doi: 10.1016/j.archoralbio.2006.06.007.

Gonzalez-Moles, M. A., J. A. Gil-Montoya, I. Ruiz-Avila, F. Esteban, and A. Bascones-Martinez. 2008. “Differences in the Expression of P53 Protein in Oral Lichen Planus Based on the Use of Monoclonal Antibodies DO7 and PAb 240.” *Oral Oncology* 44(5):496–503. doi: 10.1016/j.oraloncology.2007.06.013.

González Moles, M. A., F. Esteban, I. Ruiz-Ávila, J. A. Gil Montoya, S. Brener, A. Bascones-Martínez, and M. Muñoz. 2009. “A Role for the Substance P/NK-1 Receptor Complex in Cell Proliferation and Apoptosis in Oral Lichen Planus.” *Oral Diseases* 15(2):162–69. doi: 10.1111/j.1601-0825.2008.01504.x.

Hadzi-Mihailovic, M., H. Raybaud, R. Monteil, S. Cakic, M. Djuric, and L. Jankovic. 2010. “Bcl-2 Expression and Its Possible Influence on Malignant Transformation of Oral Lichen Planus.” *Journal of B.U.ON. : Official Journal of the Balkan Union of Oncology* 15(2):362–68.

Hadzi-Mihailovic, Milos, Renata Petrovic, Helene Raybaud, Dragan Stanimirovic, and Meltem Ozar Koray. 2017. “Expression and Role of P53 in Oral Lichen Planus Patients.” *Journal of B.U.ON. : Official Journal of the Balkan Union of Oncology* 22(5):1278–86.

Hsieh, Pi-Chuan, Yuk-Kwan Chen, Kun-Bow Tsai, Tien-Yu Shieh, Yong-Yuan Chang, Jan-Gowth Chang, Hsin-Lung Wu, and Sheng-Fung Lin. 2010. “Expression of BUBR1 in Human Oral Potentially Malignant Disorders and Squamous Cell Carcinoma.” *Oral Surgery, Oral*

Medicine, Oral Pathology, Oral Radiology, and Endodontology 109(2):257–67. doi: 10.1016/j.tripleo.2009.08.014.

Hu, X. S., Y. H. Huang, X. S. Liu, and H. Hua. 2016. “[Expression and Significance of P38 Mitogen-Activated Protein Kinase in Oral Lichen Planus and Oral Squamous Cell Cacinoma].” *Beijing Da Xue Xue Bao. Yi Xue Ban = Journal of Peking University. Health Sciences* 48(2):310–15.

Jaafari-Ashkavandi, Zohreh, and Ehsan Aslani. 2017. “Caveolin-1 Expression in Oral Lichen Planus, Dysplastic Lesions and Squamous Cell Carcinoma.” *Pathology, Research and Practice* 213(7):809–14. doi: 10.1016/j.prp.2017.03.006.

Jacques, Claudio M. C., Ana L. C. Pereira, Vivian Maia, Tullia Cuzzi, and Marcia Ramos-e-Silva. 2009. “Expression of Cytokeratins 10, 13, 14 and 19 in Oral Lichen Planus.” *Journal of Oral Science* 51(3):355–65. doi: 10.2334/josnusd.51.355.

Kilpi, A., A. M. Rich, P. C. Reade, and Y. T. Konttinen. 1996. “Studies of the Inflammatory Process and Malignant Potential of Oral Mucosal Lichen Planus.” *Australian Dental Journal* 41(2):87–90. doi: 10.1111/j.1834-7819.1996.tb05919.x.

Kitkhajornkiat, Athip, Sorasun Rungsiyanont, Sineepat Talungchit, Pimporn Jirawechwongsakul, and Patrayu Taebunpakul. 2020. “The Expression of Cathepsin L in Oral Lichen Planus.” *Journal of Oral Biology and Craniofacial Research* 10(3):281–86. doi: 10.1016/j.jobcr.2020.06.003.

Kłosek, Sebastian K., Stanisław Sporny, Olga Stasikowska-Kanicka, and Anna J. Kurnatowska. 2011. “Cigarette Smoking Induces Overexpression of C-Met Receptor in Microvessels of Oral Lichen Planus.” *Archives of Medical Science* 4:706–12. doi: 10.5114/aoms.2011.24143.

Kouhsoltani, Maryam, Amirala Aghbali, Behrooz Shokoohi, and Ronak Ahmadzadeh. 2015. “Molecular Targeting of Her-2/Neu Protein Is Not Recommended as an Adjuvant Therapy in Oral Squamous Cell Carcinoma and Oral Lichen Planus.” *Advanced Pharmaceutical Bulletin* 5(Suppl 1):649–52. doi: 10.15171/apb.2015.088.

Krauss, Eva, Stephan Rauthe, Stefan Gattenlöhner, Tobias Reuther, Michael Kochel, Ulrike Kriegebaum, Alexander C. Kübler, and Urs D. A. Müller-Richter. 2011. “MAGE-A Antigens in Lesions of the Oral Mucosa.” *Clinical Oral Investigations* 15(3):315–20. doi: 10.1007/s00784-010-0387-9.

Lee, Jang-Jaer, Mark-Yenping Kuo, Shi-Jung Cheng, Chun-Pin Chiang, Jiiang-Huei Jeng, Hao-Hueng Chang, Ying-Shiung Kuo, Wan-Hong Lan, and Sang-Heng Kok. 2005. “Higher Expressions of P53 and Proliferating Cell Nuclear Antigen (PCNA) in Atrophic Oral Lichen Planus and Patients with Areca Quid Chewing.” *Oral Surgery, Oral Medicine, Oral Pathology, Oral Radiology, and Endodontics* 99(4):471–78. doi: 10.1016/j.tripleo.2004.10.018.

Leyva-Huerta, Elba-Rosa, Constantino Ledesma-Montes, Rebeca-Estela Rojo-Botello, and Elisa

Vega-Memije. 2012. “P53 and Bcl-2 Immunoexpression in Patients with Oral Lichen Planus and Oral Squamous Cell Carcinoma.” *Medicina Oral, Patologia Oral y Cirugia Bucal* 17(5):e745--50. doi: 10.4317/medoral.18013.

Li, Hao-Bo, Ying-Huai Zhang, Hui-Zhen Chen, and Yong Chen. 2015. “Expression of Human DNA Mismatch-Repair Protein, HMSH2, in Patients with Oral Lichen Planus.” *Experimental and Therapeutic Medicine* 9(1):203–6. doi: 10.3892/etm.2014.2053.

Li, Ning, Qiong Hu, Canhua Jiang, Feng Guo, Krishna Munnee, Xinchun Jian, Yanjia Hu, and Zhangui Tang. 2013. “Cys-X-Cys Ligand 9 Might Be an Immunological Factor in the Pathogenesis of Oral Submucous Fibrosis and Its Concomitant Oral Lichenoid Lesion.” *Clinical Oral Investigations* 17(4):1251–58. doi: 10.1007/s00784-012-0799-9.

Li, Tie-Jun, and Jun Cui. 2013. “COX-2, MMP-7 Expression in Oral Lichen Planus and Oral Squamous Cell Carcinoma.” *Asian Pacific Journal of Tropical Medicine* 6(8):640–43. doi: 10.1016/S1995-7645(13)60110-8.

Liu, Yuan, Guicai Liu, Qing Liu, Jun Tan, Xin Hu, Jinjin Wang, Qintao Wang, and Xinwen Wang. 2017. “The Cellular Character of Liquefaction Degeneration in Oral Lichen Planus and the Role of Interferon Gamma.” *Journal of Oral Pathology & Medicine : Official Publication of the International Association of Oral Pathologists and the American Academy of Oral Pathology* 46(10):1015–22. doi: 10.1111/jop.12595.

Ma, Lihua, Hao Wang, Hui Yao, Laikuan Zhu, Wei Liu, and Zengtong Zhou. 2013. “Bmi1 Expression in Oral Lichen Planus and the Risk of Progression to Oral Squamous Cell Carcinoma.” *Annals of Diagnostic Pathology* 17(4):327–30. doi: 10.1016/j.anndiagpath.2013.03.002.

Martín-Ezquerra, G., R. Salgado, A. Toll, M. Gilaberte, T. Baró, F. Alameda Quitllet, M. Yébenes, F. Solé, M. Garcia-Muret, B. Espinet, and R. M. Pujol. 2010. “Multiple Genetic Copy Number Alterations in Oral Squamous Cell Carcinoma: Study of MYC , TP53 , CCDN1, EGFR and ERBB2 Status in Primary and Metastatic Tumours.” *British Journal of Dermatology* 163(5):1028–35. doi: 10.1111/j.1365-2133.2010.09947.x.

Martín-Ezquerra, Gemma, Rocío Salgado, Agustí Toll, Teresa Baró, Sergi Mojal, Mireia Yébenes, Ma Pilar Garcia-Muret, Francesc Solé, Francesc Alameda Quitllet, Blanca Espinet, and Ramon M. Pujol. 2011. “CDC28 Protein Kinase Regulatory Subunit 1B (CKS1B) Expression and Genetic Status Analysis in Oral Squamous Cell Carcinoma.” *Histology and Histopathology* 26(1):71–77. doi: 10.14670/HH-26.71.

Martínez-Lara, I., M. A. González-Moles, I. Ruiz-Avila, M. Bravo, M. C. Ramos, and J. A. Fernández-Martínez. 1996. “Proliferating Cell Nuclear Antigen (PCNA) as a Marker of Dysplasia in Oral Mucosa.” *Acta Stomatologica Belgica* 93(1):29–32.

Miri-Moghaddam, Masoud, and Hamideh Kadeh. 2016. “Immunohistochemical Expression of Stromelysin-2 (St-2) In Patients with Oral Lichen Planus and Its Clinical Significance.” *Journal of Dentistry (Shiraz, Iran)* 17(3 Suppl):250–55.

Montebugnoli, L., M. Venturi, D. B. Gissi, E. Leonardi, A. Farnedi, and Maria P. Foschini. 2011. "Immunohistochemical Expression of P16INK4A Protein in Oral Lichen Planus." *Oral Surgery, Oral Medicine, Oral Pathology, Oral Radiology, and Endodontology* 112(2):222–27. doi: 10.1016/j.tripleo.2011.02.029.

Muraki, Y., C. Yoshioka, J. Fukuda, T. Haneji, and N. Kobayashi. 1997. "Immunohistochemical Detection of Fas Antigen in Oral Epithelia." *Journal of Oral Pathology & Medicine : Official Publication of the International Association of Oral Pathologists and the American Academy of Oral Pathology* 26(2):57–62. doi: 10.1111/j.1600-0714.1997.tb00022.x.

Nafarzadeh, Shima, Samad Ejtehadi, Pouyan Amini Shakib, Majid Fereidooni, and Ali Bijani. 2013. "Comparative Study of Expression of Smad3 in Oral Lichen Planus and Normal Oral Mucosa." *International Journal of Molecular and Cellular Medicine* 2(4):194–98.

Nafarzadeh, Shima, Sina Jafari, and Ali Bijani. 2013. "Assessment of Bax and Bcl-2 Immunoexpression in Patients with Oral Lichen Planus and Oral Squamous Cell Carcinoma." *International Journal of Molecular and Cellular Medicine* 2(3):136–42.

Nagao, Y., M. Sata, M. Kage, T. Kameyama, and T. Ueno. 2000. "Histopathological and Immunohistochemical Study of Oral Lichen Planus-Associated HCV Infection." *European Journal of Internal Medicine* 11(5):277–82. doi: 10.1016/s0953-6205(00)00107-2.

Neppelberg, Evelyn, and Anne Christine Johannessen. 2007. "DNA Content, Cyclooxygenase-2 Expression and Loss of E-Cadherin Expression Do Not Predict Risk of Malignant Transformation in Oral Lichen Planus." *European Archives of Oto-Rhino-Laryngology* 264(10):1223–30. doi: 10.1007/s00405-007-0346-5.

Ögmundsdóttir, H. M., J. Björnsson, and W. P. Holbrook. 2009. "Role of TP53 in the Progression of Pre-malignant and Malignant Oral Mucosal Lesions. A Follow-up Study of 144 Patients." *Journal of Oral Pathology & Medicine* 38(7):565–71. doi: 10.1111/j.1600-0714.2009.00766.x.

Ögmundsdóttir, H. M., H. Hilmarsdóttir, J. Björnsson, and W. P. Holbrook. 2009. "Longitudinal Study of TP53 Mutations in Eight Patients with Potentially Malignant Oral Mucosal Disorders." *Journal of Oral Pathology & Medicine* 38(9):716–21. doi: 10.1111/j.1600-0714.2009.00767.x.

Oliveira Alves, Mg, I. Balducci, Y. Rodarte Carvalho, Lag Cabral, Fd Nunes, and Jd Almeida. 2013. "Evaluation of the Expression of P53, MDM2, and SUMO-1 in Oral Lichen Planus." *Oral Diseases* 19(8):775–80. doi: 10.1111/odi.12068.

Oluwadara, Oluwadayo, Luca Giacomelli, Russell Christensen, George Kossan, Raisa Avezova, and Francesco Chiappelli. 2009. "LCK, Survivin and PI-3K in the Molecular Biomarker Profiling of Oral Lichen Planus and Oral Squamous Cell Carcinoma." *Bioinformation* 4(6):249–57. doi: 10.6026/97320630004248.

Pariyawathee, Sarinthon, Ekarat Phattarataratip, and Kobkan Thongprasom. 2020. "CD146 Expression in Oral Lichen Planus and Oral Cancer." *Clinical Oral Investigations* 24(1):325–

32. doi: 10.1007/s00784-019-02871-7.

Pérez, Miguel Ángel, Mariana Silvia Gandolfo, Patricia Masquijo Bisio, María Luisa Paparella, and María Elina Itoiz. 2018a. “Different Expression Patterns of Carbonic Anhydrase IX in Oral Lichen Planus and Leukoplakia.” *Acta Odontologica Latinoamericana : AOL* 31(2):77–81.

Pérez, Miguel Ángel, Mariana Silvia Gandolfo, Patricia Masquijo Bisio, María Luisa Paparella, and María Elina Itoiz. 2018b. “Different Expression Patterns of Carbonic Anhydrase IX in Oral Lichen Planus and Leukoplakia.” *Acta Odontologica Latinoamericana : AOL* 31(2):77–81.

Peter, Celestina D., R. Shashidara, Vipin Jain, Vanishree C. Haragannavar, Pradeep Samuel, and Suhas R. Nayak. 2023. “Senescence in Oral Lichen Planus as Assessed by the Immunohistochemical Evaluation of Senescence Marker Protein-30 (Regucalcin).” *Indian Journal of Pathology & Microbiology* 66(1):9–13. doi: 10.4103/ijpm.ijpm_864_21.

Pigatti, Fernanda Mombrini, Luís Antônio de Assis Taveira, and Cléverson Teixeira Soares. 2015. “Immunohistochemical Expression of Bcl-2 and Ki-67 in Oral Lichen Planus and Leukoplakia with Different Degrees of Dysplasia.” *International Journal of Dermatology* 54(2):150–55. doi: 10.1111/ijd.12279.

Pirkić, Ahmed, Dolores Biocina-Lukenda, Ana Cekić-Arambasin, Dino Buković, Ljubomir Pavelić, and Sime Sakić. 2004. “Changes in the Tissue Expression of the C-ErbB-2 Oncogen in the Oral Lichen Ruber.” *Collegium Antropologicum* 28(1):455–61.

Poomsawat, Sopee, Waranun Buajeeb, Siribang-on Piboonniyom Khovidhunkit, and Jirapa Punyasingh. 2011. “Overexpression of Cdk4 and P16 in Oral Lichen Planus Supports the Concept of Premalignancy.” *Journal of Oral Pathology & Medicine* 40(4):294–99. doi: 10.1111/j.1600-0714.2010.01001.x.

Prodromidis, Georgios, Nikolaos G. Nikitakis, and Alexandra Sklavounou. 2013. “Immunohistochemical Analysis of the Activation Status of the Akt/MTOR/PS6 Signaling Pathway in Oral Lichen Planus.” *International Journal of Dentistry* 2013:743456. doi: 10.1155/2013/743456.

Radwan-Oczko, Malgorzata, Julia Bar, Agnieszka Hałoń, and Anna Lis-Nawara. 2022. “Comparison of Biomarker Expression in Oral Lichen Planus and Oral Lichenoid Lesions.” *Advances in Clinical and Experimental Medicine* 31(12):1327–34. doi: 10.17219/acem/152429.

Redder, C. Pramod, Siddharth Pandit, Dinkar Desai, V. Suresh Kandagal, Pramod S. Ingaleshwar, Sharan J. Shetty, and Nupura Vibhute. 2014. “Comparative Analysis of Cell Proliferation Ratio in Plaque and Erosive Oral Lichen Planus: An Immunohistochemical Study.” *Dental Research Journal* 11(3):316–20.

Rivarola de Gutierrez, E., A. C. Innocenti, M. J. Cippitelli, S. Salomon, and L. M. Vargas-Roig. 2014. “Determination of Cytokeratins 1, 13 and 14 in Oral Lichen Planus.” *Medicina Oral*

Patología Oral y Cirugia Bucal e359--e365. doi: 10.4317/medoral.19289.

Salehinejad, Jahanshah, Nourieh Sharifi, Maryam Amirchaghmaghi, Narges Ghazi, Mohammad Taghi Shakeri, and Ala Ghazi. 2014. “Immunohistochemical Expression of P16 Protein in Oral Squamous Cell Carcinoma and Lichen Planus.” *Annals of Diagnostic Pathology* 18(4):210–13. doi: 10.1016/j.anndiagpath.2014.03.009.

Satelur, Krishnanand Prakash, Shiny Bopaiah, Radhika Manoj Bavle, and Prashant Ramachandra. 2017. “Role of Cathepsin B as a Marker of Malignant Transformation in Oral Lichen Planus: An Immunohistochemical Study.” *Journal of Clinical and Diagnostic Research : JCDR* 11(8):ZC29--ZC32. doi: 10.7860/JCDR/2017/30740.10274.

Shailaja, G., J. Vijay Kumar, P. V Baghirath, U. Kumar, G. Ashalata, and A. Bhargavi Krishna. 2015. “Estimation of Malignant Transformation Rate in Cases of Oral Epithelial Dysplasia and Lichen Planus Using Immunohistochemical Expression of Ki-67, P53, BCL-2, and BAX Markers.” *Dental Research Journal* 12(3):235–42.

Sheelam, Suchita, Shyam Prasad Reddy, Pavan G. Kulkarni, Srk Nandan, M. Keerthi, and G. Shyam Raj. 2018. “Role of Cell Proliferation and Vascularity in Malignant Transformation of Potentially Malignant Disorders.” *Journal of Oral and Maxillofacial Pathology : JOMFP* 22(2):281. doi: 10.4103/jomfp.JOMFP_182_17.

Shen, Li-jia, Ping Ruan, Fang-fang Xie, and Tong Zhao. 2004. “[Expressions of Fas/FasL and Granzyme B in Oral Lichen Planus and Their Significance].” *Di 1 Jun Yi Da Xue Xue Bao = Academic Journal of the First Medical College of PLA* 24(12):1362–66.

Shi, Peng, Wei Liu, Zeng-Tong Zhou, Qing-Bo He, and Wei-Wen Jiang. 2010. “Podoplanin and ABCG2: Malignant Transformation Risk Markers for Oral Lichen Planus.” *Cancer Epidemiology, Biomarkers & Prevention* 19(3):844–49. doi: 10.1158/1055-9965.EPI-09-0699.

Shi, Yong-jian, Li-jia Shen, and Cao Yin. 2010. “[Expression of Caspase-8, Receptor Interacting Protein and Nuclear Factor-KappaBp65 in Oral Lichen Planus].” *Zhonghua Kou Qiang Yi Xue Za Zhi = Zhonghua Kouqiang Yixue Zazhi = Chinese Journal of Stomatology* 45(1):11–15.

Shiva, Atena, Ali Zamanian, Shahin Arab, and Mahsa Boloki. 2018. “Immunohistochemical Study of P53 Expression in Patients with Erosive and Non-Erosive Oral Lichen Planus.” *Journal of Dentistry (Shiraz, Iran)* 19(2):118–23.

Siponen, Maria, Carolina Cavalcante Bitu, Ahmed Al-Samadi, Pentti Nieminen, and Tuula Salo. 2016. “Cathepsin K Expression Is Increased in Oral Lichen Planus.” *Journal of Oral Pathology & Medicine* 45(10):758–65. doi: 10.1111/jop.12446.

Siponen, Maria, Arja Kullaa, Pentti Nieminen, Tuula Salo, and Sanna Pasonen-Seppänen. 2015. “Altered Expression of Hyaluronan, HAS1-2, and HYAL1-2 in Oral Lichen Planus.” *Journal of Oral Pathology & Medicine* 44(6):401–9. doi: 10.1111/jop.12294.

de Sousa, Fernando Augusto Cervantes Garcia, Thaís Cachuté Paradella, Yasmin Rodarte

Carvalho, and Luiz Eduardo Blumer Rosa. 2009. “Comparative Analysis of the Expression of Proliferating Cell Nuclear Antigen, P53, Bax, and Bcl-2 in Oral Lichen Planus and Oral Squamous Cell Carcinoma.” *Annals of Diagnostic Pathology* 13(5):308–12. doi: 10.1016/j.anndiagpath.2009.06.001.

Squarzanti, Diletta Francesca, Tiziana Cena, Rita Sorrentino, Mario Migliario, Annalisa Chiocchetti, Lia Rimondini, Barbara Azzimonti, and Guido Valente. 2019. “Implications on Pathogenesis and Risk of Oral Lichen Planus Neoplastic Transformation: An Ex-Vivo Retrospective Immunohistochemical Study.” *Histology and Histopathology* 34(9):1015–24. doi: 10.14670/HH-18-104.

Sridevi, Ugrappa, Ajay Jain, Velpula Nagalaxmi, Ugrappa Vijay Kumar, and Stuti Goyal. 2015. “Expression of E-Cadherin in Normal Oral Mucosa, in Oral Precancerous Lesions and in Oral Carcinomas.” *European Journal of Dentistry* 9(3):364–72. doi: 10.4103/1305-7456.163238.

Sudha, V. M., and S. Hemavathy. 2011. “Role of Bcl-2 Oncoprotein in Oral Potentially Malignant Disorders and Squamous Cell Carcinoma: An Immunohistochemical Study.” *Indian Journal of Dental Research : Official Publication of Indian Society for Dental Research* 22(4):520–25. doi: 10.4103/0970-9290.90286.

Sun, Lili, Jinqiu Feng, Lihua Ma, Wei Liu, and Zengtong Zhou. 2013. “CD133 Expression in Oral Lichen Planus Correlated with the Risk for Progression to Oral Squamous Cell Carcinoma.” *Annals of Diagnostic Pathology* 17(6):486–89. doi: 10.1016/j.anndiagpath.2013.06.004.

Tanda, N., S. Mori, K. Saito, K. Ikawa, and S. Sakamoto. 2000. “Expression of Apoptotic Signaling Proteins in Leukoplakia and Oral Lichen Planus: Quantitative and Topographical Studies.” *Journal of Oral Pathology & Medicine : Official Publication of the International Association of Oral Pathologists and the American Academy of Oral Pathology* 29(8):385–93. doi: 10.1034/j.1600-0714.2000.290804.x.

Taniguchi, Y., T. Nagao, H. Maeda, Y. Kameyama, and K. A. A. S. Warnakulasuriya. 2002. “Epithelial Cell Proliferation in Oral Lichen Planus.” *Cell Proliferation* 35 Suppl 1(Suppl 1):103–9. doi: 10.1046/j.1365-2184.35.s1.11.x.

Thongprasom, Kobkan, Kittipong Dhanuthai, Wilairat Sarideechaigul, Ponlatham Chaiyarit, and Mantharop Chaimusig. 2006. “Expression of TNF-Alpha in Oral Lichen Planus Treated with Fluocinolone Acetonide 0.1%.” *Journal of Oral Pathology & Medicine : Official Publication of the International Association of Oral Pathologists and the American Academy of Oral Pathology* 35(3):161–66. doi: 10.1111/j.1600-0714.2006.00392.x.

van der Velden, L. A., J. J. Manni, F. C. Ramaekers, and W. Kuijpers. 1999. “Expression of Intermediate Filament Proteins in Benign Lesions of the Oral Mucosa.” *European Archives of Oto-Rhino-Laryngology : Official Journal of the European Federation of Oto-Rhino-Laryngological Societies (EUFOS) : Affiliated with the German Society for Oto-Rhino-Laryngology - Head and Neck Surgery* 256(10):514–19. doi: 10.1007/s004050050202.

Wang, Qing-Mei, Xue-Ying Huang, and Wei-Qun Guan. 2022. “Expressions of Interleukin-27 in

Oral Lichen Planus, Oral Leukoplakia, and Oral Squamous Cell Carcinoma." *Inflammation* 45(3):1023–38. doi: 10.1007/s10753-021-01599-5.

Xu, Ziyuan, Zhengyu Shen, Linjun Shi, Hongying Sun, Wei Liu, and Zengtong Zhou. 2013. "Aldehyde Dehydrogenase 1 Expression Correlated with Malignant Potential of Oral Lichen Planus." *Annals of Diagnostic Pathology* 17(5):408–11. doi: 10.1016/j.anndiagpath.2013.04.008.

Yadav, M., M. Arivananthan, A. Chandrashekran, B. S. Tan, and B. Y. Hashim. 1997. "Human Herpesvirus-6 (HHV-6) DNA and Virus-Encoded Antigen in Oral Lesions." *Journal of Oral Pathology & Medicine : Official Publication of the International Association of Oral Pathologists and the American Academy of Oral Pathology* 26(9):393–401. doi: 10.1111/j.1600-0714.1997.tb00238.x.

Younes, F., E. L. Quartey, S. Kiguwa, and M. Partridge. 1996. "Expression of TNF and the 55-KDa TNF Receptor in Epidermis, Oral Mucosa, Lichen Planus and Squamous Cell Carcinoma." *Oral Diseases* 2(1):25–31. doi: 10.1111/j.1601-0825.1996.tb00199.x.

Zargaran, Massoumeh, Fahimeh Baghaei, and Abbas Moghimbeigi. 2018. "Comparative Study of $β$-Catenin and CD44 Immunoexpression in Oral Lichen Planus and Squamous Cell Carcinoma." *International Journal of Dermatology* 57(7):794–98. doi: 10.1111/ijd.14007.

Zhang, L., E. Mao, R. Priddy, and M. Rosin. 1996. "P53 Overexpression in Oral Lichen Planus." *Oncology Reports* 3(6):1145–48. doi: 10.3892/or.3.6.1145.

Zhang, Zhi-Rui, Li-Ya Chen, Hong-Yan Qi, and Shao-Hua Sun. 2018. "Expression and Clinical Significance of Periostin in Oral Lichen Planus." *Experimental and Therapeutic Medicine* 15(6):5141–47. doi: 10.3892/etm.2018.6029.

Zhao, Min, Xiu-lan Fu, and Hong Lv. 2012. "[The Expression of EGFR in Oral Lichen Planus, Squamous Cell Papilloma and Squamous Cell Carcinoma]." *Shanghai Kou Qiang Yi Xue = Shanghai Journal of Stomatology* 21(6):673–76.

Zolfaghari Saravi, Zahra, Maryam Seyedmajidi, Majid Sharbatdaran, Ali Bijani, Fatemeh Mozaffari, and Pouyan Aminishakib. 2017. "VEGFR-3 Expression in Oral Lichen Planus." *Asian Pacific Journal of Cancer Prevention : APJCP* 18(2):381–84. doi: 10.22034/APJCP.2017.18.2.381.

Zyada, Manal M., and Hala E. Fikry. 2010. "Immunohistochemical Study of Syndecan-1 down-Regulation and the Expression of P35 Protein in Oral Lichen Planus: A Clinicopathologic Correlation with Hepatitis C Infection in the Egyptian Population." *Annals of Diagnostic Pathology* 14(3):153–61. doi: 10.1016/j.anndiagpath.2009.12.006.

11. List of excluded studies with reasons

11.1 List S2. Lack of essential data (n=72)

Abé, T., Kitagawa, N., Yoshimoto, S., Maruyama, S., Yamazaki, M., Inai, T., Hashimoto, S., & Saku, T. (2020). Keratin 17-positive Civatte bodies in oral lichen planus-distribution variety, diagnostic significance and histopathogenesis. *Scientific Reports*, *10*(1), 14586. https://doi.org/10.1038/s41598-020-71496-8

Aghbari, S. M. H., Abushouk, A. I., Shakir, O. G., Zayed, S. O., & Attia, A. (2018). Correlation between tissue expression of microRNA-137 and CD8 in oral lichen planus. *Clinical Oral Investigations*, *22*(3), 1463–1467. https://doi.org/10.1007/s00784-017-2252-6

Aliev, A. D., Mikhailovskii, V. M., Perlamutrov, Y. N., & Kushlinskii, N. E. (2007). Soluble Fas antigen in the serum of women with oral lichen planus. *Bulletin of Experimental Biology and Medicine*, *143*(6), 727–729. https://doi.org/10.1007/s10517-007-0225-5

Alrashdan, M. S., Angel, C., Cirillo, N., & McCullough, M. (2016). Smoking habits and clinical patterns can alter the inflammatory infiltrate in oral lichenoid lesions. *Oral Surgery, Oral Medicine, Oral Pathology and Oral Radiology*, *121*(1), 49–57. https://doi.org/10.1016/j.oooo.2015.08.020

Amin, N. R., Yussif, N., & Ahmed, E. (2020). The effect of smoking on clinical presentation and expression of TLR-2 and CD34 in Oral lichen Planus patients: clinical and immunohistochemical study. *BMC Oral Health*, *20*(1), 129. https://doi.org/10.1186/s12903-020-01118-2

Bascones-Ilundain, C., González-Moles, M. A., Campo-Trapero, J., Gil-Montoya, J. A., Esparza-Gómez, G. C., Cano-Sánchez, J., & Bascones-Martínez, A. (2008). No differences in caspase-3 and Bax expression in atrophic-erosive vs. reticular oral lichen planus. *Journal of the European Academy of Dermatology and Venereology : JEADV*, *22*(2), 204–212. https://doi.org/10.1111/j.1468-3083.2007.02387.x

Beevi, B. H., Nayak, S. R., Peter, C. D., Haridas, A. K., Jacob, L., & Aboobakker, A. (2019). Analysis of Ki-67 Expression in Oral Premalignant Lesions and Normal Oral Mucosa: An Immunohistochemical Study. *Journal of Pharmacy & Bioallied Sciences*, *11*(Suppl 2), S232–S235. https://doi.org/10.4103/JPBS.JPBS_305_18

Boisnic, S., Ouhayoun, J. P., Branchet, M. C., Frances, C., Béranger, J. Y., Le Charpentier, Y., & Szpirglas, H. (1995). Alteration of cytokeratin expression in oral lichen planus. *Oral Surgery, Oral Medicine, Oral Pathology, Oral Radiology, and Endodontics*, *79*(2), 207–215. https://doi.org/10.1016/s1079-2104(05)80283-5

Bruno, E., Alessandrini, M., Russo, S., D'Erme, G., Nucci, R., & Calabretta, F. (2002). Malignant degeneration of oral lichen planus: our clinical experience and review of the literature. *Anales Otorrinolaringologicos Ibero-Americanos*, *29*(4), 349–357. http://www.ncbi.nlm.nih.gov/pubmed/12462928

Costa, N. L., Gonçalves, A. S., Souza-Lima, N. C., Jaime-Paiva, L. G., Junqueira-Kipnis, A. P., Silva, T. A., Mendonça, E. F., & Batista, A. C. (2011). Distinct expression of perforin and granzyme B in lip and oral cavity squamous cell carcinoma. *Journal of Oral Pathology & Medicine : Official Publication of the International Association of Oral Pathologists and the American Academy of Oral Pathology*, *40*(5), 380–384. https://doi.org/10.1111/j.1600-0714.2011.01014.x

Cuevas-Nunez, M. C., Gomes, C. B. F., Woo, S.-B., Ramsey, M. R., Chen, X. L., Xu, S., Xu, T., Zhan, Q., Murphy, G. F., & Lian, C. G. (2018). Biological significance of 5-hydroxymethylcytosine in oral epithelial dysplasia and oral squamous cell carcinoma. *Oral Surgery, Oral Medicine, Oral Pathology and Oral Radiology*, *125*(1), 59-73.e2. https://doi.org/10.1016/j.oooo.2017.06.006

DeAngelis, L. M., Cirillo, N., Perez-Gonzalez, A., & McCullough, M. (2023). Characterization of Mucosal-Associated Invariant T Cells in Oral Lichen Planus. *International Journal of Molecular Sciences*, *24*(2). https://doi.org/10.3390/ijms24021490

Du, G., Chen, J., Wang, Y., Cao, T., Zhou, L., Wang, Y., Han, X., & Tang, G. (2018). Differential expression of STAT-3 in subtypes of oral lichen planus: a preliminary study. *Oral Surgery, Oral Medicine, Oral Pathology and Oral Radiology*, *125*(3), 236-243.e1. https://doi.org/10.1016/j.oooo.2017.10.016

Ebrahimi, M., Wahlin, Y.-B., Coates, P. J., Sjöström, B., & Nylander, K. (2006). Decreased expression of p63 in oral lichen planus and graft-vs.-host disease associated with oral inflammation. *Journal of Oral Pathology & Medicine : Official Publication of the International Association of Oral Pathologists and the American Academy of Oral Pathology*, *35*(1), 46–50. https://doi.org/10.1111/j.1600-0714.2005.00376.x

Ezzatt, O. M., & Helmy, I. M. (2019). Topical pimecrolimus versus betamethasone for oral lichen planus: a randomized clinical trial. *Clinical Oral Investigations*, *23*(2), 947–956. https://doi.org/10.1007/s00784-018-2519-6

Flatharta, C. O., Flint, S., Toner, M., & Mabruk, M. (2008). hTR RNA component as a marker of cellular proliferation in oral lichen planus. *Asian Pacific Journal of Cancer Prevention : APJCP*, *9*(2), 287–290. http://www.ncbi.nlm.nih.gov/pubmed/18712976

Giannelli, G., Milillo, L., Marinosci, F., Lo Muzio, L., Serpico, R., & Antonaci, S. (2001). Altered expression of integrins and basement membrane proteins in malignant and pre-malignant lesions of oral mucosa. *Journal of Biological Regulators and Homeostatic Agents*, *15*(4), 375–380. http://www.ncbi.nlm.nih.gov/pubmed/11860227

Girod, S. C., Krueger, G., & Pape, H. D. (1993). p53 and Ki 67 expression in preneoplastic and neoplastic lesions of the oral mucosa. *International Journal of Oral and Maxillofacial Surgery*, *22*(5), 285–288. https://doi.org/10.1016/s0901-5027(05)80517-x

Gombos, F., Serpico, R., Femiano, F., Zabatta, A., & Chiacchio, R. (1993). [The quantitative assessment of DNA in potentially cancerous cases of oral lichen]. *Minerva Stomatologica*, *42*(6), 257–264. http://www.ncbi.nlm.nih.gov/pubmed/8232132

Hadzi-Mihailovic, M., Cakic, S., Jankovic, S., Raybaud, H., Nedeljkovic, N., & Jankovic, L. (2012). Ki-67 expression in oral lichen planus. *Journal of B.U.ON. : Official Journal of the Balkan Union of Oncology*, *17*(1), 132–137. http://www.ncbi.nlm.nih.gov/pubmed/22517707

Hadzi-Mihailovic, M., Raybaud, H., Monteil, R., & Jankovic, L. (2009). Expression of Fas/FasL in patients with oral lichen planus. *Journal of B.U.ON. : Official Journal of the Balkan Union of Oncology*, *14*(3), 487–493. http://www.ncbi.nlm.nih.gov/pubmed/19810143

Häkkinen, L., Kainulainen, T., Salo, T., Grenman, R., & Larjava, H. (1999). Expression of integrin alpha9 subunit and tenascin in oral leukoplakia, lichen planus, and squamous cell carcinoma. *Oral Diseases*, *5*(3), 210–217. https://doi.org/10.1111/j.1601-0825.1999.tb00303.x

Hämäläinen, L., Soini, Y., Pasonen-Seppänen, S., & Siponen, M. (2019). Alterations in the expression of EMT-related proteins claudin-1, claudin-4 and claudin-7, E-cadherin, TWIST1 and ZEB1 in oral lichen planus. *Journal of Oral Pathology & Medicine : Official Publication of the International Association of Oral Pathologists and the American Academy of Oral Pathology*, *48*(8), 735–744. https://doi.org/10.1111/jop.12917

Hazzaa, H. H., El Shiekh, M. A. M., Abdelgawad, N., Gouda, O. M., & Kamal, N. M. (2020). Correlation of VEGF and MMP-2 levels in oral lichen planus: An in vivo immunohistochemical study. *Journal of Oral Biology and Craniofacial Research*, *10*(4), 747–752. https://doi.org/10.1016/j.jobcr.2020.10.009

Huang, X. F., Zhang, W. G., Song, L. J., & Fu, H. B. (1997). [Immunohistochemical detection of PCNA and p53 protein in the premalignant lesions and squamous cell carcinomas of the oral mucosa]. *Shanghai Kou Qiang Yi Xue = Shanghai Journal of Stomatology*, *6*(2), 73–74. http://www.ncbi.nlm.nih.gov/pubmed/15159933

Jainkittivong, A., Kuvatanasuchati, J., Pipattanagovit, P., & Sinheng, W. (2007). Candida in oral lichen planus patients undergoing topical steroid therapy. *Oral Surgery, Oral Medicine, Oral Pathology, Oral Radiology, and Endodontics*, *104*(1), 61–66. https://doi.org/10.1016/j.tripleo.2006.10.024

Kainulainen, T., Autio-Harmainen, H., Oikarinen, A., Salo, S., Tryggvason, K., & Salo, T. (1997). Altered distribution and synthesis of laminin-5 (kalinin) in oral lichen planus, epithelial dysplasias and squamous cell carcinomas. *The British Journal of Dermatology*, *136*(3), 331–336. http://www.ncbi.nlm.nih.gov/pubmed/9115910

Kainulainen, T., Grenman, R., Oikarinen, A., Greenspan, D. S., & Salo, T. (1997). Distribution and synthesis of type VII collagen in oral squamous cell carcinoma. *Journal of Oral Pathology & Medicine : Official Publication of the International Association of Oral Pathologists and the American Academy of Oral Pathology*, *26*(9), 414–418. https://doi.org/10.1111/j.1600-0714.1997.tb00241.x

Kashima, H. K., Kutcher, M., Kessis, T., Levin, L. S., de Villiers, E. M., & Shah, K. (1990). Human papillomavirus in squamous cell carcinoma, leukoplakia, lichen planus, and clinically normal epithelium of the oral cavity. *The Annals of Otology, Rhinology, and Laryngology*,

99(1), 55–61. https://doi.org/10.1177/000348949009900110

Katou, F., Ohtani, H., Watanabe, Y., Nakayama, T., Yoshie, O., & Hashimoto, K. (2007). Differing phenotypes between intraepithelial and stromal lymphocytes in early-stage tongue cancer. *Cancer Research*, *67*(23), 11195–11201. https://doi.org/10.1158/0008-5472.CAN-07-2637

Kis, A., Fehér, E., Gáll, T., Tar, I., Boda, R., Tóth, E. D., Méhes, G., Gergely, L., & Szarka, K. (2009). Epstein-Barr virus prevalence in oral squamous cell cancer and in potentially malignant oral disorders in an eastern Hungarian population. *European Journal of Oral Sciences*, *117*(5), 536–540. https://doi.org/10.1111/j.1600-0722.2009.00660.x

Kövesi, G. (2001). [Evaluation of clinical data and immuno-modulating treatment of patients with oral lichen planus]. *Fogorvosi Szemle*, *94*(1), 9–14. http://www.ncbi.nlm.nih.gov/pubmed/11262802

Kulkarni, G., Sakki, E. P., Kumar, Y. V., Kolimi, S., Perika, R., Karthik, K. V., Kumar, K. M., & Kalyan, V. S. (2016). Expression of CD1a by Langerhan's Cells in Oral Lichen Planus - A Retrospective Analysis. *Journal of Clinical and Diagnostic Research : JCDR*, *10*(6), ZC28-31. https://doi.org/10.7860/JCDR/2016/19189.7966

Li, W., Ling, Z., Wang, J., Su, Z., Lu, J., Yang, X., Cheng, B., & Tao, X. (2023). ASCT2-mediated glutamine uptake promotes Th1 differentiation via ROS-EGR1-PAC1 pathway in oral lichen planus. *Biochemical Pharmacology*, *216*, 115767. https://doi.org/10.1016/j.bcp.2023.115767

Liu, T., Zhang, H., Yang, X., Li, X., Shi, Y., Niu, W., & Liu, T. (2018). Study on expression of p16 and human papillomavirus 16 and 18 (E6) in OLP and its malignant transformation. *Pathology, Research and Practice*, *214*(2), 296–302. https://doi.org/10.1016/j.prp.2017.09.014

Liu, Y., Cui, L., Huang, J., Ji, E. H., Chen, W., Messadi, D., & Hu, S. (2016). SOX4 Promotes Progression in OLP-Associated Squamous Cell Carcinoma. *Journal of Cancer*, *7*(11), 1534–1540. https://doi.org/10.7150/jca.15689

Lončar-Brzak, B., Klobučar, M., Veliki-Dalić, I., Sabol, I., Kraljević Pavelić, S., Krušlin, B., & Mravak-Stipetić, M. (2018). Expression of small leucine-rich extracellular matrix proteoglycans biglycan and lumican reveals oral lichen planus malignant potential. *Clinical Oral Investigations*, *22*(2), 1071–1082. https://doi.org/10.1007/s00784-017-2190-3

Lu, J., Su, Z., Li, W., Ling, Z., Cheng, B., Yang, X., & Tao, X. (2023). ASCT2-mediated glutamine uptake of epithelial cells facilitates CCL5-induced T cell infiltration via ROS-STAT3 pathway in oral lichen planus. *International Immunopharmacology*, *119*, 110216. https://doi.org/10.1016/j.intimp.2023.110216

Maloth, A. K., Dorankula, S. P. R., Pasupula, A. P., Thokala, M. R., Muddana, K., & Ramavath, R. (2015). A Comparative Immunohistochemical Analysis of Langerhans Cells in Oral

Mucosa, Oral Lichen Planus and Oral Squamous Cell Carcinoma. *Journal of Clinical and Diagnostic Research : JCDR*, *9*(7), ZC76-9. https://doi.org/10.7860/JCDR/2015/14170.6235

Mane, D. R., Kale, A. D., & Belaldavar, C. (2017). Validation of immunoexpression of tenascin-C in oral precancerous and cancerous tissues using ImageJ analysis with novel immunohistochemistry profiler plugin: An immunohistochemical quantitative analysis. *Journal of Oral and Maxillofacial Pathology : JOMFP*, *21*(2), 211–217. https://doi.org/10.4103/jomfp.JOMFP_234_16

Mattila, R., Alanen, K., & Syrjänen, S. (2007). Immunohistochemical study on topoisomerase IIalpha, Ki-67 and cytokeratin-19 in oral lichen planus lesions. *Archives of Dermatological Research*, *298*(8), 381–388. https://doi.org/10.1007/s00403-006-0711-z

Mattila, R., Alanen, K., & Syrjänen, S. (2008). Desmocollin expression in oral atrophic lichen planus correlates with clinical behavior and DNA content. *Journal of Cutaneous Pathology*, *35*(9), 832–838. https://doi.org/10.1111/j.1600-0560.2007.00903.x

Mittal, N., Shankari, G. M., & Palaskar, S. (2012). Role of angiogenesis in the pathogenesis of oral lichen planus. *Journal of Oral and Maxillofacial Pathology : JOMFP*, *16*(1), 45–48. https://doi.org/10.4103/0973-029X.92972

Motta, A., Zhan, Q., Larson, A., Lerman, M., Woo, S.-B., Soiffer, R. J., Murphy, G. F., & Treister, N. S. (2018). Immunohistopathological characterization and the impact of topical immunomodulatory therapy in oral chronic graft-versus-host disease: A pilot study. *Oral Diseases*, *24*(4), 580–590. https://doi.org/10.1111/odi.12813

Muniz, J. M., Bibiano Borges, C. R., Beghini, M., de Araújo, M. S., Miranda Alves, P., de Lima, L. M. B., Pereira, S. A. de L., Nogueira, R. D., Napimoga, M. H., Rodrigues, V., & Rodrigues, D. B. R. (2015). Galectin-9 as an important marker in the differential diagnosis between oral squamous cell carcinoma, oral leukoplakia and oral lichen planus. *Immunobiology*, *220*(8), 1006–1011. https://doi.org/10.1016/j.imbio.2015.04.004

Parajuli, H., Teh, M.-T., Abrahamsen, S., Christoffersen, I., Neppelberg, E., Lybak, S., Osman, T., Johannessen, A. C., Gullberg, D., Skarstein, K., & Costea, D. E. (2017). Integrin α11 is overexpressed by tumour stroma of head and neck squamous cell carcinoma and correlates positively with alpha smooth muscle actin expression. *Journal of Oral Pathology & Medicine : Official Publication of the International Association of Oral Pathologists and the American Academy of Oral Pathology*, *46*(4), 267–275. https://doi.org/10.1111/jop.12493

Pérez-Sayáns, M., Lorenzo-Pouso, A. I., Chamorro-Petronacci, C. M., Suárez-Peñaranda, J. M., Padín-Iruegas, E., González-Moles, M. A., Marichalar-Mendía, X., García-García, A., & Blanco-Carrión, A. (n.d.). Immunoexpression of Apoptosis and Cell-cycle Arrest Markers in Oral Lichen Planus. *Applied Immunohistochemistry & Molecular Morphology : AIMM*, *29*(5), 374–381. https://doi.org/10.1097/PAI.0000000000000876

Pimenta, F. J. G. S., Pinheiro, M. das G. R., & Gomez, R. S. (2004). Expression of hMSH2 protein

of the human DNA mismatch repair system in oral lichen planus. *International Journal of Medical Sciences*, *1*(3), 146–151. https://doi.org/10.7150/ijms.1.146

Pramod, R., Pandit, S., Desai, D., Suresh, K., Ingaleshwar, P., Shetty, S., & Ahamad, S. (2014). Immunohistochemical assessment of proliferating cell nuclear antigen protein expression in plaque, reticular and erosive types of oral lichen planus. *Annals of Medical and Health Sciences Research*, *4*(4), 598–602. https://doi.org/10.4103/2141-9248.139337

Raybaud, H., Olivieri, C. V, Lupi-Pegurier, L., Pagnotta, S., Marsault, R., Cardot-Leccia, N., & Doglio, A. (2018). Epstein-Barr Virus-Infected Plasma Cells Infiltrate Erosive Oral Lichen Planus. *Journal of Dental Research*, *97*(13), 1494–1500. https://doi.org/10.1177/0022034518788282

Rosa, E. A., Hurtado-Puerto, A. M., Falcão, D. P., Brietzke, A. P., De Almeida Prado Franceschi, L. E., Cavalcanti Neto, F. F., Tiziane, V., Carneiro, F. P., Kogawa, E. M., Moreno, H., & Amorim, R. F. B. (2018). Oral lichen planus and malignant transformation: The role of p16, Ki-67, Bub-3 and SOX4 in assessing precancerous potential. *Experimental and Therapeutic Medicine*, *15*(5), 4157–4166. https://doi.org/10.3892/etm.2018.5971

Rusanen, P., Marttila, E., Uittamo, J., Hagström, J., Salo, T., & Rautemaa-Richardson, R. (2017). TLR1-10, NF-κB and p53 expression is increased in oral lichenoid disease. *PloS One*, *12*(7), e0181361. https://doi.org/10.1371/journal.pone.0181361

Shahidi, M., Jafari, S., Barati, M., Mahdipour, M., & Gholami, M. S. (2017). Predictive value of salivary microRNA-320a, vascular endothelial growth factor receptor 2, CRP and IL-6 in Oral lichen planus progression. *Inflammopharmacology*. https://doi.org/10.1007/s10787-017-0352-1

Shi, W., Yang, J., Li, S., Shan, X., Liu, X., Hua, H., Zhao, C., Feng, Z., Cai, Z., Zhang, L., & Zhou, D. (2015). Potential involvement of miR-375 in the premalignant progression of oral squamous cell carcinoma mediated via transcription factor KLF5. *Oncotarget*, *6*(37), 40172–40185. https://doi.org/10.18632/oncotarget.5502

Shrikaar, M., Suwasini, S., Chatterjee, K., Jha, A., Kumar, M., & Dave, K. (2023). A retrospective study on immunohistochemical evaluation of CD34 in the pathogenesis of oral lichen planus. *Journal of Oral and Maxillofacial Pathology : JOMFP*, *27*(1), 49–53. https://doi.org/10.4103/jomfp.jomfp_437_20

Siponen, M., Kauppila, J. H., Soini, Y., & Salo, T. (2012). TLR4 and TLR9 are induced in oral lichen planus. *Journal of Oral Pathology & Medicine : Official Publication of the International Association of Oral Pathologists and the American Academy of Oral Pathology*, *41*(10), 741–747. https://doi.org/10.1111/j.1600-0714.2012.01169.x

Sniezek, J. C., Matheny, K. E., Westfall, M. D., & Pietenpol, J. A. (2004). Dominant negative p63 isoform expression in head and neck squamous cell carcinoma. *The Laryngoscope*, *114*(12), 2063–2072. https://doi.org/10.1097/01.mlg.0000149437.35855.4b

Suganya, G., Bavle, R. M., Paremala, K., Makarla, S., Sudhakar, M., & Reshma, V. (2016).

Survivin expression in oral lichen planus: Role in malignant transformation. *Journal of Oral and Maxillofacial Pathology : JOMFP*, *20*(2), 234–238. https://doi.org/10.4103/0973-029X.185912

Sun, Y., Liu, N., Guan, X., Wu, H., Sun, Z., & Zeng, H. (2016). Immunosuppression Induced by Chronic Inflammation and the Progression to Oral Squamous Cell Carcinoma. *Mediators of Inflammation*, *2016*, 5715719. https://doi.org/10.1155/2016/5715719

Sutinen, M., Kainulainen, T., Hurskainen, T., Vesterlund, E., Alexander, J. P., Overall, C. M., Sorsa, T., & Salo, T. (1998). Expression of matrix metalloproteinases (MMP-1 and -2) and their inhibitors (TIMP-1, -2 and -3) in oral lichen planus, dysplasia, squamous cell carcinoma and lymph node metastasis. *British Journal of Cancer*, *77*(12), 2239–2245. https://doi.org/10.1038/bjc.1998.372

Syrjänen, S. M., Syrjänen, K. J., & Lamberg, M. A. (1986). Detection of human papillomavirus DNA in oral mucosal lesions using in situ DNA-hybridization applied on paraffin sections. *Oral Surgery, Oral Medicine, and Oral Pathology*, *62*(6), 660–667. https://doi.org/10.1016/0030-4220(86)90262-8

Valente, G., Pagano, M., Carrozzo, M., Carbone, M., Bobba, V., Palestro, G., & Gandolfo, S. (2001). Sequential immunohistochemical p53 expression in biopsies of oral lichen planus undergoing malignant evolution. *Journal of Oral Pathology & Medicine : Official Publication of the International Association of Oral Pathologists and the American Academy of Oral Pathology*, *30*(3), 135–140. https://doi.org/10.1034/j.1600-0714.2001.300302.x

van Heerden, W. F., Swart, T. J., van Heerden, M. B., Pekarsky, Y., Sutherland, R., & Huebner, K. (2001). Fhit protein expression in oral epithelium: immunohistochemical evaluation of three antisera. *Anticancer Research*, *21*(4A), 2419–2423. http://www.ncbi.nlm.nih.gov/pubmed/11724301

Wang, K., Shen, Y., Xu, J., Li, Z., Liu, Y., Yu, C., Peng, L., Zheng, J., & Zeng, Y. (2020). Evaluation of synuclein-γ levels by novel monoclonal antibody in saliva and cancer tissues from oral squamous cell carcinoma patients. *Neoplasma*, *67*(3), 707–713. https://doi.org/10.4149/neo_2020_190619N523

Wang, L.-L., Wang, R.-C., Wang, L.-Z., Sun, L.-R., & Qi, X.-M. (2018). Expression of keratinocyte growth factor and its receptor in oral lichen planus. *International Journal of Clinical and Experimental Pathology*, *11*(2), 757–764. http://www.ncbi.nlm.nih.gov/pubmed/31938162

Wang, L., Wu, W., Chen, J., Li, Y., Xu, M., & Cai, Y. (2018). MicroRNA Microarray-Based Identification of Involvement of miR-155 and miR-19a in Development of Oral Lichen Planus (OLP) by Modulating Th1/Th2 Balance via Targeting eNOS and Toll-Like Receptor 2 (TLR2). *Medical Science Monitor : International Medical Journal of Experimental and Clinical Research*, *24*, 3591–3603. https://doi.org/10.12659/MSM.907497

Wang, X.-X., Sun, H.-Y., Yang, Q.-Z., Guo, B., Sai, Y., & Zhang, J. (2017). Hypoxia-inducible

factor-1α and glucose transporter 1 in the malignant transformation of oral lichen planus. *International Journal of Clinical and Experimental Pathology*, *10*(8), 8369–8376. http://www.ncbi.nlm.nih.gov/pubmed/31966688

Yang, Q., Sun, H., Wang, X., Yu, X., Zhang, J., Guo, B., & Hexige, S. (2020). Metabolic changes during malignant transformation in primary cells of oral lichen planus: Succinate accumulation and tumour suppression. *Journal of Cellular and Molecular Medicine*, *24*(2), 1179–1188. https://doi.org/10.1111/jcmm.14376

Yao, X., Yin, C., Shen, L., & Xie, S. (2007). [Expressions of NF--kappaBp65, TRAF2, cyclinD1 and their association with cell apoptosis in oral lichen planus]. *Nan Fang Yi Ke Da Xue Xue Bao = Journal of Southern Medical University*, *27*(11), 1657–1660. http://www.ncbi.nlm.nih.gov/pubmed/18024283

Yao, Y., Pan, L., Wei, Y., Feng, M., Li, X., Sun, L., Tang, G., & Wang, Y. (2023). TRIM21 promotes inflammation by ubiquitylating NF-κB in T cells of oral lichen planus. *Journal of Oral Pathology & Medicine : Official Publication of the International Association of Oral Pathologists and the American Academy of Oral Pathology*, *52*(5), 448–455. https://doi.org/10.1111/jop.13428

Zargaran, M., Jamshidi, S., Eshghyar, N., & Moghimbeigi, A. (2013). Suitability/unsuitability of cell proliferation as an indicator of malignant potential in oral lichen planus: an immunohistochemical study. *Asian Pacific Journal of Cancer Prevention : APJCP*, *14*(11), 6979–6983. https://doi.org/10.7314/apjcp.2013.14.11.6979

Zhao, Z. Z., Savage, N. W., & Walsh, L. J. (1998). Associations between mast cells and laminin in oral lichen planus. *Journal of Oral Pathology & Medicine : Official Publication of the International Association of Oral Pathologists and the American Academy of Oral Pathology*, *27*(4), 163–167. https://doi.org/10.1111/j.1600-0714.1998.tb01934.x

11.2 List S3. Non-Immunohistochemistry technique (n=245)

Abdel Hay, R. M., Fawzy, M. M., Metwally, D., Kadry, D., Ezzat, M., Rashwan, W., & Rashed, L. A. (2012). DNA polymorphisms and tissue cyclooxygenase-2 expression in oral lichen planus: a case-control study. *Journal of the European Academy of Dermatology and Venereology : JEADV*, *26*(9), 1122–1126. https://doi.org/10.1111/j.1468-3083.2011.04229.x

Accurso, B. T., Warner, B. M., Knobloch, T. J., Weghorst, C. M., Shumway, B. S., Allen, C. M., & Kalmar, J. R. (2011). Allelic imbalance in oral lichen planus and assessment of its classification as a premalignant condition. *Oral Surgery, Oral Medicine, Oral Pathology, Oral Radiology, and Endodontics*, *112*(3), 359–366. https://doi.org/10.1016/j.tripleo.2011.03.042

Adtani, P., & Malathi, N. (2015). Epstein-Barr virus and its association with rheumatoid arthritis and oral lichen planus. *Journal of Oral and Maxillofacial Pathology : JOMFP*, *19*(3), 282–285. https://doi.org/10.4103/0973-029X.174643

Agha-Hosseini, F., Barati, H., & Moosavi, M.-S. (2020). Aquaporin3 (AQP3) expression in oral epithelium in oral lichen planus. *Experimental and Molecular Pathology*, *115*, 104441. https://doi.org/10.1016/j.yexmp.2020.104441

Agha-Hosseini, F., & Mirzaii-Dizgah, I. (2013). p53 as a neoplastic biomarker in patients with erosive and plaque like forms of oral lichen planus. *The Journal of Contemporary Dental Practice*, *14*(1), 1–3. https://doi.org/10.5005/jp-journals-10024-1259

Agha-Hosseini, F., & Mirzaii-Dizgah, I. (2015). Serum and saliva collagenase-3 (MMP-13) in patients with oral lichen planus and oral squamous cell carcinoma. *Medical Journal of the Islamic Republic of Iran*, *29*, 218. http://www.ncbi.nlm.nih.gov/pubmed/26478876

Agha-Hosseini, F., Mirzaii-Dizgah, I., Farmanbar, N., & Abdollahi, M. (2012). Oxidative stress status and DNA damage in saliva of human subjects with oral lichen planus and oral squamous cell carcinoma. *Journal of Oral Pathology & Medicine : Official Publication of the International Association of Oral Pathologists and the American Academy of Oral Pathology*, *41*(10), 736–740. https://doi.org/10.1111/j.1600-0714.2012.01172.x

Agha-Hosseini, F., Mirzaii-Dizgah, I., Mahboobi, N., Shirazian, S., & Harirchi, I. (2015). Serum and Saliva MMP-3 in Patients with OLP and Oral SCC. *The Journal of Contemporary Dental Practice*, *16*(2), 107–111. https://doi.org/10.5005/jp-journals-10024-1645

Agha-Hosseini, F., Mirzaii-Dizgah, I., & Miri-Zarandi, N. (2015). Unstimulated salivary p53 in patients with oral lichen planus and squamous cell carcinoma. *Acta Medica Iranica*, *53*(7), 439–443. http://www.ncbi.nlm.nih.gov/pubmed/26520632

Agha-Hosseini, F., Mohebbian, M., Sarookani, M.-R., Harirchi, I., & Mirzaii-Dizgah, I. (2015). Comparative Evaluation of EGF in Oral Lichen Planus and Oral Squamous Cell Carcinoma. *Acta Medica Iranica*, *53*(8), 471–475. http://www.ncbi.nlm.nih.gov/pubmed/26545991

Aghbari, S. M. H., Gaafar, S. M., Shaker, O. G., Ashiry, S. El, & Zayed, S. O. (2018). Evaluating

the accuracy of microRNA27b and microRNA137 as biomarkers of activity and potential malignant transformation in oral lichen planus patients. *Archives of Dermatological Research*, *310*(3), 209–220. https://doi.org/10.1007/s00403-018-1805-0

Ahmadi-Motamayel, F., Bayat, Z., Hajilooi, M., Shahryar-Hesami, S., Mahdavinezhad, A., Samie, L., & Solgi, G. (2017). Evaluation of the miRNA-146a and miRNA-155 Expression Levels in Patients with Oral Lichen Planus. *Iranian Journal of Immunology : IJI*, *14*(4), 316–324. http://www.ncbi.nlm.nih.gov/pubmed/29276184

Al-Mohaya, M. A., Al-Harthi, F., Arfin, M., & Al-Asmari, A. (2015). TNF-α, TNF-β and IL-10 gene polymorphism and association with oral lichen planus risk in Saudi patients. *Journal of Applied Oral Science : Revista FOB*, *23*(3), 295–301. https://doi.org/10.1590/1678-775720150075

Al-Mohaya, M. A. M., Al-Otaibi, L., Al-Harthi, F., Al Bakr, E., Arfin, M., & Al-Asmari, A. (2016). Association of genetic polymorphisms in interferon-γ, interleukin-6 and transforming growth factor-β1 gene with oral lichen planus susceptibility. *BMC Oral Health*, *16*(1), 76. https://doi.org/10.1186/s12903-016-0277-x

Aliev, A. D., Mikhailovskii, V. M., Perlamutrov, Y. N., & Kushlinskii, N. E. (2007). Soluble Fas antigen in the serum of women with oral lichen planus. *Bulletin of Experimental Biology and Medicine*, *143*(6), 727–729. https://doi.org/10.1007/s10517-007-0225-5

Alikhani, M., Ghalaiani, P., Askariyan, E., Khunsaraki, Z. A., Tavangar, A., & Naderi, A. (2017). Association between the clinical severity of oral lichen planus and anti-TPO level in thyroid patients. *Brazilian Oral Research*, *31*, e10. https://doi.org/10.1590/1807-3107BOR-2017.vol31.0010

Aniyan, K. Y., Guledgud, M. V, & Patil, K. (2018). Alterations of Serum Lipid Profile Patterns in Oral Lichen Planus Patients: A Case-Control Study. *Contemporary Clinical Dentistry*, *9*(Suppl 1), S112–S121. https://doi.org/10.4103/ccd.ccd_111_18

Arirachakaran, P., Chansaengroj, J., Lurchachaiwong, W., Kanjanabud, P., Thongprasom, K., & Poovorawan, Y. (2013). Oral lichen planus in thai patients has a low prevalence of human papillomavirus. *ISRN Dentistry*, *2013*, 362750. https://doi.org/10.1155/2013/362750

Arreaza, A., Rivera, H., & Correnti, M. (2015). p53 expression in oral lichenoid lesions and oral lichen planus. *General Dentistry*, *63*(1), 69–72. http://www.ncbi.nlm.nih.gov/pubmed/25574723

Artico, G., Freitas, R. S., Santos Filho, A. M., Benard, G., Romiti, R., & Migliari, D. A. (2014). Prevalence of Candida spp., xerostomia, and hyposalivation in oral lichen planus--a controlled study. *Oral Diseases*, *20*(3), e36-41. https://doi.org/10.1111/odi.12120

Ayinampudi, B. K., & Narsimhan, M. (2012). Salivary copper and zinc levels in oral pre-malignant and malignant lesions. *Journal of Oral and Maxillofacial Pathology : JOMFP*, *16*(2), 178–182. https://doi.org/10.4103/0973-029X.98452

Azab, N. A., Abd El Salam, L., Ahmed, E., El Sharkawy, M., ElSharkawy, A., & El Asheiry, S. G.

(2018). Interferon gamma and interleukin 8 gene polymorphisms in patients with hepatitis C virus related oral lichen planus. *Archives of Oral Biology*, *96*, 189–194. https://doi.org/10.1016/j.archoralbio.2018.09.015

Bai, J., Jiang, L., Lin, M., Zeng, X., Wang, Z., & Chen, Q. (2009). Association of polymorphisms in the tumor necrosis factor-alpha and interleukin-10 genes with oral lichen planus: a study in a chinese cohort with Han ethnicity. *Journal of Interferon & Cytokine Research : The Official Journal of the International Society for Interferon and Cytokine Research*, *29*(7), 381–388. https://doi.org/10.1089/jir.2008.0089

Bai, J., Lin, M., Zeng, X., Zhang, Y., Wang, Z., Shen, J., Jiang, L., Gao, F., & Chen, Q. (2008). Association of polymorphisms in the human IFN-gamma and IL-4 gene with oral lichen planus: a study in an ethnic Chinese cohort. *Journal of Interferon & Cytokine Research : The Official Journal of the International Society for Interferon and Cytokine Research*, *28*(6), 351–358. https://doi.org/10.1089/jir.2007.0056

Banerjee, S., Mukherjee, S., Mitra, S., & Singhal, P. (2020). Comparative Evaluation of Mitochondrial Antioxidants in Oral Potentially Malignant Disorders. *The Kurume Medical Journal*, *66*(1), 15–27. https://doi.org/10.2739/kurumemedj.MS661009

Battino, M., Greabu, M., Totan, A., Bullon, P., Bucur, A., Tovaru, S., Mohora, M., Didilescu, A., Parlatescu, I., Spinu, T., & Totan, C. (2008). Oxidative stress markers in oral lichen planus. *BioFactors (Oxford, England)*, *33*(4), 301–310. https://doi.org/10.1002/biof.5520330406

Batu, Ş., Ofluoğlu, D., Ergun, S., Warnakulasuriya, S., Uslu, E., Güven, Y., & Tanyeri, H. (2016). Evaluation of prolidase activity and oxidative stress in patients with oral lichen planus and oral lichenoid contact reactions. *Journal of Oral Pathology & Medicine : Official Publication of the International Association of Oral Pathologists and the American Academy of Oral Pathology*, *45*(4), 281–288. https://doi.org/10.1111/jop.12391

Bediaga, N. G., Marichalar-Mendia, X., Aguirre-Urizar, J. M., Calvo, B., Echebarria-Goicouria, M. A., de Pancorbo, M. M., & Acha-Sagredo, A. (2014). Global DNA methylation: uncommon event in oral lichenoid disease. *Oral Diseases*, *20*(8), 821–826. https://doi.org/10.1111/odi.12243

Bokor-Bratic, M., Cankovic, M., & Dragnic, N. (2013). Unstimulated whole salivary flow rate and anxiolytics intake are independently associated with oral Candida infection in patients with oral lichen planus. *European Journal of Oral Sciences*, *121*(5), 427–433. https://doi.org/10.1111/eos.12073

Bombeccari, G. P., Tettamanti, M., Pallotti, F., Spadari, F., & Giannì, A. B. (2017). Exacerbations of oral lichen planus and elevated levels of aminotransferases. *International Journal of Dermatology*, *56*(8), 842–849. https://doi.org/10.1111/ijd.13626

Bose, A., Teh, M.-T., Hutchison, I. L., Wan, H., Leigh, I. M., & Waseem, A. (2012). Two mechanisms regulate keratin K15 expression in keratinocytes: role of PKC/AP-1 and FOXM1 mediated signalling. *PloS One*, *7*(6), e38599. https://doi.org/10.1371/journal.pone.0038599

Brands, R. C., Köhler, O., Rauthe, S., Hartmann, S., Ebhardt, H., Seher, A., Linz, C., Kübler, A. C., & Müller-Richter, U. D. A. (2017). The prognostic value of GLUT-1 staining in the detection of malignant transformation in oral mucosa. *Clinical Oral Investigations*, *21*(5), 1631–1637. https://doi.org/10.1007/s00784-016-1954-5

Bruno, E., Alessandrini, M., Russo, S., D'Erme, G., Nucci, R., & Calabretta, F. (2002). Malignant degeneration of oral lichen planus: our clinical experience and review of the literature. *Anales Otorrinolaringologicos Ibero-Americanos*, *29*(4), 349–357. http://www.ncbi.nlm.nih.gov/pubmed/12462928

Campisi, G., Giovannelli, L., Aricò, P., Lama, A., Di Liberto, C., Ammatuna, P., & D'Angelo, M. (2004). HPV DNA in clinically different variants of oral leukoplakia and lichen planus. *Oral Surgery, Oral Medicine, Oral Pathology, Oral Radiology, and Endodontics*, *98*(6), 705–711. https://doi.org/10.1016/j.tripleo.2004.04.012

Carrozzo, M., Dametto, E., Fasano, M. E., Arduino, P., Bertolusso, G., Uboldi de Capei, F., Rendine, S., & Amoroso, A. (2007). Cytokine gene polymorphisms in hepatitis C virus-related oral lichen planus. *Experimental Dermatology*, *16*(9), 730–736. https://doi.org/10.1111/j.1600-0625.2007.00577.x

Carrozzo, M., Francia Di Celle, P., Gandolfo, S., Carbone, M., Conrotto, D., Fasano, M. E., Roggero, S., Rendine, S., & Ghisetti, V. (2001). Increased frequency of HLA-DR6 allele in Italian patients with hepatitis C virus-associated oral lichen planus. *The British Journal of Dermatology*, *144*(4), 803–808. https://doi.org/10.1046/j.1365-2133.2001.04136.x

Carrozzo, M., Uboldi de Capei, M., Dametto, E., Fasano, M. E., Arduino, P., Broccoletti, R., Vezza, D., Rendine, S., Curtoni, E. S., & Gandolfo, S. (2004). Tumor necrosis factor-alpha and interferon-gamma polymorphisms contribute to susceptibility to oral lichen planus. *The Journal of Investigative Dermatology*, *122*(1), 87–94. https://doi.org/10.1046/j.0022-202X.2003.22108.x

Ceballos-Salobreña, A., Aguirre-Urizar, J. M., & Bagan-Sebastian, J. V. (1996). Oral manifestations associated with human immunodeficiency virus infection in a Spanish population. *Journal of Oral Pathology & Medicine : Official Publication of the International Association of Oral Pathologists and the American Academy of Oral Pathology*, *25*(10), 523–526. https://doi.org/10.1111/j.1600-0714.1996.tb01725.x

Chainani-Wu, N., Madden, E., Lozada-Nur, F., & Silverman, S. (2012). High-dose curcuminoids are efficacious in the reduction in symptoms and signs of oral lichen planus. *Journal of the American Academy of Dermatology*, *66*(5), 752–760. https://doi.org/10.1016/j.jaad.2011.04.022

Chaiyarit, P., Ma, N., Hiraku, Y., Pinlaor, S., Yongvanit, P., Jintakanon, D., Murata, M., Oikawa, S., & Kawanishi, S. (2005). Nitrative and oxidative DNA damage in oral lichen planus in relation to human oral carcinogenesis. *Cancer Science*, *96*(9), 553–559. https://doi.org/10.1111/j.1349-7006.2005.00096.x

Chaiyarit, P., Taweechaisupapong, S., Jaresitthikunchai, J., Phaonakrop, N., & Roytrakul, S.

(2015). Comparative evaluation of 5-15-kDa salivary proteins from patients with different oral diseases by MALDI-TOF/TOF mass spectrometry. *Clinical Oral Investigations*, *19*(3), 729–737. https://doi.org/10.1007/s00784-014-1293-3

Chang, J. Y.-F., Chiang, C.-P., Hsiao, C. K., & Sun, A. (2009). Significantly higher frequencies of presence of serum autoantibodies in Chinese patients with oral lichen planus. *Journal of Oral Pathology & Medicine : Official Publication of the International Association of Oral Pathologists and the American Academy of Oral Pathology*, *38*(1), 48–54. https://doi.org/10.1111/j.1600-0714.2008.00686.x

Chauhan, I., Beena, V. T., Srinivas, L., Sathyan, S., & Banerjee, M. (2013). Association of cytokine gene polymorphisms with oral lichen planus in Malayalam-speaking ethnicity from South India (Kerala). *Journal of Interferon & Cytokine Research : The Official Journal of the International Society for Interferon and Cytokine Research*, *33*(8), 420–427. https://doi.org/10.1089/jir.2012.0115

Chen, J., Du, G., Wang, Y., Shi, L., Mi, J., & Tang, G. (2017). Integrative analysis of mRNA and miRNA expression profiles in oral lichen planus: preliminary results. *Oral Surgery, Oral Medicine, Oral Pathology and Oral Radiology*, *124*(4), 390-402.e17. https://doi.org/10.1016/j.oooo.2017.05.513

Chen, J., Wang, Y., Du, G., Zhang, W., Cao, T., Shi, L., Wang, Y., Mi, J., & Tang, G. (2019). Down-regulation of miRNA-27b-3p suppresses keratinocytes apoptosis in oral lichen planus. *Journal of Cellular and Molecular Medicine*, *23*(6), 4326–4337. https://doi.org/10.1111/jcmm.14324

Cheng, B., Rhodus, N. L., Williams, B., & Griffin, R. J. (2004). Detection of apoptotic cells in whole saliva of patients with oral premalignant and malignant lesions: a preliminary study. *Oral Surgery, Oral Medicine, Oral Pathology, Oral Radiology, and Endodontics*, *97*(4), 465–470. https://doi.org/10.1016/j.tripleo.2003.12.020

Cheng, Y.-S. L., Jordan, L., Rees, T., Chen, H.-S., Oxford, L., Brinkmann, O., & Wong, D. (2014). Levels of potential oral cancer salivary mRNA biomarkers in oral cancer patients in remission and oral lichen planus patients. *Clinical Oral Investigations*, *18*(3), 985–993. https://doi.org/10.1007/s00784-013-1041-0

Chitturi, R. T., Nirmal, R. M., Sunil, P. M., Devy, A. S., & Reddy, B. V. R. (2014). Evaluation of ploidy status using DNA-image cytometry of exfoliated mucosal cells in oral lichen planus. *Journal of Cytology*, *31*(3), 131–135. https://doi.org/10.4103/0970-9371.145629

Cox, M., Maitland, N., & Scully, C. (1993). Human herpes simplex-1 and papillomavirus type 16 homologous DNA sequences in normal, potentially malignant and malignant oral mucosa. *European Journal of Cancer. Part B, Oral Oncology*, *29B*(3), 215–219. https://doi.org/10.1016/0964-1955(93)90025-a

Cruz, I., Van den Brule, A. J., Steenbergen, R. D., Snijders, P. J., Meijer, C. J., Walboomers, J. M., Snow, G. B., & Van der Waal, I. (1997). Prevalence of Epstein-Barr virus in oral squamous cell carcinomas, premalignant lesions and normal mucosa--a study using the polymerase

chain reaction. *Oral Oncology*, *33*(3), 182–188. https://doi.org/10.1016/s0964-1955(96)00054-1

Czerninski, R., Basile, J. R., Kartin-Gabay, T., Laviv, A., & Barak, V. (2014). Cytokines and tumor markers in potentially malignant disorders and oral squamous cell carcinoma: a pilot study. *Oral Diseases*, *20*(5), 477–481. https://doi.org/10.1111/odi.12160

Dang, J., Bian, Y.-Q., Sun, J. Y., Chen, F., Dong, G.-Y., Liu, Q., Wang, X.-W., Kjems, J., Gao, S., & Wang, Q.-T. (2013). MicroRNA-137 promoter methylation in oral lichen planus and oral squamous cell carcinoma. *Journal of Oral Pathology & Medicine : Official Publication of the International Association of Oral Pathologists and the American Academy of Oral Pathology*, *42*(4), 315–321. https://doi.org/10.1111/jop.12012

Danielsson, K., Nylander, E., Sjöström, M., & Ebrahimi, M. (2018). Epstein-Barr virus is not detected in mucosal lichen planus. *Medicina Oral, Patologia Oral y Cirugia Bucal*, *23*(5), e560–e563. https://doi.org/10.4317/medoral.22617

Danielsson, K., Wahlin, Y. B., Gu, X., Boldrup, L., & Nylander, K. (2012). Altered expression of miR-21, miR-125b, and miR-203 indicates a role for these microRNAs in oral lichen planus. *Journal of Oral Pathology & Medicine : Official Publication of the International Association of Oral Pathologists and the American Academy of Oral Pathology*, *41*(1), 90–95. https://doi.org/10.1111/j.1600-0714.2011.01084.x

de Barros Gallo, C., Marichalar-Mendia, X., Setien-Olarra, A., Acha-Sagredo, A., Bediaga, N. G., Gainza-Cirauqui, M. L., Sugaya, N. N., & Aguirre-Urizar, J. M. (2017). Toll-like receptor 2 rs4696480 polymorphism and risk of oral cancer and oral potentially malignant disorder. *Archives of Oral Biology*, *82*, 109–114. https://doi.org/10.1016/j.archoralbio.2017.06.003

de Carvalho Fraga, C. A., Alves, L. R., Marques-Silva, L., de Sousa, A. A., Jorge, A. S. B., de Jesus, S. F., Vilela, D. N., Pinheiro, U. B., Jones, K. M., de Paula, A. M. B., & Guimarães, A. L. S. (2013). High HIF-1α expression genotypes in oral lichen planus. *Clinical Oral Investigations*, *17*(9), 2011–2015. https://doi.org/10.1007/s00784-013-0920-8

Desai, S. S., Ghaisas, S. D., Jakhi, S. D., & Bhide, S. V. (1996). Cytogenetic damage in exfoliated oral mucosal cells and circulating lymphocytes of patients suffering from precancerous oral lesions. *Cancer Letters*, *109*(1–2), 9–14. https://doi.org/10.1016/s0304-3835(96)04390-x

Dillenburg, C. S., Martins, M. A. T., Almeida, L. O., Meurer, L., Squarize, C. H., Martins, M. D., & Castilho, R. M. (2015). Epigenetic Modifications and Accumulation of DNA Double-Strand Breaks in Oral Lichen Planus Lesions Presenting Poor Response to Therapy. *Medicine*, *94*(30), e997. https://doi.org/10.1097/MD.0000000000000997

Divya, V. C., & Sathasivasubramanian, S. (2014). Estimation of serum and salivary immunoglobulin G and immunoglobulin A in oral pre-cancer: A study in oral submucous fibrosis and oral lichen planus. *Journal of Natural Science, Biology, and Medicine*, *5*(1), 90–94. https://doi.org/10.4103/0976-9668.127294

Ebrahimi, M., Boldrup, L., Coates, P. J., Wahlin, Y.-B., Bourdon, J.-C., & Nylander, K. (2008). Expression of novel p53 isoforms in oral lichen planus. *Oral Oncology*, *44*(2), 156–161. https://doi.org/10.1016/j.oraloncology.2007.01.014

Ebrahimi, M., Boldrup, L., Wahlin, Y.-B., Coates, P. J., & Nylander, K. (2008). Decreased expression of the p63 related proteins beta-catenin, E-cadherin and EGFR in oral lichen planus. *Oral Oncology*, *44*(7), 634–638. https://doi.org/10.1016/j.oraloncology.2007.08.001

Fan, Y., Zhan, Z., & Liu, J. (2007). [Detection of differentially expressed genes in oral lichen planus]. *Hua Xi Kou Qiang Yi Xue Za Zhi = Huaxi Kouqiang Yixue Zazhi = West China Journal of Stomatology*, *25*(4), 378–382. http://www.ncbi.nlm.nih.gov/pubmed/17896497

Farah, C. S., Kordbacheh, F., John, K., Bennett, N., & Fox, S. A. (2018). Molecular classification of autofluorescence excision margins in oral potentially malignant disorders. *Oral Diseases*, *24*(5), 732–740. https://doi.org/10.1111/odi.12818

Fehér, E., Kardos, G., Gáll, T., Kis, A., Gergely, L., & Szarka, K. (2011). Comparison of diversity of torque teno virus 1 in different mucosal tissues and disorders. *Acta Microbiologica et Immunologica Hungarica*, *58*(4), 319–337. https://doi.org/10.1556/AMicr.58.2011.4.8

Femiano, F., & Scully, C. (2005). DNA cytometry of oral leukoplakia and oral lichen planus. *Medicina Oral, Patologia Oral y Cirugia Bucal*, *10 Suppl 1*, E9-14. http://www.ncbi.nlm.nih.gov/pubmed/15800471

Ferreira, S. J., Machado, M. Â. N., de Lima, A. A. S., Johann, A. C. B. R., Grégio, A. M. T., & Azevedo-Alanis, L. R. (2017). Identification of AgNORs and cytopathological changes in oral lichen planus lesions. *Acta Histochemica*, *119*(1), 32–38. https://doi.org/10.1016/j.acthis.2016.11.004

Flatharta, C. O., Flint, S., Toner, M., & Mabruk, M. (2008). hTR RNA component as a marker of cellular proliferation in oral lichen planus. *Asian Pacific Journal of Cancer Prevention : APJCP*, *9*(2), 287–290. http://www.ncbi.nlm.nih.gov/pubmed/18712976

Fujita, H., Kobayashi, T., Tai, H., Nagata, M., Hoshina, H., Nishizawa, R., Takagi, R., & Yoshie, H. (2009). Assessment of 14 functional gene polymorphisms in Japanese patients with oral lichen planus: a pilot case-control study. *International Journal of Oral and Maxillofacial Surgery*, *38*(9), 978–983. https://doi.org/10.1016/j.ijom.2009.05.001

Fujita, H., Nagata, M., Hoshina, H., Nagashima, K., Seki, Y., Tanaka, K., Nishizawa, R., Shingaki, S., Ohnishi, M., & Takagi, R. (2004). Clinical significance and usefulness of quantification of telomerase activity in oral malignant and nonmalignant lesions. *International Journal of Oral and Maxillofacial Surgery*, *33*(7), 693–699. https://doi.org/10.1016/j.ijom.2004.01.016

Furrer, V. E., Benitez, M. B., Furnes, M., Lanfranchi, H. E., & Modesti, N. M. (2006). Biopsy vs. superficial scraping: detection of human papillomavirus 6, 11, 16, and 18 in potentially

malignant and malignant oral lesions. *Journal of Oral Pathology & Medicine : Official Publication of the International Association of Oral Pathologists and the American Academy of Oral Pathology*, *35*(6), 338–344. https://doi.org/10.1111/j.1600-0714.2006.00423.x

Gainza-Cirauqui, M. L., Nieminen, M. T., Novak Frazer, L., Aguirre-Urizar, J. M., Moragues, M. D., & Rautemaa, R. (2013). Production of carcinogenic acetaldehyde by Candida albicans from patients with potentially malignant oral mucosal disorders. *Journal of Oral Pathology & Medicine : Official Publication of the International Association of Oral Pathologists and the American Academy of Oral Pathology*, *42*(3), 243–249. https://doi.org/10.1111/j.1600-0714.2012.01203.x

Garg, D., Sunil, M. K., Singh, P. P., Singla, N., Rani, S. R. A., & Kaur, B. (2014). Serum lipid profile in oral precancer and cancer: a diagnostic or prognostic marker? *Journal of International Oral Health : JIOH*, *6*(2), 33–39. http://www.ncbi.nlm.nih.gov/pubmed/24876700

Gassling, V., Hampe, J., Açil, Y., Braesen, J. H., Wiltfang, J., & Häsler, R. (2013). Disease-associated miRNA-mRNA networks in oral lichen planus. *PloS One*, *8*(5), e63015. https://doi.org/10.1371/journal.pone.0063015

Ghapanchi, J., Andisheh-Tadbir, A., Torkaman, P., Malekzadeh, M., & Mardani, M. (2019). Evaluation of the serum levels of galectin-3 in patients with oral lichen planus disease. *Oral Diseases*, *25*(2), 466–470. https://doi.org/10.1111/odi.13012

Ghapanchi, J., Ghaderi, H., Haghshenas, M. R., Jamshidi, S., Rezazadeh, F., Azad, A., Farzin, M., Derafshi, R., & Kalantari, A. H. (2019). Observational Molecular Case-Control Study of Genetic Polymorphisms 1 in Programmed Cell Death Protein-1 in Patients with Oral Lichen Planus. *Asian Pacific Journal of Cancer Prevention : APJCP*, *20*(2), 421–424. https://doi.org/10.31557/APJCP.2019.20.2.421

Ghapanchi, J., Haghshenas, M. R., Ghaderi, H., Amanpour, S., Nemati, V., & Kamali, F. (2014). Ctla-4 gene polymorphism in +49 a/g position: a case control study on patients with oral lichen planus. *Journal of International Oral Health : JIOH*, *6*(5), 17–21. http://www.ncbi.nlm.nih.gov/pubmed/25395787

Gholizadeh, N., Emami Razavi, A., Mohammadpour, H., Tavakol, F., & Sheykhbahaei, N. (2020). Association of MAPK and its regulatory miRNAs (603, 4301, 8485, and 4731) with the malignant transformation of oral lichen planus. *Molecular Biology Reports*, *47*(2), 1223–1232. https://doi.org/10.1007/s11033-019-05223-6

Giovannelli, L., Campisi, G., Colella, G., Capra, G., Di Liberto, C., Caleca, M. P., Matranga, D., D'Angelo, M., Lo Muzio, L., & Ammatuna, P. (2006). Brushing of oral mucosa for diagnosis of HPV infection in patients with potentially malignant and malignant oral lesions. *Molecular Diagnosis & Therapy*, *10*(1), 49–55. https://doi.org/10.1007/BF03256442

Gissi, D. B., Gabusi, A., Tarsitano, A., Asioli, S., Rossi, R., Marchetti, C., Montebugnoli, L., Foschini, M. P., & Morandi, L. (2020). Application of a non-invasive oral brushing procedure based on bisulfite sequencing of a 13-gene panel to study high-risk OSCC

patients. *Cancer Biomarkers : Section A of Disease Markers*, *28*(4), 499–510. https://doi.org/10.3233/CBM-190422

Goel, S., Marwah, A., Kaushik, S., Garg, V. K., & Gupta, S. (2015). Role of serum interleukin-6 in deciding therapy for multidrug resistant oral lichen planus. *Journal of Clinical and Experimental Dentistry*, *7*(4), e477-82. https://doi.org/10.4317/jced.52376

Gomez-Armayones, S., Chimenos-Küstner, E., Marí, A., Tous, S., Penin, R., Clavero, O., Quirós, B., Pavon, M. A., Taberna, M., Alemany, L., Servitje, O., & Mena, M. (2019). Human papillomavirus in premalignant oral lesions: No evidence of association in a Spanish cohort. *PloS One*, *14*(1), e0210070. https://doi.org/10.1371/journal.pone.0210070

Gonzalez Segura, I., Secchi, D., Carrica, A., Barello, R., Arbelo, D., Burgos, A., Brunotto, M., & Zarate, A. M. (2015). Exfoliative cytology as a tool for monitoring pre-malignant and malignant lesions based on combined stains and morphometry techniques. *Journal of Oral Pathology & Medicine : Official Publication of the International Association of Oral Pathologists and the American Academy of Oral Pathology*, *44*(3), 178–184. https://doi.org/10.1111/jop.12219

Gorugantula, L. M., Rees, T., Plemons, J., Chen, H.-S., & Cheng, Y.-S. L. (2012). Salivary basic fibroblast growth factor in patients with oral squamous cell carcinoma or oral lichen planus. *Oral Surgery, Oral Medicine, Oral Pathology and Oral Radiology*, *114*(2), 215–222. https://doi.org/10.1016/j.oooo.2012.03.013

Hadzi-Mihailovic, M., Stanimirovic, D., & Pasoski, B. (2020). Role of tumor suppressor protein p16 in patients with oral lichen planus. *Journal of B.U.ON. : Official Journal of the Balkan Union of Oncology*, *25*(2), 1193–1198. http://www.ncbi.nlm.nih.gov/pubmed/32521925

He, H., Xia, X., Yang, H., Peng, Q., & Zheng, J. (2020). A pilot study: a possible implication of Candida as an etiologically endogenous pathogen for oral lichen planus. *BMC Oral Health*, *20*(1), 72. https://doi.org/10.1186/s12903-020-1042-8

Hebbar, P. B., Pai, A., & D, S. (2013). Mycological and histological associations of Candida in oral mucosal lesions. *Journal of Oral Science*, *55*(2), 157–160. https://doi.org/10.2334/josnusd.55.157

Helm, T. N., Camisa, C., Liu, A. Y., Valenzuela, R., & Bergfeld, W. F. (1994). Lichen planus associated with neoplasia: a cell-mediated immune response to tumor antigens? *Journal of the American Academy of Dermatology*, *30*(2 Pt 1), 219–224. https://doi.org/10.1016/s0190-9622(94)70020-6

Hsu, H.-J., Yang, Y.-H., Shieh, T.-Y., Chen, C.-H., Kao, Y.-H., Yang, C.-F., & Ko, E. C.-C. (2014). Role of cytokine gene (interferon-γ, transforming growth factor-β1, tumor necrosis factor-α, interleukin-6, and interleukin-10) polymorphisms in the risk of oral precancerous lesions in Taiwanese. *The Kaohsiung Journal of Medical Sciences*, *30*(11), 551–558. https://doi.org/10.1016/j.kjms.2014.09.003

Ishikawa, S., Sugimoto, M., Edamatsu, K., Sugano, A., Kitabatake, K., & Iino, M. (2020).

Discrimination of oral squamous cell carcinoma from oral lichen planus by salivary metabolomics. *Oral Diseases*, *26*(1), 35–42. https://doi.org/10.1111/odi.13209

Jaafari-Ashkavandi, Z., & Fatemi, F.-S. (2013). Evaluation of proliferation activity in dysplastic and nondysplastic oral lichen planus through the analysis of argyrophilic nucleolar organizer regions. *The Journal of Craniofacial Surgery*, *24*(3), 788–791. https://doi.org/10.1097/SCS.0b013e31828b6e0e

Jablonska, E., Garley, M., Surazynski, A., Grubczak, K., Iwaniuk, A., Borys, J., Moniuszko, M., & Ratajczak-Wrona, W. (2020a). Neutrophil extracellular traps (NETs) formation induced by TGF-β in oral lichen planus - Possible implications for the development of oral cancer. *Immunobiology*, *225*(2), 151901. https://doi.org/10.1016/j.imbio.2019.151901

Jablonska, E., Garley, M., Surazynski, A., Grubczak, K., Iwaniuk, A., Borys, J., Moniuszko, M., & Ratajczak-Wrona, W. (2020b). Neutrophil extracellular traps (NETs) formation induced by TGF-β in oral lichen planus – Possible implications for the development of oral cancer. *Immunobiology*, *225*(2). https://doi.org/10.1016/j.imbio.2019.151901

Jainkittivong, A., Kuvatanasuchati, J., Pipattanagovit, P., & Sinheng, W. (2007). Candida in oral lichen planus patients undergoing topical steroid therapy. *Oral Surgery, Oral Medicine, Oral Pathology, Oral Radiology, and Endodontics*, *104*(1), 61–66. https://doi.org/10.1016/j.tripleo.2006.10.024

Jana, A., Thomas, J., & Ghosh, P. (2017). P-glycoprotein expression in oral lichen planus. *Brazilian Oral Research*, *31*, e95. https://doi.org/10.1590/1807-3107BOR-2017.vol31.0095

Jiang, C., Wei, W., Wang, Y., Song, C., Pan, L., Sun, K., Du, G., Deng, Y., & Tang, G. (2020). TRIM21 causes abnormal expression of IL-6 in oral lichen planus via the TRIB2-MAPK signal axis. *American Journal of Translational Research*, *12*(8), 4648–4658. http://www.ncbi.nlm.nih.gov/pubmed/32913538

Jontell, M., Watts, S., Wallström, M., Levin, L., & Sloberg, K. (1990). Human papilloma virus in erosive oral lichen planus. *Journal of Oral Pathology & Medicine : Official Publication of the International Association of Oral Pathologists and the American Academy of Oral Pathology*, *19*(6), 273–277. https://doi.org/10.1111/j.1600-0714.1990.tb00841.x

Juretić, M., Cerović, R., Belušić-Gobić, M., Brekalo Pršo, I., Kqiku, L., Špalj, S., & Pezelj-Ribarić, S. (2013). Salivary levels of TNF-α and IL-6 in patients with oral premalignant and malignant lesions. *Folia Biologica*, *59*(2), 99–102. http://www.ncbi.nlm.nih.gov/pubmed/23746176

Kämmerer, P. W., Koch, F. P., Santoro, M., Babaryka, G., Biesterfeld, S., Brieger, J., & Kunkel, M. (2013). Prospective, blinded comparison of cytology and DNA-image cytometry of brush biopsies for early detection of oral malignancy. *Oral Oncology*, *49*(5), 420–426. https://doi.org/10.1016/j.oraloncology.2012.12.006

Kaplan, I., Nabiochtchikov, I., Leshno, A., Moshkowitz, M., Shlomi, B., Kleinman, S., Dagan, Y., Meshiach, Y., Galazan, L., Arber, N., Avivi-Arber, L., & Kraus, S. (2015). Association of

CD24 and the adenomatous polyposis coli gene polymorphisms with oral lichen planus. *Oral Surgery, Oral Medicine, Oral Pathology and Oral Radiology*, *120*(3), 378–385. https://doi.org/10.1016/j.oooo.2015.05.015

Kaur, J., & Jacobs, R. (2015). Proinflammatory cytokine levels in oral lichen planus, oral leukoplakia, and oral submucous fibrosis. *Journal of the Korean Association of Oral and Maxillofacial Surgeons*, *41*(4), 171–175. https://doi.org/10.5125/jkaoms.2015.41.4.171

Kaur, J., Politis, C., & Jacobs, R. (2016). Salivary 8-hydroxy-2-deoxyguanosine, malondialdehyde, vitamin C, and vitamin E in oral pre-cancer and cancer: diagnostic value and free radical mechanism of action. *Clinical Oral Investigations*, *20*(2), 315–319. https://doi.org/10.1007/s00784-015-1506-4

Kawanishi, S., Hiraku, Y., Pinlaor, S., & Ma, N. (2006). Oxidative and nitrative DNA damage in animals and patients with inflammatory diseases in relation to inflammation-related carcinogenesis. *Biological Chemistry*, *387*(4), 365–372. https://doi.org/10.1515/BC.2006.049

Kazanowska-Dygdała, M., Duś, I., & Radwan-Oczko, M. (2016). The presence of Helicobacter pylori in oral cavities of patients with leukoplakia and oral lichen planus. *Journal of Applied Oral Science : Revista FOB*, *24*(1), 18–23. https://doi.org/10.1590/1678-775720150203

Ke, Y., Dang, E., Shen, S., Zhang, T., Qiao, H., Chang, Y., Liu, Q., & Wang, G. (2017). Semaphorin4D Drives CD8+ T-Cell Lesional Trafficking in Oral Lichen Planus via CXCL9/CXCL10 Upregulations in Oral Keratinocytes. *The Journal of Investigative Dermatology*, *137*(11), 2396–2406. https://doi.org/10.1016/j.jid.2017.07.818

Kho, H.-S., Chang, J.-Y., Kim, Y.-Y., & Kim, Y. (2013). MUC1 and Toll-like receptor-2 expression in burning mouth syndrome and oral lichen planus. *Archives of Oral Biology*, *58*(7), 837–842. https://doi.org/10.1016/j.archoralbio.2013.01.008

Kilpi, A., Rich, A. M., Konttinen, Y. T., & Reade, P. C. (1996). Expression of c-erbB-2 protein in keratinocytes of oral mucosal lichen planus and subsequent squamous cell carcinoma. *European Journal of Oral Sciences*, *104*(3), 278–284. https://doi.org/10.1111/j.1600-0722.1996.tb00078.x

Kim, J., Yook, J. I., Lee, E. H., Ryu, M. H., Yoon, J. H., Hong, J. C., Kim, D. J., & Kim, H. S. (2001). Evaluation of premalignant potential in oral lichen planus using interphase cytogenetics. *Journal of Oral Pathology & Medicine : Official Publication of the International Association of Oral Pathologists and the American Academy of Oral Pathology*, *30*(2), 65–72. https://doi.org/10.1034/j.1600-0714.2001.300201.x

Kimkong, I., Nakkuntod, J., Sodsai, P., Hirankarn, N., & Kitkumthorn, N. (2012). Association of interferon-gamma gene polymorphisms with susceptibility to oral lichen planus in the Thai population. *Archives of Oral Biology*, *57*(5), 491–494. https://doi.org/10.1016/j.archoralbio.2011.10.009

Kleier, C., Werkmeister, R., & Joos, U. (1998). [Zinc and vitamin A deficiency in diseases of the mouth mucosa]. *Mund-, Kiefer- Und Gesichtschirurgie : MKG*, *2*(6), 320–325. https://doi.org/10.1007/s100060050080

Kordbacheh, F., Bhatia, N., & Farah, C. S. (2016). Patterns of differentially expressed genes in oral mucosal lesions visualised under autofluorescence (VELscope(TM)). *Oral Diseases*, *22*(4), 285–296. https://doi.org/10.1111/odi.12438

Kövesi, G. (2001). [Evaluation of clinical data and immuno-modulating treatment of patients with oral lichen planus]. *Fogorvosi Szemle*, *94*(1), 9–14. http://www.ncbi.nlm.nih.gov/pubmed/11262802

Kragelund, C., Jensen, S. B., Hansen Cand Scient, C., Broesen, K., Torpet, L. A., & Reibel, J. (2014). Subgrouping of patients with oral lichen planus according to cytochrome P450 enzyme phenotype and genotype. *Oral Surgery, Oral Medicine, Oral Pathology and Oral Radiology*, *118*(4), 469–474. https://doi.org/10.1016/j.oooo.2014.06.008

Kragelund, C., Thomsen, C. E., Bardow, A., Pedersen, A. M., Nauntofte, B., Reibel, J., & Torpet, L. A. (2003). Oral lichen planus and intake of drugs metabolized by polymorphic cytochrome P450 enzymes. *Oral Diseases*, *9*(4), 177–187. https://doi.org/10.1034/j.1601-0825.2003.02892.x

Krishnamoorthy, B., Gn, S., N S, M., M B, S., & Garlapati, K. (2014). Lipid profile and metabolic syndrome status in patients with oral lichen planus, oral lichenoid reaction and healthy individuals attending a dental college in northern India - a descriptive study. *Journal of Clinical and Diagnostic Research : JCDR*, *8*(11), ZC92-5. https://doi.org/10.7860/JCDR/2014/9649.5188

Kujundzic, B., Zeljic, K., Supic, G., Magic, M., Stanimirovic, D., Ilic, V., Jovanovic, B., & Magic, Z. (2016). Association of vdr, cyp27b1, cyp24a1 and mthfr gene polymorphisms with oral lichen planus risk. *Clinical Oral Investigations*, *20*(4), 781–789. https://doi.org/10.1007/s00784-015-1572-7

Li, X.-Z., Yang, X.-Y., Wang, Y., Zhang, S.-N., Zou, W., Wang, Y., Li, X.-N., Wang, L.-S., Zhang, Z.-G., & Xie, L.-Z. (2017). Urine metabolic profiling for the pathogenesis research of erosive oral lichen planus. *Archives of Oral Biology*, *73*, 206–213. https://doi.org/10.1016/j.archoralbio.2016.10.014

Lisa Cheng, Y.-S., Jordan, L., Gorugantula, L. M., Schneiderman, E., Chen, H.-S., & Rees, T. (2014). Salivary interleukin-6 and -8 in patients with oral cancer and patients with chronic oral inflammatory diseases. *Journal of Periodontology*, *85*(7), 956–965. https://doi.org/10.1902/jop.2013.130320

Liu, J., Geng, F., Sun, H., Wang, X., Zhang, H., Yang, Q., & Zhang, J. (2018). Candida albicans induces TLR2/MyD88/NF-κB signaling and inflammation in oral lichen planus-derived keratinocytes. *Journal of Infection in Developing Countries*, *12*(9), 780–786. https://doi.org/10.3855/jidc.8062

Liu, Q., Wang, X., Liu, Y., Wei, M., & Chen, L. (2016). A combinative analysis of gene expression profiles and microRNA expression profiles identifies critical genes and microRNAs in oral lichen planus. *Archives of Oral Biology*, *68*, 61–65. https://doi.org/10.1016/j.archoralbio.2016.03.018

Llena-Puy, M. C., Montañana-Llorens, C., & Forner-Navarro, L. (2004). Optimal assay conditions for quantifying fibronectin in saliva. *Medicina Oral : Organo Oficial de La Sociedad Espanola de Medicina Oral y de La Academia Iberoamericana de Patologia y Medicina Bucal*, *9*(3), 191–196. http://www.ncbi.nlm.nih.gov/pubmed/15122119

Lodi, G., Carrozzo, M., Hallett, R., D'Amico, E., Piattelli, A., Teo, C. G., Gandolfo, S., Carbone, M., & Porter, S. R. (1997). HCV genotypes in Italian patients with HCV-related oral lichen planus. *Journal of Oral Pathology & Medicine : Official Publication of the International Association of Oral Pathologists and the American Academy of Oral Pathology*, *26*(8), 381–384. https://doi.org/10.1111/j.1600-0714.1997.tb00235.x

Lundegard, M., Nylander, K., & Danielsson, K. (2015). Difficulties detecting miRNA-203 in human whole saliva by the use of PCR. *Medicina Oral, Patologia Oral y Cirugia Bucal*, *20*(2), e130-4. https://doi.org/10.4317/medoral.20172

Lysitsa, S., Samson, J., Gerber-Wicht, C., Lang, U., & Lombardi, T. (2008). COX-2 expression in oral lichen planus. *Dermatology (Basel, Switzerland)*, *217*(2), 150–155. https://doi.org/10.1159/000137672

Madhulika, N., Rangdhol, R. V., Sitra, G., Ballaiah, J., Jaikumar, R. A., & Brooklyin, S. (2015). A case-control study to detect the extent of DNA damage in oral lichen planus and oral lichenoid reactions using comet assay. *Journal of Pharmacy & Bioallied Sciences*, *7*(Suppl 2), S451-6. https://doi.org/10.4103/0975-7406.163499

Maeda, H., Reibel, J., & Holmstrup, P. (1994). Keratin staining pattern in clinically normal and diseased oral mucosa of lichen planus patients. *Scandinavian Journal of Dental Research*, *102*(4), 210–215. https://doi.org/10.1111/j.1600-0722.1994.tb01182.x

Mansourian, A., Shanbehzadeh, N., Kia, S. J., & Moosavi, M.-S. (2017). Increased salivary aldehyde dehydrogenase 1 in non-reticular oral lichen planus. *Anais Brasileiros de Dermatologia*, *92*(2), 168–171. https://doi.org/10.1590/abd1806-4841.20174964

Maraki, D., Yalcinkaya, S., Pomjanski, N., Megahed, M., Boecking, A., & Becker, J. (2006). Cytologic and DNA-cytometric examination of oral lesions in lichen planus. *Journal of Oral Pathology & Medicine : Official Publication of the International Association of Oral Pathologists and the American Academy of Oral Pathology*, *35*(4), 227–232. https://doi.org/10.1111/j.1600-0714.2006.00401.x

Marshall, A., Celentano, A., Cirillo, N., McCullough, M., & Porter, S. (2017). Tissue-specific regulation of CXCL9/10/11 chemokines in keratinocytes: Implications for oral inflammatory disease. *PloS One*, *12*(3), e0172821. https://doi.org/10.1371/journal.pone.0172821

Marttila, E., Uittamo, J., Rusanen, P., Lindqvist, C., Salaspuro, M., & Rautemaa, R. (2013). Acetaldehyde production and microbial colonization in oral squamous cell carcinoma and oral lichenoid disease. *Oral Surgery, Oral Medicine, Oral Pathology and Oral Radiology*, *116*(1), 61–68. https://doi.org/10.1016/j.oooo.2013.02.009

Masson Regnault, M., Vigarios, E., Projetti, F., Herbault-Barres, B., Tournier, E., Lamant, L., & Sibaud, V. (2017). No detection of Merkel cell polyomavirus in oral lichen planus: Results of a preliminary study in a French cohort of patients. *Journal of Medical Virology*, *89*(11), 2055–2057. https://doi.org/10.1002/jmv.24887

Mattila, R., Alanen, K., & Syrjänen, S. (2004). DNA content as a prognostic marker of oral lichen planus with a risk of cancer development. *Analytical and Quantitative Cytology and Histology*, *26*(5), 278–284. http://www.ncbi.nlm.nih.gov/pubmed/15560534

Mattila, R., Rautava, J., & Syrjänen, S. (2012). Human papillomavirus in oral atrophic lichen planus lesions. *Oral Oncology*, *48*(10), 980–984. https://doi.org/10.1016/j.oraloncology.2012.04.009

Mehdipour, M., Shahidi, M., Manifar, S., Jafari, S., Mashhadi Abbas, F., Barati, M., Mortazavi, H., Shirkhoda, M., Farzanegan, A., & Elmi Rankohi, Z. (2018). Diagnostic and prognostic relevance of salivary microRNA-21, -125a, -31 and -200a levels in patients with oral lichen planus - a short report. *Cellular Oncology (Dordrecht)*, *41*(3), 329–334. https://doi.org/10.1007/s13402-018-0372-x

Messadi, D. V, Younai, F. S., Liu, H.-H., Guo, G., & Wang, C.-Y. (2014). The clinical effectiveness of reflectance optical spectroscopy for the in vivo diagnosis of oral lesions. *International Journal of Oral Science*, *6*(3), 162–167. https://doi.org/10.1038/ijos.2014.39

Mizukawa, N., Sugiyama, K., Ueno, T., Mishima, K., Takagi, S., & Sugahara, T. (1999). Defensin-1, an antimicrobial peptide present in the saliva of patients with oral diseases. *Oral Diseases*, *5*(2), 139–142. https://doi.org/10.1111/j.1601-0825.1999.tb00078.x

Momen-Heravi, F., Trachtenberg, A. J., Kuo, W. P., & Cheng, Y. S. (2014). Genomewide Study of Salivary MicroRNAs for Detection of Oral Cancer. *Journal of Dental Research*, *93*(7 Suppl), 86S-93S. https://doi.org/10.1177/0022034514531018

Montague, L. J., Bhattacharyya, I., Islam, M. N., Cohen, D. M., & Fitzpatrick, S. G. (2015). Direct immunofluorescence testing results in cases of premalignant and malignant oral lesions. *Oral Surgery, Oral Medicine, Oral Pathology and Oral Radiology*, *119*(6), 675–683. https://doi.org/10.1016/j.oooo.2015.02.478

Montebugnoli, L., Gissi, D. B., Scapoli, L., Palmieri, A., Morandi, L., Manelli, I., & Foschini, M. P. (2014). p16(INK4) expression is not associated with human papillomavirus in oral lichen planus. *Oral Surgery, Oral Medicine, Oral Pathology and Oral Radiology*, *118*(6), 694–702. https://doi.org/10.1016/j.oooo.2014.09.004

Morandi, L., Gissi, D., Tarsitano, A., Asioli, S., Gabusi, A., Marchetti, C., Montebugnoli, L., & Foschini, M. P. (2017). CpG location and methylation level are crucial factors for the early

detection of oral squamous cell carcinoma in brushing samples using bisulfite sequencing of a 13-gene panel. *Clinical Epigenetics*, *9*, 85. https://doi.org/10.1186/s13148-017-0386-7

Morandi, L., Gissi, D., Tarsitano, A., Asioli, S., Monti, V., Del Corso, G., Marchetti, C., Montebugnoli, L., & Foschini, M. P. (2015). DNA methylation analysis by bisulfite next-generation sequencing for early detection of oral squamous cell carcinoma and high-grade squamous intraepithelial lesion from oral brushing. *Journal of Cranio-Maxillo-Facial Surgery : Official Publication of the European Association for Cranio-Maxillo-Facial Surgery*, *43*(8), 1494–1500. https://doi.org/10.1016/j.jcms.2015.07.028

Nagao, T., Warnakulasuriya, S., Ikeda, N., Fukano, H., Yamamoto, S., Yano, M., Miyazaki, H., & Ito, Y. (2001). Serum antioxidant micronutrient levels in oral lichen planus. *Journal of Oral Pathology & Medicine : Official Publication of the International Association of Oral Pathologists and the American Academy of Oral Pathology*, *30*(5), 264–267. https://doi.org/10.1034/j.1600-0714.2001.300502.x

Nagao, Y., & Sata, M. (2012). A retrospective case-control study of hepatitis C virus infection and oral lichen planus in Japan: association study with mutations in the core and NS5A region of hepatitis C virus. *BMC Gastroenterology*, *12*, 31. https://doi.org/10.1186/1471-230X-12-31

Nagao, Y., Sata, M., Ide, T., Suzuki, H., Tanikawa, K., Itoh, K., & Kameyama, T. (1996). Development and exacerbation of oral lichen planus during and after interferon therapy for hepatitis C. *European Journal of Clinical Investigation*, *26*(12), 1171–1174. https://doi.org/10.1046/j.1365-2362.1996.610607.x

Nagao, Y., Sata, M., Noguchi, S., Seno'o, T., Kinoshita, M., Kameyama, T., & Ueno, T. (2000). Detection of hepatitis C virus RNA in oral lichen planus and oral cancer tissues. *Journal of Oral Pathology & Medicine : Official Publication of the International Association of Oral Pathologists and the American Academy of Oral Pathology*, *29*(6), 259–266. https://doi.org/10.1034/j.1600-0714.2000.290604.x

Nagao, Y., Sata, M., Noguchi, S., Suzuki, H., Mizokami, M., Kameyama, T., & Tanikawa, K. (1997). GB virus infection in patients with oral cancer and oral lichen planus. *Journal of Oral Pathology & Medicine : Official Publication of the International Association of Oral Pathologists and the American Academy of Oral Pathology*, *26*(3), 138–141. https://doi.org/10.1111/j.1600-0714.1997.tb00037.x

Nagao, Y., & Tanigawa, T. (2019). Red complex periodontal pathogens are risk factors for liver cirrhosis. *Biomedical Reports*, *11*(5), 199–206. https://doi.org/10.3892/br.2019.1245

Nagao, Y., Tsubone, K., Kimura, R., Hanada, S., Kumashiro, R., Ueno, T., & Sata, M. (2002). High prevalence of anticardiolipin antibodies in patients with HCV-associated oral lichen planus. *International Journal of Molecular Medicine*, *9*(3), 293–297. https://doi.org/10.3892/ijmm.9.3.293

Naik, S. R., Gupta, P., Khaitan, T., & Shukla, A. K. (2020). Reduced levels of serum vitamin B12

in symptomatic cases of oral lichen planus: A cross-sectional study. *Journal of Oral Biology and Craniofacial Research*, *10*(4), 578–582. https://doi.org/10.1016/j.jobcr.2020.07.010

Németh, C. G., Röcken, C., Siebert, R., Wiltfang, J., Ammerpohl, O., & Gassling, V. (2019). Recurrent chromosomal and epigenetic alterations in oral squamous cell carcinoma and its putative premalignant condition oral lichen planus. *PloS One*, *14*(4), e0215055. https://doi.org/10.1371/journal.pone.0215055

Nicolae, I., Mitran, C. I., Mitran, M. I., Ene, C. D., Tampa, M., & Georgescu, S. R. (2017). Ascorbic acid deficiency in patients with lichen planus. *Journal of Immunoassay & Immunochemistry*, *38*(4), 430–437. https://doi.org/10.1080/15321819.2017.1319863

Noguchi, H., Iwase, T., Omagari, D., Asano, M., Nakamura, R., Ueki, K., Shinozuka, K., Kaneko, T., Tonogi, M., & Ohki, H. (2017). Rapid detection of Candida albicans in oral exfoliative cytology samples by loop-mediated isothermal amplification. *Journal of Oral Science*, *59*(4), 541–547. https://doi.org/10.2334/josnusd.16-0717

Nosratzehi, F., Nosratzehi, T., Alijani, E., & Rad, S. S. (2020). Salivary β2-microglobulin levels in patients with erosive oral lichen planus and squamous cell carcinoma. *BMC Research Notes*, *13*(1), 294. https://doi.org/10.1186/s13104-020-05135-w

Nosratzehi, T., Alijani, E., & Moodi, M. (2017). Salivary MMP-1, MMP-2, MMP-3 and MMP-13 Levels in Patients with Oral Lichen Planus and Squamous Cell Carcinoma. *Asian Pacific Journal of Cancer Prevention : APJCP*, *18*(7), 1947–1951. https://doi.org/10.22034/APJCP.2017.18.7.1947

Nosratzehi, T., Risbaf Fakour, S., Alijani, E., & Salehi, M. (2017). Investigating the level of salivary endothelin-1 in premalignant and malignant lesions. *Special Care in Dentistry : Official Publication of the American Association of Hospital Dentists, the Academy of Dentistry for the Handicapped, and the American Society for Geriatric Dentistry*, *37*(3), 134–139. https://doi.org/10.1111/scd.12217

Nylander, E., Ebrahimi, M., Wahlin, Y.-B., Boldrup, L., & Nylander, K. (2012). Changes in miRNA expression in sera and correlation to duration of disease in patients with multifocal mucosal lichen planus. *Journal of Oral Pathology & Medicine : Official Publication of the International Association of Oral Pathologists and the American Academy of Oral Pathology*, *41*(1), 86–89. https://doi.org/10.1111/j.1600-0714.2011.01063.x

O'Flatharta, C., Leader, M., Kay, E., Flint, S. R., Toner, M., Robertson, W., & Mabruk, M. J. E. M. F. (2002). Telomerase activity detected in oral lichen planus by RNA in situ hybridisation: not a marker for malignant transformation. *Journal of Clinical Pathology*, *55*(8), 602–607. https://doi.org/10.1136/jcp.55.8.602

Ognjenović, M., Karelović, D., Cindro, V. V, & Tadin, I. (1998). Oral lichen planus and HLA A. *Collegium Antropologicum*, *22 Suppl*, 89–92. http://www.ncbi.nlm.nih.gov/pubmed/9951146

Ohashi, M., Iwase, M., & Nagumo, M. (1999). Elevated production of salivary nitric oxide in oral mucosal diseases. *Journal of Oral Pathology & Medicine : Official Publication of the International Association of Oral Pathologists and the American Academy of Oral Pathology*, *28*(8), 355–359. https://doi.org/10.1111/j.1600-0714.1999.tb02053.x

Ostwald, C., Rutsatz, K., Schweder, J., Schmidt, W., Gundlach, K., & Barten, M. (2003). Human papillomavirus 6/11, 16 and 18 in oral carcinomas and benign oral lesions. *Medical Microbiology and Immunology*, *192*(3), 145–148. https://doi.org/10.1007/s00430-002-0161-y

Pandey, M., Prakash, O., Santhi, W. S., Soumithran, C. S., & Pillai, R. M. (2008). Overexpression of COX-2 gene in oral cancer is independent of stage of disease and degree of differentiation. *International Journal of Oral and Maxillofacial Surgery*, *37*(4), 379–383. https://doi.org/10.1016/j.ijom.2008.01.004

Pavelic, J., Gall-Troselj, K., Mravak-Stipetic, M., & Pavelic, K. (1998). The p53 and nm23-H1 genes are not deleted in oral benign epithelial lesions. *Anticancer Research*, *18*(5A), 3527–3531. http://www.ncbi.nlm.nih.gov/pubmed/9858935

Peng, Q., Zhang, J., & Zhou, G. (2018). Differentially circulating exosomal microRNAs expression profiling in oral lichen planus. *American Journal of Translational Research*, *10*(9), 2848–2858. http://www.ncbi.nlm.nih.gov/pubmed/30323871

Peng, Q., Zhang, J., & Zhou, G. (2019). Circulating exosomes regulate T-cell-mediated inflammatory response in oral lichen planus. *Journal of Oral Pathology & Medicine : Official Publication of the International Association of Oral Pathologists and the American Academy of Oral Pathology*, *48*(2), 143–150. https://doi.org/10.1111/jop.12804

Pentenero, M., Monticone, M., Marino, R., Aiello, C., Marchitto, G., Malacarne, D., Giaretti, W., Gandolfo, S., & Castagnola, P. (2017). High-resolution DNA content analysis of microbiopsy samples in oral lichen planus. *Oral Diseases*, *23*(3), 318–323. https://doi.org/10.1111/odi.12605

Perdigão, P. F., Guimarães, A. L. S., Victoria, J. M. N., Xavier, G. M., Romano-Silva, M. A., & Gomez, R. S. (2007). Serotonin transporter gene polymorphism (5-HTTLPR) in patients with oral lichen planus. *Archives of Oral Biology*, *52*(9), 889–893. https://doi.org/10.1016/j.archoralbio.2007.02.001

Polesello, V., Segat, L., Biasotto, M., Ottaviani, G., Gobbo, M., Di Lenarda, R., Crovella, S., & Zupin, L. (2019). Mannose-Binding Lectin 2 (MBL2) combined genotypes deficiency is associated with susceptibility for Oral Lichen Planus. *Genetics and Molecular Biology*, *42*(1), 9–14. https://doi.org/10.1590/1678-4685-GMB-2018-0015

Popovska, M., Atanasovska-Stojanovska, A., Todoroska, S., Radojkova-Nikolovska, V., Bedhxeti, L. Z., Spasovska-Gjorgovska, A., Spasovski, S., & Ivanovska-Stojanoska, M. (2020). Oral Lichen Planus - Related Connection with HLA-System Antigens. *Prilozi (Makedonska Akademija Na Naukite i Umetnostite. Oddelenie Za Medicinski Nauki)*, *41*(1), 65–77. https://doi.org/10.2478/prilozi-2020-0024

Pourshahidi, S., Fakhri, F., Ebrahimi, H., Fakhraei, B., Alipour, A., Ghapanchi, J., & Farjadian, S. (2012). Lack of association between Helicobacter pylori infection and oral lichen planus. *Asian Pacific Journal of Cancer Prevention : APJCP*, *13*(5), 1745–1747. https://doi.org/10.7314/apjcp.2012.13.5.1745

Prasad, G., Seers, C., Reynolds, E., & McCullough, M. J. (2017). A panel of microRNAs can be used to determine oral squamous cell carcinoma. *Journal of Oral Pathology & Medicine : Official Publication of the International Association of Oral Pathologists and the American Academy of Oral Pathology*, *46*(10), 940–948. https://doi.org/10.1111/jop.12592

Pursanova, A. E., Kazarina, L. N., Guschina, O. O., Serhel, E. V, Belozyorov, A. E., & Abaev, Z. M. (2018). [Clinical and immunological features of oral and vermillion border precancer diseases]. *Stomatologiia*, *97*(5), 23–26. https://doi.org/10.17116/stomat20189705123

Rai, B., Kaur, J., Jacobs, R., & Singh, J. (2010). Possible action mechanism for curcumin in pre-cancerous lesions based on serum and salivary markers of oxidative stress. *Journal of Oral Science*, *52*(2), 251–256. https://doi.org/10.2334/josnusd.52.251

Rao, D. S., Ali, I. M., & Annigeri, R. G. (2017). Evaluation of diagnostic value of AgNOR and PAP in early detection of dysplastic changes in leukoplakia and lichen planus - a preliminary case-control study. *Journal of Oral Pathology & Medicine : Official Publication of the International Association of Oral Pathologists and the American Academy of Oral Pathology*, *46*(1), 56–60. https://doi.org/10.1111/jop.12457

Rashed, L., Abdel Hay, R., AlKaffas, M., Ali, S., Kadry, D., & Abdallah, S. (2017). Studying the association between methylenetetrahydrofolate reductase (MTHFR) 677 gene polymorphism, cardiovascular risk and lichen planus. *Journal of Oral Pathology & Medicine : Official Publication of the International Association of Oral Pathologists and the American Academy of Oral Pathology*, *46*(10), 1023–1029. https://doi.org/10.1111/jop.12588

Rezazadeh, F., Salehi, S., & Rezaee, M. (2019). Salivary Level of Trace Element in Oral Lichen Planus, A Premalignant Condition. *Asian Pacific Journal of Cancer Prevention : APJCP*, *20*(7), 2009–2013. https://doi.org/10.31557/APJCP.2019.20.7.2009

Rhodus, N. L., Cheng, B., Myers, S., Miller, L., Ho, V., & Ondrey, F. (2005). The feasibility of monitoring NF-kappaB associated cytokines: TNF-alpha, IL-1alpha, IL-6, and IL-8 in whole saliva for the malignant transformation of oral lichen planus. *Molecular Carcinogenesis*, *44*(2), 77–82. https://doi.org/10.1002/mc.20113

Riggio, M. P., Lennon, A., & Wray, D. (2000). Detection of Helicobacter pylori DNA in recurrent aphthous stomatitis tissue by PCR. *Journal of Oral Pathology & Medicine : Official Publication of the International Association of Oral Pathologists and the American Academy of Oral Pathology*, *29*(10), 507–513. https://doi.org/10.1034/j.1600-0714.2000.291005.x

Rode, M., Flezar, M. S., Kogoj-Rode, M., & Us-Krasovec, M. (2006). Image cytometric evaluation of nuclear texture features and DNA content of the reticular form of oral

lichen planus. *Analytical and Quantitative Cytology and Histology*, *28*(5), 262–268. http://www.ncbi.nlm.nih.gov/pubmed/17067008

Sahebjamiee, M., Sand, L., Karimi, S., Biettolahi, J. M., Jabalameli, F., & Jalouli, J. (2015). Prevalence of human papillomavirus in oral lichen planus in an Iranian cohort. *Journal of Oral and Maxillofacial Pathology : JOMFP*, *19*(2), 170–174. https://doi.org/10.4103/0973-029X.164528

Salem, A., Almahmoudi, R., Hagström, J., Stark, H., Nordström, D., Salo, T., & Eklund, K. K. (2019). Human β-Defensin 2 Expression in Oral Epithelium: Potential Therapeutic Targets in Oral Lichen Planus. *International Journal of Molecular Sciences*, *20*(7). https://doi.org/10.3390/ijms20071780

Salem, A., Almahmoudi, R., Vehviläinen, M., & Salo, T. (2018). Role of the high mobility group box 1 signalling axes via the receptor for advanced glycation end-products and toll-like receptor-4 in the immunopathology of oral lichen planus: a potential drug target? *European Journal of Oral Sciences*, *126*(3), 244–248. https://doi.org/10.1111/eos.12416

Sánchez-Siles, M., Aliaga-Sánchez, A., Medina, S., Adoamnei, E., Fernández-Ruiz, J. A., Pelegrín-Hernández, J. P., Corno-Caparrós, A., Rosa-Salazar, V., & Camacho-Alonso, F. (2019). Genotyping of the C>T allele of rs16906252, predictor of O16-methylguanine-DNA methyltransferase (MGMT) promoter methylation status, in erosive atrophic lesions of oral lichen planus. *International Journal of Dermatology*, *58*(9), 1078–1082. https://doi.org/10.1111/ijd.14473

Sand, L., Jalouli, J., Larsson, P. A., & Hirsch, J. M. (2000). Human papilloma viruses in oral lesions. *Anticancer Research*, *20*(2B), 1183–1188. http://www.ncbi.nlm.nih.gov/pubmed/10810419

Sand, L. P., Jalouli, J., Larsson, P.-A., & Hirsch, J.-M. (2002). Prevalence of Epstein-Barr virus in oral squamous cell carcinoma, oral lichen planus, and normal oral mucosa. *Oral Surgery, Oral Medicine, Oral Pathology, Oral Radiology, and Endodontics*, *93*(5), 586–592. https://doi.org/10.1067/moe.2002.124462

Saruhanoğlu, A., Ergun, S., Kaya, M., Warnakulasuriya, S., Erbağcı, M., Öztürk, Ş., Deniz, E., Özel, S., Çefle, K., Palanduz, Ş., & Tanyeri, H. (2014). Evaluation of micronuclear frequencies in both circulating lymphocytes and buccal epithelial cells of patients with oral lichen planus and oral lichenoid contact reactions. *Oral Diseases*, *20*(5), 521–527. https://doi.org/10.1111/odi.12169

Segura, S., Rozas-Muñoz, E., Toll, A., Martín-Ezquerra, G., Masferrer, E., Espinet, B., Rodriguez, M., Baró, T., Barranco, C., & Pujol, R. M. (2013). Evaluation of MYC status in oral lichen planus in patients with progression to oral squamous cell carcinoma. *The British Journal of Dermatology*, *169*(1), 106–114. https://doi.org/10.1111/bjd.12303

Shariati, M., Mokhtari, M., & Masoudifar, A. (2018). Association between oral lichen planus and Epstein-Barr virus in Iranian patients. *Journal of Research in Medical Sciences : The Official Journal of Isfahan University of Medical Sciences*, *23*, 24.

https://doi.org/10.4103/jrms.JRMS_438_17

Sharma, R., Sircar, K., Singh, S., & Rastogi, V. (2011). Role of mast cells in pathogenesis of oral lichen planus. *Journal of Oral and Maxillofacial Pathology : JOMFP*, *15*(3), 267–271. https://doi.org/10.4103/0973-029X.86674

Shen, H., Liu, Q., Huang, P., Fan, H., Zang, F., Liu, M., Zhuo, L., Wu, J., Wu, G., Yu, R., & Yang, J. (2020). Vitamin D receptor genetic polymorphisms are associated with oral lichen planus susceptibility in a Chinese Han population. *BMC Oral Health*, *20*(1), 26. https://doi.org/10.1186/s12903-020-1002-3

Shi, Y., Shen, L., & Yin, C. (2010). [Expression of caspase-8, receptor interacting protein and nuclear factor-kappaBp65 in oral lichen planus]. *Zhonghua Kou Qiang Yi Xue Za Zhi = Zhonghua Kouqiang Yixue Zazhi = Chinese Journal of Stomatology*, *45*(1), 11–15. http://www.ncbi.nlm.nih.gov/pubmed/20368033

Shin, J.-A., Seo, J.-M., Oh, S., Cho, S.-D., & Lee, K.-E. (2016). Myeloid cell leukemia-1 is a molecular indicator for malignant transformation of oral lichen planus. *Oncology Letters*, *11*(2), 1603–1607. https://doi.org/10.3892/ol.2016.4083

Shirol, P. D., Naik, V., & Kale, A. (2015). Fibrinogen Demonstration in Oral Lichen Planus: An Immunofluorescence Study on Archival Tissues. *The Journal of Contemporary Dental Practice*, *16*(10), 824–828. https://doi.org/10.5005/jp-journals-10024-1764

Shirzad, A., Pouramir, M., Seyedmajidi, M., Jenabian, N., Bijani, A., & Motallebnejad, M. (2014). Salivary total antioxidant capacity and lipid peroxidation in patients with erosive oral lichen planus. *Journal of Dental Research, Dental Clinics, Dental Prospects*, *8*(1), 35–39. https://doi.org/10.5681/joddd.2014.006

Shiva, A., Arab, S., Mousavi, S. J., Zamanian, A., & Maboudi, A. (2020). Serum and Salivary Level of Nitric Oxide (NOx) and CRP in Oral Lichen Planus (OLP) Patients. *Journal of Dentistry (Shiraz, Iran)*, *21*(1), 6–11. https://doi.org/10.30476/DENTJODS.2019.77842

Silverman, S., Gorsky, M., & Lozada-Nur, F. (1985). A prospective follow-up study of 570 patients with oral lichen planus: persistence, remission, and malignant association. *Oral Surgery, Oral Medicine, and Oral Pathology*, *60*(1), 30–34. https://doi.org/10.1016/0030-4220(85)90210-5

Simark-Mattsson, C., & Eklund, C. (2013). Reduced immune responses to purified protein derivative and Candida albicans in oral lichen planus. *Journal of Oral Pathology & Medicine : Official Publication of the International Association of Oral Pathologists and the American Academy of Oral Pathology*, *42*(9), 691–697. https://doi.org/10.1111/jop.12069

Sperandio, M., Klinikowski, M. F., Brown, A. L., Shirlaw, P. J., Challacombe, S. J., Morgan, P. R., Warnakulasuriya, S., & Odell, E. W. (2016). Image-based DNA ploidy analysis aids prediction of malignant transformation in oral lichen planus. *Oral Surgery, Oral Medicine, Oral Pathology and Oral Radiology*, *121*(6), 643–650.

https://doi.org/10.1016/j.oooo.2016.02.008

Srisuttee, R., Arayataweegool, A., Mahattanasakul, P., Tangjaturonrasme, N., Kerekhanjanarong, V., Keelawat, S., Mutirangura, A., & Kitkumthorn, N. (2020). Evaluation of NID2 promoter methylation for screening of Oral squamous cell carcinoma. *BMC Cancer*, *20*(1), 218. https://doi.org/10.1186/s12885-020-6692-z

Stanimirovic, D., Zeljic, K., Jankovic, L., Magic, M., Hadzi-Mihajlovic, M., & Magic, Z. (2013). TLR2, TLR3, TLR4 and CD14 gene polymorphisms associated with oral lichen planus risk. *European Journal of Oral Sciences*, *121*(5), 421–426. https://doi.org/10.1111/eos.12074

Sun, A., & Chiang, C. P. (2001). Levamisole and/or Chinese medicinal herbs can modulate the serum level of squamous cell carcinoma associated antigen in patients with erosive oral lichen planus. *Journal of Oral Pathology & Medicine : Official Publication of the International Association of Oral Pathologists and the American Academy of Oral Pathology*, *30*(9), 542–548. https://doi.org/10.1034/j.1600-0714.2001.300906.x

Supic, G., Kozomara, R., Zeljic, K., Stanimirovic, D., Magic, M., Surbatovic, M., Jovic, N., & Magic, Z. (2015). HMGB1 genetic polymorphisms in oral squamous cell carcinoma and oral lichen planus patients. *Oral Diseases*, *21*(4), 536–543. https://doi.org/10.1111/odi.12318

Szarka, K., Tar, I., Fehér, E., Gáll, T., Kis, A., Tóth, E. D., Boda, R., Márton, I., & Gergely, L. (2009). Progressive increase of human papillomavirus carriage rates in potentially malignant and malignant oral disorders with increasing malignant potential. *Oral Microbiology and Immunology*, *24*(4), 314–318. https://doi.org/10.1111/j.1399-302X.2009.00516.x

Tabata, M., Yonezawa, S., Sugihara, K., Yamashita, S., & Maruyama, I. (1995). The use of thrombomodulin to study epithelial cell differentiation in neoplastic and non-neoplastic oral lesions. *Journal of Oral Pathology & Medicine : Official Publication of the International Association of Oral Pathologists and the American Academy of Oral Pathology*, *24*(10), 443–449. https://doi.org/10.1111/j.1600-0714.1995.tb01131.x

Tabatabaei, S. H., Sheikhha, M. H., Karbasi, M. H. A., Zarmehi, S., & Hoseini, M. (2018). Evaluation of polymorphism of P53 protein codon 72 in oral lichen planus by PCR technique. *Journal of Dental Research, Dental Clinics, Dental Prospects*, *12*(4), 245–251. https://doi.org/10.15171/joddd.2018.038

Tarsariya, V. M., Mehta, D. N., Raval, N., Patadiya, H. H., Vachhrajani, K., & Ashem, A. (2020). Evaluation of serum immunoglobulin (IgG, IgM, IgA) in potentially malignant disorders of oral cavity - A case control study. *Journal of Oral Biology and Craniofacial Research*, *10*(4), 665–669. https://doi.org/10.1016/j.jobcr.2020.09.002

Teja, C. S. R., Devy, A. S., Nirmal, R. M., Sunil, P. M., & Deepasree, M. (2014). Cytomorphometric analysis of exfoliated cells in oral lichen planus. *CytoJournal*, *11*, 3. https://doi.org/10.4103/1742-6413.127214

Thongprasom, K., Mutirangura, A., & Cheerat, S. (1998). Telomerase activity in oral lichen

planus. *Journal of Oral Pathology & Medicine : Official Publication of the International Association of Oral Pathologists and the American Academy of Oral Pathology*, *27*(8), 395–398. https://doi.org/10.1111/j.1600-0714.1998.tb01973.x

Thongprasom, K., Youngnak, P., & Aneksuk, V. (2001). Folate and vitamin B12 levels in patients with oral lichen planus, stomatitis or glossitis. *The Southeast Asian Journal of Tropical Medicine and Public Health*, *32*(3), 643–647. http://www.ncbi.nlm.nih.gov/pubmed/11944731

Tiwari, R., David, C. M., Mahesh, D. R., Sambargi, U., Rashmi, K. J., & Benakanal, P. (2016). Assessment of serum copper, iron and immune complexes in potentially malignant disorders and oral cancer. *Brazilian Oral Research*, *30*(1), e101. https://doi.org/10.1590/1807-3107BOR-2016.vol30.0101

Totan, A., Miricescu, D., Parlatescu, I., Mohora, M., & Greabu, M. (2015). Possible salivary and serum biomarkers for oral lichen planus. *Biotechnic & Histochemistry : Official Publication of the Biological Stain Commission*, *90*(7), 552–558. https://doi.org/10.3109/10520295.2015.1016115

Uma Maheswari, T. N., Nivedhitha, M. S., & Ramani, P. (2020). Expression profile of salivary micro RNA-21 and 31 in oral potentially malignant disorders. *Brazilian Oral Research*, *34*, e002. https://doi.org/10.1590/1807-3107bor-2020.vol34.0002

Vankadara, S., K, P., Balmuri, P. K., G, N., & G, V. R. (2018). Evaluation of Serum C-Reactive Protein Levels in Oral Premalignancies and Malignancies: A Comparative Study. *Journal of Dentistry (Tehran, Iran)*, *15*(6), 358–364. http://www.ncbi.nlm.nih.gov/pubmed/30842796

Venkatesiah, S. S., Kale, A. D., Hallikeremath, S. R., & Kotrashetti, V. S. (2013). Histomorphometric analysis of nuclear and cellular volumetric alterations in oral lichen planus, lichenoid lesions and normal oral mucosa using image analysis software. *Indian Journal of Dental Research : Official Publication of Indian Society for Dental Research*, *24*(2), 277. https://doi.org/10.4103/0970-9290.116678

Vesper, M., Riethdorf, S., Christoph, E., Ruthke, A., Schmelzle, R., & Löning, T. (1997). [Detection of human papillomavirus (HVP)-DNA in oral manifestation of lichen planus]. *Mund-, Kiefer- Und Gesichtschirurgie : MKG*, *1*(3), 146–149. https://doi.org/10.1007/BF03043534

Vohra, S., Singal, A., & Sharma, S. B. (2016). Clinical and serological efficacy of topical calcineurin inhibitors in oral lichen planus: a prospective randomized controlled trial. *International Journal of Dermatology*, *55*(1), 101–105. https://doi.org/10.1111/ijd.12887

Wang, J., Yang, L., Wang, L., Yang, Y., & Wang, Y. (2018). Forkhead box p3 controls progression of oral lichen planus by regulating microRNA-146a. *Journal of Cellular Biochemistry*, *119*(11), 8862–8871. https://doi.org/10.1002/jcb.27139

Wang, J., Zhai, X., Guo, J., Li, Y., Yang, Y., Wang, L., Yang, L., & Liu, F. (2019). Long non-coding

RNA DQ786243 modulates the induction and function of CD4+ Treg cells through Foxp3-miR-146a-NF-κB axis: Implications for alleviating oral lichen planus. *International Immunopharmacology*, *75*, 105761. https://doi.org/10.1016/j.intimp.2019.105761

Wang, K., Lu, W., Tu, Q., Ge, Y., He, J., Zhou, Y., Gou, Y., Van Nostrand, J. D., Qin, Y., Li, J., Zhou, J., Li, Y., Xiao, L., & Zhou, X. (2016). Preliminary analysis of salivary microbiome and their potential roles in oral lichen planus. *Scientific Reports*, *6*, 22943. https://doi.org/10.1038/srep22943

Wang, X., Tang, G., & Sun, H. (2015). [Effect of hypoxia on the proliferation and expressions of hypoxia-inducible factor-1α, vascular endothelial growth factor and matrix metalloproteinase-9 in keratinocytes obtained from oral lichen planus lesions]. *Zhonghua Kou Qiang Yi Xue Za Zhi = Zhonghua Kouqiang Yixue Zazhi = Chinese Journal of Stomatology*, *50*(2), 89–94. http://www.ncbi.nlm.nih.gov/pubmed/25908192

Watanabe, T., Ohishi, M., Tanaka, K., & Sato, H. (1986). Analysis of HLA antigens in Japanese with oral lichen planus. *Journal of Oral Pathology*, *15*(10), 529–533. https://doi.org/10.1111/j.1600-0714.1986.tb00571.x

Werneck, J. T., Costa, T. de O., Stibich, C. A., Leite, C. A., Dias, E. P., & Silva Junior, A. (2015). Oral lichen planus: study of 21 cases. *Anais Brasileiros de Dermatologia*, *90*(3), 321–326. https://doi.org/10.1590/abd1806-4841.20153704

White, F. H., Jin, Y., & Yang, L. (1994). Quantitative cellular and nuclear volumetric alterations in epithelium from lichen planus lesions of human buccal mucosa. *Journal of Oral Pathology & Medicine : Official Publication of the International Association of Oral Pathologists and the American Academy of Oral Pathology*, *23*(5), 205–208. https://doi.org/10.1111/j.1600-0714.1994.tb01114.x

Wu, D., Chen, X., Dong, C., Liu, Q., Yang, Y., He, C., Wang, J., Sun, M., & Wu, Y. (2015). Association of single nucleotide polymorphisms in MPO and COX genes with oral lichen planus. *International Journal of Immunogenetics*, *42*(3), 161–167. https://doi.org/10.1111/iji.12193

Xavier, G. M., de Sá, A. R., Guimarães, A. L. S., da Silva, T. A., & Gomez, R. S. (2007). Investigation of functional gene polymorphisms interleukin-1beta, interleukin-6, interleukin-10 and tumor necrosis factor in individuals with oral lichen planus. *Journal of Oral Pathology & Medicine : Official Publication of the International Association of Oral Pathologists and the American Academy of Oral Pathology*, *36*(8), 476–481. https://doi.org/10.1111/j.1600-0714.2007.00560.x

Yadav, M., Arivananthan, M., Chandrashekran, A., Tan, B. S., & Hashim, B. Y. (1997). Human herpesvirus-6 (HHV-6) DNA and virus-encoded antigen in oral lesions. *Journal of Oral Pathology & Medicine : Official Publication of the International Association of Oral Pathologists and the American Academy of Oral Pathology*, *26*(9), 393–401. https://doi.org/10.1111/j.1600-0714.1997.tb00238.x

Yahalom, R., Yarom, N., Shani, T., Amariglio, N., Kaplan, I., Trakhtenbrot, L., & Hirshberg, A.

(2016). Oral lichen planus patients exhibit consistent chromosomal numerical aberrations: A follow-up analysis. *Head & Neck*, *38 Suppl 1*, E741-6. https://doi.org/10.1002/hed.24086

Yamamoto, T., Yoneda, K., Ueta, E., Hirota, J., & Osaki, T. (1991). Serum cytokine levels in patients with oral mucous membrane disorders. *Journal of Oral Pathology & Medicine : Official Publication of the International Association of Oral Pathologists and the American Academy of Oral Pathology*, *20*(6), 275–279. https://doi.org/10.1111/j.1600-0714.1991.tb00927.x

Yamamoto, T., Yoneda, K., Ueta, E., & Osaki, T. (1994). Serum cytokines, interleukin-2 receptor, and soluble intercellular adhesion molecule-1 in oral disorders. *Oral Surgery, Oral Medicine, and Oral Pathology*, *78*(6), 727–735. https://doi.org/10.1016/0030-4220(94)90087-6

Yan, S.-K., Wei, B.-J., Lin, Z.-Y., Yang, Y., Zhou, Z.-T., & Zhang, W.-D. (2008). A metabonomic approach to the diagnosis of oral squamous cell carcinoma, oral lichen planus and oral leukoplakia. *Oral Oncology*, *44*(5), 477–483. https://doi.org/10.1016/j.oraloncology.2007.06.007

Yang, Q., Guo, B., Sun, H., Zhang, J., Liu, S., Hexige, S., Yu, X., & Wang, X. (2017). Identification of the key genes implicated in the transformation of OLP to OSCC using RNA-sequencing. *Oncology Reports*, *37*(4), 2355–2365. https://doi.org/10.3892/or.2017.5487

Yang, Q., Sun, H., Wang, X., Yu, X., Zhang, J., Guo, B., & Hexige, S. (2020). Metabolic changes during malignant transformation in primary cells of oral lichen planus: Succinate accumulation and tumour suppression. *Journal of Cellular and Molecular Medicine*, *24*(2), 1179–1188. https://doi.org/10.1111/jcmm.14376

Yang, Q., Xu, B., Sun, H., Wang, X., Zhang, J., Yu, X., & Ma, X. (2017). A genome-wide association scan of biological processes involved in oral lichen planus and oral squamous cell carcinoma. *Medicine*, *96*(25), e7012.https://doi.org/10.1097/MD.0000000000007012

Yang, X.-Y., Li, X.-Z., & Zhang, S.-N. (2018). Metabolomics analysis of oral mucosa reveals profile perturbation in reticular oral lichen planus. *Clinica Chimica Acta; International Journal of Clinical Chemistry*, *487*, 28–32. https://doi.org/10.1016/j.cca.2018.09.021

Yang, X.-Y., Zhang, S.-N., Li, X.-Z., Wang, Y., & Yin, X.-D. (2017). Analysis of human serum metabolome for potential biomarkers identification of erosive oral lichen planus. *Clinica Chimica Acta; International Journal of Clinical Chemistry*, *468*, 46–50. https://doi.org/10.1016/j.cca.2017.02.010

Yao, H., Wu, B., & Wu, Q. (1999). [Study and detection of telomerase activity in oral squamous cell carcinomas and precancerous lesions]. *Zhonghua Kou Qiang Yi Xue Za Zhi = Zhonghua Kouqiang Yixue Zazhi = Chinese Journal of Stomatology*, *34*(6), 328–330. http://www.ncbi.nlm.nih.gov/pubmed/11776870

Yao, H., & Wu, B. X. (1998). [The content of estradiol receptors in oral mucosa precancerous

lesions and its effect]. *Shanghai Kou Qiang Yi Xue = Shanghai Journal of Stomatology*, *7*(4), 204–206. http://www.ncbi.nlm.nih.gov/pubmed/15071625

Yarom, N., Shani, T., Amariglio, N., Taicher, S., Kaplan, I., Vered, M., Rechavi, G., Trakhtenbrot, L., & Hirshberg, A. (2009). Chromosomal numerical aberrations in oral lichen planus. *Journal of Dental Research*, *88*(5), 427–432. https://doi.org/10.1177/0022034509337089

Ye, X., Zhang, J., Lu, R., & Zhou, G. (2014). HBO: a possible supplementary therapy for oral potentially malignant disorders. *Medical Hypotheses*, *83*(2), 131–136. https://doi.org/10.1016/j.mehy.2014.05.011

Zarate, A. M., Don, J., Secchi, D., Carrica, A., Galindez Costa, F., Panico, R., Brusa, M., Barra, J. L., & Brunotto, M. (2017). Study of the TP53 codon 72 polymorphism in oral cancer and oral potentially malignant disorders in Argentine patients. *Tumour Biology : The Journal of the International Society for Oncodevelopmental Biology and Medicine*, *39*(5), 1010428317699113. https://doi.org/10.1177/1010428317699113

Zeng, X., Chen, Q., Nie, M., & Li, B. (2004). [The attribute of Candida albicans isolates from patients with oral lichen planus]. *Zhonghua Kou Qiang Yi Xue Za Zhi = Zhonghua Kouqiang Yixue Zazhi = Chinese Journal of Stomatology*, *39*(2), 149–152. http://www.ncbi.nlm.nih.gov/pubmed/15061893

Zeng, X., Chen, Q., Nie, M., & Li, B. (2005a). [Adhesion to buccal epithelial cells of Candida albicans isolates from oral lichen planus]. *Hua Xi Kou Qiang Yi Xue Za Zhi = Huaxi Kouqiang Yixue Zazhi = West China Journal of Stomatology*, *23*(6), 537–538. http://www.ncbi.nlm.nih.gov/pubmed/16430191

Zeng, X., Chen, Q., Nie, M., & Li, B. (2005b). [The genotypic profiles of Candida albicans isolates from patients with oral lichen planus]. *Sichuan Da Xue Xue Bao. Yi Xue Ban = Journal of Sichuan University. Medical Science Edition*, *36*(2), 193–195. http://www.ncbi.nlm.nih.gov/pubmed/15807264

Zeng, X., Xiong, C., Wang, Z., Jiang, L., Hou, X., Shen, J., Zhou, M., & Chen, Q. (2008). Genotypic profiles and virulence attributes of Candida albicans isolates from patients with oral lichen planus. *APMIS : Acta Pathologica, Microbiologica, et Immunologica Scandinavica*, *116*(4), 284–291. https://doi.org/10.1111/j.1600-0463.2008.00741.x

Zhang, L., Cheng, X., Li, Y., Poh, C., Zeng, T., Priddy, R., Lovas, J., Freedman, P., Daley, T., & Rosin, M. P. (2000). High frequency of allelic loss in dysplastic lichenoid lesions. *Laboratory Investigation; a Journal of Technical Methods and Pathology*, *80*(2), 233–237. https://doi.org/10.1038/labinvest.3780026

Zhang, L., Mao, E., Priddy, R., & Rosin, M. (1996). p53 overexpression in oral lichen planus. *Oncology Reports*, *3*(6), 1145–1148. https://doi.org/10.3892/or.3.6.1145

Zhang, L., Michelsen, C., Cheng, X., Zeng, T., Priddy, R., & Rosin, M. P. (1997). Molecular analysis of oral lichen planus. A premalignant lesion? *The American Journal of Pathology*, *151*(2), 323–327. http://www.ncbi.nlm.nih.gov/pubmed/9250145

11.3 List S4. Overlapping population (n=9)

Bascones-Ilundain, C., Gonzalez-Moles, M. A., Esparza-Gómez, G., Gil-Montoya, J. A., & Bascones-Martínez, A. (2006). Importance of apoptotic mechanisms in inflammatory infiltrate of oral lichen planus lesions. *Anticancer Research*, *26*(1A), 357–362. http://www.ncbi.nlm.nih.gov/pubmed/16475718

de Sousa, F. A. C. G., Paradella, T. C., Carvalho, Y. R., & Rosa, L. E. B. (2009). Comparative analysis of the expression of proliferating cell nuclear antigen, p53, bax, and bcl-2 in oral lichen planus and oral squamous cell carcinoma. *Annals of Diagnostic Pathology*, *13*(5), 308–312. https://doi.org/10.1016/j.anndiagpath.2009.06.001

Kilpi, A., Rich, A. M., Konttinen, Y. T., & Reade, P. C. (1995). The expression of c-erbB-2 protein in the keratinocytes of oral mucosal lichen planus. *The British Journal of Dermatology*, *133*(6), 847–852. https://doi.org/10.1111/j.1365-2133.1995.tb06915.x

Kilpi, A., Rich, A. M., Konttinen, Y. T., & Reade, P. C. (1996). Expression of c-erbB-2 protein in keratinocytes of oral mucosal lichen planus and subsequent squamous cell carcinoma. *European Journal of Oral Sciences*, *104*(3), 278–284. https://doi.org/10.1111/j.1600-0722.1996.tb00078.x

Montebugnoli, L., Farnedi, A., Marchetti, C., Magrini, E., Pession, A., & Foschini, M. P. (2006). High proliferative activity and chromosomal instability in oral lichen planus. *International Journal of Oral and Maxillofacial Surgery*, *35*(12), 1140–1144. https://doi.org/10.1016/j.ijom.2006.07.018

Montebugnoli, L., Gissi, D. B., Scapoli, L., Palmieri, A., Morandi, L., Manelli, I., & Foschini, M. P. (2014). p16(INK4) expression is not associated with human papillomavirus in oral lichen planus. *Oral Surgery, Oral Medicine, Oral Pathology and Oral Radiology*, *118*(6), 694–702. https://doi.org/10.1016/j.oooo.2014.09.004

Ogmundsdóttir, H. M., Hilmarsdóttir, H., Astvaldsdóttir, A., Jóhannsson, J. H., & Holbrook, W. P. (2002). Oral lichen planus has a high rate of TP53 mutations. A study of oral mucosa in icelanD. *European Journal of Oral Sciences*, *110*(3), 192–198. https://doi.org/10.1034/j.1600-0447.2002.21235.x

Shi, Y., Shen, L., & Yin, C. (2009). [Expressions of receptor-interacting protein and caspase-8 in oral squamous cell carcinoma and oral precancerous lesions]. *Nan Fang Yi Ke Da Xue Xue Bao = Journal of Southern Medical University*, *29*(9), 1802–1805. http://www.ncbi.nlm.nih.gov/pubmed/19778795

Zhang, W., Chen, Y., Geng, N., Tian, K., Bao, D., & Yang, M. (2006). [The role of matrix metalloproteinases and their tissue inhibitors in oral lichen planus]. *Zhonghua Kou Qiang Yi Xue Za Zhi = Zhonghua Kouqiang Yixue Zazhi = Chinese Journal of Stomatology*, *41*(7), 420–421. http://www.ncbi.nlm.nih.gov/pubmed/17067460

11.4 List S5. No distinction between oral, cutaneous and genital lichen planus (n=1)

Viguier M, Pérals C, Poirier B, Battistella M, Aubin F, Bachelez H, et al. Human papilloma virus-16-specific CD8+ T-cell expansions characterize different clinical forms of lichen planus and not lichen sclerosus et atrophicus. Exp Dermatol [Internet]. 2023 Jun;32(6):859–68. Available from: http://www.ncbi.nlm.nih.gov/pubmed/36922453
